# Five Decades of Citizen Science Reveal Erosion of Hotspots of Multifaceted Plant Diversity

**DOI:** 10.1111/gcb.71150

**Published:** 2026-10-27

**Authors:** Viktoria Ferenc, Cornelia Krause, Jörn Pagel, Michael Boxriker, Monika Voggesberger, Arno Wörz, Mike Thiv, Pierre Liancourt

**Affiliations:** ^1^ Department of Botany State Museum of Natural History Stuttgart Germany; ^2^ Institute of Landscape and Plant Ecology University of Hohenheim Stuttgart Germany; ^3^ KomBioTa—Center for Biodiversity and Integrative Taxonomy University of Hohenheim & State Museum of Natural History Stuttgart Germany; ^4^ Plant Ecology University of Tübingen Tübingen Germany

**Keywords:** diversity change, facets of diversity, floristic mapping, functional, incongruence, phylogenetic, taxonomic, very long‐term project

## Abstract

Biodiversity includes taxonomic, phylogenetic, and functional diversity but the changes in these facets at regional scales remain unclear and often context dependent. Using 2.5 million citizen science observations of 1957 vascular plant species across 53 years in SW‐Germany, we assessed changes in seven biodiversity metrics. Rather than a general decline, we found different facets exhibited different trends in both direction and magnitude of change. Phylogenetic and taxonomic diversity change were mostly negatively correlated, and change in functional diversity was independent from the others. We identified the role of climate and human activity influencing the change in taxonomic and functional diversity, while phylogenetic diversity was also affected by geodiversity. Crucially, we detected consistent erosion of biodiversity hotspots across all facets, while areas with low initial diversity tended to gain species. These findings highlight the need to consider multiple biodiversity facets and local context dependency to better understand biodiversity change at regional scales.

## Graphical abstract

Using 2.5 million citizen‐science records of 1957 plant species collected across southwest Germany over 53 years, we examined changes in species richness, evolutionary diversity, and plant functional traits. These facets changed independently and were explained by different environmental drivers: climate and human activity primarily shaped taxonomic and functional change, whereas phylogenetic change was additionally linked to geodiversity; nevertheless, biodiversity hotspots consistently lost diversity.

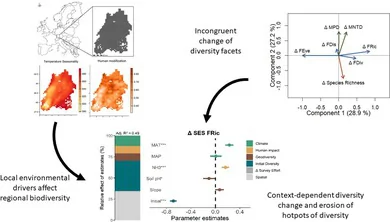

## 1 Introduction

Biodiversity is undergoing major changes globally, to the point where some consider it a potential sixth mass extinction (Barnosky et al. 2011; Pimm et al. 2014, but see Wiens and Saban 2025). However, studies have revealed that biodiversity change might be more nuanced than previously thought (Cardinale et al. 2018; Dornelas et al. 2014). The general decline of biodiversity remains heavily debated, with some arguing that the lack of precision in underlying databases and the bias towards short time series may prevent firm conclusions (Gonzalez et al. 2016; Vellend et al. 2017; Johnson et al. 2024). Moreover, the context‐dependency of these changes, the fact that some habitats or regions could be more vulnerable than others due to historical context, local climate or land use, can lead to idiosyncratic biodiversity trends, adds to the complexity of the problem (Vellend et al. 2017). Besides global and continental trends, understanding biodiversity change at regional scales is therefore crucial, as it is at these scales that conservation action is often implemented (e.g., regional red lists for endangered species or black lists for invasive species; IUCN 2012; Kowarik et al. 2003) and local context is more likely to influence trends (Vellend et al. 2017).

Importantly, biodiversity change is often assessed only in terms of taxonomic diversity (TD), particularly at large geographical scales, but a growing number of studies recognize its multifaceted nature (Cadotte et al. 2019; Le Bagousse‐Pinguet et al. 2021; IPBES 2025). Besides taxonomic diversity, two key facets of diversity are identified: phylogenetic diversity (PD), which reflects the evolutionary relationships among species (Webb et al. 2002; Cavender‐Bares et al. 2009), and functional diversity (FD), which measures the distribution of species' ecological strategies in multidimensional trait space (Villéger et al. 2008; Mouchet et al. 2010). Clear links have been established between these three facets (e.g., Cavender‐Bares et al. 2009). For instance, more species (higher TD) can lead to higher functional and phylogenetic diversity (Pavoine et al. 2013; Villéger et al. 2008). Likewise, if functional traits are phylogenetically conserved, closely related species should be functionally similar (Pavoine et al. 2013), leading to a positive correlation between analogous metrics of functional and phylogenetic diversity (Cadotte et al. 2019, e.g., functional and phylogenetic dispersion). That said, taxonomic, functional, and phylogenetic diversity are not necessarily redundant (E‐Vojtkó et al. 2023), and not all functional traits are tightly coupled to phylogeny. Such decoupling can produce divergent or idiosyncratic patterns in functional versus phylogenetic diversity (Cadotte et al. 2019; Mazel et al. 2018). Moreover, several independent aspects of functional diversity can be investigated (Mouchet et al. 2010). Functional diversity includes functional richness (FRic), evenness (FEve), divergence (FDiv), and dispersion (FDis) (Table 1). A positive correlation between taxonomic diversity (TD) and FRic is expected (but not with FEve), with more species contributing to higher functional richness (Mouchet et al. 2010). Altogether, this should result in three independent axes describing biodiversity: one axis combining TD and FRic; one dominated by FEve; and one by PD and FDiv—with functional dispersion (FDis) relating to both FRic and FDiv (Tucker et al. 2018; Mouchet et al. 2010). Finally, these diversity metrics and their relationships have been primarily explored for local communities; their application at a larger scale can produce signals inconsistent with local‐scale expectations (Gross et al. 2024; Oliveira et al. 2022; Hatfield et al. 2022; Franke et al. 2020; Qian et al. 2019; Jarzyna and Jetz 2018). It therefore remains unclear how congruent their temporal changes at regional scales are, given that each facet may respond differently to environmental drivers (Dornelas et al. 2023).

**TABLE 1:** Overview of the diversity metrics used to describe each diversity facet and their description.

Diversity facet	Diversity metric	Description
Taxonomic diversity	Number of species	Count of the total number of species per cell
Phylogenetic diversity	Mean Pairwise Distance (MPD)	Mean Pairwise Distance reflects phylogenetic diversity at the root of the phylogenetic tree (Webb et al. 2002)
	Mean Nearest Taxon Distance (MNTD)	Mean Nearest Taxon Distance reflects phylogenetic diversity at the tips of the phylogenetic tree (Webb et al. 2002)
Functional diversity	Functional Richness (FRic)	Functional Richness describes the functional space occupied by the species in the cell. It is larger, the larger the range between minimum and maximum trait values is (Villéger et al. 2008)
	Functional Evenness (FEve)	Functional Evenness describes how regularly traits are distributed within the functional space (Villéger et al. 2008)
	Functional Divergence (FDiv)	Functional Divergence represents how traits towards the extremes within the functional space are distributed (Villéger et al. 2008)
	Functional Dispersion (FDis)	Functional Dispersion describes the dispersion of traits within the trait space by measuring the distance of each trait to the center of all species in the cell (Laliberté and Legendre 2010)

Biodiversity and its different facets are strongly shaped by the combination of climate conditions (Gross et al. 2024; Hähn et al. 2025), the geodiversity (Dong et al. 2023), and land‐use intensity (Le Bagousse‐Pinguet et al. 2025), which together can influence both the magnitude and direction of biodiversity change. For instance, cold regions may be more vulnerable to species loss because they harbor cold‐adapted species that are especially sensitive to warming (Boxriker et al. 2025). In contrast, areas with high anthropogenic activity may even experience species gain (e.g., non‐native and range‐expanding nitrophilous species) (Eichenberg et al. 2021). Additionally, areas with high initial biodiversity, so‐called hotspots, may be particularly susceptible to change because they tend to host more range‐restricted and specialized species whose niches are more likely to erode under environmental pressures (Pinkert et al. 2025; Trew and Maclean 2021; Eichenberg et al. 2021; Le Roux et al. 2019). Accounting for these factors as potential sources of context dependency should provide invaluable insight into biodiversity change.

We use a unique dataset that sought to provide the complete checklist of vascular plant species of every 6.2 × 5.5‐km grid cell across SW‐Germany (Baden‐Württemberg). Baden‐Württemberg is estimated to contain 50%–66% of the vascular plant diversity of Germany (depending on how species lists are aggregated; Buttler et al. 2018; Wisskirchen and Haeupler 1998), and can therefore be considered representative of the Central European flora. Unlike large‐scale, unsupervised mapping projects based on the collection of data from multiple sources, the floristic mapping of Baden‐Württemberg aimed to record exhaustive species inventories. This was achieved through the joint effort of citizen science coordinated by professional botanists and providing expert validation, thereby capturing the composition of entire regional species pools. To investigate changes in biodiversity and how these might be explained by environmental factors, we use this dataset comprising more than 2.5 million observations of the vascular plant flora collected in two consecutive mappings covering 53 years. We specifically investigate (i) how has diversity changed and is the change of different facets of biodiversity congruent? (ii) what environmental factors can explain changes in different biodiversity facets?

## 2 Material and Methods

### 2.1 Floristic Mapping Data

We investigate changes in biodiversity over time and across diverse habitats using the floristic mapping of Baden‐Württemberg (Southwest Germany, 35,751 km^2^, Figure 1). The region is highly heterogeneous and comprises a wide range of environmental conditions (Figure 1). Elevation varies from 85 to 1493 m a.s.l.; Mean Annual Temperature (MAT) and Mean Annual Precipitation (MAP) vary from 4.8°C to 10.9°C and from 640 to 2000 mm respectively.

**FIGURE 1:**
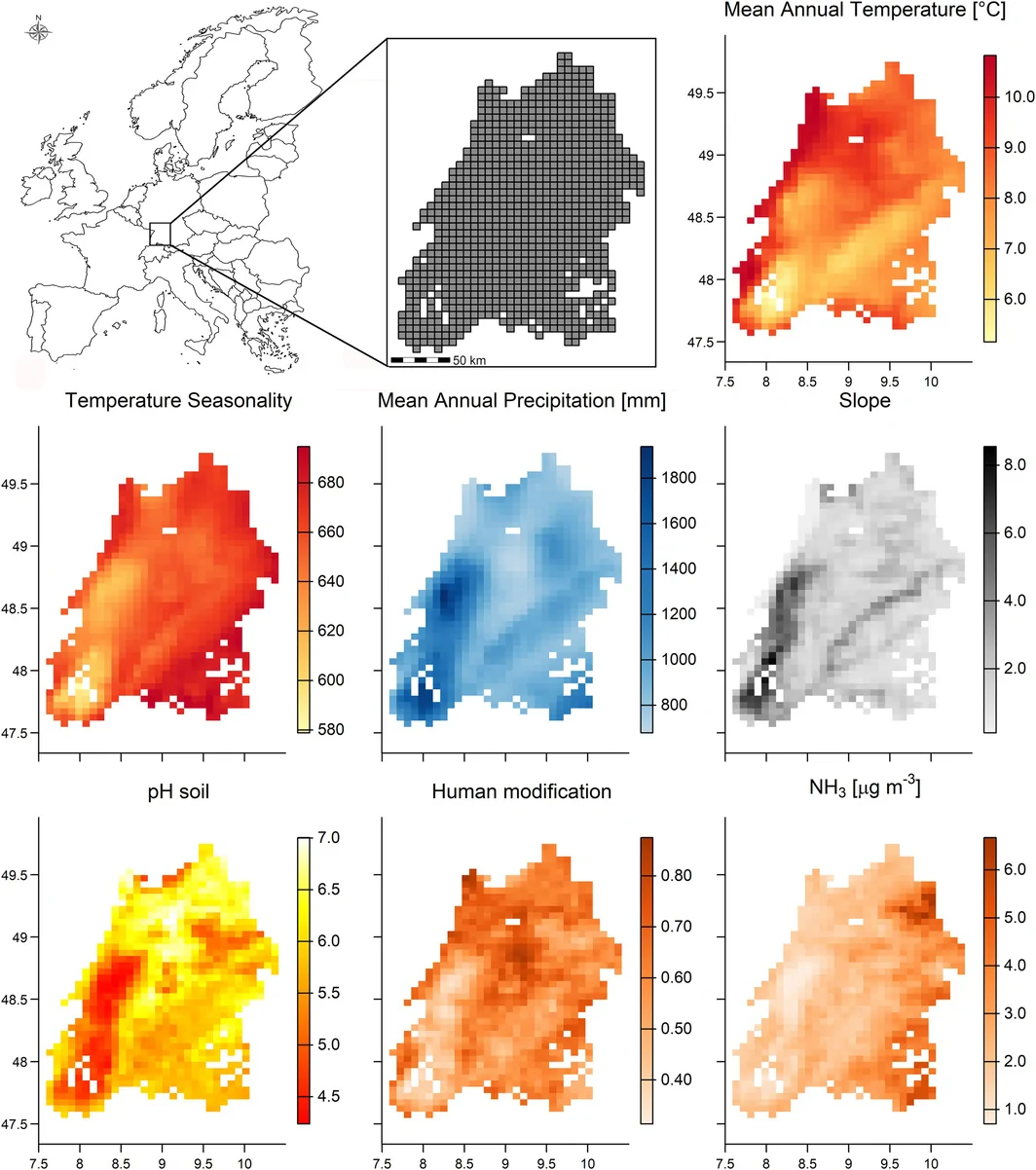
Location of the study area Baden‐Württemberg in Southwest Germany (top‐left panel). The enlarged image section on the right depicts the 906 raster cells colored in grey that were used for the analysis. The remaining seven panels visualize the spatial distribution of the used environmental variables (average values per cell, see Methods). Map lines delineate study areas and do not necessarily depict accepted national boundaries.

We use a comprehensive dataset from over 50 years of systematic floristic mapping, guided and coordinated by the State Natural History Museum Stuttgart involving more than 250 citizen scientists and professional botanists (see Wörz and Thiv 2015 for a more detailed description). Baden‐Württemberg was divided into 954 grid cells (6.2 × 5.5 km), where the occurrence of all species was continuously recorded during two distinct survey periods: the first from 1970 to 2000, when the survey was considered complete, and then starting a second survey from 2001 to 2023, with over 2.5 million plant species observations. Each grid cell was surveyed continuously over the entire survey interval instead of sequentially, so observations from each cell from both surveys are distributed across their respective periods; this minimizes temporal gaps between cells and ensures that the two floristic surveys are as comparable as possible (see Data S1, Figure S1). Our study is at a regional scale which we define as a grain size of the grid cells, with each containing multiple local communities, and a total extent covering the state of Baden‐Württemberg, SW‐Germany. Surveys sought to compile exhaustive species checklists, rather than partial samples, with multiple visits per cell to ensure comprehensive coverage and reduce the influence of seasonal fluctuations. The average survey effort per cell in the two survey periods was 57 and 69 observer visits per grid cell respectively.

All observations submitted by citizen scientists were validated by taxonomic experts from the museum before entering the database. Taxonomic harmonization was ensured using a local standardized species list (Wisskirchen and Haeupler 1998). Observations of crops were not considered. To facilitate analyses, all taxa were aggregated to species level, hybrids were removed and some species were aggregated (cf. Buttler et al. 2019) resulting in 1957 species in the dataset (details in Data S2). To identify potentially under‐sampled cells, we flagged any grid cell with fewer than 76 of the 100 most widespread species in either survey—a threshold set at the lowest 2.5% of the frequency distribution. Local experts then inspected these flagged cells, resulting in the exclusion of 5% of all cells. If a cell was deemed under‐sampled in either survey period, it was removed from both. This two‐step filter minimized the impact of differential sampling effort; including all cells in a control analysis did not alter our main results (see Data S1 for details). Details on the treatment of potentially under‐sampled cells and control analyses addressing potential survey effort biases are provided in Data S1. All further analyses are carried out using 906 cells comprising a total of 2,600,626 observations which corresponds to 916,570 unique observations (Figure 1).

### 2.2 Taxonomic, Phylogenetic and Functional Biodiversity

The three facets of diversity were quantified for each cell for the two survey periods. We described taxonomic diversity as species richness per cell. To assess phylogenetic diversity we used DaPhnE, a dated phylogeny of European plant species (Durka and Michalski 2012), pruned to our species of interest. Out of 25 species missing after taxonomic harmonization, 16 could be added based on TimeTree 5 (Kumar et al. 2022; Data S2). All phylogenetic analyses are based on 1948 species. Tree manipulations were done using the packages ‘picante’ (Kembel et al. 2010), ‘phytools’ (Revell 2012) and ‘TreeTools’ (Smith 2019). Phylogenetic diversity was assessed via phylogenetic dispersion (MPD) between species occurring in a given cell, measured in branch length, and mean nearest taxon distance (MNTD) (see also Table 1; Webb 2000; Webb et al. 2002). These metrics were calculated for each cell using the mpd and mntd functions in the ‘picante’ package (Kembel et al. 2010).

We used plant height, specific leaf area (SLA), and seed mass to capture functional diversity. These traits reflect key axes of plant ecological strategies (Díaz et al. 2016; Westoby 1998). Plant height reflects competition for light, with taller plants reaching better light conditions but requiring more resources for tissue growth and resource transport. SLA reflects resource‐use strategies, with low values showing conservative use and long‐lived leaves, while high values suggest exploitative strategies and higher photosynthetic rates. Seed mass reflects the trade‐off between growing heavier seeds with a higher chance of survival and seedling establishment and smaller seeds that increase the chance of successful spread (Liancourt et al. 2009; Westoby 1998).

We used the functional trait measures publicly available from TRY Database (Kattge et al. 2020; requested on March 1, 2023; version 5). We thinned down the dataset by removing all observations of juvenile or dead individuals, observations from above 1500 m a.s.l. (highest elevation of Baden‐Württemberg) and experimental data. To maximize trait data coverage, we included both leaf and leaflet measures. We used the average of all observations per species. We standardized all trait values as *Z*‐scores before further analyses to ensure comparability (Villéger et al. 2008). The traits were only weakly correlated, especially SLA with plant height and seed mass (Data S3, Figures S5 and S6) and multivariate phylogenetic signal for the three traits was statistically significant but weak (*K*_mult_ = 0.13, *p* = 0.001, Adams 2014).

Species without trait data comprise a small fraction of the composition of each grid cell. They occur in fewer cells than trait‐included species (84.4 vs. 310.9 cells in the first period). However, excluded species are distributed across multiple families, with no single clade dominating, and include a mix of trend categories (decliners, stable, increasers) that are broadly similar to trait‐included species. The mean proportion of trait‐complete species per grid cell was 88.8% for the first survey period and 88.4% for the second survey period. The mean change in per‐cell trait coverage from the first to the second survey period was—0.3%, indicating that trait coverage remained highly consistent between survey periods. Most importantly, the mean change in grid‐cell occupancy among excluded species is very low (+2.1 cells), indicating limited influence on overall patterns.

Functional diversity metrics were calculated using species with available data for plant height, SLA, and seed mass (1330 species). We assessed functional diversity with four commonly used diversity metrics (see Table 1) as multi‐trait measures based on plant height, SLA, and seed mass using the functions fd_fdis, fd_fdiv, fd_feve, fd_fric in the ‘fundiversity’ package (Grenié and Gruson 2023).

### 2.3 Environmental Data

To explain the observed pattern of plant diversity change in Baden‐Württemberg, we incorporated environmental variables of three groups: climate, geodiversity and human impact. For climate data we used the 19 bioclimatic variables (Data S3, Table S1) provided by WorldClim2 (Fick and Hijmans 2017). We calculated embergerQ, synthesizing temperature and humidity of climate (Emberger 1955), and thermal continentality from the bioclimatic variables with the package ‘ENVIREM’ (Title and Bemmels 2018). We assessed mean slope and soil pH per cell. Aspects of topography such as slope were shown to affect plant diversity and are predicted to interact with climate change (Frei et al. 2025; Cowles et al. 2018), as slope influences soil depth, water retention, erosion risk, and land‐use intensity (Frei et al. 2025; Lawrence et al. 2021). Variations in underlying bedrock composition play a critical role in shaping plant communities, interacting with precipitation variability (Palpurina et al. 2017; Nemer et al. 2021; Dong et al. 2023). We used elevation data to calculate the mean slope (steepness of the terrain) per cell with the package ‘spatialEco’ (Evans et al. 2023). Secondly, we extracted soil pH (soil water pH from 0 to 5 cm depth) from the SoilGrids 2.0 database at 250 m resolution (Poggio et al. 2021) as an approximation for soil type and weathering. We note that soil pH can vary in transition zones between different bedrock types which might not be fully captured by resampling methods of environmental data. We used atmospheric ammonia concentration (NH_3_) (LUBW 2023) to approximate agricultural impact. For human impact we used the ‘Human modification’ index which aggregates 13 variables to a continuous variable between 0 and 1, across five categories of anthropogenic stressors: human settlement, agriculture, transportation, mining and energy production, and electrical infrastructure (Kennedy et al. 2019). For all raster maps, matching resolutions were achieved using the resample function with bilinear interpolation of the package ‘terra’ (Hijmans 2023), which refines spatial data by weighting values from neighboring grid cells. We performed a Principal Component Analysis (PCA) on all 21 climate variables (Data S3, Figure S7) using the princomp function to reduce dimensionality. Three climate variables, Mean Annual Temperature, Mean Annual Precipitation and Temperature Seasonality, represented independent axes of variation and were selected and used together with the variables of geodiversity and human impact (Data S3, Figure S8). Figure 1 shows the spatial distribution of these seven environmental variables across the study area.

### 2.4 Statistical Approach

We standardized the phylogenetic and functional diversity metrics for variation in species richness by calculating standardized effect sizes (SES) using the output of null models to control for the mathematical dependence of diversity metrics and species richness. At our regional scale, SES values quantify departures from random assembly based on the entire regional species pool recorded across the entire study period (i.e., the two survey periods together, 53 years), thereby reflecting broad‐scale environmental and biogeographic filtering processes rather than local community assembly (i.e., competitive exclusion Webb et al. 2002). Null models were performed with the package ‘picante’ for phylogenetic diversity metrics by shuffling the species labels (Kembel et al. 2010). For functional diversity metrics, null models were run manually by generating 1000 random species assemblages per cell in which species were sampled with replacement and with probabilities proportional to their frequency of occurrence pooled across the two survey periods, thus retaining observed species richness while accounting for varying species prevalence. A control analysis with separate null models sampling the random species assemblages from their respective survey period yielded qualitatively similar results (data not shown). SES was then calculated as the deviation of observed values from the mean of the randomized metric distributions, divided by their standard deviation.

To explore congruence in diversity changes between the different facets of diversity, we performed a PCA on changes of the diversity metrics between the two surveys (e.g., ∆MPD = SES_MPD second mapping—SES_MPD first mapping) using the ‘principal’ function of the package ‘psych’ (Revelle 2024). We applied Horn's parallel analysis of the package ‘paran’ (Dinno 2024) to determine the dimensionality of the PCA followed by a varimax rotation (see loadings of the diversity metrics in Data S3, Table S2). Pairwise correlations among diversity metrics are provided in Data S3 (Figure S9).

For the second question regarding the influence of environmental factors on the seven biodiversity change metrics, we used all seven *z*‐scored environmental variables and the respective initial diversity measure for the first mapping as covariate (see Data S1 for additional analyses on the role of initial diversity). To account for possible differences in survey effort between the two mappings, we added ∆ Survey Effort to all models (∆ Survey Effort = Survey Effort Second Mapping—Survey Effort First Mapping). This approach assumes that changes in diversity metrics scale approximately linearly with changes in sampling effort. While this simplification may not capture more complex, non‐linear effects of effort, it controls for the primary bias expected from differences in survey intensity (see Data S1). To account for spatial autocorrelation, we used Moran's Eigenvector Maps (MEMs) that were calculated based on a first‐order queen contiguity spatial weight matrix among the grid cells using ‘spdep’ package (Bivand and Wong 2018). We retained only those positive MEMs that significantly reduced residual spatial autocorrelation in a forward procedure (Dray et al. 2025). We used LASSO regression for variable selection (Friedman et al. 2010; Tibshirani 1996) via the ‘glmnet’ package (Friedman et al. 2010), with all variables and retained MEMs standardized. The optimal penalty term (*λ*) was chosen through 10‐fold cross‐validation by minimizing mean squared error. Variables with non‐zero coefficients at the optimal *λ* were retained. We ran a GLM model for each diversity metric separately with the variables retained after LASSO and tested the residuals of the fitted models for spatial autocorrelation using Moran's *I*. All final models (except for ∆MNTD) showed no significant spatial structure in the residuals, confirming that the approach effectively controlled for spatial dependence. Variable importance was expressed as the percentage of variance explained, calculated by comparing the absolute value of each standardized regression coefficient to the total sum of all standardized regression coefficients in the model (Le Bagousse‐Pinguet et al. 2021). Variance inflation factors (VIF) for all variables retained in the final models were below commonly used thresholds for problematic multicollinearity (Data S3, Table S3). To assess the robustness of our findings to potential sampling bias, we applied the FRESCALO local frequency scaling method to correct species occurrence data for spatial and temporal variation in recorder effort. This correction did not alter our core results qualitatively, providing confidence in the reliability of our analyses (Data S1). All analyses were carried out in R version 4.6.0 (R Core Team 2026).

## 3 Results

### 3.1 Incongruent Change in Biodiversity Facets

The change in diversity was spatially incongruent for all diversity metrics (Figure 2, Data S3, Figure S9). The functional diversity metrics ∆FEve, ∆FDiv, and ∆FRic are correlated with Component 1, with ∆FEve negatively related to ∆FDiv and ∆FRic (Figure 2a). The phylogenetic diversity metrics ∆MPD and ∆MNTD are correlated with Component 2 and are negatively related to taxonomic diversity, the ∆Species richness (Figure 2a,b). ∆FDis is correlated with Component 3 (Figure 2b).

**FIGURE 2:**
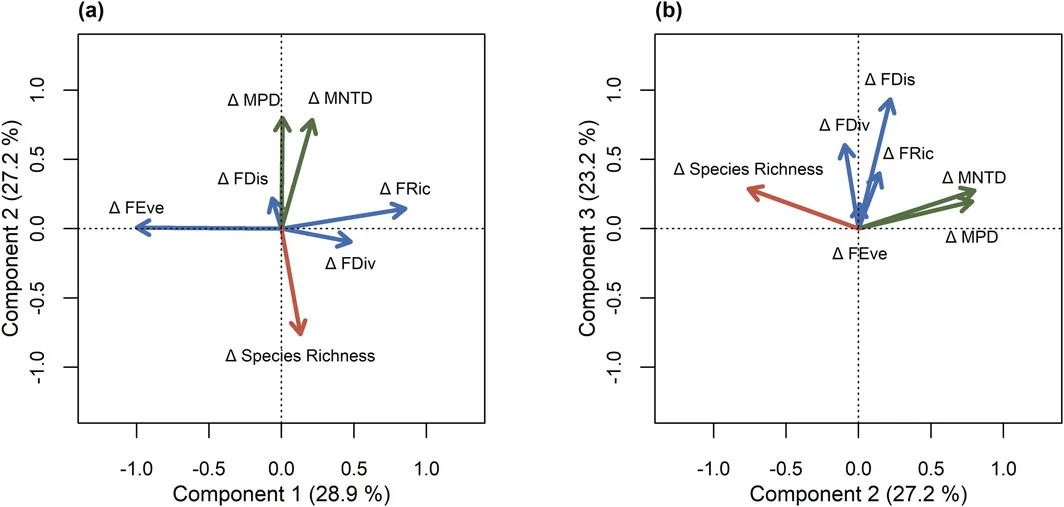
Principal Component Analysis followed by varimax rotation of the change between first and second mapping for all used diversity metrics (Species richness, FDis, Functional Dispersion; FDiv, Functional Divergence; FEve, Functional Evenness; FRic, Functional Richness; MNTD, Mean Nearest Taxon Distance; MPD, Mean Pairwise Distance). (a) First and second component (and proportion of variance explained), (b) second and third component (and proportion of variance explained). Colors of arrows indicate the facet of biodiversity (red—taxonomic diversity, green—phylogenetic diversity, blue—functional diversity). All PCA scores are normalized to improve visualization.

### 3.2 Context‐Dependency in Biodiversity Change

We showed that change in all biodiversity facets was clearly not uniform across the entire region. Instead, some locations increased, and others decreased in diversity. We also found that the change was not uniform across species either, although more species decreased than increased in number of cells in which they occur (1005 species and 878 species respectively, see Data S3, Tables S4 and S5). Inspection of increasing and decreasing species indicated that primarily nitrophilous, weedy, and non‐native species increased (half of the 50 most increasing species, Data S3, Table S4), while cold‐adapted or wetland species and species of high conservation value more commonly declined (of the 50 strongest decreasers about 25% belong to Orchidaceae, followed by Cyperaceae, Data S3, Table S5).

The LASSO regression, corrected for spatial autocorrelation, supported the high context‐dependency in the observed change over the last 50 years, and highlighted the influence of the local climate conditions, the level of human influence, and geodiversity (Figure 3). However, the relative contribution of these environmental factors varied depending on the biodiversity facet and metric considered. Importantly, across all facets and metrics, initial diversity always had the strongest effect on biodiversity change. Diversity declined in initially very diverse locations but often increased in initially low diverse locations (Figure 3). Using FRESCALO corrected data, the results are qualitatively unchanged (Data S1, Figure S4).

**FIGURE 3:**
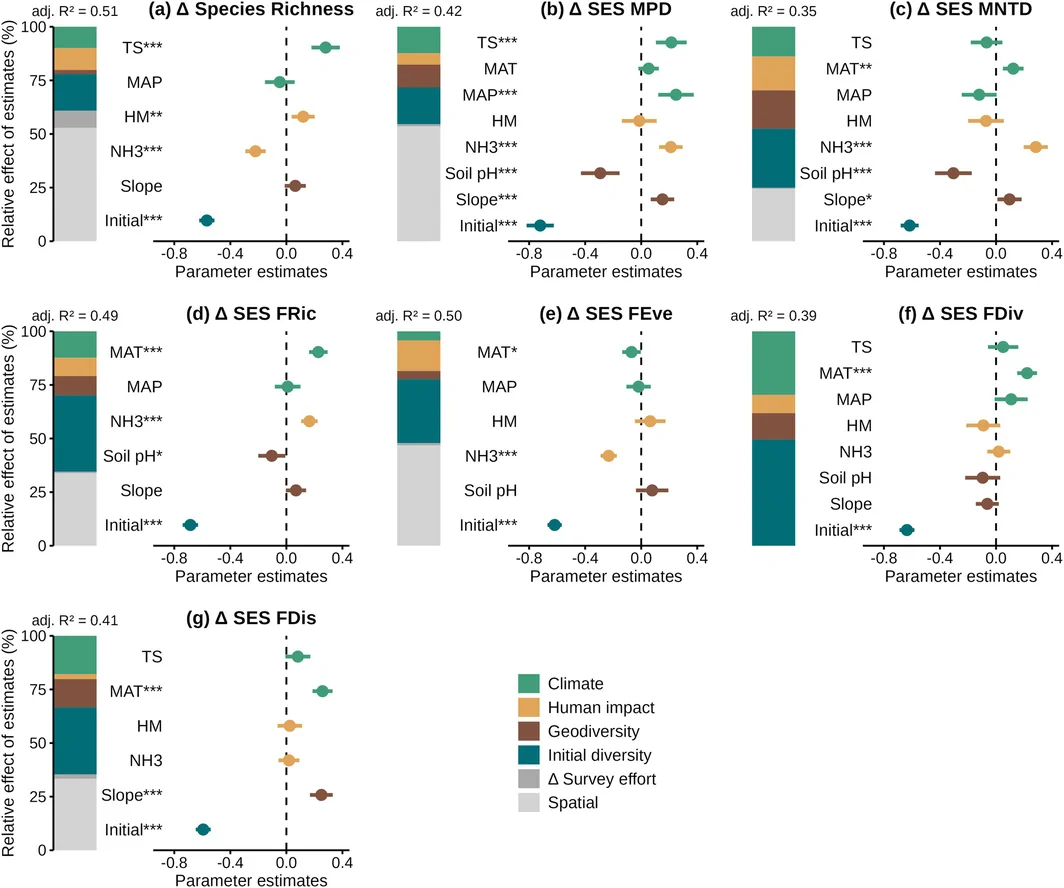
Results of models for the change of each diversity metric. Depicted are parameter estimates for each environmental variable (TS—Temperature Seasonality, MAP—Mean Annual Precipitation, MAT—Mean Annual Temperature, HM—Human Modification, NH_3_, Soil pH and Slope) on each ∆diversity metric (panel a) Taxonomic diversity: ∆Species Richness; panels (b) and (c) Phylogenetic diversity: ∆ SES MPD and ∆ SES MNTD; panels (d–g) Functional diversity: ∆ SES FRic, ∆ SES FEve, ∆ SES FDiv and ∆ SES FDis. The dots indicate mean and the lines standard deviation of the estimate. *p*‐values of each variable are given as: *p < 0.05; **p < 0.01; ***p < 0.001. To the left of each parameter estimate plot we additionally depict the relative effect of the estimates on the total variance explained by the variables. The variables were classified into six categories, each corresponding to a distinct color: TS, MAP and MAT in Climate (green), HM and NH_3_ in Human impact (yellow), Soil pH and Slope as Geodiversity (brown), the measure of initial diversity for each diversity metric respectively (blue), the change in survey effort between mappings (dark grey) and the sum of spatial eigenvectors as Spatial (light grey). The given adjusted *R* ^2^ values indicate the total variance explained by the model.

The change in taxonomic diversity (Figure 3a) was mostly explained by climatic variables, particularly temperature seasonality (higher annual temperature range led to greater diversity increase), and human impact, with high ammonia (NH_3_) levels leading to a decrease and high human modification to an increase. Change in both phylogenetic metrics was mostly explained by the same environmental variables (Figure 3b,c). Both metrics were positively associated with NH_3_ and steeper slopes and negatively associated with soil pH. They differed in relevant climate variables, with ∆MPD positively related to temperature seasonality and MAP, while ∆MNTD was positively associated with MAT.

Different environmental variables explained the change in each of the four functional diversity metrics as well. ∆FRic (Figure 3d) was primarily influenced by positive effects of MAT and NH_3_, with locations having higher values increasing in FRic (broader trait range). Conversely, ∆FEve (Figure 3e) was mainly explained by a negative effect of NH_3_ and MAT, with high MAT and NH_3_ levels leading to lower Functional Evenness (more clustered trait values). ∆FDiv (Figure 3f) was mainly influenced by climate, with a positive effect of MAT. High temperature leads to greater Functional Divergence, indicating more distinct trait values. ∆FDis (Figure 3g) is mainly explained by climate and geodiversity, with locations having higher temperature and steeper slopes increasing Functional Dispersion, reflecting greater trait variation.

## 4 Discussion

Our study reveals that biodiversity change over the past 50 years varies in direction and magnitude across diversity facets, highlighting their incongruence and complementarity for regional‐scale assessments of biodiversity change. These changes were not uniform across the study area, and all diversity facets showed a high degree of context‐dependency, with gains and losses influenced by climate and land use.

More species declined than increased, consistent with recent national observations (Jandt et al. 2022). Yet, biodiversity change was closely linked to initial diversity, echoing the global pattern of higher vulnerability in biodiversity hotspots (Trew and Maclean 2021; Eichenberg et al. 2021; Le Roux et al. 2019). We were surprised to see that in contrast, locations with initially low diversity tended to gain species. Species that increase include many widespread, nitrophilous, weedy, disturbance‐tolerant and neophytic taxa (see also Nielsen et al. 2019; Wörz and Thiv 2015, Data S3, Tables S4 and S5). Declining species, by contrast, are often range‐restricted or habitat specialists, including glacial relict taxa (Boxriker et al. 2025). This combination of losses among narrowly distributed species and gains among widespread or expanding ones is consistent with processes that drive biotic homogenization and suggests an ongoing shift from specialized, high‐quality communities toward more generalized, disturbance‐adapted assemblages and biodiversity degradation (McKinney and Lockwood 1999; Daru et al. 2021).

Instead of the expected positive correlation, we found TD and PD changes to be negatively correlated over time, indicating that species richness declines were accompanied by increases in phylogenetic diversity. This pattern aligns with Knapp et al. (2017) but contrasts with other studies reporting positive or decoupled relationships (Barber et al. 2017; Qian et al. 2023). Such a negative correlation may result from the loss of phylogenetically redundant species, which increases average branch lengths and thus phylogenetic diversity despite fewer species. An alternative explanation is the colonization of cells by phylogenetically distant non‐native species, which would also increase phylogenetic diversity. A more precise inspection of the compositional changes would be necessary to verify the cause underlying our pattern.

Functional diversity changes revealed additional complexity. We also found that some functional diversity metrics (∆FRic, ∆FEve, ∆FDiv) were independent from taxonomic and phylogenetic diversity change. This is only partly consistent with previous work on phylogenetic and functional diversity (Cadotte et al. 2019; Hähn et al. 2025). Notably, while previous studies underline potential decoupling of functional and phylogenetic diversity depending on the traits and phylogenetic clades investigated (E‐Vojtkó et al. 2023; Mazel et al. 2018), a recent review found ubiquitous congruence between traits and phylogeny (Cadotte et al. 2019). A simulation study found that the correlation between phylogenetic and functional diversity strengthens with more traits, but varies by trait optima (as with our traits reflecting different ecological strategies) (Tucker et al. 2018). Likewise, numerical simulations found for instance that FRic, FEve and FDiv define three relatively independent components of functional diversity (Mouchet et al. 2010). Instead, our results showed that ∆FEve was negatively related to ∆FRic and ∆FDiv, indicating a decreased functional redundancy with the loss of functional richness and functional divergence (loss of extreme traits). Thus, with functional richness and divergence declining, trait space contracts while evenness increases. This highlights a broader issue within biodiversity research: the lack of operational clarity when ‘biodiversity’ is used as a general term as each facet offers unique insights and may follow distinct patterns (e.g., E‐Vojtkó et al. 2023), particularly for functional diversity metrics, which depend strongly on the choice of traits.

The commonly observed relationship among species richness, phylogenetic and functional diversity metrics, likely differs from our results for several reasons. Firstly, studies comparing different facets of diversity have often focused on local communities (see Cadotte et al. 2019 for review). Unlike ours, they usually include species abundance to weight trait values in the calculation of the metrics. Secondly, regional biodiversity changes observed here contrast with local community expectations, where coexistence stabilizes diverse assemblages (Tilman et al. 2014; Hatton et al. 2024) and non‐native species often locally reduce richness yet increase it regionally (Vellend et al. 2017). Such mechanisms are less relevant at regional scales, where species rarely co‐occur (McGill 2010). This can lead to mismatched signals depending on spatial grain. For instance, functional and phylogenetic shifts may be more visible at local scales (e.g., loss of particular strategies), while diversity structure could be maintained across larger scales. The discrepancies found between local scale studies and ours also illustrate that assembly processes, particularly those related to competitive coexistence, cannot be used directly to infer patterns of diversity change at large biogeographical scales, despite the similarity of the metrics and analytical approaches (see McGill 2010).

Besides initial diversity, environmental context matters (Gonzalez et al. 2016; Vellend et al. 2017). All changes in diversity facets were explained by environmental variables, though the identity, magnitude, and direction of effects differed between facets. Climate variables explained biodiversity change. Temperature seasonality, the difference between winter and summer temperatures, was positively linked to changes in species richness and phylogenetic dispersion (∆MPD) and marginally positive to functional dispersion (∆FDis). Milder winters and reduced temperature differences may drive the loss of cold‐adapted species, as shown by those with the greatest declines (Data S3, Table S5; Boxriker et al. 2025). In our analysis, Mean Annual Temperature and, to a lesser extent, Mean Annual Precipitation were found to explain changes in phylogenetic and functional but not taxonomic diversity. This could be attributed to species turnover, where the species composition shifts across different temperature and precipitation regimes while maintaining the same total number of species.

Taxonomic, phylogenetic, and functional metrics also responded differently to human impact (Lopez et al. 2018). The human modification index only influenced taxonomic diversity. However, atmospheric NH_3_ had a broader influence on diversity metrics (changes in taxonomic diversity, ∆MNTD and ∆FEve, and to a lesser extent, ∆MPD and ∆FRic). Higher NH_3_ seems to reduce species richness while increasing phylogenetic diversity, illustrating the aforementioned loss of phylogenetically redundant species. For functional diversity, higher NH_3_ increased ∆FRic but decreased ∆FEve; this expands the trait space but reduces trait evenness, likely because nitrogen enrichment favors fast‐growing, nitrogen‐demanding species (Wörz and Thiv 2015). ∆FDiv and ∆FDis are unaffected by NH_3_, confirming that changes in ∆FRic and ∆FEve are not due to species with extreme traits (Mouchet et al. 2010). Altogether, this supports previous findings showing areas with high agricultural land use are more prone to changes (Le Bagousse‐Pinguet et al. 2025).

Finally, geodiversity also had an effect on phylogenetic and functional diversity metrics. We observed an increase in phylogenetic diversity in locations with more acidic soils, a trend not mirrored by taxonomic or functional diversity (except ∆FRic). Species characteristic of these soil types are vulnerable to global changes (Harásek et al. 2023) while variations in underlying bedrock composition may interact with precipitation variability (Nemer et al. 2021; Dong et al. 2023). This may favor species from certain lineages, increasing phylogenetic diversity. We found a positive effect of steeper slopes on phylogenetic and functional diversity change (∆FDis and ∆FRic). This might be explained by areas with topographic heterogeneity hosting high species diversity and their inaccessibility to land‐use intensification, serving as refugia for species (Frei et al. 2025; Lawrence et al. 2021; Ronk et al. 2020).

Interpreting biodiversity change requires caution, especially when the species occurrence records are aggregated from multiple sources (atlases, monographs, checklists, museum collections) and multiple data providers or were overall collected in an unsupervised manner. Such occurrence records could be sensitive to the attractiveness of the region or its accessibility (Geldmann et al. 2016; Daru et al. 2018). However, the floristic mapping of Baden‐Württemberg, being a supervised standardized effort, designed to be exhaustive and conducted by a single institution, is less likely to suffer from this problem (Petřík et al. 2010). Altogether, accounting for the difference in survey effort between mappings as covariate, using standardized effect sizes from a null model approach and additionally performing a FRESCALO correction to explicitly account for variation in recorder effort, all consistently showed that a possible sampling bias would not qualitatively affect the observed pattern.

## 5 Conclusions

Leveraging over 50 years of comprehensive, expertly validated botanical surveys, our study provides detailed documentation of biodiversity change across taxonomic, phylogenetic, and functional diversity at a regional scale. We found that declines in diversity have been particularly pronounced in species‐rich hotspots, highlighting regional differences and the importance of context dependency in biodiversity change. The environmental factors influencing diversity change varied across facets. Our findings highlight the complementary nature of diversity facets, where maximizing taxonomic diversity may reduce phylogenetic diversity, and vice versa. Importantly, our long‐term data allow for the identification of priority areas—such as hotspots for functional or phylogenetic diversity—that may warrant particular conservation attention. This underscores the imperative of examining the change in multiple diversity facets rather than relying solely on species richness, which may obscure important aspects of biodiversity trajectories across space and time.

## Author Contributions

**Viktoria Ferenc:** conceptualization, methodology, investigation, formal analysis, writing – original draft, writing – review and editing, visualization, data curation. **Cornelia Krause:** data curation, writing – review and editing, project administration. **Jörn Pagel:** formal analysis, writing – review and editing. **Michael Boxriker:** formal analysis, writing – review and editing. **Monika Voggesberger:** data curation. **Arno Wörz:** data curation, project administration. **Mike Thiv:** data curation, writing – review and editing, project administration. **Pierre Liancourt:** conceptualization, methodology, investigation, formal analysis, supervision, writing – review and editing, writing – original draft.

## Funding

This work was supported by the Ministry of Science, Research and Arts Baden‐Württemberg and the Landesanstalt für Umwelt Baden‐Württemberg. P.L. was partly supported by the 111 Project (D23029).

## Conflicts of Interest

The authors declare no conflicts of interest.

## Supporting information


**Figure S1:** Temporal coverage and number of observations across mapping periods. (a and b) Barcode plots illustrate the distribution of years with at least one plant record for 100 randomly selected grid cells under two scenarios. Maximal approach (a): every year in each reported time range is included. Conservative approach (b): only range endpoints and explicit single years are counted. Barcode plots display years sampled (dots, colored by mapping period: blue = first, red = second). (c) Barplot shows the number of unique observations for the first and second mapping periods.
**Figure S2:** Scatterplot of change in sampling effort (∆ Survey Effort = Survey Effort Second Mapping—Survey Effort First Mapping) and initial taxonomic diversity (number of species in first mapping).
**Figure S3:** Comparison of raw occurrence data and FRESCALO‐corrected occurrence probabilities. Panel (a) presents density curves of raw and effort‐corrected species richness changes, illustrating that the FRESCALO adjustment notably moderates the extremes of the distribution. This correction reduces the magnitude of the most pronounced changes, which are likely to be influenced by observer bias. Panel (b) shows a linear regression comparing raw and corrected species richness changes per site, highlighting a strong positive correlation between the two.
**Figure S4:** Results of the multiple regression model for the change in taxonomic diversity using FRESCALO‐corrected species richness as the dependent variable. The right panel shows parameter estimates (mean and standard deviation) for each environmental variable: TS (Temperature Seasonality), MAP (Mean Annual Precipitation), MAT (Mean Annual Temperature), HM (Human Modification), NH_3_ and Soil pH. *p*‐values are indicated as: *p < 0.05; **p < 0.01; ***p < 0.001. The left barplot depicts the relative effect of each variable group on the total variance explained, classified into climate (green), human impact (yellow), geodiversity (brown), initial diversity (blue), change in survey effort (dark grey), and spatial variables (light grey). The adjusted *p* ^2^ value indicates the variance explained by the model.

**Data S2:** Supporting Information.

**Figure S5:** Pearson's correlation matrix of the used traits.
**Figure S6:** PCA of the used traits.
**Figure S7:** PCA of all 21 climate variables.
**Figure S8:** PCA of all used environmental variables.
**Figure S9:** Pearson's correlation matrix of the Δ diversity metrics.
**Table S1:** List of the 19 bioclimatic variables provided by WorldClim 2 (Fick and Hijmans 2017).
**Table S2:** Loadings of the Δ diversity metrics in the three components after applying a varimax rotation to the corresponding PCAs. Numbers depicted in bold indicate the highest contribution of the Δ diversity metric.
**Table S3:** Variance Inflation Factor (VIF) for all variables retained in the respective model.
**Table S4:** Species that have increased most between the first and second mapping.
**Table S5:** Species that have decreased most between the first and second mapping.

## Data Availability

The data that support the findings of this study are available on figshare, doi: 10.6084/m9.figshare.30618800.
