# Supplementary material for: Five Decades of Citizen Science Reveal Erosion of Hotspots of Multifaceted Plant Diversity
Source: Glob Chang Biol. 2026 Oct 27;32(10):e71150. doi: 10.1111/gcb.71150 (PMC13711242; doi:10.1111/gcb.71150)
Supplement: Supplementary file 1 [file GCB-32-e71150-s001.pdf]

## Supporting Information 1

# Five decades of Citizen Science reveal erosion of hotspots of multifaceted plant diversity

Viktoria Ferenc[*1,2,3], viktoria.ferenc@smns-bw.de, +49 711-8936203
Cornelia Krause[1,3], cornelia.krause@smns-bw.de
Jörn Pagel[2,3], joern.pagel@uni-hohenheim.de
Michael Boxriker[1,3], michael.boxriker@smns-bw.de
Monika Voggesberger[1], monika.voggesberger@smns-bw.de
Arno Wörz[1], awoerz@gmx.de
Mike Thiv[1,3], mike.thiv@smns-bw.de
Pierre Liancourt[1,3,4], pierre.liancourt@smns-bw.de

[1]Department of Botany, State Museum of Natural History, Stuttgart, Germany
[2]Institute of Landscape and Plant Ecology, University of Hohenheim, Stuttgart, Germany
[3]KomBioTa – Center for Biodiversity and Integrative Taxonomy, University of Hohenheim & State Museum of Natural History, Stuttgart, Germany
[4]Plant Ecology, University of Tübingen, Tübingen, Germany

*Corresponding author

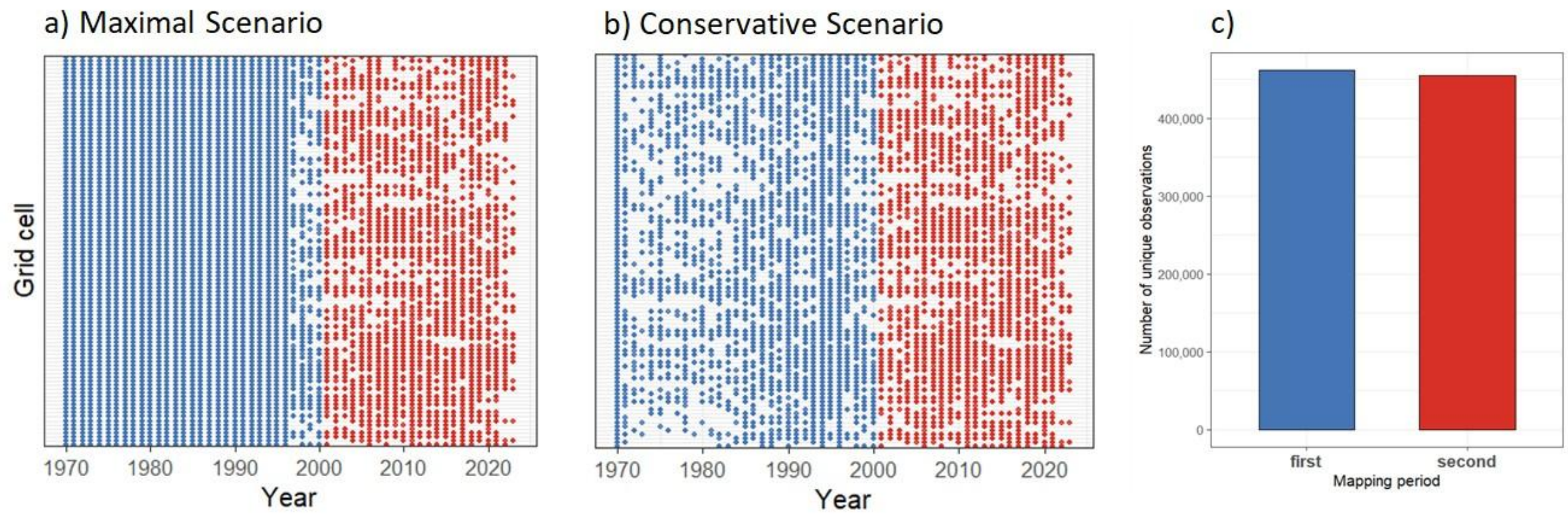


**Figure S1.** Temporal coverage and number of observations across mapping periods. (a and b) Barcode plots illustrate the distribution of years with at least one plant record for 100 randomly selected grid cells under two scenarios: Maximal approach (a): every year in each reported time range is included. Conservative approach (b): only range endpoints and explicit single years are counted. Barcode plots display years sampled (dots, colored by mapping period: blue = first, red = second). (c) Barplot shows the number of unique observations for the first and second mapping periods.

Identification of Potentially Undersampled Grid Cells

We applied a two-step filtering approach to identify grid cells with insufficient sampling. First, we compiled a list of the 100 most frequently recorded species across both mapping periods. Grid cells in which fewer than 76 of these widespread and conspicuous species were recorded were considered potentially undersampled. This threshold corresponds to the lowest 2.5% of the frequency distribution and led to the exclusion of 4 cells from the first mapping (0.4%) and 45 from the second mapping (4.7%), amounting to 5% of all cells. This objective criterion was complemented by expert judgement: regional coordinators of the floristic mapping, with long-term knowledge of local flora and survey coverage, supported the classification of these cells as potentially undersampled. This conservative approach minimizes the influence of differential sampling effort between the two mappings.

To confirm that this filtering did not bias our results, we conducted a control analysis including all grid cells regardless of their sampling completeness. The results were qualitatively unchanged, indicating that the exclusion of potentially undersampled cells did not influence the main conclusions (data not shown).

Quantifying and Evaluating Sampling Effort Differences

To evaluate changes in sampling effort between mapping periods (Bowler et al. 2023, Ecography, https://doi.org/10.1111/ecog.06219), we calculated the number of unique observer–date–cell combinations for each cell and mapping period as a proxy for survey intensity. We then computed the difference (Δ Survey Effort) between the two periods. A regression analysis revealed no significant relationship between Δ Survey Effort and initial species richness, indicating no directional bias in resurvey intensity (Supporting Information 1, Fig. S2).

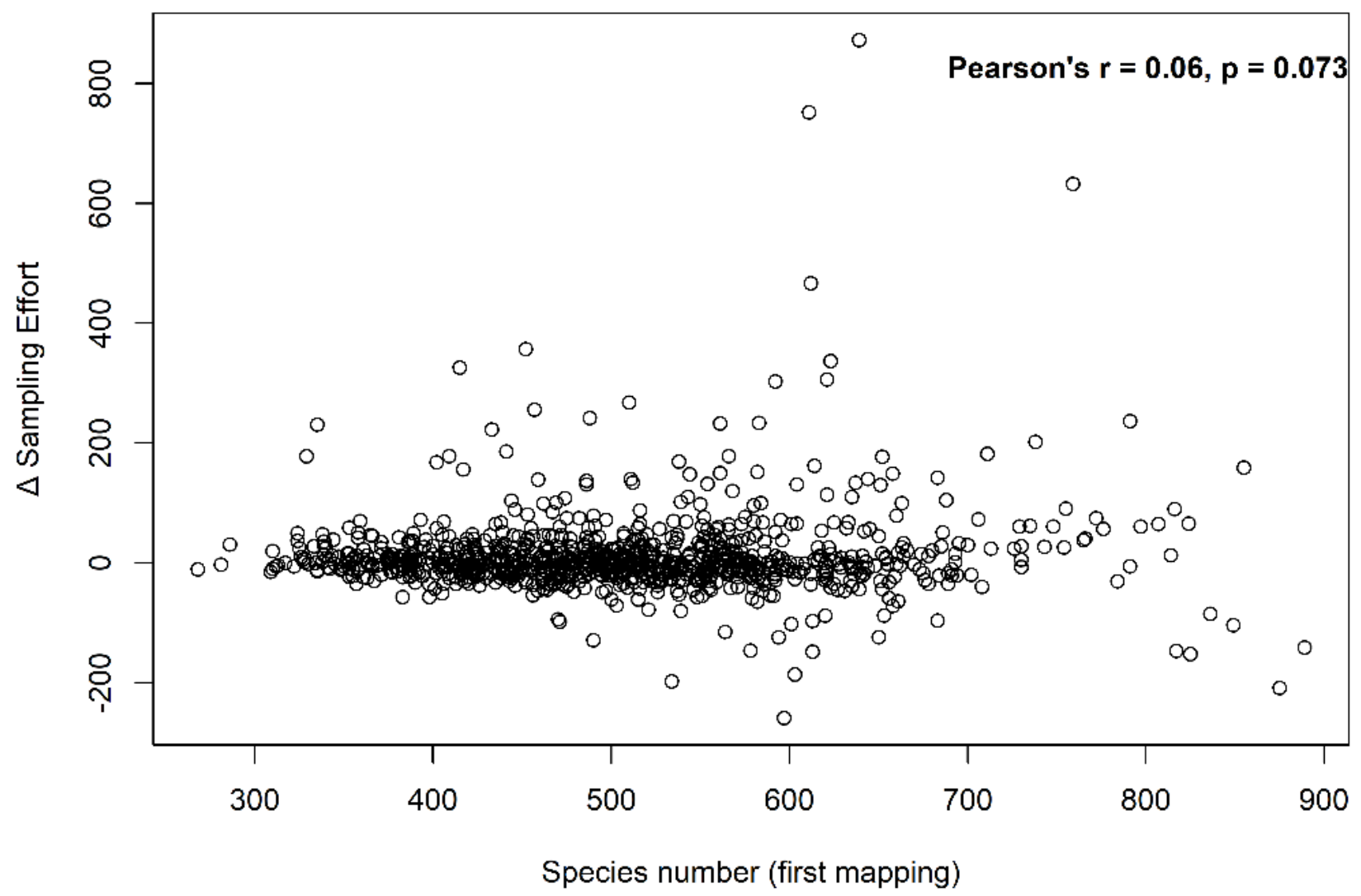


**Figure S2.** Scatterplot of change in sampling effort (Δ Survey Effort = Survey Effort Second Mapping – Survey Effort First Mapping) and initial taxonomic diversity (Number of species in first mapping).

Correcting for Sampling Bias Using FRESCALO

To evaluate the robustness of our results to variation in recorder effort, we applied the FRESCALO algorithm (Hill, 2012, MEE, https://doi.org/10.1111/j.2041-210X.2011.00146.x) to the plant occurrence data. This method estimates and adjusts for spatial and temporal biases in sampling intensity using local frequency scaling. Performing this complementary analysis allowed us to verify that accounting for heterogeneous survey effort did not qualitatively alter our main conclusions. We therefore provide this analysis as a quality control and methodological validation. Geographic coordinates (latitude and longitude) were obtained as centroids for each grid cell, and pairwise geodesic distances between cells were calculated. Environmental similarity was quantified using 26 environmental variables, including bioclimatic indices, soil pH, elevation, and human modification index (see Methods and Supporting Information 3, Figs. S7, S8). A combined distance matrix was constructed by normalizing and equally weighting geographic and environmental distances to define ecologically coherent neighbourhoods. For each focal cell, the 10 nearest neighbours in this combined space were identified. Neighbourhood weights were derived using inverse distance weighting, normalized to sum to one, reflecting both spatial proximity and environmental similarity.

Within each neighbourhood, FRESCALO estimates the sampling intensity multiplier (alpha) iteratively to standardize local species frequency curves to a target sampling intensity. Benchmark species were identified as those with the top 27% of standardized species frequency ranks in each neighbourhood. These benchmarks form the basis for assessing local recorder effort. Temporal correction involves calculating the proportion of benchmark species recorded in each grid cell for each mapping period, serving as a proxy for local sampling effort. Species-specific time factors are then estimated by fitting observed occurrences against model expectations adjusted by sampling intensity. These time factors quantify relative changes in species frequencies between mappings, allowing corrected occurrence probabilities to be computed per species, site, and mapping.

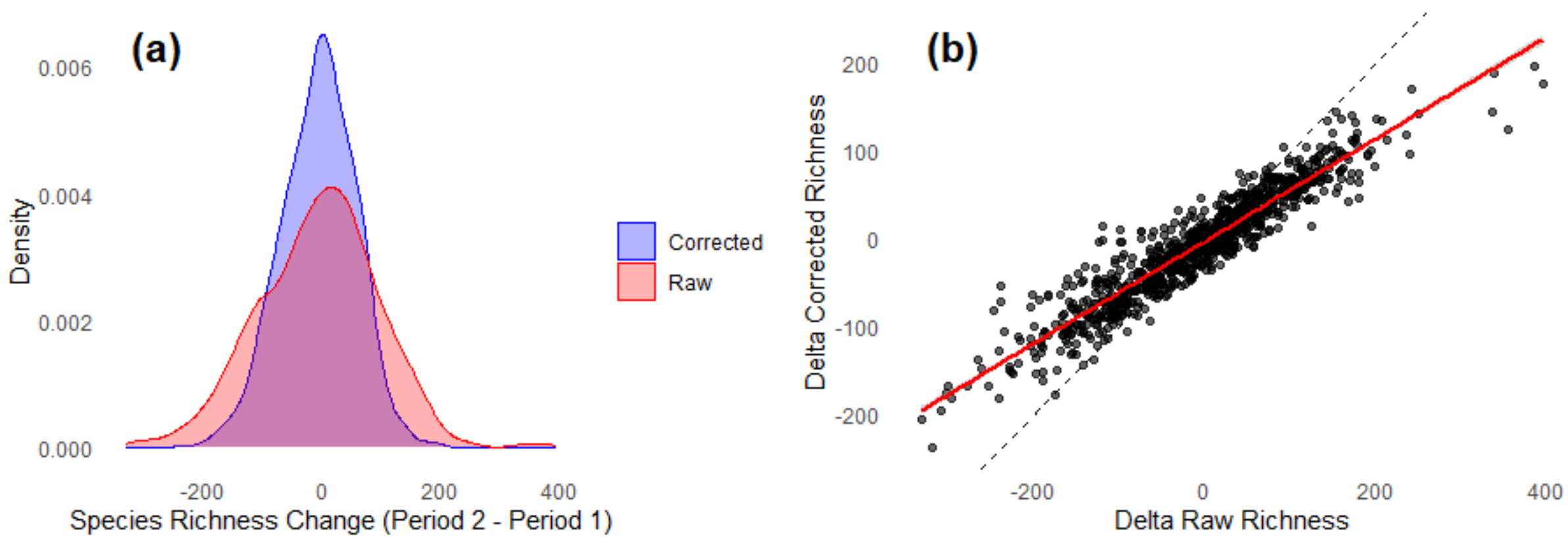


**Figure S3.** Comparison of raw occurrence data and FRESCALO-corrected occurrence probabilities. Panel (a) presents density curves of raw and effort-corrected species richness changes, illustrating that the FRESCALO adjustment notably moderates the extremes of the distribution. This correction reduces the magnitude of the most pronounced changes, which are likely to be influenced by

observer bias. Panel (b) shows a linear regression comparing raw and corrected species richness changes per site, highlighting a strong positive correlation between the two.

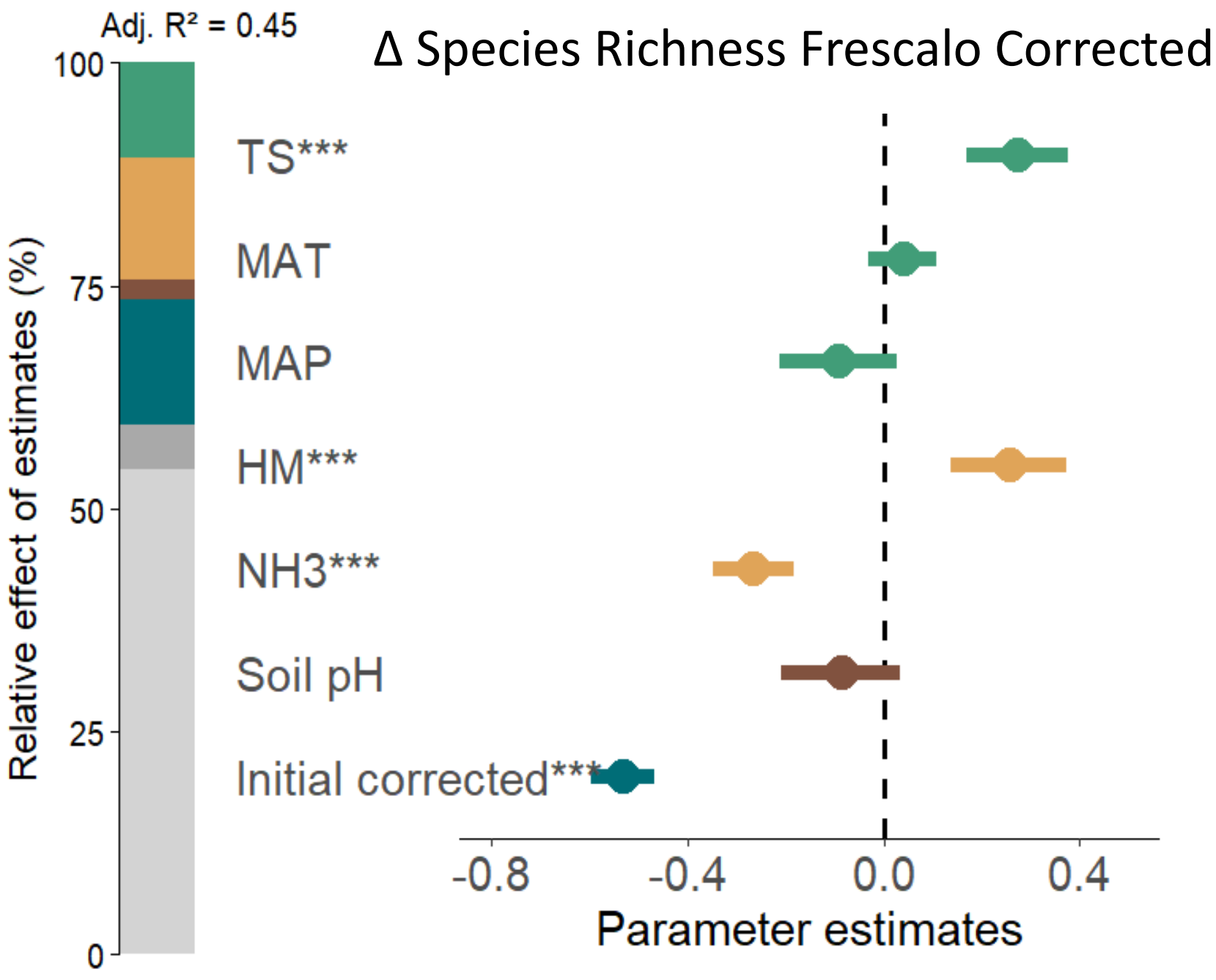


**Figure S4.** Results of the multiple regression model for the change in taxonomic diversity using FRESCALO-corrected species richness as the dependent variable. The right panel shows parameter estimates (mean and standard deviation) for each environmental variable: TS (Temperature Seasonality), MAP (Mean Annual Precipitation), MAT (Mean Annual Temperature), HM (Human Modification), $NH_3$ and Soil pH. P-values are indicated as: *$P < 0.05$; **$P < 0.01$; ***$P < 0.001$. The left barplot depicts the relative effect of each variable group on the total variance explained, classified into climate (green), human impact (yellow), geodiversity (brown), initial diversity (blue), change in survey effort (dark grey), and spatial variables (light grey). The adjusted $R^2$ value indicates the variance explained by the model.
