# Supplementary material for: Five Decades of Citizen Science Reveal Erosion of Hotspots of Multifaceted Plant Diversity
Source: Glob Chang Biol. 2026 Oct 27;32(10):e71150. doi: 10.1111/gcb.71150 (PMC13711242; doi:10.1111/gcb.71150)
Supplement: Supplementary file 2 [file GCB-32-e71150-s002.pdf]

originally recorded species identity	species identity	author	included in taxonomic_diversity	included in phylogenetic_diversity	included in functional_diversity	reference/reason for species aggregation	reference or synonym for addition to daphne	synonym in TRY
Abies alba	*Abies alba*	Mill.	yes	yes	yes			
Abutilon theophrasti	*Abutilon theophrasti*	Medik.	yes	yes	no			
Acer campestre	*Acer campestre*	L.	yes	yes	yes			
Acer campestre subsp. campestre	*Acer campestre*	L.	yes	yes	yes	aggregate on species level		
Acer negundo	*Acer negundo*	L.	yes	yes	yes			
Acer opalus	*Acer opalus*	Mill.	yes	yes	yes			
Acer platanoides	*Acer platanoides*	L.	yes	yes	yes			
Acer pseudoplatanus	*Acer pseudoplatanus*	L.	yes	yes	yes			
Acer saccharinum	*Acer saccharinum*	L.	yes	yes	no		Gao et al 2020, added to node N4580	
Aceras anthropophorum	*Aceras anthropophorum*	(L.) W. T. Aiton	yes	yes	yes			*Orchis anthropophora*
Achillea collina	*Achillea collina*	Becker ex Rchb.	yes	yes	yes			
Achillea filipendulina	*Achillea filipendulina*	Lam.	yes	yes	no		Ehrendorfer and Guo 2006, added to node N7889	
Achillea millefolium	*Achillea millefolium*	L.	yes	yes	yes			
Achillea millefolium agg.	*Achillea millefolium*	L.	yes	yes	yes	aggregate on species level		

Achillea millefolium f. rosea	*Achillea millefolium*	L.	yes	yes	yes	aggregate on species level		
Achillea millefolium subsp. millefolium	*Achillea millefolium*	L.	yes	yes	yes	aggregate on species level		
Achillea nobilis	*Achillea nobilis*	L.	yes	yes	no			
Achillea nobilis subsp. nobilis	*Achillea nobilis*	L.	yes	yes	no	aggregate on species level		
Achillea pratensis	*Achillea millefolium*	L.	yes	yes	no	taxonomic inconsistencies between first and second mapping		
Achillea ptarmica	*Achillea ptarmica*	L.	yes	yes	yes			
Achillea ptarmica agg.	*Achillea ptarmica*	L.	yes	yes	yes	aggregate on species level		
Acinos arvensis	*Acinos arvensis*	(Lam.) Dandy	yes	yes	yes			*Clinopodium acinos*
Aconitum lycoctonum	*Aconitum lycoctonum*	L.	yes	yes	yes			
Aconitum lycoctonum subsp. lycoctonum	*Aconitum lycoctonum*	L.	yes	yes	yes	aggregate on species level		
Aconitum lycoctonum subsp. vulparia	*Aconitum lycoctonum*	L.	yes	yes	yes	aggregate on species level		
Aconitum napellus	*Aconitum napellus*	L.	yes	yes	yes			
Aconitum napellus subsp. napellus	*Aconitum napellus*	L.	yes	yes	yes	aggregate on species level		

Aconitum tauricum	*Aconitum tauricum*	(Wulfen) Gáyer	yes	yes	no			
Aconitum variegatum	*Aconitum variegatum*	L.	yes	yes	no			
Aconitum variegatum subsp. variegatum	*Aconitum variegatum*	L.	yes	yes	no	aggregate on species level		
Aconogonon polystachyum	*Aconogonon polystachyum*	Wall. ex Meisn.	yes	yes	no			
Acorus calamus	*Acorus calamus*	L.	yes	yes	yes			
Actaea spicata	*Actaea spicata*	L.	yes	yes	yes			
Adenostyles alliariae	*Adenostyles alliariae*	(Gouan) Kern.	yes	yes	yes			
Adenostyles glabra	*Adenostyles glabra*	(Mill.) DC.	yes	yes	no			
Adonis aestivalis	*Adonis aestivalis*	L.	yes	yes	yes			
Adonis annua	*Adonis annua*	L.	yes	yes	no			
Adonis flammea	*Adonis flammea*	Jacq.	yes	yes	no			
Adonis flammea subsp. flammea	*Adonis flammea*	Jacq.	yes	yes	no	aggregate on species level		
Adoxa moschatellina	*Adoxa moschatellina*	L.	yes	yes	yes			
Aegopodium podagraria	*Aegopodium podagraria*	L.	yes	yes	yes			
Aesculus hippocastanum	*Aesculus hippocastanum*	L.	yes	yes	yes			
Aethusa cynapium	*Aethusa cynapium*	L.	yes	yes	yes			
Aethusa cynapium subsp. cynapium	*Aethusa cynapium*	L.	yes	yes	yes	aggregate on species level		
Aethusa cynapium subsp. elata	*Aethusa cynapium*	L.	yes	yes	yes	aggregate on species level		
Agrimonia eupatoria	*Agrimonia eupatoria*	L.	yes	yes	yes			
Agrimonia eupatoria subsp. eupatoria	*Agrimonia eupatoria*	L.	yes	yes	yes	aggregate on species level		
Agrimonia procera	*Agrimonia procera*	Wallr.	yes	yes	yes			

Agropyron cristatum subsp. pectinatum	*Agropyron cristatum*	(M. Bieb.) Tzvelev	yes	yes	no	aggregate on species level	Gallaher et al 2022, added to node N1720	
Agrostemma githago	*Agrostemma githago*	L.	yes	yes	yes			
Agrostis canina	*Agrostis canina*	L.	yes	yes	yes			
Agrostis canina agg.	*Agrostis canina*	L.	yes	yes	yes	aggregate on species level		
Agrostis capillaris	*Agrostis capillaris*	L.	yes	yes	yes			
Agrostis gigantea	*Agrostis gigantea*	Roth	yes	yes	yes			
Agrostis stolonifera	*Agrostis stolonifera*	L.	yes	yes	yes			
Agrostis stolonifera agg.	*Agrostis stolonifera*	L.	yes	yes	yes	aggregate on species level		
Agrostis vinealis	*Agrostis vinealis*	Schreb.	yes	yes	yes			
Ailanthus altissima	*Ailanthus altissima*	(Mill.) Swingle	yes	yes	no			
Aira caryophyllea	*Aira caryophyllea*	L.	yes	yes	yes			
Aira caryophyllea subsp. caryophyllea	*Aira caryophyllea*	L.	yes	yes	yes	aggregate on species level		
Aira praecox	*Aira praecox*	L.	yes	yes	yes			
Ajuga chamaepitys	*Ajuga chamaepitys*	(L.) Schreb.	yes	yes	yes			
Ajuga genevensis	*Ajuga genevensis*	L.	yes	yes	yes			
Ajuga pyramidalis	*Ajuga pyramidalis*	L.	yes	yes	yes			
Ajuga reptans	*Ajuga reptans*	L.	yes	yes	yes			
Alcea rosea	*Alcea rosea*	L.	yes	yes	yes			

Alchemilla connivens	*Alchemilla vulgaris*	L.	yes	yes	yes	part of A. vulgaris-complex (Sebald et al. 1992)		
Alchemilla coriacea	*Alchemilla vulgaris*	L.	yes	yes	yes	part of A. vulgaris-complex (Sebald et al. 1992)		
Alchemilla crinita	*Alchemilla vulgaris*	L.	yes	yes	yes	part of A. vulgaris-complex (Sebald et al. 1992)		
Alchemilla decumbens	*Alchemilla vulgaris*	L.	yes	yes	yes	part of A. vulgaris-complex (Sebald et al. 1992)		
Alchemilla effusa	*Alchemilla vulgaris*	L.	yes	yes	yes	part of A. vulgaris-complex (Sebald et al. 1992)		

Alchemilla filicaulis	*Alchemilla vulgaris*	L.	yes	yes	yes	part of A. vulgaris-complex (Sebald et al. 1992)		
Alchemilla glabra	*Alchemilla vulgaris*	L.	yes	yes	yes	part of A. vulgaris-complex (Sebald et al. 1992)		
Alchemilla glaucescens	*Alchemilla vulgaris*	L.	yes	yes	yes	part of A. vulgaris-complex (Sebald et al. 1992)		
Alchemilla impexa	*Alchemilla vulgaris*	L.	yes	yes	yes	part of A. vulgaris-complex (Sebald et al. 1992)		
Alchemilla lineata	*Alchemilla vulgaris*	L.	yes	yes	yes	part of A. vulgaris-complex (Sebald et al. 1992)		

Alchemilla micans	*Alchemilla vulgaris*	L.	yes	yes	yes	part of A. vulgaris-complex (Sebald et al. 1992)		
Alchemilla mollis	*Alchemilla vulgaris*	L.	yes	yes	yes	part of A. vulgaris-complex (Sebald et al. 1992)		
Alchemilla mollis agg.	*Alchemilla vulgaris*	L.	yes	yes	yes	part of A. vulgaris-complex (Sebald et al. 1992)		
Alchemilla monticola	*Alchemilla vulgaris*	L.	yes	yes	yes	part of A. vulgaris-complex (Sebald et al. 1992)		
Alchemilla nitida	*Alchemilla vulgaris*	L.	yes	yes	yes	part of A. vulgaris-complex (Sebald et al. 1992)		

Alchemilla obtusa	*Alchemilla vulgaris*	L.	yes	yes	yes	part of A. vulgaris-complex (Sebald et al. 1992)		
Alchemilla plicata	*Alchemilla vulgaris*	L.	yes	yes	yes	part of A. vulgaris-complex (Sebald et al. 1992)		
Alchemilla propinqua	*Alchemilla vulgaris*	L.	yes	yes	yes	part of A. vulgaris-complex (Sebald et al. 1992)		
Alchemilla reniformis	*Alchemilla vulgaris*	L.	yes	yes	yes	part of A. vulgaris-complex (Sebald et al. 1992)		
Alchemilla straminea	*Alchemilla vulgaris*	L.	yes	yes	yes	part of A. vulgaris-complex (Sebald et al. 1992)		

Alchemilla strigosula	*Alchemilla vulgaris*	L.	yes	yes	yes	part of A. vulgaris-complex (Sebald et al. 1992)		
Alchemilla subcrenata	*Alchemilla vulgaris*	L.	yes	yes	yes	part of A. vulgaris-complex (Sebald et al. 1992)		
Alchemilla vulgaris	*Alchemilla vulgaris*	L.	yes	yes	yes	part of A. vulgaris-complex (Sebald et al. 1992)		
Alchemilla vulgaris agg.	*Alchemilla vulgaris*	L.	yes	yes	yes	part of A. vulgaris-complex (Sebald et al. 1992)		
Alchemilla xanthochlora	*Alchemilla vulgaris*	L.	yes	yes	yes	part of A. vulgaris-complex (Sebald et al. 1992)		
Alisma gramineum	*Alisma gramineum*	Lej.	yes	yes	no			
Alisma lanceolatum	*Alisma lanceolatum*	With.	yes	yes	no			

Alisma plantago-aquatica	*Alisma plantago-aquatica*	L.	yes	yes	yes			*ALISMA PLANTAGO-AQUATICA*
Alisma plantago-aquatica agg.	*Alisma plantago-aquatica*	L.	yes	yes	yes	aggregate on species level		
Alliaria petiolata	*Alliaria petiolata*	(M. Bieb.) Cavara & Grande	yes	yes	yes			
Allium angulosum	*Allium angulosum*	L.	yes	yes	no			
Allium carinatum	*Allium carinatum*	L.	yes	yes	yes			
Allium carinatum subsp. carinatum	*Allium carinatum*	L.	yes	yes	yes	aggregate on species level		
Allium carinatum subsp. pulchellum	*Allium carinatum*	L.	yes	yes	yes	aggregate on species level		
Allium cepa	*Allium cepa*	L.	yes	yes	no			
Allium fistulosum	*Allium fistulosum*	L.	yes	yes	no			
Allium nigrum	*Allium nigrum*	L.	yes	yes	no			
Allium oleraceum	*Allium oleraceum*	L.	yes	yes	yes			
Allium paradoxum	*Allium paradoxum*	(M. Bieb.) G. Don	yes	yes	yes			
Allium sativum	*Allium sativum*	L.	yes	yes	no			
Allium sativum ssp. ophioscorodon	*Allium sativum*	L.	yes	yes	no	aggregate on species level		
Allium schoenoprasum	*Allium schoenoprasum*	L.	yes	yes	yes			
Allium schoenoprasum var. alpinum	*Allium schoenoprasum*	L.	yes	yes	yes	aggregate on species level		
Allium scorodoprasum agg.	*Allium scorodoprasum*	L.	yes	yes	no	aggregate on species level		

Allium scorodoprasum subsp. rotundum	*Allium scorodoprasum*	L.	yes	yes	no	aggregate on species level		
Allium scorodoprasum subsp. scorodoprasum	*Allium scorodoprasum*	L.	yes	yes	no	aggregate on species level		
Allium senescens	*Allium senescens*	L.	yes	yes	yes		*Allium lusitanicum*	
Allium senescens subsp. montanum	*Allium senescens*	(Fr.) Holub	yes	yes	yes	aggregate on species level	*Allium lusitanicum*	
Allium sphaerocephalon	*Allium sphaerocephalon*	L.	yes	yes	yes			
Allium suaveolens	*Allium suaveolens*	Jacq.	yes	yes	no			
Allium ursinum	*Allium ursinum*	L.	yes	yes	yes			
Allium vineale	*Allium vineale*	L.	yes	yes	yes			
Alnus alnobetula	*Alnus alnobetula*	(Ehrh.) K. Koch	yes	yes	yes			
Alnus glutinosa	*Alnus glutinosa*	(L.) P. Gaertn.	yes	yes	yes			
Alnus incana	*Alnus incana*	(L.) Moench	yes	yes	yes			
Alopecurus aequalis	*Alopecurus aequalis*	Sobol.	yes	yes	yes			
Alopecurus geniculatus	*Alopecurus geniculatus*	L.	yes	yes	yes			
Alopecurus myosuroides	*Alopecurus myosuroides*	Huds.	yes	yes	yes			
Alopecurus pratensis	*Alopecurus pratensis*	L.	yes	yes	yes			
Alopecurus pratensis agg.	*Alopecurus pratensis*	L.	yes	yes	yes	aggregate on species level		
Alopecurus pratensis subsp. pratensis	*Alopecurus pratensis*	L.	yes	yes	yes	aggregate on species level		
Alopecurus pratensis subsp. pseudonigricans	*Alopecurus pratensis*	L.	yes	yes	yes	aggregate on species level		
Althaea hirsuta	*Althaea hirsuta*	L.	yes	yes	no			

Althaea officinalis	*Althaea officinalis*	L.	yes	yes	yes			
Alyssum alyssoides	*Alyssum alyssoides*	(L.) L.	yes	yes	yes			
Alyssum montanum	*Alyssum montanum*	L.	yes	yes	yes			
Alyssum montanum subsp. gmelinii	*Alyssum montanum*	L.	yes	yes	yes	aggregate on species level		
Alyssum montanum subsp. montanum	*Alyssum montanum*	L.	yes	yes	yes	aggregate on species level		
Amaranthus albus	*Amaranthus albus*	L.	yes	yes	no			
Amaranthus blitoides	*Amaranthus blitoides*	S. Watson	yes	yes	yes			
Amaranthus blitum	*Amaranthus blitum*	L.	yes	yes	yes			
Amaranthus blitum subsp. blitum	*Amaranthus blitum*	L.	yes	yes	yes	aggregate on species level		
Amaranthus blitum subsp. emarginatus	*Amaranthus blitum*	L.	yes	yes	yes	aggregate on species level		
Amaranthus blitum var. emarginatus	*Amaranthus blitum*	L.	yes	yes	yes	aggregate on species level		
Amaranthus blitum var. oleraceus	*Amaranthus blitum*	L.	yes	yes	yes	aggregate on species level		
Amaranthus blitum var. pseudogracilis	*Amaranthus blitum*	L.	yes	yes	yes	aggregate on species level		
Amaranthus bouchonii	*Amaranthus bouchonii*	Thell.	yes	yes	no			
Amaranthus caudatus	*Amaranthus caudatus*	L.	yes	yes	yes			
Amaranthus cruentus	*Amaranthus cruentus*	L.	yes	yes	no			
Amaranthus deflexus	*Amaranthus deflexus*	L.	yes	yes	no			
Amaranthus graecizans	*Amaranthus graecizans*	L.	yes	yes	no			

Amaranthus graecizans subsp. silvestris	*Amaranthus graecizans*	L.	yes	yes	no	aggrega te on species level			
Amaranthus hybridus	*Amaranthus hybridus*	L.	yes	yes	no		*Amara nthus lusitani cum*		
Amaranthus hybridus agg.	*Amaranthus hybridus*	L.	yes	yes	no	aggrega te on species level	*Amara nthus lusitani cum*		
Amaranthus hypochondriacus	*Amaranthus hypochondriacus*	L.	yes	yes	no				
Amaranthus powellii	*Amaranthus powellii*	S. Watson	yes	yes	no				
Amaranthus retroflexus	*Amaranthus retroflexus*	L.	yes	yes	yes				
Ambrosia artemisiifolia	*Ambrosia artemisiifolia*	L.	yes	yes	yes				
Ambrosia coronopifolia	*Ambrosia coronopifolia*	NA	yes	yes	yes			*Ambro sia psilost achya*	
Amelanchier lamarckii	*Amelanchier lamarckii*	F. G. Schroed.	yes	yes	yes				
Amelanchier ovalis	*Amelanchier ovalis*	Medik.	yes	yes	yes				
Amorpha fruticosa	*Amorpha fruticosa*	L.	yes	yes	yes				
Anacamptis pyramidalis	*Anacamptis pyramidalis*	(L.) Rich.	yes	yes	yes				
Anagallis arvensis	*Anagallis arvensis*	L.	yes	yes	yes				
Anagallis arvensis subsp. arvensis	*Anagallis arvensis*	L.	yes	yes	yes	aggrega te on species level			
Anagallis foemina	*Anagallis foemina*	Mill.	yes	yes	yes				
Anagallis minima	*Anagallis minima*	L.	yes	yes	yes		*Centun culus minimu s*		
Anagallis tenella	*Anagallis tenella*	(L.) L.	yes	yes	yes				
Anaphalis margaritacea	*Anaphalis margaritacea*	(L.) Benth.	yes	yes	no				

Anchusa arvensis	*Anchusa arvensis*	(L.) M. Bieb.	yes	yes	yes			
Anchusa arvensis subsp. arvensis	*Anchusa arvensis*	(L.) M. Bieb.	yes	yes	yes	aggregate on species level		
Anchusa azurea	*Anchusa azurea*	Mill.	yes	yes	yes			*Anchusa arvensis*
Anchusa officinalis	*Anchusa officinalis*	L.	yes	yes	yes			
Anchusa officinalis agg.	*Anchusa officinalis*		yes	yes	yes	aggregate on species level		
Andromeda polifolia	*Andromeda polifolia*	L.	yes	yes	yes			
Andromeda polifolia subsp. polifolia	*Andromeda polifolia*	L.	yes	yes	yes	aggregate on species level		
Androsace lactea	*Androsace lactea*	L.	yes	yes	no			
Androsace septentrionalis	*Androsace septentrionalis*	L.	yes	yes	yes			
Anemone blanda	*Anemone blanda*	Schott & Kotschy	yes	yes	no			
Anemone narcissiflora	*Anemone narcissiflora*	L.	yes	yes	yes			
Anemone nemorosa	*Anemone nemorosa*	L.	yes	yes	yes			
Anemone ranunculoides	*Anemone ranunculoides*	L.	yes	yes	yes			
Anemone sylvestris	*Anemone sylvestris*	L.	yes	yes	no			
Anethum graveolens	*Anethum graveolens*	L.	yes	yes	yes			
Angelica archangelica	*Angelica archangelica*	L.	yes	yes	yes			
Angelica archangelica subsp. archangelica	*Angelica archangelica*	L.	yes	yes	yes	aggregate on species level		
Angelica sylvestris	*Angelica sylvestris*	L.	yes	yes	yes			
Angelica sylvestris subsp. sylvestris	*Angelica sylvestris*	L.	yes	yes	yes	aggregate on species level		

Antennaria dioica	*Antennaria dioica*	(L.) P. Gaertn.	yes	yes	yes			
Anthemis arvensis	*Anthemis arvensis*	L.	yes	yes	yes			
Anthemis austriaca	*Anthemis austriaca*	Jacq.	yes	yes	no			
Anthemis cotula	*Anthemis cotula*	L.	yes	yes	yes			
Anthemis ruthenica	*Anthemis ruthenica*	M. Bieb.	yes	yes	yes			
Anthemis tinctoria	*Anthemis tinctoria*	L.	yes	yes	yes			*Cota tinctoria*
Anthemis tinctoria agg.	*Anthemis tinctoria*	L.	yes	yes	yes	aggregate on species level		
Anthemis tinctoria subsp. tinctoria	*Anthemis tinctoria*	L.	yes	yes	yes	aggregate on species level		
Anthericum liliago	*Anthericum liliago*	L.	yes	yes	yes			
Anthericum ramosum	*Anthericum ramosum*	L.	yes	yes	yes			
Anthoxanthum alpinum	*Anthoxanthum alpinum*	Å. Loeve & D. Loeve	yes	yes	yes			*Anthoxanthum odoratum*
Anthoxanthum aristatum	*Anthoxanthum aristatum*	Boiss.	yes	yes	yes			
Anthoxanthum odoratum	*Anthoxanthum odoratum*	L.	yes	yes	yes			
Anthoxanthum odoratum agg.	*Anthoxanthum odoratum*	L.	yes	yes	yes	aggregate on species level		
Anthriscus caucalis	*Anthriscus caucalis*	M. Bieb.	yes	yes	yes			
Anthriscus cerefolium	*Anthriscus cerefolium*	(L.) Hoffm.	yes	yes	yes			
Anthriscus cerefolium var. cerefolium	*Anthriscus cerefolium*	(L.) Hoffm.	yes	yes	yes	aggregate on species level		
Anthriscus cerefolium var. trichocarpus	*Anthriscus cerefolium*	(L.) Hoffm.	yes	yes	yes	aggregate on species level		

Anthriscus nitidus	*Anthriscus nitidus*	(Wahlenb.) Hazsl.	yes	yes	no		*Anthriscus nitida*			
Anthriscus sylvestris	*Anthriscus sylvestris*	(L.) Hoffm.	yes	yes	yes					
Anthriscus sylvestris agg.	*Anthriscus sylvestris*	(L.) Hoffm.	yes	yes	yes	aggregate on species level				
Anthriscus sylvestris subsp. stenophyllus	*Anthriscus sylvestris subsp. stenophyllus*	(Rouy & Camus) Briq.	yes	yes	no	ecologically and morphologically clearly distinct from other ssp. (Wörz 1992)	Kumar et al 2022, sistertaxon to A. sylvestris			
Anthriscus sylvestris subsp. sylvestris	*Anthriscus sylvestris*	(L.) Hoffm.	yes	yes	yes	aggregate on species level				
Anthyllis vulneraria	*Anthyllis vulneraria*	L.	yes	yes	yes					
Anthyllis vulneraria subsp. alpestris	*Anthyllis vulneraria*	L.	yes	yes	yes	aggregate on species level				
Anthyllis vulneraria subsp. alpicola	*Anthyllis vulneraria*	L.	yes	yes	yes	aggregate on species level				
Anthyllis vulneraria subsp. carpatica	*Anthyllis vulneraria*	L.	yes	yes	yes	aggregate on species level				
Anthyllis vulneraria subsp. polyphylla	*Anthyllis vulneraria*	L.	yes	yes	yes	aggregate on species level				
Anthyllis vulneraria subsp. pseudovulneraria	*Anthyllis vulneraria*	L.	yes	yes	yes	aggregate on species level				

Anthyllis vulneraria subsp. vulneraria	*Anthyllis vulneraria*	L.	yes	yes	yes	aggregate on species level		
Antirrhinum majus	*Antirrhinum majus*	L.	yes	yes	yes			
Apera interrupta	*Apera interrupta*	(L.) P. Beauv.	yes	yes	yes			
Apera spica-venti	*Apera spica-venti*	(L.) P. Beauv.	yes	yes	yes			*APERA SPICA-VENTI*
Aphanes arvensis	*Aphanes arvensis*	L.	yes	yes	yes			
Aphanes inexspectata	*Aphanes inexspectata*	W. Lippert	yes	yes	yes		*Aphanes australis*	*Aphanes australis*
Apium graveolens	*Apium graveolens*	L.	yes	yes	yes			
Apium nodiflorum	*Apium nodiflorum*	(L.) Lag.	yes	yes	yes			*Helosciadium nodiflorum*
Apium repens	*Apium repens*	(Jacq.) Lag.	yes	yes	no			
Aposeris foetida	*Aposeris foetida*	(L.) Less.	yes	yes	yes			
Aquilegia atrata	*Aquilegia atrata*	W. D. J. Koch	yes	yes	no			
Aquilegia vulgaris	*Aquilegia vulgaris*	L.	yes	yes	no			
Aquilegia vulgaris agg.	*Aquilegia vulgaris*	L.	yes	yes	no	aggregate on species level		
Aquilegia vulgaris subsp. vulgaris	*Aquilegia vulgaris*	L.	yes	yes	no	aggregate on species level		
Arabidopsis thaliana	*Arabidopsis thaliana*	(L.) Heynh.	yes	yes	yes			
Arabis alpina	*Arabis alpina*	L.	yes	yes	yes			
Arabis alpina agg.	*Arabis alpina*	L.	yes	yes	yes	aggregate on species level		
Arabis auriculata	*Arabis auriculata*	Lam.	yes	yes	no			
Arabis glabra	*Arabis glabra*	L.	yes	yes	yes			*Turritis glabra*
Arabis hirsuta	*Arabis hirsuta*	(L.) Scop.	yes	yes	yes			

Arabis hirsuta agg.	*Arabis hirsuta*	(L.) Scop.	yes	yes	yes	aggregate on species level		
Arabis nemorensis	*Arabis nemorensis*	(Hoffm.) Koch	yes	yes	no			
Arabis pauciflora	*Arabis pauciflora*	(Grimm) Garcke	yes	yes	no			
Arabis sagittata	*Arabis sagittata*	(Bertol.) DC.	yes	yes	yes			
Arabis turrita	*Arabis turrita*	L.	yes	yes	yes			*Pseudo turritis turrita*
Arctium lappa	*Arctium lappa*	L.	yes	yes	yes			
Arctium minus	*Arctium minus*	NA	yes	yes	yes			
Arctium nemorosum	*Arctium nemorosum*	Lej.	yes	yes	no			
Arctium tomentosum	*Arctium tomentosum*	Mill.	yes	yes	yes			
Arctostaphylos uva-ursi	*Arctostaphylos uva-ursi*	(L.) Spreng.	yes	yes	yes			*ARCTOSTAPHYLOS UVA-URSI*
Arenaria leptoclados	*Arenaria leptoclados*	(Rchb.) Guss.	yes	yes	yes			
Arenaria serpyllifolia	*Arenaria serpyllifolia*	L.	yes	yes	yes			
Arenaria serpyllifolia agg.	*Arenaria serpyllifolia*	L.	yes	yes	yes	aggregate on species level		
Arenaria serpyllifolia subsp. glutinosa	*Arenaria serpyllifolia*	L.	yes	yes	yes	aggregate on species level		
Arenaria serpyllifolia subsp. serpyllifolia	*Arenaria serpyllifolia*	L.	yes	yes	yes	aggregate on species level		
Aristolochia clematitis	*Aristolochia clematitis*	L.	yes	yes	yes			
Armeria maritima	*Armeria maritima*	(Mill.) Willd.	yes	yes	yes			

Armeria maritima subsp. elongata	*Armeria elongata*	(Hoffm.) W. D. J. Koch	yes	yes	yes	Buttler & Hand 2023 ; Subspecies ecologically and morphologically distinct from other ssp. (Meyer 2023)	Kumar et al 2022, add at node N5167	*Armeria maritima*
Armoracia rusticana	*Armoracia rusticana*	P. Gaertn.. B. Mey. & Scherb.	yes	yes	no			
Arnica montana	*Arnica montana*	L.	yes	yes	yes			
Arnoseris minima	*Arnoseris minima*	(L.) Schweigg. & Koerte	yes	yes	yes			
Arrhenatherum elatius	*Arrhenatherum elatius*	(L.) P. Beauv. ex J. Presl & C. Presl	yes	yes	yes			
Arrhenatherum elatius var. bulbosum	*Arrhenatherum elatius*	(L.) P. Beauv. ex J. Presl & C. Presl	yes	yes	yes	aggregate on species level		
Arrhenatherum elatius var. elatius	*Arrhenatherum elatius*	(L.) P. Beauv. ex J. Presl & C. Presl	yes	yes	yes	aggregate on species level		
Artemisia absinthium	*Artemisia absinthium*	L.	yes	yes	yes			
Artemisia campestris	*Artemisia campestris*	L.	yes	yes	yes			
Artemisia campestris subsp. campestris	*Artemisia campestris*	L.	yes	yes	yes	aggregate on species level		

Artemisia scoparia	*Artemisia scoparia*	Waldst. & Kit.	yes	yes	yes			
Artemisia verlotiorum	*Artemisia verlotiorum*	Lamotte	yes	yes	no			
Artemisia vulgaris	*Artemisia vulgaris*	L.	yes	yes	yes			
Artemisia vulgaris agg.	*Artemisia vulgaris*	L.	yes	yes	yes	aggregate on species level		
Arum maculatum	*Arum maculatum*	L.	yes	yes	yes			
Arum maculatum agg.	*Arum maculatum*	L.	yes	yes	yes	aggregate on species level		
Aruncus dioicus	*Aruncus dioicus*	(Walter) Fernald	yes	yes	no			
Asarum europaeum	*Asarum europaeum*	L.	yes	yes	yes			
Asarum europaeum subsp. europaeum	*Asarum europaeum*	L.	yes	yes	yes	aggregate on species level		
Asparagus officinalis	*Asparagus officinalis*	L.	yes	yes	yes			
Asparagus officinalis subsp. officinalis	*Asparagus officinalis*	L.	yes	yes	yes	aggregate on species level		
Asperugo procumbens	*Asperugo procumbens*	L.	yes	yes	yes			
Asperula cynanchica	*Asperula cynanchica*	L.	yes	yes	no			
Asperula tinctoria	*Asperula tinctoria*	L.	yes	yes	yes			
Asplenium adiantum-nigrum	*Asplenium adiantum-nigrum*	L.	yes	yes	no			
Asplenium ceterach	*Asplenium ceterach*	L.	yes	yes	yes		*Ceterach officinarum*	*Asplenium ceterach*
Asplenium ceterach subsp. ceterach	*Asplenium ceterach*	L.	yes	yes	yes	aggregate on species level	*Ceterach officinarum*	
Asplenium fontanum	*Asplenium fontanum*	(L.) Bernh.	yes	yes	no			

Asplenium ruta-muraria	*Asplenium ruta-muraria*	L.	yes	yes	yes			
Asplenium ruta-muraria subsp. ruta-muraria	*Asplenium ruta-muraria*	L.	yes	yes	yes	aggregate on species level		
Asplenium scolopendrium	*Asplenium scolopendrium*	L.	yes	yes	no		*Phyllitis scolopendrium*	
Asplenium septentrionale	*Asplenium septentrionale*	(L.) Hoffm.	yes	yes	no			
Asplenium septentrionale subsp. septentrionale	*Asplenium septentrionale*	(L.) Hoffm.	yes	yes	no	aggregate on species level		
Asplenium trichomanes	*Asplenium trichomanes*	L.	yes	yes	yes			
Asplenium trichomanes ssp. quadrivalens	*Asplenium trichomanes*	L.	yes	yes	yes	aggregate on species level		
Asplenium trichomanes subsp. hastatum	*Asplenium trichomanes*	L.	yes	yes	yes	aggregate on species level		
Asplenium trichomanes subsp. pachyrachis	*Asplenium trichomanes*	L.	yes	yes	yes	aggregate on species level		
Asplenium trichomanes subsp. quadrivalens	*Asplenium trichomanes*	L.	yes	yes	yes	aggregate on species level		
Asplenium trichomanes subsp. trichomanes	*Asplenium trichomanes*	L.	yes	yes	yes	aggregate on species level		
Asplenium viride	*Asplenium viride*	Huds.	yes	yes	no			
Aster amellus	*Aster amellus*	L.	yes	yes	yes			
Aster bellidiastrum	*Aster bellidiastrum*	(L.) Scop.	yes	yes	yes			*Erigeron andicola*
Aster laevis agg.	*Aster laevis*	NA	yes	yes	no	aggregate on species level		

Aster lanceolatus	*Aster lanceolatus*	Willd.	yes	yes	yes			*Symphyotrichum lanceolatum*
Aster lanceolatus agg.	*Aster lanceolatus*	NA	yes	yes	yes	aggregate on species level		
Aster linosyris	*Aster linosyris*	(L.) Bernh.	yes	yes	yes			*Galatella linosyris*
Aster novae-angliae	*Aster novae-angliae*	L.	yes	yes	yes			*Symphyotrichum novae-angliae*
Aster novi-belgii	*Aster novi-belgii*	L.	yes	yes	yes			*Symphyotrichum novi-belgii*
Aster novi-belgii agg.	*Aster novi-belgii*	NA	yes	yes	yes	aggregate on species level		
Aster parviflorus	*Aster parviflorus*	Nees	yes	yes	no			
Aster salignus	*Aster salignus*	Willd.	yes	yes	no		*Aster x salignus*	
Aster versicolor	*Aster versicolor*	Willd.	yes	yes	no		*Aster x versicolor*	
Astragalus cicer	*Astragalus cicer*	L.	yes	yes	yes			
Astragalus glycyphyllos	*Astragalus glycyphyllos*	L.	yes	yes	yes			
Astrantia major	*Astrantia major*	L.	yes	yes	yes			
Astrantia major subsp. major	*Astrantia major*	L.	yes	yes	yes	aggregate on species level		
Athamanta cretensis	*Athamanta cretensis*	L.	yes	yes	yes			*Athamanta cretensis*
Athyrium distentifolium	*Athyrium distentifolium*	Tausch ex Opiz	yes	yes	no			

Athyrium filix-femina	*Athyrium filix-femina*	(L.) Roth	yes	yes	yes			
Atriplex micrantha	*Atriplex micrantha*	Ledeb.	yes	yes	yes			
Atriplex oblongifolia	*Atriplex oblongifolia*	Waldst. & Kit.	yes	yes	yes			
Atriplex patula	*Atriplex patula*	L.	yes	yes	yes			
Atriplex prostrata	*Atriplex prostrata*	DC.	yes	yes	yes			
Atriplex prostrata agg.	*Atriplex prostrata*	DC.	yes	yes	yes	aggregate on species level		
Atriplex prostrata subsp. latifolia	*Atriplex prostrata*	DC.	yes	yes	yes	aggregate on species level		
Atriplex rosea	*Atriplex rosea*	L.	yes	yes	yes			
Atriplex sagittata	*Atriplex sagittata*	Borkh.	yes	yes	no			
Atropa bella-donna	*Atropa bella-donna*	L.	yes	yes	yes			*ATROPA BELLA-DONNA*
Aurinia saxatilis	*Aurinia saxatilis*	L.	yes	yes	no		*Alyssum saxatile*	
Avena barbata	*Avena barbata*	Pott ex Link	yes	yes	yes			
Avena fatua	*Avena fatua*	L.	yes	yes	yes			
Avena sterilis subsp. ludoviciana	*Avena sterilis*	(Durieu) Nyman	yes	yes	no	aggregate on species level		
Avena sterilis subsp. sterilis	*Avena sterilis*	L.	yes	yes	no	aggregate on species level		
Avena strigosa	*Avena strigosa*	Schreb.	yes	yes	yes			
Azolla filiculoides	*Azolla filiculoides*	Lam.	yes	yes	no			
Ballota nigra	*Ballota nigra*	NA	yes	yes	yes			
Ballota nigra subsp. meridionalis	*Ballota nigra*	L.	yes	yes	yes	aggregate on species level		

Ballota nigra subsp. nigra	*Ballota nigra*	L.	yes	yes	yes	aggregate on species level		
Ballota nigra subsp.meridionalis	*Ballota nigra*	L.	yes	yes	yes	aggregate on species level		
Barbarea intermedia	*Barbarea intermedia*	Boreau	yes	yes	no			
Barbarea stricta	*Barbarea stricta*	Andrz.	yes	yes	yes			
Barbarea vulgaris	*Barbarea vulgaris*	R. Br.	yes	yes	yes			
Barbarea vulgaris subsp. arcuata	*Barbarea vulgaris*	R. Br.	yes	yes	yes	aggregate on species level		
Barbarea vulgaris subsp. rivularis	*Barbarea vulgaris*	R. Br.	yes	yes	yes	aggregate on species level		
Barbarea vulgaris subsp. vulgaris	*Barbarea vulgaris*	R. Br.	yes	yes	yes	aggregate on species level		
Bartsia alpina	*Bartsia alpina*	L.	yes	yes	yes			
Bassia laniflora	*Bassia laniflora*	(S. G. Gmel.) A. J. Scott	yes	yes	yes			
Bassia scoparia	*Bassia scoparia*	(L.) A. J. Scott	yes	yes	yes			
Bassia scoparia subsp. densiflora	*Bassia scoparia*	(L.) A. J. Scott	yes	yes	yes	aggregate on species level		
Bellis perennis	*Bellis perennis*	L.	yes	yes	yes			
Berberis vulgaris	*Berberis vulgaris*	L.	yes	yes	yes			
Berteroa incana	*Berteroa incana*	(L.) DC.	yes	yes	yes			
Berula erecta	*Berula erecta*	(Huds.) Coville	yes	yes	yes			
Betonica officinalis	*Betonica officinalis*	L.	yes	yes	yes			*Stachys officinalis*
Betula humilis	*Betula humilis*	Schrank	yes	yes	yes			
Betula nana	*Betula nana*	L.	yes	yes	yes			
Betula pendula	*Betula pendula*	Roth	yes	yes	yes			
Betula pubescens	*Betula pubescens*	Ehrh.	yes	yes	no			

Betula pubescens subsp. carpatica	*Betula pubescens*	Ehrh.	yes	yes	no	aggregate on species level		
Betula pubescens subsp. pubescens	*Betula pubescens*	Ehrh.	yes	yes	no	aggregate on species level		
Bidens cernua	*Bidens cernua*	L.	yes	yes	yes			
Bidens connata	*Bidens connata*	H. L. Muehl. ex Willd.	yes	yes	yes			
Bidens frondosa	*Bidens frondosa*	L.	yes	yes	yes			
Bidens radiata	*Bidens radiata*	Thuill.	yes	yes	yes			
Bidens tripartita	*Bidens tripartita*	L.	yes	yes	yes			
Bifora radians	*Bifora radians*	M. Bieb.	yes	yes	yes			
Biscutella laevigata	*Biscutella laevigata*	L.	yes	yes	no			
Bistorta officinalis	*Bistorta officinalis*	(L.) Samp.	yes	yes	yes			
Bistorta vivipara	*Bistorta vivipara*	(L.) Ronse Decr.	yes	yes	yes			*Bistorta vivipara*
Blackstonia acuminata	*Blackstonia acuminata*	(W. D. J. Koch & Ziz) Domin	yes	yes	no			
Blackstonia perfoliata	*Blackstonia perfoliata*	(L.) Huds.	yes	yes	yes			*Blackstonia perfoliata*
Blackstonia perfoliata agg.	*Blackstonia perfoliata*	(L.) Huds.	yes	yes	yes	aggregate on species level		
Blackstonia perfoliata subsp. perfoliata	*Blackstonia perfoliata*	(L.) Huds.	yes	yes	yes	aggregate on species level		
Blechnum spicant	*Blechnum spicant*	(L.) Roth	yes	yes	no			
Blysmus compressus	*Blysmus compressus*	(L.) Panz. ex Link	yes	yes	yes			

Bolboschoenus laticarpus	*Bolboschoenus laticarpus*	Marhold. Hroudová. Duchácek &	yes	yes	no		Kumar et al 2022, sistertaxon to B. maritimus x B. yagara	
Bolboschoenus maritimus	*Bolboschoenus maritimus*	(L.) Palla	yes	yes	yes			
Bolboschoenus maritimus agg.	*Bolboschoenus maritimus*	(L.) Palla	yes	yes	yes	aggregate on species level		
Bolboschoenus yagara	*Bolboschoenus maritimus*	(Ohwi) A. E. Kozhevn.	yes	yes	yes	Buttler & Hand 2023		
Bothriochloa ischaemum	*Bothriochloa ischaemum*	(L.) Keng	yes	no	yes			*Bothriochloa ischaemum*
Botrychium lunaria	*Botrychium lunaria*	(L.) Sw.	yes	yes	yes			
Botrychium matricariifolium	*Botrychium matricariifolium*	(A. Braun ex Doell) W. D. J. Koch	yes	yes	no			
Brachypodium pinnatum	*Brachypodium pinnatum*	(L.) P. Beauv.	yes	yes	yes			
Brachypodium pinnatum agg.	*Brachypodium pinnatum*	(L.) P. Beauv.	yes	yes	yes	aggregate on species level		
Brachypodium rupestre	*Brachypodium rupestre*	(Host) Roem. & Schult.	yes	yes	yes			*Brachypodium pinnatum*
Brachypodium sylvaticum	*Brachypodium sylvaticum*	(Huds.) P. Beauv.	yes	yes	yes			
Brachypodium sylvaticum subsp. sylvaticum	*Brachypodium sylvaticum*	(Huds.) P. Beauv.	yes	yes	yes	aggregate on species level		
Brassica napus	*Brassica napus*	L.	yes	yes	yes			

Brassica napus subsp. napus	*Brassica napus*	L.	yes	yes	yes	aggregate on species level		
Brassica napus subsp. rapifera	*Brassica napus*	L.	yes	yes	yes	aggregate on species level		
Brassica nigra	*Brassica nigra*	(L.) W. D. J. Koch	yes	yes	yes			
Brassica oleracea	*Brassica oleracea*	L.	yes	yes	yes			
Briza media	*Briza media*	L.	yes	yes	yes			
Briza media subsp. media	*Briza media*	L.	yes	yes	yes	aggregate on species level		
Bromus arvensis	*Bromus arvensis*	L.	yes	yes	yes			
Bromus arvensis subsp. arvensis	*Bromus arvensis*	L.	yes	yes	yes	aggregate on species level		
Bromus arvensis subsp. parviflorus	*Bromus arvensis*	L.	yes	yes	yes	aggregate on species level		
Bromus benekenii	*Bromus benekenii*	(Lange) Holub	yes	yes	yes			
Bromus carinatus	*Bromus carinatus*	Hook. & Arnott	yes	yes	no			
Bromus catharticus	*Bromus catharticus*	M. Vahl	yes	yes	no			
Bromus commutatus	*Bromus commutatus*	Schrad.	yes	yes	yes			
Bromus commutatus subsp. commutatus	*Bromus commutatus*	Schrad.	yes	yes	yes	aggregate on species level		
Bromus commutatus subsp. decipiens	*Bromus commutatus*	Schrad.	yes	yes	yes	aggregate on species level		
Bromus diandrus	*Bromus diandrus*	Roth	yes	yes	no			
Bromus erectus	*Bromus erectus*	Huds.	yes	yes	yes			
Bromus erectus agg.	*Bromus erectus*	Huds.	yes	yes	yes	aggregate on species level		

Bromus erectus subsp. erectus	*Bromus erectus*	Huds.	yes	yes	yes	aggregate on species level		
Bromus grossus	*Bromus grossus*	Desf. ex DC.	yes	yes	no			
Bromus hordeaceus	*Bromus hordeaceus*	L.	yes	yes	yes			
Bromus hordeaceus agg.	*Bromus hordeaceus*	L.	yes	yes	yes	aggregate on species level		
Bromus hordeaceus subsp. divaricatus	*Bromus hordeaceus*	L.	yes	yes	yes	aggregate on species level		
Bromus hordeaceus subsp. hordeaceus	*Bromus hordeaceus*	L.	yes	yes	yes	aggregate on species level		
Bromus hordeaceus subsp. longipedicellatus	*Bromus hordeaceus*	L.	yes	yes	yes	aggregate on species level		
Bromus hordeaceus subsp. longipedicellatus var. parviglumis	*Bromus hordeaceus*	L.	yes	yes	yes	aggregate on species level		
Bromus hordeaceus subsp. mediterraneus	*Bromus hordeaceus*	L.	yes	yes	yes	aggregate on species level		
Bromus hordeaceus subsp. pseudothominei	*Bromus hordeaceus*	L.	yes	yes	yes	aggregate on species level		
Bromus inermis	*Bromus inermis*	Leyss.	yes	yes	yes			
Bromus japonicus	*Bromus japonicus*	Thunb.	yes	yes	yes			
Bromus japonicus subsp. japonicus	*Bromus japonicus*	Thunb.	yes	yes	yes	aggregate on species level		
Bromus racemosus	*Bromus racemosus*	L.	yes	yes	yes			

Bromus racemosus agg.	*Bromus racemosus*	L.	yes	yes	yes	aggregate on species level		
Bromus ramosus	*Bromus ramosus*	Huds.	yes	yes	no			
Bromus ramosus agg.	*Bromus ramosus*	Huds.	yes	yes	no	aggregate on species level		
Bromus secalinus	*Bromus secalinus*	L.	yes	yes	yes			
Bromus secalinus agg.	*Bromus secalinus*	L.	yes	yes	yes	aggregate on species level		
Bromus squarrosus	*Bromus squarrosus*	L.	yes	yes	yes			
Bromus sterilis	*Bromus sterilis*	L.	yes	yes	yes			
Bromus tectorum	*Bromus tectorum*	L.	yes	yes	yes			
Brunnera macrophylla	*Brunnera macrophylla*	(Adams) I. M. Johnst.	yes	yes	no			
Bryonia alba	*Bryonia alba*	L.	yes	yes	yes			
Bryonia dioica	*Bryonia dioica*	Jacq.	yes	yes	yes			
Buddleja davidii	*Buddleja davidii*	Franch.	yes	yes	yes			
Bunias orientalis	*Bunias orientalis*	L.	yes	yes	yes			
Bunium bulbocastanum	*Bunium bulbocastanum*	L.	yes	yes	yes			
Buphthalmum salicifolium	*Buphthalmum salicifolium*	L.	yes	yes	yes			
Bupleurum falcatum	*Bupleurum falcatum*	L.	yes	yes	yes			
Bupleurum falcatum subsp. falcatum	*Bupleurum falcatum*	L.	yes	yes	yes	aggregate on species level		
Bupleurum longifolium	*Bupleurum longifolium*	L.	yes	yes	yes			
Bupleurum rotundifolium	*Bupleurum rotundifolium*	L.	yes	yes	yes			
Butomus umbellatus	*Butomus umbellatus*	L.	yes	yes	no			
Buxus sempervirens	*Buxus sempervirens*	L.	yes	yes	yes			
Calamagrostis arundinacea	*Calamagrostis arundinacea*	(L.) Roth	yes	yes	yes			

Calamagrostis canescens	*Calamagrostis canescens*	(Weber) Roth	yes	yes	yes			
Calamagrostis canescens agg.	*Calamagrostis canescens*	(Weber) Roth	yes	yes	yes	aggregate on species level		
Calamagrostis canescens subsp. canescens	*Calamagrostis canescens*	(Weber) Roth	yes	yes	yes	aggregate on species level		
Calamagrostis epigejos	*Calamagrostis epigejos*	(L.) Roth	yes	yes	yes			
Calamagrostis phragmitoides	*Calamagrostis phragmitoides*	Hartm.	yes	yes	yes			*Calamagrostis purpurea*
Calamagrostis pseudophragmites	*Calamagrostis pseudophragmites*	(Haller f.) Koel.	yes	yes	no			
Calamagrostis stricta	*Calamagrostis stricta*	(Timm) Koeler	yes	yes	no			
Calamagrostis varia	*Calamagrostis varia*	(Schrad.) Host	yes	yes	no			
Calamintha menthifolia	*Calamintha menthifolia*	Bromf.	yes	yes	no			
Calamintha nepeta	*Calamintha nepeta*	(L.) Savi	yes	yes	yes			*Clinopodium nepeta*
Calamintha nepeta agg.	*Calamintha nepeta*	(L.) Savi	yes	yes	yes	aggregate on species level		
Calendula arvensis	*Calendula arvensis*	L.	yes	yes	yes			
Calepina irregularis	*Calepina irregularis*	(Asso) Thell.	yes	yes	no			
Calla palustris	*Calla palustris*	L.	yes	yes	yes			
Callitriche cophocarpa	*Callitriche cophocarpa*	Sendtner	yes	yes	no			
Callitriche hamulata	*Callitriche hamulata*	Kuetz. ex W. D. J. Koch	yes	yes	no			
Callitriche obtusangula	*Callitriche obtusangula*	Le Gall	yes	yes	no			
Callitriche palustris	*Callitriche palustris*	L.	yes	yes	yes			

Callitriche palustris agg.	*Callitriche palustris*	L.	yes	yes	yes	aggregate on species level		
Callitriche platycarpa	*Callitriche platycarpa*	Kuetz.	yes	yes	no			
Callitriche stagnalis	*Callitriche stagnalis*	Scop.	yes	yes	no			
Calluna vulgaris	*Calluna vulgaris*	(L.) Hull	yes	yes	yes			
Caltha palustris	*Caltha palustris*	L.	yes	yes	yes			
Caltha palustris var. palustris	*Caltha palustris*	L.	yes	yes	yes	aggregate on species level		
Calystegia sepium	*Calystegia sepium*	(L.) R. Br.	yes	yes	yes			
Calystegia sepium agg.	*Calystegia sepium*	(L.) R. Br.	yes	yes	yes	aggregate on species level		
Calystegia sepium subsp. sepium	*Calystegia sepium*	(L.) R. Br.	yes	yes	yes	aggregate on species level		
Camelina microcarpa	*Camelina microcarpa*	Andrz.	yes	yes	yes			
Camelina microcarpa subsp. pilosa	*Camelina microcarpa*	Andrz.	yes	yes	yes	aggregate on species level		
Camelina microcarpa subsp. sylvestris	*Camelina microcarpa*	Andrz.	yes	yes	yes	aggregate on species level		
Camelina sativa	*Camelina sativa*	(L.) Crantz	yes	yes	no			
Camelina sativa agg.	*Camelina sativa*	(L.) Crantz	yes	yes	no	aggregate on species level		
Camelina sativa var. sativa	*Camelina sativa*	(L.) Crantz	yes	yes	no	aggregate on species level		
Campanula cervicaria	*Campanula cervicaria*	L.	yes	yes	yes			
Campanula cochleariifolia	*Campanula cochleariifolia*	Lam.	yes	yes	yes			
Campanula glomerata	*Campanula glomerata*	L.	yes	yes	yes			

Campanula glomerata subsp. farinosa	*Campanula glomerata*	L.	yes	yes	yes	aggregate on species level		
Campanula glomerata subsp. glomerata	*Campanula glomerata*	L.	yes	yes	yes	aggregate on species level		
Campanula latifolia	*Campanula latifolia*	L.	yes	yes	yes			
Campanula patula	*Campanula patula*	L.	yes	yes	yes			
Campanula patula subsp. patula	*Campanula patula*	L.	yes	yes	yes	aggregate on species level		
Campanula persicifolia	*Campanula persicifolia*	L.	yes	yes	yes			
Campanula persicifolia subsp. persicifolia	*Campanula persicifolia*	L.	yes	yes	yes	aggregate on species level		
Campanula rapunculoides	*Campanula rapunculoides*	L.	yes	yes	yes			
Campanula rapunculus	*Campanula rapunculus*	L.	yes	yes	yes			
Campanula rhomboidalis	*Campanula rhomboidalis*	L.	yes	yes	yes			
Campanula rotundifolia	*Campanula rotundifolia*	L.	yes	yes	yes			
Campanula rotundifolia agg.	*Campanula rotundifolia*	L.	yes	yes	yes	aggregate on species level		
Campanula scheuchzeri	*Campanula scheuchzeri*	Vill.	yes	yes	yes			
Campanula trachelium	*Campanula trachelium*	L.	yes	yes	yes			
Campanula trachelium subsp. trachelium	*Campanula trachelium*	L.	yes	yes	yes	aggregate on species level		
Cannabis sativa subsp. sativa	*Cannabis sativa*	L.	yes	yes	no	aggregate on species level		
Cannabis sativa subsp. spontanea	*Cannabis sativa*	L.	yes	yes	no	aggregate on species level		

Capsella bursa-pastoris	*Capsella bursa-pastoris*	(L.) Med.	yes	yes	yes			*CAPSELLA BURSA-PASTORIS*
Capsella rubella	*Capsella rubella*	Reut.	yes	yes	no			
Cardamine amara	*Cardamine amara*	L.	yes	yes	yes			
Cardamine amara subsp. amara	*Cardamine amara*	L.	yes	yes	yes	aggregate on species level		
Cardamine bulbifera	*Cardamine bulbifera*	(L.) Crantz	yes	yes	yes		*Dentaria bulbifera*	
Cardamine enneaphyllos	*Cardamine enneaphyllos*	(L.) Crantz	yes	yes	yes		*Dentaria enneaphyllos*	
Cardamine flexuosa	*Cardamine flexuosa*	With.	yes	yes	yes			
Cardamine heptaphylla	*Cardamine heptaphylla*	(Vill.) O. E. Schulz	yes	yes	no		*Dentaria heptaphyllos*	
Cardamine hirsuta	*Cardamine hirsuta*	L.	yes	yes	yes			
Cardamine impatiens	*Cardamine impatiens*	L.	yes	yes	yes			
Cardamine pentaphyllos	*Cardamine pentaphyllos*	L.	yes	yes	no		*Dentaria pentaphyllos*	
Cardamine pratensis	*Cardamine pratensis*	L.	yes	yes	yes			
Cardamine pratensis agg.	*Cardamine pratensis*	L.	yes	yes	yes	aggregate on species level		
Cardamine pratensis subsp. pratensis	*Cardamine pratensis*	L.	yes	yes	yes	aggregate on species level		
Cardaminopsis arenosa	*Cardaminopsis arenosa*	(L.) Hayek	yes	yes	yes			
Cardaminopsis arenosa subsp. arenosa	*Cardaminopsis arenosa*	(L.) Hayek	yes	yes	yes	aggregate on species level		

Cardaminopsis arenosa subsp. borbasii	*Cardaminopsis arenosa*	(L.) Hayek	yes	yes	yes	aggregate on species level		
Cardaminopsis halleri	*Cardaminopsis halleri*	(L.) Hayek	yes	yes	yes			*Arabidopsis halleri*
Cardaria draba	*Cardaria draba*	(L.) Desv.	yes	yes	yes			*Lepidium draba*
Carduus acanthoides	*Carduus acanthoides*	L.	yes	yes	yes			
Carduus acanthoides subsp. acanthoides	*Carduus acanthoides*	L.	yes	yes	yes	aggregate on species level		
Carduus crispus	*Carduus crispus*	L.	yes	yes	yes			*Carduus crispus*
Carduus crispus subsp. crispus	*Carduus crispus*	L.	yes	yes	yes	aggregate on species level		
Carduus defloratus	*Carduus defloratus*	L.	yes	yes	yes			
Carduus defloratus agg.	*Carduus defloratus*	L.	yes	yes	yes	aggregate on species level		
Carduus defloratus subsp. defloratus	*Carduus defloratus*	L.	yes	yes	yes	aggregate on species level		
Carduus nutans	*Carduus nutans*	L.	yes	yes	yes			
Carduus nutans agg.	*Carduus nutans*	L.	yes	yes	yes	aggregate on species level		
Carduus nutans subsp. nutans	*Carduus nutans*	L.	yes	yes	yes	aggregate on species level		
Carduus personata	*Carduus personata*	(L.) Jacq.	yes	yes	no			
Carex acuta	*Carex acuta*	L.	yes	yes	yes			
Carex acutiformis	*Carex acutiformis*	Ehrh.	yes	yes	yes			
Carex alba	*Carex alba*	Scop.	yes	yes	yes			
Carex appropinquata	*Carex appropinquata*	Schumach.	yes	yes	yes			

Carex bohemica	*Carex bohemica*	Schreb.	yes	yes	no			
Carex brachystachys	*Carex brachystachys*	Schrank	yes	yes	no			
Carex brizoides	*Carex brizoides*	L.	yes	yes	yes			
Carex buxbaumii	*Carex buxbaumii*	Wahlenb.	yes	yes	yes			
Carex buxbaumii agg.	*Carex buxbaumii*	Wahlenb.	yes	yes	yes	aggregate on species level		
Carex canescens	*Carex canescens*	L.	yes	yes	yes			
Carex canescens agg.	*Carex canescens*	L.	yes	yes	yes	aggregate on species level		
Carex caryophyllea	*Carex caryophyllea*	Latourr.	yes	yes	yes			
Carex cespitosa	*Carex cespitosa*	L.	yes	yes	yes			
Carex chordorrhiza	*Carex chordorrhiza*	L.f.	yes	yes	yes			
Carex davalliana	*Carex davalliana*	Sm.	yes	yes	yes			
Carex demissa	*Carex demissa*	Hornem.	yes	yes	yes			
Carex diandra	*Carex diandra*	Schrank	yes	yes	yes			
Carex digitata	*Carex digitata*	L.	yes	yes	yes			
Carex dioica	*Carex dioica*	L.	yes	yes	yes			
Carex distans	*Carex distans*	L.	yes	yes	yes			
Carex distans subsp. distans	*Carex distans*	L.	yes	yes	yes	aggregate on species level		
Carex disticha	*Carex disticha*	Huds.	yes	yes	yes			
Carex divulsa	*Carex muricata*	L.	yes	yes	yes	taxonomic inconsistencies between first and second mapping		
Carex echinata	*Carex echinata*	Murray	yes	yes	yes			
Carex elata	*Carex elata*	All.	yes	yes	yes			
Carex elata subsp. elata	*Carex elata*	All.	yes	yes	yes	aggregate on species level		
Carex elongata	*Carex elongata*	L.	yes	yes	yes			

Carex ericetorum	*Carex ericetorum*	Pollich	yes	yes	no			
Carex flacca	*Carex flacca*	Schreb.	yes	yes	yes			
Carex flacca subsp. serrulata	*Carex flacca*	Schreb.	yes	yes	yes	aggregate on species level		
Carex flava	*Carex flava*	L.	yes	yes	no			
Carex flava agg.	*Carex flava*	L.	yes	yes	no	aggregate on species level		
Carex flava var. alpina	*Carex flava*	L.	yes	yes	no	aggregate on species level		
Carex flava var. flava	*Carex flava*	L.	yes	yes	no	aggregate on species level		
Carex frigida	*Carex frigida*	All.	yes	yes	yes			
Carex guestphalica	*Carex muricata*	L.	yes	yes	yes	taxonomic inconsistencies between first and second mapping		*Carex divulsa*
Carex hartmanii	*Carex hartmanii*	Cajander	yes	yes	no			
Carex heleonastes	*Carex heleonastes*	L.f.	yes	yes	no			
Carex hirta	*Carex hirta*	L.	yes	yes	yes			
Carex hostiana	*Carex hostiana*	DC.	yes	yes	no			
Carex humilis	*Carex humilis*	Leyss.	yes	yes	yes			
Carex lasiocarpa	*Carex lasiocarpa*	Ehrh.	yes	yes	yes			
Carex lepidocarpa	*Carex lepidocarpa*	Tausch	yes	yes	yes			
Carex leporina	*Carex leporina*	L.	yes	yes	yes		*Carex ovalis*	
Carex limosa	*Carex limosa*	L.	yes	yes	yes			
Carex montana	*Carex montana*	L.	yes	yes	yes			
Carex muricata	*Carex muricata*	L.	yes	yes	no			
Carex muricata agg.	*Carex muricata*	L.	yes	yes	no	aggregate on species level		

Carex muricata s. str.	*Carex muricata*	L.	yes	yes	no	aggregate on species level		
Carex nigra	*Carex nigra*	(L.) Reichard	yes	yes	yes			
Carex nigra agg.	*Carex nigra*	(L.) Reichard	yes	yes	yes	aggregate on species level		
Carex ornithopoda	*Carex ornithopoda*	Willd.	yes	yes	no			
Carex ornithopoda agg.	*Carex ornithopoda*	Willd.	yes	yes	no	aggregate on species level		
Carex ornithopoda subsp. ornithopoda	*Carex ornithopoda*	Willd.	yes	yes	no	aggregate on species level		
Carex ornithopoda var. ornithopoda	*Carex ornithopoda*	Willd.	yes	yes	no	aggregate on species level		
Carex otrubae	*Carex otrubae*	Podp.	yes	yes	yes			
Carex ovalis	*Carex leporina*	L.	yes	yes	yes	taxonomic inconsistencies between first and second mapping		*Carex leporina*
Carex pairae	*Carex muricata*	L.	yes	yes	no	taxonomic inconsistencies between first and second mapping		
Carex pallescens	*Carex pallescens*	L.	yes	yes	yes			
Carex panicea	*Carex panicea*	L.	yes	yes	yes			

Carex paniculata	*Carex paniculata*	L.	yes	yes	yes			
Carex pauciflora	*Carex pauciflora*	Lightf.	yes	yes	yes			
Carex pendula	*Carex pendula*	Huds.	yes	yes	yes			
Carex pendula ssp. agastachys	*Carex pendula*	Huds.	yes	yes	yes	aggregate on species level		
Carex pendula subsp. agastachys	*Carex pendula*	Huds.	yes	yes	yes	aggregate on species level		
Carex pendula subsp. pendula	*Carex pendula*	Huds.	yes	yes	yes	aggregate on species level		
Carex pilosa	*Carex pilosa*	Scop.	yes	yes	yes			
Carex pilulifera	*Carex pilulifera*	L.	yes	yes	yes			
Carex praecox	*Carex praecox*	Schreb.	yes	yes	yes			
Carex praecox s. str.	*Carex praecox*	Schreb.	yes	yes	yes	aggregate on species level		
Carex praecox subsp. intermedia	*Carex praecox*	Schreb.	yes	yes	yes	aggregate on species level		
Carex praecox subsp. praecox	*Carex praecox*	Schreb.	yes	yes	yes	aggregate on species level		
Carex pseudocyperus	*Carex pseudocyperus*	L.	yes	yes	yes			
Carex pulicaris	*Carex pulicaris*	L.	yes	yes	yes			
Carex remota	*Carex remota*	L.	yes	yes	yes			
Carex riparia	*Carex riparia*	Curtis	yes	yes	yes			
Carex rostrata	*Carex rostrata*	Stokes	yes	yes	yes			
Carex sempervirens	*Carex sempervirens*	Vill.	yes	yes	yes			
Carex spicata	*Carex muricata*	L.	yes	yes	yes	taxonomic inconsistencies between first and second mapping		

Carex strigosa	*Carex strigosa*	Huds.	yes	yes	yes			
Carex supina	*Carex supina*	Wahlenb.	yes	yes	no			
Carex sylvatica	*Carex sylvatica*	Huds.	yes	yes	yes			
Carex tomentosa	*Carex tomentosa*	L.	yes	yes	no			
Carex umbrosa	*Carex umbrosa*	Host	yes	yes	yes			
Carex vesicaria	*Carex vesicaria*	L.	yes	yes	yes			
Carex viridula	*Carex viridula*	Michx.	yes	yes	no			
Carex viridula var. viridula	*Carex viridula*	Michx.	yes	yes	no	aggregate on species level		
Carex vulpina	*Carex vulpina*	L.	yes	yes	yes			
Carex vulpina agg.	*Carex vulpina*	L.	yes	yes	yes	aggregate on species level		
Carex vulpinoidea	*Carex vulpinoidea*	Michx.	yes	yes	yes			
Carlina acaulis	*Carlina acaulis*	L.	yes	yes	yes			
Carlina acaulis subsp. acaulis	*Carlina acaulis*	L.	yes	yes	yes	aggregate on species level		
Carlina acaulis subsp. caulescens	*Carlina acaulis*	L.	yes	yes	yes	aggregate on species level		
Carlina acaulis subsp. simplex	*Carlina acaulis*	L.	yes	yes	yes	aggregate on species level		
Carlina vulgaris	*Carlina vulgaris*	L.	yes	yes	yes			
Carlina vulgaris agg.	*Carlina vulgaris*	L.	yes	yes	yes	aggregate on species level		
Carpinus betulus	*Carpinus betulus*	L.	yes	yes	yes			
Carum carvi	*Carum carvi*	L.	yes	yes	yes			
Castanea sativa	*Castanea sativa*	Mill.	yes	yes	yes			
Catabrosa aquatica	*Catabrosa aquatica*	(L.) P. Beauv.	yes	yes	yes			
Caucalis platycarpos	*Caucalis platycarpos*	L.	yes	yes	no			
Caucalis platycarpos subsp. platycarpos	*Caucalis platycarpos*	L.	yes	yes	no	aggregate on species level		

Celtis occidentalis	*Celtis occidentalis*	L.	yes	yes	no		Kumar et al 2022, sistertaxon to C. jaceae	
Centaurea cyanus	*Centaurea cyanus*	L.	yes	yes	yes			
Centaurea diffusa	*Centaurea diffusa*	Lam.	yes	yes	yes			
Centaurea jacea	*Centaurea jacea*	L.	yes	yes	yes			
Centaurea jacea agg.	*Centaurea jacea*	L.	yes	yes	yes	aggregate on species level		
Centaurea jacea s. l.	*Centaurea jacea*	L.	yes	yes	yes	aggregate on species level		
Centaurea jacea s. str.	*Centaurea jacea*	L.	yes	yes	yes	aggregate on species level		
Centaurea jacea subsp. angustifolia	*Centaurea jacea*	L.	yes	yes	yes	aggregate on species level		
Centaurea jacea subsp. jacea	*Centaurea jacea*	L.	yes	yes	yes	aggregate on species level		
Centaurea montana	*Centaurea montana*	L.	yes	yes	no			
Centaurea montana subsp. montana	*Centaurea montana*	L.	yes	yes	no	aggregate on species level		
Centaurea nigra	*Centaurea nigra*	L.	yes	yes	yes			
Centaurea nigra subsp. nemoralis	*Centaurea nigra*	Jord.	yes	yes	yes	aggregate on species level		
Centaurea nigrescens	*Centaurea nigrescens*	Willd.	yes	yes	no			
Centaurea pannonica	*Centaurea pannonica*	Schrank	yes	yes	no			
Centaurea pseudophrygia	*Centaurea pseudophrygia*	C. A. Mey.	yes	yes	no			

Centaurea scabiosa	*Centaurea scabiosa*	L.	yes	yes	yes			
Centaurea scabiosa s.l.	*Centaurea scabiosa*	L.	yes	yes	yes	aggregate on species level		
Centaurea scabiosa subsp. alpestris	*Centaurea scabiosa*	L.	yes	yes	yes	aggregate on species level		
Centaurea scabiosa subsp. scabiosa	*Centaurea scabiosa*	L.	yes	yes	yes	aggregate on species level		
Centaurea stoebe	*Centaurea stoebe*	L.	yes	yes	yes			
Centaurea stoebe agg.	*Centaurea stoebe*	L.	yes	yes	yes	aggregate on species level		
Centaurea stoebe subsp. stoebe	*Centaurea stoebe*	L.	yes	yes	yes	aggregate on species level		
Centaurium erythraea	*Centaurium erythraea*	Rafn	yes	yes	yes			
Centaurium erythraea subsp. erythraea	*Centaurium erythraea*	Rafn	yes	yes	yes	aggregate on species level		
Centaurium pulchellum	*Centaurium pulchellum*	(Sw.) Druce	yes	yes	yes			
Centaurium pulchellum subsp. pulchellum	*Centaurium pulchellum*	(Sw.) Druce	yes	yes	yes	aggregate on species level		
Centranthus ruber	*Centranthus ruber*	(L.) DC.	yes	yes	yes			
Cephalanthera damasonium	*Cephalanthera damasonium*	(Mill.) Druce	yes	yes	yes			
Cephalanthera longifolia	*Cephalanthera longifolia*	(L.) Fritsch	yes	yes	yes			
Cephalanthera rubra	*Cephalanthera rubra*	(L.) Rich.	yes	yes	no			
Cerastium arvense	*Cerastium arvense*	L.	yes	yes	yes			
Cerastium arvense subsp. arvense	*Cerastium arvense*	L.	yes	yes	yes	aggregate on species level		

Cerastium arvense subsp. strictum	*Cerastium arvense*	L.	yes	yes	yes	aggregate on species level		
Cerastium brachypetalum	*Cerastium brachypetalum*	Desp. ex Pers.	yes	yes	no			
Cerastium brachypetalum agg.	*Cerastium brachypetalum*	Desp. ex Pers.	yes	yes	no	aggregate on species level		
Cerastium brachypetalum subsp. brachypetalum	*Cerastium brachypetalum*	Desp. ex Pers.	yes	yes	no	aggregate on species level		
Cerastium diffusum agg.	*Cerastium diffusum*	Pers.	yes	yes	no	aggregate on species level		
Cerastium fontanum	*Cerastium fontanum*	Baumg.	yes	yes	yes			
Cerastium fontanum agg.	*Cerastium fontanum*	Baumg.	yes	yes	yes	aggregate on species level		
Cerastium glomeratum	*Cerastium glomeratum*	Thuill.	yes	yes	yes			
Cerastium glutinosum	*Cerastium glutinosum*	Fr.	yes	yes	no			
Cerastium holosteoides	*Cerastium holosteoides*	Fr.	yes	yes	yes			
Cerastium holosteoides subsp. vulgare	*Cerastium holosteoides*	Fr.	yes	yes	yes	aggregate on species level		
Cerastium lucorum	*Cerastium lucorum*	(Schur) Moeschl	yes	yes	yes			*Cerastium fontanum*
Cerastium pumilum	*Cerastium pumilum*	Curtis	yes	yes	yes			
Cerastium pumilum agg.	*Cerastium pumilum*	Curtis	yes	yes	yes	aggregate on species level		
Cerastium pumilum subsp. pumilum	*Cerastium pumilum*	Curtis	yes	yes	yes	aggregate on species level		
Cerastium semidecandrum	*Cerastium semidecandrum*	L.	yes	yes	yes			

Cerastium tomentosum	*Cerastium tomentosum*	L.	yes	yes	yes			
Ceratophyllum demersum	*Ceratophyllum demersum*	L.	yes	yes	no			
Ceratophyllum demersum subsp. demersum	*Ceratophyllum demersum*	L.	yes	yes	no	aggregate on species level		
Ceratophyllum submersum	*Ceratophyllum submersum*	L.	yes	yes	no			
Chaenorhinum minus	*Chaenorhinum minus*	(L.) Lange	yes	yes	yes		*Microrrhinum minus*	
Chaenorhinum minus agg.	*Chaenorhinum minus*	(L.) Lange	yes	yes	yes	aggregate on species level	*Microrrhinum minus*	
Chaerophyllum aureum	*Chaerophyllum aureum*	L.	yes	yes	yes			
Chaerophyllum bulbosum	*Chaerophyllum bulbosum*	L.	yes	yes	yes			
Chaerophyllum hirsutum	*Chaerophyllum hirsutum*	L.	yes	yes	yes			
Chaerophyllum hirsutum subsp. hirsutum	*Chaerophyllum hirsutum*	L.	yes	yes	yes	aggregate on species level		
Chaerophyllum temulum	*Chaerophyllum temulum*	L.	yes	yes	yes			
Chamaespartium sagittale	*Chamaespartium sagittale*	L.	yes	yes	yes		*Genista sagittalis*	*Genista sagittalis*
Chamaesyce humifusa	*Chamaesyce humifusa*	Willd. ex Schlecht.	yes	yes	yes			*Euphorbia humifusa*
Chamaesyce maculata	*Chamaesyce maculata*	L.	yes	yes	no			
Chamaesyce nutans	*Chamaesyce nutans*	(Lag.) Small	yes	yes	no			
Chamaesyce prostrata	*Chamaesyce prostrata*	Aiton	yes	yes	no			
Chelidonium majus	*Chelidonium majus*	L.	yes	yes	yes			
Chenopodium album	*Chenopodium album*	L.	yes	yes	yes			

Chenopodium album agg.	*Chenopodium album*	L.	yes	yes	yes	aggregate on species level		
Chenopodium album cf. subsp. album	*Chenopodium album*	L.	yes	yes	yes	aggregate on species level		
Chenopodium album cf. subsp. pedunculare	*Chenopodium album*	L.	yes	yes	yes	aggregate on species level		
Chenopodium album ssp. album	*Chenopodium album*	L.	yes	yes	yes	aggregate on species level		
Chenopodium album subp. album	*Chenopodium album*	L.	yes	yes	yes	aggregate on species level		
Chenopodium album subsp. album	*Chenopodium album*	L.	yes	yes	yes	aggregate on species level		
Chenopodium album subsp. borbasii	*Chenopodium album*	L.	yes	yes	yes	aggregate on species level		
Chenopodium album subsp. pedunculare	*Chenopodium album*	L.	yes	yes	yes	aggregate on species level		
Chenopodium album var. Pedunculare	*Chenopodium album*	L.	yes	yes	yes	aggregate on species level		
Chenopodium bonus-henricus	*Chenopodium bonus-henricus*	L.	yes	yes	yes			*Blitum bonus-henricus*
Chenopodium botrys	*Chenopodium botrys*	L.	yes	yes	no			
Chenopodium ficifolium	*Chenopodium ficifolium*	Sm.	yes	yes	yes			
Chenopodium ficifolium subsp. ficifolium	*Chenopodium ficifolium*	Sm.	yes	yes	yes	aggregate on species level		
Chenopodium foliosum	*Chenopodium foliosum*	Asch.	yes	yes	yes			

Chenopodium glaucum	*Chenopodium glaucum*	L.	yes	yes	yes			*Oxybasis glauca*
Chenopodium hybridum	*Chenopodium hybridum*	L.	yes	yes	yes			*Chenopodiastrum hybridum*
Chenopodium murale	*Chenopodium murale*	L.	yes	yes	no			
Chenopodium opulifolium	*Chenopodium opulifolium*	Schrad. ex W. D. J. Koch & Ziz	yes	yes	no			
Chenopodium polyspermum	*Chenopodium polyspermum*	L.	yes	yes	yes			*Lipandra polysperma*
Chenopodium pumilio	*Chenopodium pumilio*	R. Br.	yes	yes	no			
Chenopodium rubrum	*Chenopodium rubrum*	L.	yes	yes	yes			*Oxybasis rubra*
Chenopodium strictum	*Chenopodium strictum*	Roth	yes	yes	no			
Chenopodium strictum subsp. striatiforme	*Chenopodium strictum*	Roth	yes	yes	no	aggregate on species level		
Chenopodium strictum subsp. strictum	*Chenopodium strictum*	Roth	yes	yes	no	aggregate on species level		
Chenopodium suecicum	*Chenopodium suecicum*	Murr	yes	yes	no			
Chenopodium urbicum	*Chenopodium urbicum*	L.	yes	yes	yes			*Oxybasis urbica*
Chenopodium urbicum var. intermedium	*Chenopodium urbicum*	L.	yes	yes	yes	aggregate on species level		
Chenopodium vulvaria	*Chenopodium vulvaria*	L.	yes	yes	yes			
Chimaphila umbellata	*Chimaphila umbellata*	(L.) Barton	yes	yes	no			
Chionodoxa forbesii	*Chionodoxa forbesii*	(Stapf) Speta	yes	yes	no			
Chionodoxa luciliae	*Chionodoxa luciliae*	Boiss.	yes	yes	no			

Chionodoxa sardensis	*Chionodoxa sardensis*	Whittall ex Barr & Sugden	yes	yes	no			
Chondrilla juncea	*Chondrilla juncea*	L.	yes	yes	yes			
Chrysanthemum segetum	*Chrysanthemum segetum*	L.	yes	yes	no		*Glebionis segetum*	
Chrysosplenium alternifolium	*Chrysosplenium alternifolium*	L.	yes	yes	yes			
Chrysosplenium oppositifolium	*Chrysosplenium oppositifolium*	L.	yes	yes	yes			
Cicerbita alpina	*Cicerbita alpina*	(L.) Wallr.	yes	yes	yes			*Lactuca alpina*
Cichorium intybus	*Cichorium intybus*	L.	yes	yes	yes			
Cichorium intybus subsp. intybus	*Cichorium intybus*	L.	yes	yes	yes	aggregate on species level		
Cichorium intybus subsp. sativum	*Cichorium intybus*	L.	yes	yes	yes	aggregate on species level		
Cicuta virosa	*Cicuta virosa*	L.	yes	yes	yes			
Circaea alpina	*Circaea alpina*	L.	yes	yes	yes			
Circaea intermedia	*Circaea intermedia*	Ehrh.	yes	yes	no		*Circaea x intermedia*	
Circaea lutetiana	*Circaea lutetiana*	L.	yes	yes	yes			
Cirsium acaulon	*Cirsium acaulon*	Scop.	yes	yes	no		*Cirsium acaule*	
Cirsium arvense	*Cirsium arvense*	(L.) Scop.	yes	yes	yes			
Cirsium eriophorum	*Cirsium eriophorum*	(L.) Scop.	yes	yes	yes			
Cirsium eriophorum subsp. eriophorum	*Cirsium eriophorum*	(L.) Scop.	yes	yes	yes	aggregate on species level		
Cirsium oleraceum	*Cirsium oleraceum*	(L.) Scop.	yes	yes	yes			
Cirsium palustre	*Cirsium palustre*	(L.) Scop.	yes	yes	yes			
Cirsium rivulare	*Cirsium rivulare*	(Jacq.) All.	yes	yes	no			

Cirsium tuberosum	*Cirsium tuberosum*	(L.) All.	yes	yes	yes			
Cirsium vulgare	*Cirsium vulgare*	(Savi) Ten.	yes	yes	yes			
Cladium mariscus	*Cladium mariscus*	(L.) Pohl	yes	yes	yes			
Claytonia perfoliata	*Claytonia perfoliata*	Donn ex Willd.	yes	yes	yes			
Clematis vitalba	*Clematis vitalba*	L.	yes	yes	yes			
Clinopodium vulgare	*Clinopodium vulgare*	L.	yes	yes	yes			
Clinopodium vulgare subsp. vulgare	*Clinopodium vulgare*	L.	yes	yes	yes	aggregate on species level		
Cnidium dubium	*Cnidium dubium*	(Schkuhr) Leute	yes	yes	yes			
Cochlearia danica	*Cochlearia danica*	L.	yes	yes	yes			
Cochlearia pyrenaica	*Cochlearia pyrenaica*	DC.	yes	yes	no			
Coeloglossum viride	*Coeloglossum viride*	(L.) Hartm.	yes	yes	yes			*Dactylorhiza viridis*
Coincya monensis	*Coincya monensis*	(L.) Greuter & Burdet	yes	yes	yes			
Coincya monensis subsp. cheiranthos	*Coincya monensis*	(Vill.) Aedo & al.	yes	yes	yes	aggregate on species level		
Colchicum autumnale	*Colchicum autumnale*	L.	yes	yes	yes			
Colutea arborescens	*Colutea arborescens*	L.	yes	yes	yes			
Conium maculatum	*Conium maculatum*	L.	yes	yes	yes			
Conringia orientalis	*Conringia orientalis*	(L.) Dumort.	yes	yes	yes			
Consolida ajacis	*Consolida ajacis*	(L.) Schur	yes	yes	no			
Consolida regalis	*Consolida regalis*	Gray	yes	yes	yes			*Delphinium consolida*
Consolida regalis subsp. regalis	*Consolida regalis*	Gray	yes	yes	yes	aggregate on species level		
Convallaria majalis	*Convallaria majalis*	L.	yes	yes	yes			

Convolvulus arvensis	*Convolvulus arvensis*	L.	yes	yes	yes			
Conyza bonariensis agg.	*Conyza bonariensis*	(L.) Cronquist	yes	yes	no	aggregate on species level		
Conyza canadensis	*Conyza canadensis*	(L.) Cronquist	yes	yes	yes			
Corallorrhiza trifida	*Corallorrhiza trifida*	Chatel.	yes	no	no			
Corispermum leptopterum	*Corispermum leptopterum*	(Asch.) Iljin	yes	yes	no			
Cornus alba agg. p. p.	*Cornus alba*	L.	yes	yes	no	aggregate on species level		
Cornus mas	*Cornus mas*	L.	yes	yes	yes			
Cornus sanguinea	*Cornus sanguinea*	L.	yes	yes	yes			
Cornus sanguinea subsp. australis	*Cornus sanguinea*	L.	yes	yes	yes	aggregate on species level		
Cornus sanguinea subsp. hungarica	*Cornus sanguinea*	L.	yes	yes	yes	aggregate on species level		
Cornus sanguinea subsp. sanguinea	*Cornus sanguinea*	L.	yes	yes	yes	aggregate on species level		
Cornus sericea	*Cornus sericea*	L.	yes	yes	yes			
Coronilla coronata	*Coronilla coronata*	L.	yes	yes	yes			
Coronilla vaginalis	*Coronilla vaginalis*	Lam.	yes	yes	yes			
Coronopus didymus	*Coronopus didymus*	(L.) Sm.	yes	yes	yes			*Lepidium didymum*
Coronopus squamatus	*Coronopus squamatus*	(Forssk.) Asch.	yes	yes	yes			*Lepidium coronopus*
Corrigiola litoralis	*Corrigiola litoralis*	L.	yes	yes	yes			

Corydalis cava	*Corydalis cava*	(L.) Schweigg. & Koerte	yes	yes	yes			*Corydalis cava*
Corydalis intermedia	*Corydalis intermedia*	(L.) Mérat	yes	yes	no			
Corydalis solida	*Corydalis solida*	(L.) Clairv.	yes	yes	yes			
Corylus avellana	*Corylus avellana*	L.	yes	yes	yes			
Corynephorus canescens	*Corynephorus canescens*	(L.) P. Beauv.	yes	yes	yes			
Cotoneaster dielsianus	*Cotoneaster dielsianus*	E. Pritz.	yes	yes	no			
Cotoneaster divaricatus	*Cotoneaster divaricatus*	Rehder & E. Wilson	yes	yes	no			
Cotoneaster integerrimus	*Cotoneaster integerrimus*	Medik.	yes	yes	yes			
Cotoneaster tomentosus	*Cotoneaster tomentosus*	Lindl.	yes	yes	no			
Crataegus laevigata	*Crataegus laevigata*	(Poir.) DC.	yes	yes	yes			
Crataegus macrocarpa	*Crataegus macrocarpa*	Hegetschw.	yes	yes	yes		*Crataegus x macrocarpa*	*Crataegus macrocarpa_x*
Crataegus macrocarpa var. hadensis	*Crataegus macrocarpa*	Hegetschw.	yes	yes	yes	aggregate on species level	*Crataegus x macrocarpa*	
Crataegus macrocarpa var. macrocarpa	*Crataegus macrocarpa*	Hegetschw.	yes	yes	yes	aggregate on species level	*Crataegus x macrocarpa*	
Crataegus monogyna	*Crataegus monogyna*	Jacq.	yes	yes	yes			
Crataegus rhipidophylla	*Crataegus rhipidophylla*	Gand.	yes	yes	no			
Crataegus rhipidophylla var. lindmanii	*Crataegus rhipidophylla*	Gand.	yes	yes	no	aggregate on species level		
Crataegus rhipidophylla var. rhipidophylla	*Crataegus rhipidophylla*	Gand.	yes	yes	no	aggregate on species level		
Crepis alpestris	*Crepis alpestris*	(Jacq.) Tausch	yes	yes	no			
Crepis biennis	*Crepis biennis*	L.	yes	yes	yes			

Crepis capillaris	*Crepis capillaris*	(L.) Wallr.	yes	yes	yes			
Crepis foetida	*Crepis foetida*	L.	yes	yes	yes			
Crepis mollis	*Crepis mollis*	(Jacq.) Asch.	yes	yes	no			
Crepis mollis subsp. mollis	*Crepis mollis*	(Jacq.) Asch.	yes	yes	no	aggregate on species level		
Crepis paludosa	*Crepis paludosa*	(L.) Moench	yes	yes	yes			
Crepis praemorsa	*Crepis praemorsa*	(L.) Walther	yes	yes	no			
Crepis pulchra	*Crepis pulchra*	L.	yes	yes	no			
Crepis pulchra subsp. pulchra	*Crepis pulchra*	L.	yes	yes	no	aggregate on species level		
Crepis setosa	*Crepis setosa*	Haller f.	yes	yes	no			
Crepis tectorum	*Crepis tectorum*	L.	yes	yes	yes			
Crepis vesicaria	*Crepis vesicaria*	L.	yes	yes	yes			
Crepis vesicaria subsp. taraxacifolia	*Crepis vesicaria*	L.	yes	yes	yes	aggregate on species level		
Crocus neglectus	*Crocus vernus*	(L.) Hill	yes	yes	no	Thiv & Wörz (2015)		
Crocus vernus	*Crocus vernus*	(L.) Hill	yes	yes	no			
Crocus vernus subsp. albiflorus	*Crocus vernus*	(L.) Hill	yes	yes	no	aggregate on species level		
Crocus vernus subsp. vernus	*Crocus vernus*	(L.) Hill	yes	yes	no	aggregate on species level		
Crocus vernus var. tommasinianus	*Crocus vernus*	(L.) Hill	yes	yes	no	aggregate on species level		
Cruciata glabra	*Cruciata glabra*	Scop.	yes	yes	yes			
Cruciata laevipes	*Cruciata laevipes*	Opiz	yes	yes	yes			
Cryptogramma crispa	*Cryptogramma crispa*	(L.) R. Br. ex Hook.	yes	yes	yes			
Cucubalus baccifer	*Cucubalus baccifer*	(L.) Roth	yes	yes	no			
Cuscuta campestris	*Cuscuta campestris*	Yunck.	yes	yes	no			

Cuscuta epithymum	*Cuscuta epithymum*	(L.) L.	yes	yes	no			
Cuscuta epithymum subsp. epithymum	*Cuscuta epithymum*	(L.) L.	yes	yes	no	aggregate on species level		
Cuscuta epithymum subsp. trifolii	*Cuscuta epithymum*	(L.) L.	yes	yes	no	aggregate on species level		
Cuscuta europaea	*Cuscuta europaea*	L.	yes	yes	no			
Cuscuta europaea subsp. europaea	*Cuscuta europaea*	L.	yes	yes	no	aggregate on species level		
Cuscuta lupuliformis	*Cuscuta lupuliformis*	Krock.	yes	yes	no			
Cymbalaria muralis	*Cymbalaria muralis*	P. Gaertn.. B. Mey. & Scherb.	yes	yes	yes			
Cynodon dactylon	*Cynodon dactylon*	(L.) Pers.	yes	yes	yes			
Cynoglossum germanicum	*Cynoglossum germanicum*	Jacq.	yes	yes	no			
Cynoglossum officinale	*Cynoglossum officinale*	L.	yes	yes	yes			
Cynosurus cristatus	*Cynosurus cristatus*	L.	yes	yes	yes			
Cyperus esculentus	*Cyperus esculentus*	L.	yes	yes	no			
Cyperus flavescens	*Cyperus flavescens*	L.	yes	yes	yes			
Cyperus fuscus	*Cyperus fuscus*	L.	yes	yes	yes			
Cyperus longus	*Cyperus longus*	L.	yes	yes	yes			
Cyperus longus subsp. longus	*Cyperus longus*	L.	yes	yes	yes	aggregate on species level		
Cypripedium calceolus	*Cypripedium calceolus*	L.	yes	yes	no			
Cystopteris fragilis	*Cystopteris fragilis*	(L.) Bernh.	yes	yes	no			
Cystopteris fragilis agg.	*Cystopteris fragilis*	(L.) Bernh.	yes	yes	no	aggregate on species level		

Cystopteris montana	*Cystopteris montana*	(Lam.) Desv.	yes	yes	no			
Cytisus nigricans	*Cytisus nigricans*	L.	yes	yes	yes			*Lembotropis nigricans*
Cytisus scoparius	*Cytisus scoparius*	(L.) W. D. J. Koch	yes	yes	yes			
Cytisus scoparius subsp. maritimus	*Cytisus scoparius*	(L.) W. D. J. Koch	yes	yes	yes	aggregate on species level		
Cytisus scoparius subsp. scoparius	*Cytisus scoparius*	(L.) W. D. J. Koch	yes	yes	yes	aggregate on species level		
Dactylis glomerata	*Dactylis glomerata*	L.	yes	yes	yes			
Dactylis glomerata agg.	*Dactylis glomerata*	L.	yes	yes	yes	aggregate on species level		
Dactylis glomerata subsp. glomerata	*Dactylis glomerata*	L.	yes	yes	yes	aggregate on species level		
Dactylis polygama	*Dactylis polygama*	Horv.	yes	yes	yes			*Dactylis glomerata*
Dactylorhiza fuchsii	*Dactylorhiza fuchsii*	(Druce) Soó	yes	yes	yes			
Dactylorhiza fuchsii subsp. fuchsii	*Dactylorhiza fuchsii*	(Druce) Soó	yes	yes	yes	aggregate on species level		
Dactylorhiza incarnata	*Dactylorhiza incarnata*	(L.) Verm.	yes	yes	yes			
Dactylorhiza incarnata agg.	*Dactylorhiza incarnata*	(L.) Verm.	yes	yes	yes	aggregate on species level		
Dactylorhiza incarnata subsp. incarnata	*Dactylorhiza incarnata*	(L.) Verm.	yes	yes	yes	aggregate on species level		

Dactylorhiza incarnata subsp. ochroleuca	*Dactylorhiza incarnata*	(L.) Verm.	yes	yes	yes	aggregate on species level		
Dactylorhiza incarnata subsp. ochroleuca	*Dactylorhiza incarnata*	(L.) Verm.	yes	yes	yes	aggregate on species level		
Dactylorhiza maculata	*Dactylorhiza maculata*	(L.) Soó	yes	yes	yes			
Dactylorhiza maculata agg.	*Dactylorhiza maculata*	(L.) Soó	yes	yes	yes	aggregate on species level		
Dactylorhiza maculata subsp. maculata	*Dactylorhiza maculata*	(L.) Soó	yes	yes	yes	aggregate on species level		
Dactylorhiza majalis	*Dactylorhiza majalis*	(Rchb.) Hunt & Summerh.	yes	yes	no			
Dactylorhiza majalis agg.	*Dactylorhiza majalis*	(Rchb.) Hunt & Summerh.	yes	yes	no	aggregate on species level		
Dactylorhiza majalis subsp. majalis	*Dactylorhiza majalis*	(Rchb.) Hunt & Summerh.	yes	yes	no	aggregate on species level		
Dactylorhiza sambucina	*Dactylorhiza sambucina*	(L.) Soó	yes	yes	no			
Dactylorhiza traunsteineri	*Dactylorhiza traunsteineri*	(Saut. ex Rchb.) Soó	yes	yes	no			
Danthonia decumbens	*Danthonia decumbens*	(L.) DC.	yes	yes	yes			
Danthonia decumbens subsp. decumbens	*Danthonia decumbens*	(L.) DC.	yes	yes	yes	aggregate on species level		
Daphne cneorum	*Daphne cneorum*	L.	yes	yes	no			
Daphne laureola	*Daphne laureola*	L.	yes	yes	yes			
Daphne mezereum	*Daphne mezereum*	L.	yes	yes	yes			
Datura stramonium	*Datura stramonium*	L.	yes	yes	yes			

Datura stramonium var. tatula	*Datura stramonium*	L.	yes	yes	yes	aggregate on species level		
Daucus carota	*Daucus carota*	L.	yes	yes	yes			
Daucus carota subsp. carota	*Daucus carota*	L.	yes	yes	yes	aggregate on species level		
Daucus carota subsp. sativus	*Daucus carota*	L.	yes	yes	yes	aggregate on species level		
Deschampsia cespitosa	*Deschampsia cespitosa*	(L.) P. Beauv.	yes	yes	yes			
Deschampsia cespitosa agg.	*Deschampsia cespitosa*	(L.) P. Beauv.	yes	yes	yes	aggregate on species level		
Deschampsia cespitosa s. str.	*Deschampsia cespitosa*	(L.) P. Beauv.	yes	yes	yes	aggregate on species level		
Deschampsia cespitosa subsp. cespitosa	*Deschampsia cespitosa*	(L.) P. Beauv.	yes	yes	yes	aggregate on species level		
Deschampsia flexuosa	*Deschampsia flexuosa*	(L.) Trin.	yes	yes	yes			
Deschampsia littoralis	*Deschampsia littoralis*	(Gaudin) Reuter	yes	yes	yes			*Deschampsia cespitosa*
Deschampsia media	*Deschampsia media*	(Gouan) Roem. & Schult.	yes	yes	no			
Descurainia sophia	*Descurainia sophia*	(L.) Prantl	yes	yes	yes			
Dianthus armeria	*Dianthus armeria*	L.	yes	yes	yes			
Dianthus armeria subsp. armeria	*Dianthus armeria*	L.	yes	yes	yes	aggregate on species level		
Dianthus carthusianorum	*Dianthus carthusianorum*	L.	yes	yes	yes			

Dianthus carthusianorum agg.	*Dianthus carthusianorum*	L.	yes	yes	yes	aggregate on species level		
Dianthus carthusianorum subsp. carthusianorum	*Dianthus carthusianorum*	L.	yes	yes	yes	aggregate on species level		
Dianthus deltoides	*Dianthus deltoides*	L.	yes	yes	yes			
Dianthus gratianopolitanus	*Dianthus gratianopolitanus*	Vill.	yes	yes	yes			
Dianthus seguieri	*Dianthus seguieri*	Willd.	yes	yes	no			
Dianthus seguieri subsp. glaber	*Dianthus seguieri*	Willd.	yes	yes	no	aggregate on species level		
Dianthus superbus	*Dianthus superbus*	L.	yes	yes	yes			
Dianthus superbus subsp. silvestris	*Dianthus superbus*	L.	yes	yes	yes	aggregate on species level		
Dianthus superbus subsp. superbus	*Dianthus superbus*	L.	yes	yes	yes	aggregate on species level		
Dictamnus albus	*Dictamnus albus*	L.	yes	yes	yes			
Digitalis grandiflora	*Digitalis grandiflora*	Mill.	yes	yes	yes			
Digitalis lutea	*Digitalis lutea*	L.	yes	yes	yes			
Digitalis purpurea	*Digitalis purpurea*	L.	yes	yes	yes			
Digitaria ischaemum	*Digitaria ischaemum*	(Schreb. ex Schweigg.) Schreb. ex Muehl.	yes	yes	yes			
Digitaria sanguinalis	*Digitaria sanguinalis*	(L.) Scop.	yes	yes	yes			
Digitaria sanguinalis subsp. pectiniformis	*Digitaria sanguinalis*	(L.) Scop.	yes	yes	yes	aggregate on species level		

Digitaria sanguinalis subsp. sanguinalis	*Digitaria sanguinalis*	(L.) Scop.	yes	yes	yes	aggregate on species level		
Diphasiastrum alpinum	*Diphasiastrum alpinum*	(L.) Rothm.	yes	yes	no			
Diphasiastrum complanatum	*Diphasiastrum complanatum*	(L.) Rothm.	yes	yes	no			
Diphasiastrum complanatum agg.	*Diphasiastrum complanatum*	(L.) Rothm.	yes	yes	no	aggregate on species level		
Diphasiastrum issleri	*Diphasiastrum issleri*	(Rouy) Holub	yes	yes	no			
Diphasiastrum oellgaardii	*Diphasiastrum oellgaardii*	A. M. Stoor et al.	yes	yes	no			
Diphasiastrum tristachyum	*Diphasiastrum tristachyum*	(Pursh) Rothm.	yes	yes	no			
Diphasiastrum zeilleri	*Diphasiastrum zeilleri*	(Rouy) Holub	yes	yes	no			
Diplotaxis muralis	*Diplotaxis muralis*	(L.) DC.	yes	yes	yes			
Diplotaxis tenuifolia	*Diplotaxis tenuifolia*	(L.) DC.	yes	yes	yes			
Dipsacus fullonum	*Dipsacus fullonum*	L.	yes	yes	yes			*Dipsacus sativus*
Dipsacus laciniatus	*Dipsacus laciniatus*	L.	yes	yes	no			
Dipsacus pilosus	*Dipsacus pilosus*	L.	yes	yes	yes		*Virga pilosa*	
Dipsacus strigosus	*Dipsacus strigosus*	Willd. ex Roem. & Schult.	yes	yes	yes		*Virga strigosa*	
Dittrichia graveolens	*Dittrichia graveolens*	(L.) Greuter	yes	yes	no			
Doronicum pardalianches	*Doronicum pardalianches*	L.	yes	yes	yes			
Draba aizoides	*Draba aizoides*	L.	yes	yes	yes			
Draba muralis	*Draba muralis*	L.	yes	yes	yes			
Drosera intermedia	*Drosera intermedia*	Hayne	yes	yes	yes			
Drosera longifolia	*Drosera longifolia*	Huds.	yes	yes	no			
Drosera rotundifolia	*Drosera rotundifolia*	L.	yes	yes	yes			
Dryopteris affinis	*Dryopteris affinis*	(Lowe) Fraser-Jenk.	yes	yes	no			

Dryopteris affinis s. l.	*Dryopteris affinis*	(Lowe) Fraser-Jenk.	yes	yes	no	aggregate on species level		
Dryopteris affinis subsp. borreri	*Dryopteris affinis*	(Lowe) Fraser-Jenk.	yes	yes	no	aggregate on species level		
Dryopteris affinis var. affinis	*Dryopteris affinis*	(Lowe) Fraser-Jenk.	yes	yes	no	aggregate on species level		
Dryopteris affinis var. borreri	*Dryopteris affinis*	(Lowe) Fraser-Jenk.	yes	yes	no	aggregate on species level		
Dryopteris affinis var. disjuncta	*Dryopteris affinis*	(Lowe) Fraser-Jenk.	yes	yes	no	aggregate on species level		
Dryopteris affinis var. insubrica	*Dryopteris affinis*	(Lowe) Fraser-Jenk.	yes	yes	no	aggregate on species level		
Dryopteris affinis var. robusta	*Dryopteris affinis*	(Lowe) Fraser-Jenk.	yes	yes	no	aggregate on species level		
Dryopteris affinis var. splendens	*Dryopteris affinis*	(Lowe) Fraser-Jenk.	yes	yes	no	aggregate on species level		
Dryopteris carthusiana	*Dryopteris carthusiana*	(Vill.) H. P. Fuchs	yes	yes	no			
Dryopteris carthusiana agg.	*Dryopteris carthusiana*	(Vill.) H. P. Fuchs	yes	yes	no	aggregate on species level		
Dryopteris cristata	*Dryopteris cristata*	(L.) A. Gray	yes	yes	no			
Dryopteris dilatata	*Dryopteris dilatata*	(Hoffm.) A. Gray	yes	yes	no			
Dryopteris expansa	*Dryopteris expansa*	(C. Presl) Fraser-Jenk. & Jermy	yes	yes	no			
Dryopteris filix-mas	*Dryopteris filix-mas*	(L.) Schott	yes	yes	no			

Dryopteris filix-mas agg.	*Dryopteris filix-mas*	(L.) Schott	yes	yes	no	aggregate on species level		
Dryopteris remota	*Dryopteris remota*	(A. Braun ex Doell) Druce	yes	yes	no			
Duchesnea indica	*Duchesnea indica*	(Andrews) Focke	yes	yes	no			
Echinochloa crus-galli	*Echinochloa crus-galli*	(L.) P. Beauv.	yes	yes	yes			
Echinochloa crus-galli subsp. crus-galli	*Echinochloa crus-galli*	(L.) P. Beauv.	yes	yes	yes	aggregate on species level		
Echinochloa crus-galli subsp. spiralis	*Echinochloa crus-galli*	(L.) P. Beauv.	yes	yes	yes	aggregate on species level		
Echinops banaticus	*Echinops banaticus*	Rochel ex Schrader	yes	yes	no			
Echinops exaltatus	*Echinops exaltatus*	Schrad.	yes	yes	no			
Echinops sphaerocephalus	*Echinops sphaerocephalus*	L.	yes	yes	no			
Echium vulgare	*Echium vulgare*	L.	yes	yes	yes			
Elatine alsinastrum	*Elatine alsinastrum*	L.	yes	yes	no			
Elatine hexandra	*Elatine hexandra*	(Lapierre) DC.	yes	yes	no			
Elatine hydropiper	*Elatine hydropiper*	L.	yes	yes	no			
Elatine triandra	*Elatine triandra*	Schkuhr	yes	yes	no			
Eleocharis acicularis	*Eleocharis acicularis*	(L.) Roem. & Schult.	yes	yes	no			
Eleocharis austriaca	*Eleocharis mamillata*	Lindb.f.	yes	yes	no	Buttler & Hand 2023		
Eleocharis mamillata	*Eleocharis mamillata*	Lindb.f.	yes	yes	no			
Eleocharis mamillata s. Lat.	*Eleocharis mamillata*	Lindb.f.	yes	yes	no	aggregate on species level		
Eleocharis ovata	*Eleocharis ovata*	(Roth) Roem. & Schult.	yes	yes	yes			

Eleocharis palustris	*Eleocharis palustris*	(L.) Roem. & Schult.	yes	yes	yes			
Eleocharis palustris agg.	*Eleocharis palustris*	(L.) Roem. & Schult.	yes	yes	yes	aggregate on species level		
Eleocharis palustris subsp. palustris	*Eleocharis palustris*	(L.) Roem. & Schult.	yes	yes	yes	aggregate on species level		
Eleocharis palustris subsp. vulgaris	*Eleocharis palustris*	(L.) Roem. & Schult.	yes	yes	yes	aggregate on species level		
Eleocharis quinqueflora	*Eleocharis quinqueflora*	(Hartmann) O. Schwarz	yes	yes	yes			
Eleocharis uniglumis	*Eleocharis uniglumis*	(Link) Schult.	yes	yes	yes			
Elodea canadensis	*Elodea canadensis*	Michx.	yes	yes	no			
Elodea nuttallii	*Elodea nuttallii*	(Planch.) H. St. John	yes	yes	no			
Elymus caninus	*Elymus caninus*	(L.) L.	yes	yes	yes		*Roegneria canina*	
Elymus obtusiflorus	*Elymus obtusiflorus*	(Host) Runemark	yes	yes	no		*Elytrigia obtusiflora*	
Elymus repens	*Elymus repens*	(L.) Gould	yes	yes	yes		*Elytrigia repens*	
Elymus repens s. str.	*Elymus repens*	(L.) Gould	yes	yes	yes	aggregate on species level	*Elytrigia repens*	
Elymus repens subsp. repens	*Elymus repens*	(L.) Gould	yes	yes	yes	aggregate on species level	*Elytrigia repens*	
Elymus repens subsp. repens var. aristatus	*Elymus repens*	(L.) Gould	yes	yes	yes	aggregate on species level	*Elytrigia repens*	

Elymus repens var. caesia	*Elymus repens*	(L.) Gould	yes	yes	yes	aggregate on species level	*Elytrigia repens*	
Empetrum hermaphroditum	*Empetrum hermaphroditum*	(Hagerup) Boecher	yes	yes	no			
Empetrum nigrum	*Empetrum nigrum*	L.	yes	yes	yes			
Empetrum nigrum agg.	*Empetrum nigrum*	L.	yes	yes	yes	aggregate on species level		
Epilobium alsinifolium	*Epilobium alsinifolium*	Vill.	yes	yes	no			
Epilobium angustifolium	*Epilobium angustifolium*	L.	yes	yes	yes			
Epilobium brachycarpum	*Epilobium brachycarpum*	C. Presl	yes	yes	no			
Epilobium ciliatum	*Epilobium ciliatum*	Raf.	yes	yes	yes			
Epilobium ciliatum subsp. adenocaulon	*Epilobium ciliatum*	Raf.	yes	yes	yes	aggregate on species level		
Epilobium ciliatum subsp. ciliatum	*Epilobium ciliatum*	Raf.	yes	yes	yes	aggregate on species level		
Epilobium ciliatum subsp. glandulosum	*Epilobium ciliatum*	Raf.	yes	yes	yes	aggregate on species level		
Epilobium collinum	*Epilobium collinum*	C. C. Gmel.	yes	yes	no			
Epilobium dodonaei	*Epilobium dodonaei*	Vill.	yes	yes	yes			
Epilobium duriaei	*Epilobium duriaei*	Godr.	yes	yes	no			
Epilobium fleischeri	*Epilobium fleischeri*	Hochst.	yes	yes	yes			
Epilobium hirsutum	*Epilobium hirsutum*	L.	yes	yes	yes			
Epilobium lanceolatum	*Epilobium lanceolatum*	Sebast. & Mauri	yes	yes	no			
Epilobium montanum	*Epilobium montanum*	L.	yes	yes	yes			
Epilobium nutans	*Epilobium nutans*	F. W. Schmidt	yes	yes	yes			

Epilobium obscurum	*Epilobium obscurum*	Schreb.	yes	yes	yes			
Epilobium palustre	*Epilobium palustre*	L.	yes	yes	yes			
Epilobium parviflorum	*Epilobium parviflorum*	Schreb.	yes	yes	yes			
Epilobium roseum	*Epilobium roseum*	Schreb.	yes	yes	yes			
Epilobium tetragonum	*Epilobium tetragonum*	L.	yes	yes	yes			
Epilobium tetragonum s. l.	*Epilobium tetragonum*	L.	yes	yes	yes	aggregate on species level		
Epilobium tetragonum s. str.	*Epilobium tetragonum*	L.	yes	yes	yes	aggregate on species level		
Epilobium tetragonum subsp. lamyi	*Epilobium tetragonum*	L.	yes	yes	yes	aggregate on species level		
Epilobium tetragonum subsp. tetragonum	*Epilobium tetragonum*	L.	yes	yes	yes	aggregate on species level		
Epipactis atrorubens	*Epipactis atrorubens*	(Hoffm.) Besser	yes	yes	yes			
Epipactis helleborine	*Epipactis helleborine*	(L.) Crantz	yes	yes	yes			
Epipactis helleborine agg.	*Epipactis helleborine*	(L.) Crantz	yes	yes	yes	aggregate on species level		
Epipactis helleborine subsp. helleborine	*Epipactis helleborine*	(L.) Crantz	yes	yes	yes	aggregate on species level		
Epipactis leptochila	*Epipactis leptochila*	(Godfery) Godfery	yes	yes	no			
Epipactis leptochila subsp. neglecta	*Epipactis leptochila*	(Godfery) Godfery	yes	yes	no	aggregate on species level		
Epipactis microphylla	*Epipactis microphylla*	(Ehrh.) Sw.	yes	yes	yes			
Epipactis muelleri	*Epipactis muelleri*	Godfery	yes	yes	no			

Epipactis palustris	*Epipactis palustris*	(L.) Crantz	yes	yes	yes			
Epipactis purpurata	*Epipactis purpurata*	Sm.	yes	yes	no			
Epipogium aphyllum	*Epipogium aphyllum*	Sw.	yes	yes	no			
Equisetum arvense	*Equisetum arvense*	L.	yes	yes	no			
Equisetum fluviatile	*Equisetum fluviatile*	L.	yes	yes	no			
Equisetum hyemale	*Equisetum hyemale*	L.	yes	yes	no			
Equisetum litorale	*Equisetum litorale*	Kuehlew. ex Rupr.	yes	no	no			
Equisetum meridionale	*Equisetum meridionale*	(Milde) Chiov.	yes	no	no			
Equisetum moorei	*Equisetum moorei*	Newman	yes	no	no			
Equisetum palustre	*Equisetum palustre*	L.	yes	yes	no			
Equisetum pratense	*Equisetum pratense*	Ehrh.	yes	yes	no			
Equisetum ramosissimum	*Equisetum ramosissimum*	Desf.	yes	yes	no			
Equisetum sylvaticum	*Equisetum sylvaticum*	L.	yes	yes	no			
Equisetum telmateia	*Equisetum telmateia*	Ehrh.	yes	yes	no			
Equisetum variegatum	*Equisetum variegatum*	Schleich. ex Weber & D. Mohr	yes	yes	no			
Eragrostis cilianensis	*Eragrostis cilianensis*	(All.) Vignolo ex Janch.	yes	yes	yes			
Eragrostis minor	*Eragrostis minor*	Host	yes	yes	yes			
Eragrostis multicaulis	*Eragrostis multicaulis*	Steud.	yes	yes	no			
Eragrostis pilosa	*Eragrostis pilosa*	(L.) P. Beauv.	yes	yes	yes			
Eranthis hyemalis	*Eranthis hyemalis*	(L.) Salisb.	yes	yes	no			
Erigeron acris	*Erigeron acris*	L.	yes	yes	no			
Erigeron acris agg.	*Erigeron acris*	L.	yes	yes	no	aggregate on species level		

Erigeron acris subsp. acris	*Erigeron acris*	L.	yes	yes	no	aggregate on species level		
Erigeron acris subsp. angulosus	*Erigeron acris*	L.	yes	yes	no	aggregate on species level		
Erigeron acris subsp. serotinus	*Erigeron acris*	L.	yes	yes	no	aggregate on species level		
Erigeron annuus	*Erigeron annuus*	(L.) Pers.	yes	yes	yes			
Erigeron annuus subsp. annuus	*Erigeron annuus*	(L.) Pers.	yes	yes	yes	aggregate on species level		
Erigeron annuus subsp. septentrionalis	*Erigeron annuus*	(L.) Pers.	yes	yes	yes	aggregate on species level		
Erigeron annuus subsp. strigosus	*Erigeron annuus*	(L.) Pers.	yes	yes	yes	aggregate on species level		
Erigeron bonariensis	*Conyza bonariensis*	(L.) Cronquist	yes	yes	no			
Erigeron canadensis	*Conyza canadensis*	(L.) Cronquist	yes	yes	yes			
Erigeron muralis	*Erigeron muralis*	Lapeyr.	yes	yes	no		Kumar et al 2022, sistertaxon to E. acris	
Erigeron sumatrensis	*Erigeron sumatrensis*	Retz.	yes	yes	yes			
Eriophorum angustifolium	*Eriophorum angustifolium*	Honck.	yes	yes	yes			
Eriophorum gracile	*Eriophorum gracile*	W. D. J. Koch ex Roth	yes	yes	no			
Eriophorum latifolium	*Eriophorum latifolium*	Hoppe	yes	yes	yes			
Eriophorum vaginatum	*Eriophorum vaginatum*	L.	yes	yes	yes			
Erodium cicutarium	*Erodium cicutarium*	(L.) L'Hér.	yes	yes	yes			

Erodium cicutarium agg.	*Erodium cicutarium*	(L.) L'Her.	yes	yes	yes	aggregate on species level		
Erodium cicutarium s. str.	*Erodium cicutarium*	(L.) L'Her.	yes	yes	yes	aggregate on species level		
Erophila verna	*Erophila verna*	L.	yes	yes	yes			*Draba verna*
Erophila verna subsp. praecox	*Erophila verna*	L.	yes	yes	yes	aggregate on species level		
Erophila verna subsp. spathulata	*Erophila verna*	L.	yes	yes	yes	aggregate on species level		
Erophila verna subsp. verna	*Erophila verna*	L.	yes	yes	yes	aggregate on species level		
Erucastrum gallicum	*Erucastrum gallicum*	(Willd.) O. E. Schulz	yes	yes	yes			
Erucastrum nasturtiifolium	*Erucastrum nasturtiifolium*	(Poir.) O. E. Schulz	yes	yes	yes			
Eryngium campestre	*Eryngium campestre*	L.	yes	yes	yes			
Eryngium planum	*Eryngium planum*	L.	yes	yes	no			
Erysimum cheiranthoides	*Erysimum cheiranthoides*	L.	yes	yes	yes			
Erysimum cheiri	*Erysimum cheiri*	(L.) Crantz	yes	yes	yes			*Erysimum cheiri_x*
Erysimum crepidifolium	*Erysimum crepidifolium*	Rchb.	yes	yes	no			
Erysimum hieraciifolium	*Erysimum hieraciifolium*	P. Gaertn.. B. Mey. & Scherb.	yes	yes	no		*Erysimum virgatum*	
Erysimum hieraciifolium agg.	*Erysimum hieraciifolium*	P. Gaertn.. B. Mey. & Scherb.	yes	yes	no	aggregate on species level	*Erysimum virgatum*	

Erysimum odoratum	*Erysimum odoratum*	Ehrh.	yes	yes	yes			
Euonymus europaeus	*Euonymus europaeus*	L.	yes	yes	no		*Eonymus europaea*	
Euonymus latifolius	*Euonymus latifolius*	(L.) Mill.	yes	yes	no		*Eonymus latifolia*	
Eupatorium cannabinum	*Eupatorium cannabinum*	L.	yes	yes	yes			
Euphorbia amygdaloides	*Euphorbia amygdaloides*	L.	yes	yes	yes			
Euphorbia cyparissias	*Euphorbia cyparissias*	L.	yes	yes	yes			
Euphorbia dulcis	*Euphorbia dulcis*	L.	yes	yes	yes			
Euphorbia dulcis subsp. dulcis	*Euphorbia dulcis*	L.	yes	yes	yes	aggregate on species level		
Euphorbia dulcis subsp. incompta	*Euphorbia dulcis*	L.	yes	yes	yes	aggregate on species level		
Euphorbia dulcis subsp. purpurata	*Euphorbia dulcis*	L.	yes	yes	yes	aggregate on species level		
Euphorbia esula	*Euphorbia esula*	L.	yes	yes	no			
Euphorbia esula agg.	*Euphorbia esula*	L.	yes	yes	no	aggregate on species level		
Euphorbia exigua	*Euphorbia exigua*	L.	yes	yes	yes			
Euphorbia helioscopia	*Euphorbia helioscopia*	L.	yes	yes	yes			
Euphorbia lathyrus	*Euphorbia lathyrus*	L.	yes	yes	yes			
Euphorbia palustris	*Euphorbia palustris*	L.	yes	yes	yes			
Euphorbia peplus	*Euphorbia peplus*	L.	yes	yes	yes			
Euphorbia peplus agg.	*Euphorbia peplus*	L.	yes	yes	yes	aggregate on species level		
Euphorbia platyphyllos	*Euphorbia platyphyllos*	L.	yes	yes	no			

Euphorbia platyphyllos subsp. platyphyllos	*Euphorbia platyphyllos*	L.	yes	yes	no	aggregate on species level		
Euphorbia pseudovirgata	*Euphorbia pseudovirgata*	Schur	yes	yes	no		*Euphorbia x pseudovirgata*	
Euphorbia seguieriana	*Euphorbia seguieriana*	Neck.	yes	yes	yes			
Euphorbia stricta	*Euphorbia stricta*	L.	yes	yes	no			
Euphorbia verrucosa	*Euphorbia verrucosa*	L.	yes	yes	yes			
Euphrasia micrantha	*Euphrasia micrantha*	Rchb.	yes	yes	no			
Euphrasia nemorosa	*Euphrasia nemorosa*	(Pers.) Wallr.	yes	yes	no			
Euphrasia nemorosa agg.	*Euphrasia nemorosa*	(Pers.) Wallr.	yes	yes	no	aggregate on species level		
Euphrasia nemorosa subsp. nemorosa	*Euphrasia nemorosa*	(Pers.) Wallr.	yes	yes	no	aggregate on species level		
Euphrasia officinalis	*Euphrasia officinalis*	Hayne	yes	yes	yes			
Euphrasia officinalis subsp. pratensis	*Euphrasia officinalis*	Hayne	yes	yes	yes	aggregate on species level		
Euphrasia officinalis subsp. rostkoviana	*Euphrasia officinalis*	Hayne	yes	yes	yes	aggregate on species level		
Euphrasia rostkoviana	*Euphrasia officinalis*	Hayne	yes	yes	yes	aggregate on species level		
Euphrasia rostkoviana subsp. montana	*Euphrasia officinalis*	Hayne	yes	yes	yes	aggregate on species level		
Euphrasia salisburgensis	*Euphrasia salisburgensis*	Funck ex Hoppe	yes	yes	no			
Euphrasia stricta	*Euphrasia stricta*	D. Wolff ex J. F. Lehmann	yes	yes	yes			

Euphrasia stricta agg.	*Euphrasia stricta*	D. Wolff ex J. F. Lehmann	yes	yes	yes	aggregate on species level		
Fagus sylvatica	*Fagus sylvatica*	L.	yes	yes	yes			
Falcaria vulgaris	*Falcaria vulgaris*	Bernh.	yes	yes	yes			
Fallopia convolvulus	*Fallopia convolvulus*	(L.) Å. Loeve	yes	yes	yes			
Fallopia dumetorum	*Fallopia dumetorum*	(L.) Holub	yes	yes	yes			
Fallopia japonica	*Fallopia japonica*	Houtt.	yes	yes	yes			*Reynoutria japonica*
Fallopia japonica var. compacta	*Fallopia japonica*	Houtt.	yes	yes	yes	aggregate on species level		
Fallopia japonica var. japonica	*Fallopia japonica*	Houtt.	yes	yes	yes	aggregate on species level		
Fallopia sachalinensis	*Fallopia sachalinensis*	(F. Schmidt) Nakai	yes	yes	yes			*Reynoutria sachalinensis*
Festuca altissima	*Festuca altissima*	All.	yes	yes	yes			
Festuca amethystina	*Festuca amethystina*	L.	yes	yes	no			
Festuca arundinacea	*Festuca arundinacea*	Schreb.	yes	yes	yes			
Festuca arundinacea subsp. arundinacea	*Festuca arundinacea*	Schreb.	yes	yes	yes	aggregate on species level		
Festuca arundinacea subsp. orientalis	*Festuca arundinacea*	Schreb.	yes	yes	yes	aggregate on species level		
Festuca arundinacea subsp. uechtriziana	*Festuca arundinacea*	Schreb.	yes	yes	yes	aggregate on species level		
Festuca brevipila	*Festuca ovina*	L.	yes	yes	yes	part of F. ovina-complex		

Festuca filiformis	*Festuca ovina*	L.	yes	yes	yes	Rothmaler 2021		
Festuca gigantea	*Festuca gigantea*	(L.) Vill.	yes	yes	no			
Festuca guestfalica	*Festuca ovina*	L.	yes	yes	yes	part of F. ovina-complex		
Festuca guestfalica subsp. guestfalica	*Festuca ovina*	L.	yes	yes	yes	Rothmaler 2021		
Festuca heteromalla	*Festuca rubra*	L.	yes	yes	yes	part of F. rubra-complex		
Festuca heterophylla	*Festuca heterophylla*	Lam.	yes	yes	no			
Festuca nigrescens	*Festuca rubra*	L.	yes	yes	yes	Rothmaler 2021		
Festuca ovina	*Festuca ovina*	L.	yes	yes	yes			
Festuca ovina agg.	*Festuca ovina*	L.	yes	yes	yes	aggregate on species level		
Festuca ovina subsp. guestfalica	*Festuca ovina*	L.	yes	yes	yes	aggregate on species level		
Festuca pallens	*Festuca ovina*	L.	yes	yes	yes	Rothmaler 2021		
Festuca pratensis	*Festuca pratensis*	Huds.	yes	yes	no			
Festuca pratensis s. str.	*Festuca pratensis*	Huds.	yes	yes	no	aggregate on species level		
Festuca pratensis subsp. pratensis	*Festuca pratensis*	Huds.	yes	yes	no	aggregate on species level		
Festuca rubra	*Festuca rubra*	L.	yes	yes	yes			

Festuca rubra agg.	*Festuca rubra*	L.	yes	yes	yes	aggregate on species level		
Festuca rubra subsp. juncea	*Festuca rubra*	L.	yes	yes	yes	aggregate on species level		
Festuca rubra subsp. rubra	*Festuca rubra*	L.	yes	yes	yes	aggregate on species level		
Festuca rupicola	*Festuca ovina*	L.	yes	yes	yes	Rothmaler 2021		
Festuca valesiaca subsp. parviflora	*Festuca rubra*	L.	yes	yes	yes	Rothmaler 2021		
Filago arvensis	*Filago arvensis*	L.	yes	yes	yes			
Filago lutescens	*Filago lutescens*	Jord.	yes	yes	yes			
Filago minima	*Filago minima*	(Sm.) Pers.	yes	yes	yes			
Filago pyramidata	*Filago pyramidata*	L.	yes	yes	yes			
Filago vulgaris	*Filago vulgaris*	Lam.	yes	yes	yes			
Filago vulgaris agg.	*Filago vulgaris*	Lam.	yes	yes	yes	aggregate on species level		
Filipendula ulmaria	*Filipendula ulmaria*	(L.) Maxim.	yes	yes	yes			
Filipendula ulmaria subsp. denudata	*Filipendula ulmaria*	(L.) Maxim.	yes	yes	yes	aggregate on species level		
Filipendula ulmaria subsp. ulmaria	*Filipendula ulmaria*	(L.) Maxim.	yes	yes	yes	aggregate on species level		
Filipendula ulmaria var. denudata	*Filipendula ulmaria*	(L.) Maxim.	yes	yes	yes	aggregate on species level		
Filipendula ulmaria var. ulmaria	*Filipendula ulmaria*	(L.) Maxim.	yes	yes	yes	aggregate on species level		
Filipendula vulgaris	*Filipendula vulgaris*	Moench	yes	yes	yes			

Fragaria moschata	*Fragaria moschata*	(Duchesne) Weston	yes	yes	no			
Fragaria vesca	*Fragaria vesca*	L.	yes	yes	yes			
Fragaria viridis	*Fragaria viridis*	(Duchesne) Weston	yes	yes	yes			
Frangula alnus	*Frangula alnus*	Mill.	yes	yes	yes			
Frangula alnus var. alnus	*Frangula alnus*	Mill.	yes	yes	yes	aggregate on species level		
Fraxinus excelsior	*Fraxinus excelsior*	L.	yes	yes	yes			
Fraxinus excelsior subsp. excelsior	*Fraxinus excelsior*	L.	yes	yes	yes	aggregate on species level		
Fraxinus ornus	*Fraxinus ornus*	L.	yes	yes	yes			
Fritillaria meleagris	*Fritillaria meleagris*	L.	yes	yes	yes			
Fumana procumbens	*Fumana procumbens*	(Dunal) Gren. & Godr.	yes	yes	yes			
Fumaria officinalis	*Fumaria officinalis*	L.	yes	yes	yes			
Fumaria officinalis subsp. officinalis	*Fumaria officinalis*	L.	yes	yes	yes	aggregate on species level		
Fumaria officinalis subsp. wirtgenii	*Fumaria officinalis*	L.	yes	yes	yes	aggregate on species level		
Fumaria parviflora	*Fumaria parviflora*	Lam.	yes	yes	yes			
Fumaria parviflora agg.	*Fumaria parviflora*	Lam.	yes	yes	yes	aggregate on species level		
Fumaria schleicheri	*Fumaria schleicheri*	Soy.-Will.	yes	yes	no			
Fumaria vaillantii	*Fumaria vaillantii*	Loisel.	yes	yes	yes			
Fumaria vaillantii subsp. schrammii	*Fumaria vaillantii*	Loisel.	yes	yes	yes	aggregate on species level		

Fumaria vaillantii subsp. vaillantii	*Fumaria vaillantii*	Loisel.	yes	yes	yes	aggregate on species level		
Gagea lutea	*Gagea lutea*	(L.) Ker Gawl.	yes	yes	yes			
Gagea pomeranica	*Gagea pomeranica*	Ruthe	yes	yes	no			
Gagea pratensis	*Gagea pratensis*	(Pers.) Dumort.	yes	yes	no			
Gagea pratensis agg.	*Gagea pratensis*	(Pers.) Dumort.	yes	yes	no	aggregate on species level		
Gagea spathacea	*Gagea spathacea*	(Hayne) Salisb.	yes	yes	no			
Gagea villosa	*Gagea villosa*	(M. Bieb.) Sweet	yes	yes	no			
Galanthus nivalis	*Galanthus nivalis*	L.	yes	yes	yes			
Galega officinalis	*Galega officinalis*	L.	yes	yes	yes			
Galeopsis angustifolia	*Galeopsis angustifolia*	Hoffm.	yes	yes	no			
Galeopsis bifida	*Galeopsis bifida*	Boenn.	yes	yes	yes			
Galeopsis ladanum	*Galeopsis ladanum*	L.	yes	yes	yes			
Galeopsis ladanum agg.	*Galeopsis ladanum*	L.	yes	yes	yes	aggregate on species level		
Galeopsis pubescens	*Galeopsis pubescens*	Besser	yes	yes	yes			
Galeopsis pubescens subsp. pubescens	*Galeopsis pubescens*	Besser	yes	yes	yes	aggregate on species level		
Galeopsis segetum	*Galeopsis segetum*	Neck.	yes	yes	yes			
Galeopsis speciosa	*Galeopsis speciosa*	Mill.	yes	yes	yes			
Galeopsis tetrahit	*Galeopsis tetrahit*	L.	yes	yes	yes			
Galeopsis tetrahit agg.	*Galeopsis tetrahit*	L.	yes	yes	yes	aggregate on species level		

Galinsoga ciliata	*Galinsoga ciliata*	(Raf.) S. F. Blake	yes	yes	yes			*Galinsoga quadriradiata*
Galinsoga parviflora	*Galinsoga parviflora*	Cav.	yes	yes	yes			
Galium album	*Galium mollugo*	Mill.	yes	yes	yes	taxonomic inconsistencies between first and second mapping		
Galium album subsp. album	*Galium mollugo*	Mill.	yes	yes	yes	taxonomic inconsistencies between first and second mapping		
Galium album subsp. pycnotrichum	*Galium mollugo*	Mill.	yes	yes	yes	taxonomic inconsistencies between first and second mapping		
Galium anisophyllon	*Galium anisophyllon*	Vill.	yes	yes	no			
Galium aparine	*Galium aparine*	L.	yes	yes	yes			
Galium aparine agg.	*Galium aparine*	L.	yes	yes	yes	aggregate on species level		
Galium boreale	*Galium boreale*	L.	yes	yes	yes			
Galium glaucum	*Galium glaucum*	L.	yes	yes	yes			
Galium mollugo	*Galium mollugo*	L.	yes	yes	yes			

Galium mollugo agg.	*Galium mollugo*	L.	yes	yes	yes	aggregate on species level		
Galium mollugo var. pubescens	*Galium mollugo*	L.	yes	yes	yes	aggregate on species level		
Galium odoratum	*Galium odoratum*	(L.) Scop.	yes	yes	yes			
Galium palustre	*Galium palustre*	L.	yes	yes	yes			
Galium palustre subsp. elongatum	*Galium palustre*	L.	yes	yes	yes	aggregate on species level		
Galium palustre subsp. palustre	*Galium palustre*	L.	yes	yes	yes	aggregate on species level		
Galium parisiense	*Galium parisiense*	L.	yes	yes	yes			
Galium parisiense var. leiocarpum	*Galium parisiense*	Tausch	yes	yes	yes	aggregate on species level		
Galium pomeranicum	*Galium pomeranicum*	Retz.	yes	yes	no		*Galium x pomeranicum*	
Galium pumilum	*Galium pumilum*	Murray	yes	yes	yes			
Galium pusillum	*Galium pusillum*	L.	yes	yes	no		Kumar et al 2022, add to node N6156	
Galium pusillum agg.	*Galium pusillum*	L.	yes	yes	no	aggregate on species level	Kumar et al 2022, add to node N6156	
Galium rotundifolium	*Galium rotundifolium*	L.	yes	yes	yes			
Galium saxatile	*Galium saxatile*	L.	yes	yes	yes			
Galium spurium	*Galium spurium*	L.	yes	yes	no			

Galium spurium subsp. spurium	*Galium spurium*	L.	yes	yes	no	aggregate on species level		
Galium spurium subsp. vaillantii	*Galium spurium*	L.	yes	yes	no	aggregate on species level		
Galium sylvaticum	*Galium sylvaticum*	L.	yes	yes	yes			
Galium sylvaticum agg.	*Galium sylvaticum*	L.	yes	yes	yes	aggregate on species level		
Galium sylvaticum f. pubescens	*Galium sylvaticum*	L.	yes	yes	yes	Import from raw data		
Galium tricornutum	*Galium tricornutum*	Dandy	yes	yes	yes			
Galium uliginosum	*Galium uliginosum*	L.	yes	yes	yes			
Galium verum	*Galium verum*	L.	yes	yes	yes			
Galium verum agg.	*Galium verum*	L.	yes	yes	yes	aggregate on species level		
Galium verum subsp. verum	*Galium verum*	L.	yes	yes	yes	aggregate on species level		
Galium wirtgenii	*Galium verum*	L.	yes	yes	yes			*Galium verum subsp. wirtgenii*
Genista anglica	*Genista anglica*	L.	yes	yes	yes			*Genista anglica*
Genista germanica	*Genista germanica*	L.	yes	yes	no			
Genista pilosa	*Genista pilosa*	L.	yes	yes	yes			
Genista tinctoria	*Genista tinctoria*	L.	yes	yes	yes			
Genista tinctoria subsp. tinctoria	*Genista tinctoria*	L.	yes	yes	yes	aggregate on species level		
Gentiana asclepiadea	*Gentiana asclepiadea*	L.	yes	yes	yes			

Gentiana cruciata	*Gentiana cruciata*	L.	yes	yes	yes			
Gentiana lutea	*Gentiana lutea*	L.	yes	yes	yes			
Gentiana pneumonanthe	*Gentiana pneumonanthe*	L.	yes	yes	yes			
Gentiana utriculosa	*Gentiana utriculosa*	L.	yes	yes	no			
Gentiana verna	*Gentiana verna*	L.	yes	yes	yes			
Gentianella campestris	*Gentianella campestris*	(L.) Boerner	yes	yes	no			
Gentianella campestris subsp. campestris	*Gentianella campestris*	(L.) Boerner	yes	yes	no	aggregate on species level		
Gentianella ciliata	*Gentianella ciliata*	L.	yes	yes	no			
Gentianella germanica	*Gentianella germanica*	Willd.	yes	yes	no			
Gentianella germanica agg.	*Gentianella germanica*	Willd.	yes	yes	no	aggregate on species level		
Geranium columbinum	*Geranium columbinum*	L.	yes	yes	yes			
Geranium dissectum	*Geranium dissectum*	L.	yes	yes	yes			
Geranium molle	*Geranium molle*	L.	yes	yes	yes			
Geranium molle agg.	*Geranium molle*	L.	yes	yes	yes	aggregate on species level		
Geranium palustre	*Geranium palustre*	L.	yes	yes	yes			
Geranium phaeum	*Geranium phaeum*	L.	yes	yes	yes			
Geranium phaeum subsp. phaeum	*Geranium phaeum*	L.	yes	yes	yes	aggregate on species level		
Geranium pratense	*Geranium pratense*	L.	yes	yes	yes			
Geranium purpureum	*Geranium purpureum*	Vill.	yes	yes	no			
Geranium pusillum	*Geranium pusillum*	Burm.f.	yes	yes	yes			
Geranium pyrenaicum	*Geranium pyrenaicum*	Burm.f.	yes	yes	yes			
Geranium robertianum	*Geranium robertianum*	L.	yes	yes	yes			

Geranium robertianum agg.	*Geranium robertianum*	L.	yes	yes	yes	aggregate on species level		
Geranium robertianum subsp. robertianum	*Geranium robertianum*	L.	yes	yes	yes	aggregate on species level		
Geranium rotundifolium	*Geranium rotundifolium*	L.	yes	yes	yes			
Geranium sanguineum	*Geranium sanguineum*	L.	yes	yes	yes			
Geranium sylvaticum	*Geranium sylvaticum*	L.	yes	yes	yes			
Geum rivale	*Geum rivale*	L.	yes	yes	yes			
Geum urbanum	*Geum urbanum*	L.	yes	yes	yes			
Gladiolus palustris	*Gladiolus palustris*	Gaudin	yes	yes	no			
Glechoma hederacea	*Glechoma hederacea*	L.	yes	yes	yes			
Glechoma hederacea agg.	*Glechoma hederacea*	NA	yes	yes	yes	aggregate on species level		
Globularia punctata	*Globularia punctata*	Lapeyr.	yes	yes	yes		*Globularia bisnagarica*	
Glyceria declinata	*Glyceria declinata*	Bréb.	yes	yes	no			
Glyceria fluitans	*Glyceria fluitans*	(L.) R. Br.	yes	yes	no			
Glyceria fluitans agg.	*Glyceria fluitans*	(L.) R. Br.	yes	yes	no	aggregate on species level		
Glyceria maxima	*Glyceria maxima*	(Hartm.) Holmb.	yes	yes	yes			
Glyceria maxima subsp. maxima	*Glyceria maxima*	(Hartm.) Holmb.	yes	yes	yes	aggregate on species level		
Glyceria notata	*Glyceria notata*	Chevall.	yes	yes	no			
Glyceria striata	*Glyceria striata*	(Lam.) Hitchc.	yes	yes	no			
Gnaphalium norvegicum	*Gnaphalium norvegicum*	Gunnerus	yes	yes	yes			
Gnaphalium sylvaticum	*Gnaphalium sylvaticum*	L.	yes	yes	yes			

Gnaphalium sylvaticum var. pumilum	*Gnaphalium sylvaticum*	L.	yes	yes	yes	aggregate on species level		
Gnaphalium uliginosum	*Gnaphalium uliginosum*	L.	yes	yes	yes			
Goodyera repens	*Goodyera repens*	(L.) R. Br.	yes	yes	yes			
Gratiola officinalis	*Gratiola officinalis*	L.	yes	yes	yes			
Groenlandia densa	*Groenlandia densa*	(L.) Fourr.	yes	yes	yes			
Gymnadenia conopsea	*Gymnadenia conopsea*	(L.) R. Br.	yes	yes	yes			
Gymnadenia conopsea agg.	*Gymnadenia conopsea*	(L.) R. Br.	yes	yes	yes	aggregate on species level		
Gymnadenia densiflora	*Gymnadenia conopsea*	(L.) R. Br.	yes	yes	yes	Rothmaler (2017)		*Gymnadenia densiflora_x*
Gymnadenia odoratissima	*Gymnadenia odoratissima*	(L.) Rich.	yes	yes	yes			
Gymnocarpium dryopteris	*Gymnocarpium dryopteris*	(L.) Newman	yes	yes	no			
Gymnocarpium robertianum	*Gymnocarpium robertianum*	(Hoffm.) Newman	yes	yes	yes			
Gypsophila muralis	*Gypsophila muralis*	L.	yes	yes	no			
Gypsophila repens	*Gypsophila repens*	L.	yes	yes	yes			
Hammarbya paludosa	*Hammarbya paludosa*	(L.) Kuntze	yes	yes	no			
Hedera helix	*Hedera helix*	L.	yes	yes	yes			
Helianthemum apenninum	*Helianthemum apenninum*	(L.) Mill.	yes	yes	no			
Helianthemum canum	*Helianthemum canum*	(L.) Baumg.	yes	yes	no			
Helianthemum nummularium	*Helianthemum nummularium*	(L.) Mill.	yes	yes	yes			
Helianthemum nummularium subsp. grandiflorum	*Helianthemum nummularium*	(L.) Mill.	yes	yes	yes	aggregate on species level		
Helianthemum nummularium subsp. nummularium	*Helianthemum nummularium*	(L.) Mill.	yes	yes	yes	aggregate on species level		

Helianthemum nummularium subsp. obscurum	*Helianthemum nummularium*	(L.) Mill.	yes	yes	yes	aggregate on species level		
Helianthemum nummularium subsp. obscurum var. fruticans	*Helianthemum nummularium*	(L.) Mill.	yes	yes	yes	aggregate on species level		
Helianthus tuberosus	*Helianthus tuberosus*	L.	yes	yes	yes			
Helichrysum arenarium	*Helichrysum arenarium*	(L.) Moench	yes	yes	yes			
Helictotrichon pratense	*Helictotrichon pratense*	(L.) Besser	yes	yes	yes			
Helictotrichon pubescens	*Helictotrichon pubescens*	(Huds.) Pilg.	yes	yes	yes			
Helictotrichon pubescens subsp. pubescens	*Helictotrichon pubescens*	(Huds.) Pilg.	yes	yes	yes	aggregate on species level		
Heliotropium europaeum	*Heliotropium europaeum*	L.	yes	yes	no			
Helleborus foetidus	*Helleborus foetidus*	L.	yes	yes	yes			
Helleborus viridis	*Helleborus viridis*	L.	yes	yes	yes			
Helleborus viridis subsp. occidentalis	*Helleborus viridis*	L.	yes	yes	yes	aggregate on species level		
Hemerocallis fulva	*Hemerocallis fulva*	(L.) L.	yes	yes	no			
Hemerocallis lilioasphodelus	*Hemerocallis lilioasphodelus*	L.	yes	yes	no			
Hepatica nobilis	*Hepatica nobilis*	Schreb.	yes	yes	yes			*Anemone hepatica*
Heracleum mantegazzianum	*Heracleum mantegazzianum*	Sommier & Levier	yes	yes	yes			
Heracleum sphondylium	*Heracleum sphondylium*	L.	yes	yes	yes			
Heracleum sphondylium subsp. elegans	*Heracleum sphondylium*	L.	yes	yes	yes	aggregate on species level		

Heracleum sphondylium subsp. sibiricum	*Heracleum sphondylium*	L.	yes	yes	yes	aggregate on species level		
Heracleum sphondylium subsp. sphondylium	*Heracleum sphondylium*	L.	yes	yes	yes	aggregate on species level		
Herminium monorchis	*Herminium monorchis*	(L.) R. Br.	yes	yes	no			
Herniaria glabra	*Herniaria glabra*	L.	yes	yes	yes			
Herniaria glabra subsp. glabra	*Herniaria glabra*	L.	yes	yes	yes	aggregate on species level		
Herniaria hirsuta	*Herniaria hirsuta*	L.	yes	yes	yes			
Herniaria hirsuta subsp. hirsuta	*Herniaria hirsuta*	L.	yes	yes	yes	aggregate on species level		
Hesperis matronalis	*Hesperis matronalis*	L.	yes	yes	yes			
Hesperis matronalis subsp. matronalis	*Hesperis matronalis*	L.	yes	yes	yes	aggregate on species level		
Hieracium aurantiacum	*Hieracium aurantiacum*	L.	yes	yes	yes			*Pilosella aurantiaca*
Hieracium aurantiacum subsp. aurantiacum	*Hieracium aurantiacum*	L.	yes	yes	yes	aggregate on species level		
Hieracium caespitosum	*Hieracium caespitosum*	Dumort.	yes	yes	yes			*Pilosella caespitosa*
Hieracium caespitosum subsp. caespitosum	*Hieracium caespitosum*	Dumort.	yes	yes	yes	aggregate on species level		
Hieracium caespitosum subsp. colliniforme	*Hieracium caespitosum*	Dumort.	yes	yes	yes	aggregate on species level		

Hieracium caespitosum subsp. dissolutum	*Hieracium caespitosum*	Dumort.	yes	yes	yes	aggregate on species level			
Hieracium caespitosum subsp. scheuerlei	*Hieracium caespitosum*	Dumort.	yes	yes	yes	aggregate on species level			
Hieracium glaucinum	*Hieracium glaucinum*	NA	yes	yes	no				
Hieracium glaucinum ssp. bounophilum	*Hieracium glaucinum*	NA	yes	yes	no	aggregate on species level			
Hieracium glaucinum subsp. basalticum	*Hieracium glaucinum*	NA	yes	yes	no	aggregate on species level			
Hieracium glaucinum subsp. bounophilum	*Hieracium glaucinum*	NA	yes	yes	no	aggregate on species level			
Hieracium glaucinum subsp. fallens	*Hieracium glaucinum*	NA	yes	yes	no	aggregate on species level			
Hieracium glaucinum subsp. fragile	*Hieracium glaucinum*	NA	yes	yes	no	aggregate on species level			
Hieracium glaucinum subsp. fraternum	*Hieracium glaucinum*	NA	yes	yes	no	aggregate on species level			
Hieracium glaucinum subsp. furcillatum	*Hieracium glaucinum*	NA	yes	yes	no	aggregate on species level			
Hieracium glaucinum subsp. glauciniforme	*Hieracium glaucinum*	NA	yes	yes	no	aggregate on species level			
Hieracium glaucinum subsp. glaucinum	*Hieracium glaucinum*	NA	yes	yes	no	aggregate on species level			
Hieracium glaucinum subsp. heteroschistum	*Hieracium glaucinum*	NA	yes	yes	no	aggregate on species level			

Hieracium glaucinum subsp. medium	*Hieracium glaucinum*	NA	yes	yes	no	aggregate on species level		
Hieracium glaucinum subsp. oegocladum	*Hieracium glaucinum*	NA	yes	yes	no	aggregate on species level		
Hieracium glaucinum subsp. oigocladum	*Hieracium glaucinum*	NA	yes	yes	no	aggregate on species level		
Hieracium glaucinum subsp. ovalifolium	*Hieracium glaucinum*	NA	yes	yes	no	aggregate on species level		
Hieracium glaucinum subsp. pallidifrons	*Hieracium glaucinum*	NA	yes	yes	no	aggregate on species level		
Hieracium glaucinum subsp. pallidulum	*Hieracium glaucinum*	NA	yes	yes	no	aggregate on species level		
Hieracium glaucinum subsp. pinicolium	*Hieracium glaucinum*	NA	yes	yes	no	aggregate on species level		
Hieracium glaucinum subsp. praecociforme	*Hieracium glaucinum*	NA	yes	yes	no	aggregate on species level		
Hieracium glaucinum subsp. praecox	*Hieracium glaucinum*	NA	yes	yes	no	aggregate on species level		
Hieracium glaucinum subsp. recensitum	*Hieracium glaucinum*	NA	yes	yes	no	aggregate on species level		
Hieracium glaucinum subsp. similatum	*Hieracium glaucinum*	NA	yes	yes	no	aggregate on species level		
Hieracium glaucinum subsp. vernum	*Hieracium glaucinum*	NA	yes	yes	no	aggregate on species level		
Hieracium lachenalii	*Hieracium lachenalii*	C. C. Gmel.	yes	yes	yes			

Hieracium lachenalii subsp. acuminatum	*Hieracium lachenalii*	C. C. Gmel.	yes	yes	yes	aggregate on species level		
Hieracium lachenalii subsp. anfractum	*Hieracium lachenalii*	C. C. Gmel.	yes	yes	yes	aggregate on species level		
Hieracium lachenalii subsp. argillaceum	*Hieracium lachenalii*	C. C. Gmel.	yes	yes	yes	aggregate on species level		
Hieracium lachenalii subsp. aurulentum	*Hieracium lachenalii*	C. C. Gmel.	yes	yes	yes	aggregate on species level		
Hieracium lachenalii subsp. brevidentatum	*Hieracium lachenalii*	C. C. Gmel.	yes	yes	yes	aggregate on species level		
Hieracium lachenalii subsp. chlorophyllum	*Hieracium lachenalii*	C. C. Gmel.	yes	yes	yes	aggregate on species level		
Hieracium lachenalii subsp. consociatum	*Hieracium lachenalii*	C. C. Gmel.	yes	yes	yes	aggregate on species level		
Hieracium lachenalii subsp. festinum	*Hieracium lachenalii*	C. C. Gmel.	yes	yes	yes	aggregate on species level		
Hieracium lachenalii subsp. irriguiceps	*Hieracium lachenalii*	C. C. Gmel.	yes	yes	yes	aggregate on species level		
Hieracium lachenalii subsp. irriguum	*Hieracium lachenalii*	C. C. Gmel.	yes	yes	yes	aggregate on species level		
Hieracium lachenalii subsp. jaccardii	*Hieracium lachenalii*	C. C. Gmel.	yes	yes	yes	aggregate on species level		
Hieracium lachenalii subsp. lachenalii	*Hieracium lachenalii*	C. C. Gmel.	yes	yes	yes	aggregate on species level		

Hieracium lachenalii subsp. melanoporphyreum	*Hieracium lachenalii*	C. C. Gmel.	yes	yes	yes	aggregate on species level		
Hieracium lachenalii subsp. mertinii	*Hieracium lachenalii*	C. C. Gmel.	yes	yes	yes	aggregate on species level		
Hieracium lachenalii subsp. perscissum	*Hieracium lachenalii*	C. C. Gmel.	yes	yes	yes	aggregate on species level		
Hieracium lachenalii subsp. pinnatifidum	*Hieracium lachenalii*	C. C. Gmel.	yes	yes	yes	aggregate on species level		
Hieracium lachenalii subsp. pseudopollichiae	*Hieracium lachenalii*	C. C. Gmel.	yes	yes	yes	aggregate on species level		
Hieracium lachenalii subsp. sciaphilum	*Hieracium lachenalii*	C. C. Gmel.	yes	yes	yes	aggregate on species level		
Hieracium lachenalii subsp. stipatiforme	*Hieracium lachenalii*	C. C. Gmel.	yes	yes	yes	aggregate on species level		
Hieracium lachenalii subsp. subacuminatum	*Hieracium lachenalii*	C. C. Gmel.	yes	yes	yes	aggregate on species level		
Hieracium lachenalii subsp. subirriguifrons	*Hieracium lachenalii*	C. C. Gmel.	yes	yes	yes	aggregate on species level		
Hieracium lachenalii subsp. subirriguum	*Hieracium lachenalii*	C. C. Gmel.	yes	yes	yes	aggregate on species level		
Hieracium lachenalii subsp. substipatiforme	*Hieracium lachenalii*	C. C. Gmel.	yes	yes	yes	aggregate on species level		
Hieracium lactucella	*Hieracium lactucella*	Wallr.	yes	yes	yes			*Pilosella lactucella*

Hieracium lactucella subsp. lactucella	*Hieracium lactucella*	Wallr.	yes	yes	yes	aggregate on species level		
Hieracium laevigatum	*Hieracium laevigatum*	Willd.	yes	yes	no			
Hieracium laevigatum subsp. abnobae	*Hieracium laevigatum*	Willd.	yes	yes	no	aggregate on species level		
Hieracium laevigatum subsp. amaurolepis	*Hieracium laevigatum*	Willd.	yes	yes	no	aggregate on species level		
Hieracium laevigatum subsp. grandidens	*Hieracium laevigatum*	Willd.	yes	yes	no	aggregate on species level		
Hieracium laevigatum subsp. laevigans	*Hieracium laevigatum*	Willd.	yes	yes	no	aggregate on species level		
Hieracium laevigatum subsp. laevigatum	*Hieracium laevigatum*	Willd.	yes	yes	no	aggregate on species level		
Hieracium maculatum	*Hieracium maculatum*	NA	yes	yes	yes			
Hieracium maculatum subsp. approximatum	*Hieracium maculatum*	NA	yes	yes	yes	aggregate on species level		
Hieracium maculatum subsp. arrectariicaule	*Hieracium maculatum*	NA	yes	yes	yes	aggregate on species level		
Hieracium maculatum subsp. commixtum	*Hieracium maculatum*	NA	yes	yes	yes	aggregate on species level		
Hieracium maculatum subsp. divisum	*Hieracium maculatum*	NA	yes	yes	yes	aggregate on species level		
Hieracium maculatum subsp. maculatum	*Hieracium maculatum*	NA	yes	yes	yes	aggregate on species level		

Hieracium maculatum subsp. onosmotrichum	*Hieracium maculatum*	NA	yes	yes	yes	aggregate on species level		
Hieracium maculatum subsp. pollichiae	*Hieracium maculatum*	NA	yes	yes	yes	aggregate on species level		
Hieracium maculatum subsp. tinctum	*Hieracium maculatum*	NA	yes	yes	yes	aggregate on species level		
Hieracium murorum	*Hieracium murorum*	L.	yes	yes	yes			
Hieracium murorum agg.	*Hieracium murorum*	L.	yes	yes	yes	aggregate on species level		
Hieracium murorum pseudogentile	*Hieracium murorum*	L.	yes	yes	no	aggregate on species level		
Hieracium murorum semisilvaticum	*Hieracium murorum*	L.	yes	yes	no	aggregate on species level		
Hieracium murorum subsp. acanthopolitanum	*Hieracium murorum*	L.	yes	yes	yes	aggregate on species level		
Hieracium murorum subsp. amaurocymum	*Hieracium murorum*	L.	yes	yes	yes	aggregate on species level		
Hieracium murorum subsp. amoenopictum	*Hieracium murorum*	L.	yes	yes	yes	aggregate on species level		
Hieracium murorum subsp. aspreticola	*Hieracium murorum*	L.	yes	yes	yes	aggregate on species level		
Hieracium murorum subsp. aspreticoliforme	*Hieracium murorum*	L.	yes	yes	yes	aggregate on species level		
Hieracium murorum subsp. bruyeranum	*Hieracium murorum*	L.	yes	yes	yes	aggregate on species level		

Hieracium murorum subsp. cacarophyllum	*Hieracium murorum*	L.	yes	yes	yes	aggregate on species level		
Hieracium murorum subsp. calvifrons	*Hieracium murorum*	L.	yes	yes	yes	aggregate on species level		
Hieracium murorum subsp. cardiophyllum	*Hieracium murorum*	L.	yes	yes	yes	aggregate on species level		
Hieracium murorum subsp. euchloroprasinum	*Hieracium murorum*	L.	yes	yes	yes	aggregate on species level		
Hieracium murorum subsp. exotericoides	*Hieracium murorum*	L.	yes	yes	yes	aggregate on species level		
Hieracium murorum subsp. exotericum	*Hieracium murorum*	L.	yes	yes	yes	aggregate on species level		
Hieracium murorum subsp. gentile	*Hieracium murorum*	L.	yes	yes	yes	aggregate on species level		
Hieracium murorum subsp. grandidens	*Hieracium murorum*	L.	yes	yes	yes	aggregate on species level		
Hieracium murorum subsp. integratum	*Hieracium murorum*	L.	yes	yes	yes	aggregate on species level		
Hieracium murorum subsp. lepistoides	*Hieracium murorum*	L.	yes	yes	yes	aggregate on species level		
Hieracium murorum subsp. microcranum	*Hieracium murorum*	L.	yes	yes	yes	aggregate on species level		
Hieracium murorum subsp. microps	*Hieracium murorum*	L.	yes	yes	yes	aggregate on species level		

Hieracium murorum subsp. micropsilon	*Hieracium murorum*	L.	yes	yes	yes	aggregate on species level		
Hieracium murorum subsp. nemorense	*Hieracium murorum*	L.	yes	yes	yes	aggregate on species level		
Hieracium murorum subsp. oblongum	*Hieracium murorum*	L.	yes	yes	yes	aggregate on species level		
Hieracium murorum subsp. perviride	*Hieracium murorum*	L.	yes	yes	yes	aggregate on species level		
Hieracium murorum subsp. pseudogentile	*Hieracium murorum*	L.	yes	yes	yes	aggregate on species level		
Hieracium murorum subsp. pseudonemorense	*Hieracium murorum*	L.	yes	yes	yes	aggregate on species level		
Hieracium murorum subsp. pseudosilvularum	*Hieracium murorum*	L.	yes	yes	yes	aggregate on species level		
Hieracium murorum subsp. scabripes	*Hieracium murorum*	L.	yes	yes	yes	aggregate on species level		
Hieracium murorum subsp. semisilvaticum	*Hieracium murorum*	L.	yes	yes	yes	aggregate on species level		
Hieracium murorum subsp. serratifolium	*Hieracium murorum*	L.	yes	yes	yes	aggregate on species level		
Hieracium murorum subsp. silvularum	*Hieracium murorum*	L.	yes	yes	yes	aggregate on species level		
Hieracium murorum subsp. subfarinellum	*Hieracium murorum*	L.	yes	yes	yes	aggregate on species level		

Hieracium murorum subsp. subnemorense	*Hieracium murorum*	L.	yes	yes	yes	aggregate on species level		
Hieracium murorum subsp. sylvularum	*Hieracium murorum*	L.	yes	yes	yes	aggregate on species level		
Hieracium murorum subsp. torticeps	*Hieracium murorum*	L.	yes	yes	yes	aggregate on species level		
Hieracium murorum subsp. viridicollum	*Hieracium murorum*	L.	yes	yes	yes	aggregate on species level		
Hieracium murorum subsp. wulfenii	*Hieracium murorum*	L.	yes	yes	yes	aggregate on species level		
Hieracium nemorense	*Hieracium murorum*	NA	yes	yes	yes	aggregate on species level		
Hieracium pilosella	*Hieracium pilosella*	L.	yes	yes	yes			*Pilosella officinarum*
Hieracium pilosella subsp. angustius	*Hieracium pilosella*	L.	yes	yes	yes	aggregate on species level		
Hieracium pilosella subsp. minuticeps	*Hieracium pilosella*	L.	yes	yes	yes	aggregate on species level		
Hieracium pilosella subsp. pilosella	*Hieracium pilosella*	L.	yes	yes	yes	aggregate on species level		
Hieracium pilosella subsp. rosulatum	*Hieracium pilosella*	L.	yes	yes	yes	aggregate on species level		
Hieracium pilosella subsp. subvirescenticeps	*Hieracium pilosella*	L.	yes	yes	yes	aggregate on species level		

Hieracium piloselloides	*Hieracium piloselloides*	Vill.	yes	yes	yes			*Pilosella piloselloides*
Hieracium piloselloides agg.	*Hieracium piloselloides*	Vill.	yes	yes	yes	aggregate on species level		
Hieracium piloselloides subsp. albidobracteum	*Hieracium piloselloides*	Vill.	yes	yes	yes	aggregate on species level		
Hieracium piloselloides subsp. alethes	*Hieracium piloselloides*	Vill.	yes	yes	yes	aggregate on species level		
Hieracium piloselloides subsp. anadenium	*Hieracium piloselloides*	Vill.	yes	yes	yes	aggregate on species level		
Hieracium piloselloides subsp. assimile	*Hieracium piloselloides*	Vill.	yes	yes	yes	aggregate on species level		
Hieracium piloselloides subsp. cymigeritropum	*Hieracium piloselloides*	Vill.	yes	yes	yes	aggregate on species level		
Hieracium piloselloides subsp. duerkhemiense	*Hieracium piloselloides*	Vill.	yes	yes	yes	aggregate on species level		
Hieracium piloselloides subsp. floccipedunculum	*Hieracium piloselloides*	Vill.	yes	yes	yes	aggregate on species level		
Hieracium piloselloides subsp. floccosum	*Hieracium piloselloides*	Vill.	yes	yes	yes	aggregate on species level		
Hieracium piloselloides subsp. herbipolitanum	*Hieracium piloselloides*	Vill.	yes	yes	yes	aggregate on species level		
Hieracium piloselloides subsp. obscurum	*Hieracium piloselloides*	Vill.	yes	yes	yes	aggregate on species level		

Hieracium piloselloides subsp. parcifloccum	*Hieracium piloselloides*	Vill.	yes	yes	yes	aggregate on species level		
Hieracium piloselloides subsp. praealtum	*Hieracium piloselloides*	Vill.	yes	yes	yes	aggregate on species level		
Hieracium piloselloides subsp. radiatum	*Hieracium piloselloides*	Vill.	yes	yes	yes	aggregate on species level		
Hieracium piloselloides subsp. subcymigerum	*Hieracium piloselloides*	Vill.	yes	yes	yes	aggregate on species level		
Hieracium sabaudum	*Hieracium sabaudum*	L.	yes	yes	yes			
Hieracium sabaudum subsp. concinnum	*Hieracium sabaudum*	L.	yes	yes	yes	aggregate on species level		
Hieracium sabaudum subsp. nemorivagum	*Hieracium sabaudum*	L.	yes	yes	yes	aggregate on species level		
Hieracium sabaudum subsp. quercetorum	*Hieracium sabaudum*	L.	yes	yes	yes	aggregate on species level		
Hieracium sabaudum subsp. rigidicaule	*Hieracium sabaudum*	L.	yes	yes	yes	aggregate on species level		
Hieracium sabaudum subsp. sedunense	*Hieracium sabaudum*	L.	yes	yes	yes	aggregate on species level		
Hieracium sabaudum subsp. sublactucaceum	*Hieracium sabaudum*	L.	yes	yes	yes	aggregate on species level		
Hieracium sabaudum subsp. subrectum	*Hieracium sabaudum*	L.	yes	yes	yes	aggregate on species level		
Hieracium sabaudum subsp. vagum	*Hieracium sabaudum*	L.	yes	yes	yes	aggregate on species level		

Hieracium sabaudum subsp. virgultorum	*Hieracium sabaudum*	L.	yes	yes	yes	aggregate on species level		
Hieracium sabaudum subspec. rigidiforme	*Hieracium sabaudum*	L.	yes	yes	yes	aggregate on species level		
Hieracium sylvaticum	*Hieracium murorum*	L.	yes	yes	yes	Buttler & Hand (2023)		
Hieracium umbellatum	*Hieracium umbellatum*	L.	yes	yes	yes			
Hieracium umbellatum subsp. umbellatum	*Hieracium umbellatum*	L.	yes	yes	yes	aggregate on species level		
Hierarcium murorum subsp. contaminatus	*Hieracium murorum*	L.	yes	yes	yes	aggregate on species level		
Himantoglossum hircinum	*Himantoglossum hircinum*	(L.) Spreng.	yes	yes	no			
Hippocrepis comosa	*Hippocrepis comosa*	L.	yes	yes	yes			
Hippocrepis emerus	*Hippocrepis emerus*	(L.) Lassen	yes	yes	no			
Hippophae rhamnoides	*Hippophae rhamnoides*	L.	yes	yes	yes			
Hippophae rhamnoides subsp. fluviatilis	*Hippophae rhamnoides*	L.	yes	yes	yes	aggregate on species level		
Hippuris vulgaris	*Hippuris vulgaris*	L.	yes	yes	no			
Holcus lanatus	*Holcus lanatus*	L.	yes	yes	yes			
Holcus mollis	*Holcus mollis*	L.	yes	yes	yes			
Holcus mollis subsp. mollis	*Holcus mollis*	L.	yes	yes	yes	Import from raw data		
Holosteum umbellatum	*Holosteum umbellatum*	L.	yes	yes	yes			
Holosteum umbellatum subsp. umbellatum	*Holosteum umbellatum*	L.	yes	yes	yes	aggregate on species level		
Homogyne alpina	*Homogyne alpina*	(L.) Cass.	yes	yes	yes			

Hordelymus europaeus	*Hordelymus europaeus*	(L.) Jessen ex Harz	yes	yes	yes			
Hordeum jubatum	*Hordeum jubatum*	L.	yes	yes	yes			
Hordeum murinum	*Hordeum murinum*	L.	yes	yes	yes			
Hordeum murinum agg.	*Hordeum murinum*	L.	yes	yes	yes	aggregate on species level		
Hordeum murinum subsp. leporinum	*Hordeum murinum*	L.	yes	yes	yes	aggregate on species level		
Hordeum murinum subsp. murinum	*Hordeum murinum*	L.	yes	yes	yes	aggregate on species level		
Hottonia palustris	*Hottonia palustris*	L.	yes	yes	no			
Humulus lupulus	*Humulus lupulus*	L.	yes	yes	yes			
Huperzia selago	*Huperzia selago*	(L.) Bernh. ex Schrank & Martens	yes	yes	no			
Hydrocharis morsus-ranae	*Hydrocharis morsus-ranae*	L.	yes	yes	yes			*HYDROCHARIS MORSUS-RANAE*
Hydrocotyle vulgaris	*Hydrocotyle vulgaris*	L.	yes	yes	yes			
Hyoscyamus niger	*Hyoscyamus niger*	L.	yes	yes	yes			
Hypericum desetangsii subsp. desetangsii	*Hypericum desetangsii*	Lamotte	yes	yes	no	aggregate on species level	*Hypericum x desetangsii*	
Hypericum hirsutum	*Hypericum hirsutum*	L.	yes	yes	yes			
Hypericum humifusum	*Hypericum humifusum*	L.	yes	yes	yes			
Hypericum maculatum	*Hypericum maculatum*	Crantz	yes	yes	yes			

Hypericum maculatum agg.	*Hypericum maculatum*	Crantz	yes	yes	yes	aggregate on species level		
Hypericum maculatum subsp. maculatum	*Hypericum maculatum*	Crantz	yes	yes	yes	aggregate on species level		
Hypericum maculatum subsp. obtusiusculum	*Hypericum maculatum*	Crantz	yes	yes	yes	aggregate on species level		
Hypericum montanum	*Hypericum montanum*	L.	yes	yes	yes			
Hypericum perforatum	*Hypericum perforatum*	L.	yes	yes	yes			
Hypericum perforatum subsp. angustifolium	*Hypericum perforatum*	L.	yes	yes	yes	aggregate on species level		
Hypericum perforatum subsp. perforatum	*Hypericum perforatum*	L.	yes	yes	yes	aggregate on species level		
Hypericum perforatum subsp. perforatum	*Hypericum perforatum*	L.	yes	yes	yes	aggregate on species level		
Hypericum perforatum subsp. veronense	*Hypericum perforatum*	L.	yes	yes	yes	aggregate on species level		
Hypericum perforatum var. angustifolium	*Hypericum perforatum*	L.	yes	yes	yes	aggregate on species level		
Hypericum perforatum var. microphyllum	*Hypericum perforatum*	L.	yes	yes	yes	aggregate on species level		
Hypericum perforatum var. perforatum	*Hypericum perforatum*	L.	yes	yes	yes	aggregate on species level		
Hypericum pulchrum	*Hypericum pulchrum*	L.	yes	yes	yes			
Hypericum tetrapterum	*Hypericum tetrapterum*	Fr.	yes	yes	yes			
Hypochaeris glabra	*Hypochaeris glabra*	L.	yes	yes	yes			

Hypochaeris maculata	*Hypochaeris maculata*	L.	yes	yes	yes			
Hypochaeris radicata	*Hypochaeris radicata*	L.	yes	yes	yes			
Iberis amara	*Iberis amara*	L.	yes	yes	no			
Ilex aquifolium	*Ilex aquifolium*	L.	yes	yes	yes			
Illecebrum verticillatum	*Illecebrum verticillatum*	L.	yes	yes	yes			
Impatiens glandulifera	*Impatiens glandulifera*	Royle	yes	yes	yes			
Impatiens noli-tangere	*Impatiens noli-tangere*	L.	yes	yes	yes			*IMPATIENS NOLI-TANGERE*
Impatiens parviflora	*Impatiens parviflora*	DC.	yes	yes	yes			
Inula britannica	*Inula britannica*	L.	yes	yes	no			
Inula conyzae	*Inula conyzae*	DC.	yes	yes	yes			*Inula conyza*
Inula helenium	*Inula helenium*	L.	yes	yes	yes			
Inula hirta	*Inula hirta*	L.	yes	yes	yes			*Pentanema hirtum*
Inula salicina	*Inula salicina*	L.	yes	yes	yes			
Iris germanica	*Iris germanica*	L.	yes	yes	yes			*Iris germanica_x*
Iris germanica agg.	*Iris germanica*	L.	yes	yes	yes	aggregate on species level		
Iris pseudacorus	*Iris pseudacorus*	L.	yes	yes	yes			
Iris sambucina agg.	*Iris sambucina*	L.	yes	yes	no	aggregate on species level		
Iris sibirica	*Iris sibirica*	L.	yes	yes	yes			
Iris variegata	*Iris variegata*	L.	yes	yes	no			
Isatis tinctoria	*Isatis tinctoria*	L.	yes	yes	yes			
Isatis tinctoria subsp. tinctoria	*Isatis tinctoria*	L.	yes	yes	yes	aggregate on species level		
Isoetes echinospora	*Isoetes echinospora*	L.	yes	yes	no			
Isoetes lacustris	*Isoetes lacustris*	L.	yes	yes	no			
Isolepis setacea	*Isolepis setacea*	L.	yes	yes	yes			
Iva xanthiifolia	*Iva xanthiifolia*	Nutt.	yes	yes	yes			
Jasione laevis	*Jasione laevis*	Lam.	yes	yes	no			

Jasione montana	*Jasione montana*	L.	yes	yes	yes			
Jovibarba globifera subsp. globifera	*Jovibarba globifera*	(L.) J. Parn.	yes	yes	no	aggregate on species level		
Juglans regia	*Juglans regia*	L.	yes	yes	yes			
Juncus acutiflorus	*Juncus acutiflorus*	Ehrh. ex Hoffm.	yes	yes	yes			
Juncus alpinus	*Juncus alpinus*	Chaix	yes	yes	yes			*Juncus alpinoarticulatus*
Juncus articulatus	*Juncus articulatus*	L.	yes	yes	yes			
Juncus articulatus subsp. articulatus	*Juncus articulatus*	L.	yes	yes	yes	aggregate on species level		
Juncus bufonius	*Juncus bufonius*	L.	yes	yes	yes			
Juncus bufonius agg.	*Juncus bufonius*	L.	yes	yes	yes	aggregate on species level		
Juncus bulbosus	*Juncus bulbosus*	L.	yes	yes	yes			
Juncus bulbosus subsp. bulbosus	*Juncus bulbosus*	L.	yes	yes	yes	aggregate on species level		
Juncus compressus	*Juncus compressus*	Jacq.	yes	yes	yes			
Juncus compressus agg.	*Juncus compressus*	Jacq.	yes	yes	yes	aggregate on species level		
Juncus conglomeratus	*Juncus conglomeratus*	L.	yes	yes	yes			
Juncus effusus	*Juncus effusus*	L.	yes	yes	yes			
Juncus filiformis	*Juncus filiformis*	L.	yes	yes	yes			
Juncus inflexus	*Juncus inflexus*	L.	yes	yes	yes			
Juncus sphaerocarpus	*Juncus sphaerocarpus*	Nees	yes	yes	yes			
Juncus squarrosus	*Juncus squarrosus*	L.	yes	yes	yes			
Juncus stygius	*Juncus stygius*	L.	yes	yes	no			
Juncus subnodulosus	*Juncus subnodulosus*	Schrank	yes	yes	yes			
Juncus tenageia	*Juncus tenageia*	Ehrh.	yes	yes	yes			
Juncus tenuis	*Juncus tenuis*	Willd.	yes	yes	yes			

Juniperus communis	*Juniperus communis*	L.	yes	yes	yes			
Juniperus communis subsp. communis	*Juniperus communis*	L.	yes	yes	yes	aggregate on species level		
Jurinea cyanoides	*Jurinea cyanoides*	(L.) Rchb.	yes	yes	no			
Kernera saxatilis	*Kernera saxatilis*	(L.) Sweet	yes	yes	yes			
Kerria japonica	*Kerria japonica*	(Thunb.) DC.	yes	yes	no			
Kickxia elatine	*Kickxia elatine*	(L.) Dumort.	yes	yes	yes			
Kickxia elatine subsp. elatine	*Kickxia elatine*	(L.) Dumort.	yes	yes	yes	aggregate on species level		
Kickxia spuria	*Kickxia spuria*	(L.) Dumort.	yes	yes	yes			
Knautia arvensis	*Knautia arvensis*	(L.) Coult.	yes	yes	yes			
Knautia arvensis agg.	*Knautia arvensis*	(L.) Coult.	yes	yes	yes	aggregate on species level		
Knautia dipsacifolia	*Knautia dipsacifolia*	Kreutzer	yes	yes	yes			
Knautia dipsacifolia subsp. dipsacifolia	*Knautia dipsacifolia*	Kreutzer	yes	yes	yes	aggregate on species level		
Koeleria glauca	*Koeleria glauca*	(Spreng.) DC.	yes	yes	yes			
Koeleria macrantha	*Koeleria macrantha*	(Ledeb.) Schult.	yes	yes	yes			
Koeleria pyramidata	*Koeleria pyramidata*	(Lam.) P. Beauv.	yes	yes	yes			
Koeleria pyramidata agg.	*Koeleria pyramidata*	(Lam.) P. Beauv.	yes	yes	yes	aggregate on species level		
Koeleria pyramidata subsp. pyramidata	*Koeleria pyramidata*	(Lam.) P. Beauv.	yes	yes	yes	aggregate on species level		
Laburnum anagyroides	*Laburnum anagyroides*	Medik.	yes	yes	yes			
Lactuca perennis	*Lactuca perennis*	L.	yes	yes	yes			

Lactuca saligna	*Lactuca saligna*	L.	yes	yes	yes			
Lactuca serriola	*Lactuca serriola*	L.	yes	yes	yes			
Lactuca virosa	*Lactuca virosa*	L.	yes	yes	yes			
Lamium album	*Lamium album*	L.	yes	yes	yes			
Lamium album subsp. album	*Lamium album*	L.	yes	yes	yes	aggregate on species level		
Lamium amplexicaule	*Lamium amplexicaule*	L.	yes	yes	yes			
Lamium argentatum	*Lamium galeobdolon*	(L.) L.	yes	yes	yes	Buttler & Hand (2023)		*Lamium galeobdolon*
Lamium endtmannii	*Lamium galeobdolon*	(L.) L.	yes	yes	yes	aggregate on species level		
Lamium galeobdolon	*Lamium galeobdolon*	(L.) L.	yes	yes	yes			
Lamium galeobdolon agg.	*Lamium galeobdolon*	(L.) L.	yes	yes	yes	aggregate on species level		
Lamium galeobdolon subsp. argentatum	*Lamium galeobdolon*	(L.) L.	yes	yes	yes	aggregate on species level		
Lamium maculatum	*Lamium maculatum*	L.	yes	yes	yes			
Lamium maculatum var. album	*Lamium maculatum*	L.	yes	yes	yes	aggregate on species level		
Lamium montanum	*Lamium galeobdolon*	(L.) L.	yes	yes	yes	Buttler & Hand (2023)		*Lamium galeobdolon*
Lamium purpureum	*Lamium purpureum*	L.	yes	yes	yes			
Lamium purpureum f. rosea	*Lamium purpureum*	L.	yes	yes	yes	aggregate on species level		
Lamium purpureum var. incisum	*Lamium purpureum*	L.	yes	yes	yes	aggregate on species level		

Lamium purpureum var. molucellifolium	*Lamium purpureum*	L.	yes	yes	yes	aggregate on species level		
Lamium purpureum var. purpureum	*Lamium purpureum*	L.	yes	yes	yes	aggregate on species level		
Lappula deflexa	*Lappula deflexa*	(Wahlenb.) Garcke	yes	yes	no			
Lappula squarrosa	*Lappula squarrosa*	(Retz.) Dumort.	yes	yes	yes			
Lapsana communis	*Lapsana communis*	L.	yes	yes	yes			
Lapsana communis subsp. communis	*Lapsana communis*	L.	yes	yes	yes	aggregate on species level		
Larix decidua	*Larix decidua*	Mill.	yes	yes	yes			
Laserpitium latifolium	*Laserpitium latifolium*	L.	yes	yes	yes			
Laserpitium prutenicum	*Laserpitium prutenicum*	L.	yes	yes	no			
Laserpitium siler	*Laserpitium siler*	L.	yes	yes	no			
Lathraea squamaria	*Lathraea squamaria*	L.	yes	yes	no			
Lathraea squamaria subsp. squamaria	*Lathraea squamaria*	L.	yes	yes	no	aggregate on species level		
Lathraea squamaria subsp. tatrica	*Lathraea squamaria*	L.	yes	yes	no	aggregate on species level		
Lathyrus aphaca	*Lathyrus aphaca*	L.	yes	yes	no			
Lathyrus bauhini	*Lathyrus bauhini*	Genty	yes	yes	no			
Lathyrus heterophyllus	*Lathyrus heterophyllus*	L.	yes	yes	yes			
Lathyrus hirsutus	*Lathyrus hirsutus*	L.	yes	yes	no			
Lathyrus latifolius	*Lathyrus latifolius*	L.	yes	yes	yes			
Lathyrus linifolius	*Lathyrus linifolius*	(Reichard) Baessler	yes	yes	yes			
Lathyrus niger	*Lathyrus niger*	(L.) Bernh.	yes	yes	no			

Lathyrus nissolia	*Lathyrus nissolia*	L.	yes	yes	yes			
Lathyrus palustris	*Lathyrus palustris*	L.	yes	yes	yes			
Lathyrus pannonicus	*Lathyrus pannonicus*	(Jacq.) Garcke	yes	yes	no			
Lathyrus pannonicus subsp. collinus	*Lathyrus pannonicus*	(Jacq.) Garcke	yes	yes	no	aggregate on species level		
Lathyrus pratensis	*Lathyrus pratensis*	L.	yes	yes	yes			
Lathyrus sylvestris	*Lathyrus sylvestris*	L.	yes	yes	no			
Lathyrus sylvestris subsp. angustifolius	*Lathyrus sylvestris*	L.	yes	yes	no	aggregate on species level		
Lathyrus sylvestris subsp. platyphyllos	*Lathyrus sylvestris*	L.	yes	yes	no	aggregate on species level		
Lathyrus sylvestris subsp. sylvestris	*Lathyrus sylvestris*	L.	yes	yes	no	aggregate on species level		
Lathyrus tuberosus	*Lathyrus tuberosus*	L.	yes	yes	yes			
Lathyrus vernus	*Lathyrus vernus*	(L.) Bernh.	yes	yes	yes			
Lavatera thuringiaca	*Lavatera thuringiaca*	L.	yes	yes	no			
Ledum palustre	*Ledum palustre*	L.	yes	yes	yes			*Rhododendron tomentosum*
Leersia oryzoides	*Leersia oryzoides*	(L.) Sw.	yes	yes	yes			
Legousia hybrida	*Legousia hybrida*	(L.) Delarbre	yes	yes	yes			
Legousia speculum-veneris	*Legousia speculum-veneris*	(L.) Chaix	yes	yes	yes			
Lemna gibba	*Lemna gibba*	L.	yes	yes	no			
Lemna minor	*Lemna minor*	L.	yes	yes	no			
Lemna minuta	*Lemna minuta*	Humb.. Bonpl. & Kunth	yes	yes	no			
Lemna trisulca	*Lemna trisulca*	L.	yes	yes	no			

Leontodon autumnalis	*Leontodon autumnalis*	L.	yes	yes	yes			*Scorzoneroides autumnalis*
Leontodon autumnalis subsp. autumnalis	*Leontodon autumnalis*	L.	yes	yes	yes	aggregate on species level		
Leontodon autumnalis subsp. pratensis	*Leontodon autumnalis*	L.	yes	yes	yes	aggregate on species level		
Leontodon helveticus	*Leontodon helveticus*	Mérat	yes	yes	no			
Leontodon hispidus	*Leontodon hispidus*	L.	yes	yes	yes			
Leontodon hispidus subsp. danubialis	*Leontodon hispidus*	L.	yes	yes	yes	aggregate on species level		
Leontodon hispidus subsp. hispidus	*Leontodon hispidus*	L.	yes	yes	yes	aggregate on species level		
Leontodon hispidus subsp. hyoseroides	*Leontodon hispidus*	L.	yes	yes	yes	aggregate on species level		
Leontodon incanus	*Leontodon incanus*	(L.) Schrank	yes	yes	yes			
Leontodon saxatilis	*Leontodon saxatilis*	Lam.	yes	yes	no			
Leonurus cardiaca	*Leonurus cardiaca*	L.	yes	yes	no			
Leonurus cardiaca subsp. cardiaca	*Leonurus cardiaca*	L.	yes	yes	no	aggregate on species level		
Leonurus cardiaca subsp. villosus	*Leonurus cardiaca*	L.	yes	yes	no	aggregate on species level		
Lepidium campestre	*Lepidium campestre*	(L.) R. Br.	yes	yes	yes			
Lepidium densiflorum	*Lepidium densiflorum*	Schrad.	yes	yes	yes			
Lepidium graminifolium	*Lepidium graminifolium*	L.	yes	yes	no			

Lepidium heterophyllum	*Lepidium heterophyllum*	Benth.	yes	yes	no			
Lepidium latifolium	*Lepidium latifolium*	L.	yes	yes	yes			
Lepidium neglectum	*Lepidium neglectum*	Thell.	yes	yes	yes			*Lepidium densiflorum*
Lepidium ruderale	*Lepidium ruderale*	L.	yes	yes	yes			
Lepidium virginicum	*Lepidium virginicum*	L.	yes	yes	yes			
Leucanthemum adustum	*Leucanthemum adustum*	(W. D. J. Koch) Gremli	yes	yes	yes			
Leucanthemum ircutianum	*Leucanthemum vulgare*	Lam.	yes	yes	yes	change in taxonomy between first and second mapping		
Leucanthemum vulgare	*Leucanthemum vulgare*	Lam.	yes	yes	yes			
Leucanthemum vulgare agg.	*Leucanthemum vulgare*	Lam.	yes	yes	yes	aggregate on species level		
Leucojum vernum	*Leucojum vernum*	L.	yes	yes	yes			
Ligusticum mutellina	*Ligusticum mutellina*	(L.) Crantz	yes	yes	yes			
Ligustrum vulgare	*Ligustrum vulgare*	L.	yes	yes	yes			
Lilium bulbiferum	*Lilium bulbiferum*	L.	yes	yes	yes			
Lilium bulbiferum subsp. croceum	*Lilium bulbiferum*	L.	yes	yes	yes	aggregate on species level		
Lilium martagon	*Lilium martagon*	L.	yes	yes	yes			
Limodorum abortivum	*Limodorum abortivum*	(L.) Sw.	yes	yes	no			
Limosella aquatica	*Limosella aquatica*	L.	yes	yes	yes			
Linaria alpina	*Linaria alpina*	(L.) Mill.	yes	yes	yes			
Linaria repens	*Linaria repens*	(L.) Mill.	yes	yes	yes			

Linaria vulgaris	*Linaria vulgaris*	Mill.	yes	yes	yes			
Linaria vulgaris agg.	*Linaria vulgaris*	Mill.	yes	yes	yes	aggregate on species level		
Lindernia procumbens	*Lindernia procumbens*	(Krock.) Borbás	yes	yes	no			
Linum austriacum	*Linum austriacum*	L.	yes	yes	yes			
Linum bienne	*Linum bienne*	Mill.	yes	yes	no			
Linum catharticum	*Linum catharticum*	L.	yes	yes	yes			
Linum catharticum subsp. catharticum	*Linum catharticum*	L.	yes	yes	yes	aggregate on species level		
Linum catharticum subsp. suecicum	*Linum catharticum*	L.	yes	yes	yes	aggregate on species level		
Linum flavum	*Linum flavum*	L.	yes	yes	no			
Linum perenne	*Linum perenne*	L.	yes	yes	yes			
Linum perenne agg.	*Linum perenne*	L.	yes	yes	yes	aggregate on species level		
Linum perenne subsp. perenne	*Linum perenne*	L.	yes	yes	yes	aggregate on species level		
Linum tenuifolium	*Linum tenuifolium*	L.	yes	yes	no			
Linum usitatissimum	*Linum usitatissimum*	L.	yes	yes	yes			
Liparis loeselii	*Liparis loeselii*	(L.) Rich.	yes	yes	yes			
Listera cordata	*Listera cordata*	(L.) R. Br.	yes	yes	no			
Listera ovata	*Listera ovata*	(L.) R. Br.	yes	yes	yes			*Neottia ovata*
Lithospermum arvense	*Lithospermum arvense*	(L.) I. M. Johnst.	yes	yes	no		*Buglossoides arvensis*	
Lithospermum arvense subsp. arvense	*Lithospermum arvense*	(L.) I. M. Johnst.	yes	yes	no	aggregate on species level	*Buglossoides arvensis*	

Lithospermum arvense subsp. sibthorpianum	*Lithospermum arvense*	(L.) I. M. Johnst.	yes	yes	no	aggregate on species level	*Buglossoides arvensis*	
Lithospermum officinale	*Lithospermum officinale*	L.	yes	yes	yes			
Lithospermum purpurocaeruleum	*Lithospermum purpurocaeruleum*	L.	yes	yes	yes		*Buglossoides purpurocaerulea*	*Buglossoides purpurocaerulea*
Littorella uniflora	*Littorella uniflora*	(L.) Asch.	yes	yes	yes			*Plantago uniflora*
Lolium multiflorum	*Lolium multiflorum*	Lam.	yes	yes	yes			
Lolium perenne	*Lolium perenne*	L.	yes	yes	yes			
Lolium temulentum	*Lolium temulentum*	L.	yes	yes	yes			
Lolium temulentum agg.	*Lolium temulentum*	L.	yes	yes	yes	aggregate on species level		
Lonicera alpigena	*Lonicera alpigena*	L.	yes	yes	yes			
Lonicera caerulea	*Lonicera caerulea*	L.	yes	yes	yes			
Lonicera caprifolium	*Lonicera caprifolium*	L.	yes	yes	yes			
Lonicera nigra	*Lonicera nigra*	L.	yes	yes	no			
Lonicera periclymenum	*Lonicera periclymenum*	L.	yes	yes	yes			
Lonicera xylosteum	*Lonicera xylosteum*	L.	yes	yes	yes			
Lotus corniculatus	*Lotus corniculatus*	L.	yes	yes	yes			
Lotus corniculatus agg.	*Lotus corniculatus*	L.	yes	yes	yes	aggregate on species level		
Lotus corniculatus f. hirsutus	*Lotus corniculatus*	Walther	yes	yes	yes			*Lotus corniculatus*
Lotus corniculatus subsp. corniculatus	*Lotus corniculatus*	L.	yes	yes	yes	aggregate on species level		

Lotus corniculatus subsp. hirsutus	*Lotus corniculatus*	L.	yes	yes	yes	aggregate on species level		
Lotus corniculatus subsp. sativus	*Lotus corniculatus*	L.	yes	yes	yes	aggregate on species level		
Lotus corniculatus var. alpicola	*Lotus corniculatus*	L.	yes	yes	yes	aggregate on species level		
Lotus corniculatus var. corniculatus	*Lotus corniculatus*	L.	yes	yes	yes	aggregate on species level		
Lotus corniculatus var. hirsutus	*Lotus corniculatus*	L.	yes	yes	yes	aggregate on species level		
Lotus corniculatus var. kochii	*Lotus corniculatus*	L.	yes	yes	yes	aggregate on species level		
Lotus corniculatus var. sativus	*Lotus corniculatus*	L.	yes	yes	yes	aggregate on species level		
Lotus pedunculatus	*Lotus pedunculatus*	Cav.	yes	yes	yes			
Lotus tenuis	*Lotus corniculatus*	Waldst. & Kit. ex Willd.	yes	yes	yes			
Ludwigia palustris	*Ludwigia palustris*	(L.) Elliot	yes	yes	yes			
Lunaria annua	*Lunaria annua*	L.	yes	yes	yes			
Lunaria rediviva	*Lunaria rediviva*	L.	yes	yes	yes			
Lupinus polyphyllus	*Lupinus polyphyllus*	Lindl.	yes	yes	yes			
Luzula campestris	*Luzula campestris*	(L.) DC.	yes	yes	yes			
Luzula campestris agg.	*Luzula campestris*	(L.) DC.	yes	yes	yes	aggregate on species level		
Luzula congesta	*Luzula congesta*	(Thuill.) Lej.	yes	yes	no			
Luzula desvauxii	*Luzula desvauxii*	Kunth	yes	yes	no			
Luzula forsteri	*Luzula forsteri*	(Sm.) DC.	yes	yes	yes			

Luzula luzulina	*Luzula luzulina*	(Vill.) Dalla Torre & Sarnth.	yes	yes	no			
Luzula luzuloides	*Luzula luzuloides*	(Lam.) Dandy & Wilm.	yes	yes	yes			
Luzula luzuloides subsp. luzuloides	*Luzula luzuloides*	(Lam.) Dandy & Wilm.	yes	yes	yes	aggregate on species level		
Luzula luzuloides subsp. rubella	*Luzula luzuloides*	(Lam.) Dandy & Wilm.	yes	yes	yes	aggregate on species level		
Luzula multiflora	*Luzula multiflora*	(Ehrh.) Lej.	yes	yes	yes			
Luzula multiflora subsp. multiflora	*Luzula multiflora*	(Ehrh.) Lej.	yes	yes	yes	aggregate on species level		
Luzula pilosa	*Luzula pilosa*	(L.) Willd.	yes	yes	yes			
Luzula sudetica	*Luzula sudetica*	(Willd.) Schult.	yes	yes	yes			
Luzula sylvatica	*Luzula sylvatica*	(Huds.) Gaudin	yes	yes	yes			
Luzula sylvatica subsp. sylvatica	*Luzula sylvatica*	(Huds.) Gaudin	yes	yes	yes	aggregate on species level		
Lycium barbarum	*Lycium barbarum*	L.	yes	yes	yes			
Lycium chinense	*Lycium chinense*	Mill.	yes	yes	no			
Lycopodiella inundata	*Lycopodiella inundata*	(L.) Holub	yes	yes	no			
Lycopodium annotinum	*Lycopodium annotinum*	L.	yes	yes	no			
Lycopodium annotinum subsp. annotinum	*Lycopodium annotinum*	L.	yes	yes	no	aggregate on species level		
Lycopodium clavatum	*Lycopodium clavatum*	L.	yes	yes	no			
Lycopus europaeus	*Lycopus europaeus*	L.	yes	yes	yes			
Lycopus europaeus subsp. europaeus	*Lycopus europaeus*	L.	yes	yes	yes	aggregate on species level		

Lysimachia nemorum	*Lysimachia nemorum*	L.	yes	yes	yes			
Lysimachia nummularia	*Lysimachia nummularia*	L.	yes	yes	yes			
Lysimachia punctata	*Lysimachia punctata*	L.	yes	yes	yes			
Lysimachia thyrsiflora	*Lysimachia thyrsiflora*	L.	yes	yes	yes			
Lysimachia vulgaris	*Lysimachia vulgaris*	L.	yes	yes	yes			
Lythrum hyssopifolia	*Lythrum hyssopifolia*	L.	yes	yes	yes			
Lythrum salicaria	*Lythrum salicaria*	L.	yes	yes	yes			
Mahonia aquifolium	*Mahonia aquifolium*	(Pursh) Nutt.	yes	yes	yes			*Berberis aquifolium*
Maianthemum bifolium	*Maianthemum bifolium*	(L.) F. W. Schmidt	yes	yes	yes			
Malaxis monophyllos	*Malaxis monophyllos*	(L.) Sw.	yes	yes	no			
Malus domestica	*Malus domestica*	Borkh.	yes	yes	yes			
Malus sylvestris	*Malus sylvestris*	(L.) Mill.	yes	yes	yes			
Malus sylvestris agg.	*Malus sylvestris*	(L.) Mill.	yes	yes	yes	aggregate on species level		
Malva alcea	*Malva alcea*	L.	yes	yes	yes			
Malva moschata	*Malva moschata*	L.	yes	yes	yes			
Malva neglecta	*Malva neglecta*	Wallr.	yes	yes	yes			
Malva pusilla	*Malva pusilla*	Sm.	yes	yes	yes			
Malva sylvestris	*Malva sylvestris*	L.	yes	yes	yes			
Malva sylvestris subsp. mauritiana	*Malva sylvestris*	L.	yes	yes	yes	aggregate on species level		
Malva sylvestris subsp. sylvestris	*Malva sylvestris*	L.	yes	yes	yes	aggregate on species level		
Malva verticillata	*Malva verticillata*	L.	yes	yes	yes			
Marrubium vulgare	*Marrubium vulgare*	L.	yes	yes	yes			
Marsilea quadrifolia	*Marsilea quadrifolia*	L.	yes	yes	no			

Matricaria discoidea	*Matricaria discoidea*	DC.	yes	yes	yes			
Matricaria recutita	*Matricaria recutita*	L.	yes	yes	yes			*Matricaria chamomilla*
Matteuccia struthiopteris	*Matteuccia struthiopteris*	(L.) Tod.	yes	yes	no			
Medicago arabica	*Medicago arabica*	(L.) Huds.	yes	yes	yes			
Medicago falcata	*Medicago falcata*	L.	yes	yes	yes			
Medicago lupulina	*Medicago lupulina*	L.	yes	yes	yes			
Medicago minima	*Medicago minima*	(L.) L.	yes	yes	yes			
Medicago sativa	*Medicago sativa*	L.	yes	yes	yes			
Medicago sativa agg.	*Medicago sativa*	L.	yes	yes	yes	aggregate on species level		
Medicago varia	*Medicago sativa*	L.	yes	yes	yes	taxonomic inconsistencies between first and second mapping		
Melampyrum arvense	*Melampyrum arvense*	L.	yes	yes	yes			
Melampyrum cristatum	*Melampyrum cristatum*	L.	yes	yes	yes			
Melampyrum pratense	*Melampyrum pratense*	L.	yes	yes	yes			
Melampyrum pratense subsp. commutatum	*Melampyrum pratense*	L.	yes	yes	yes	aggregate on species level		
Melampyrum pratense subsp. pratense	*Melampyrum pratense*	L.	yes	yes	yes	aggregate on species level		
Melampyrum sylvaticum	*Melampyrum sylvaticum*	L.	yes	yes	yes			
Melica ciliata	*Melica ciliata*	L.	yes	yes	yes			

Melica ciliata agg.	*Melica ciliata*	L.	yes	yes	yes	aggregate on species level		
Melica ciliata subsp. ciliata	*Melica ciliata*	L.	yes	yes	yes	aggregate on species level		
Melica ciliata subsp. nebrodensis	*Melica ciliata*	L.	yes	yes	yes	aggregate on species level		
Melica nutans	*Melica nutans*	L.	yes	yes	yes			
Melica picta	*Melica picta*	K. Koch	yes	yes	no			
Melica transsilvanica	*Melica transsilvanica*	Schur	yes	yes	yes			
Melica uniflora	*Melica uniflora*	Retz.	yes	yes	yes			
Melilotus albus	*Melilotus albus*	Medik.	yes	yes	yes			*Trigonella alba*
Melilotus altissimus	*Melilotus altissimus*	Thuill.	yes	yes	yes			*Trigonella altissima*
Melilotus officinalis	*Melilotus officinalis*	(L.) Lam.	yes	yes	yes			*Trigonella officinalis*
Melissa officinalis	*Melissa officinalis*	L.	yes	yes	no			
Melittis melissophyllum	*Melittis melissophyllum*	L.	yes	yes	yes			
Mentha aquatica	*Mentha aquatica*	L.	yes	yes	yes			
Mentha aquatica agg.	*Mentha aquatica*	L.	yes	yes	yes	aggregate on species level		
Mentha arvensis	*Mentha arvensis*	L.	yes	yes	yes			
Mentha arvensis agg.	*Mentha arvensis*	L.	yes	yes	yes	aggregate on species level		
Mentha arvensis subsp. austriaca	*Mentha arvensis*	L.	yes	yes	yes	aggregate on species level		
Mentha longifolia	*Mentha longifolia*	(L.) Huds.	yes	yes	yes			

Mentha longifolia subsp. longifolia	*Mentha longifolia*	(L.) Huds.	yes	yes	yes	aggregate on species level		
Mentha piperita agg.	*Mentha piperita*	NA	yes	yes	no	aggregate on species level	*Mentha x piperita*	
Mentha pulegium	*Mentha pulegium*	L.	yes	yes	yes			
Mentha rotundifolia	*Mentha rotundifolia*	(L.) Huds.	yes	yes	no		*Mentha x rotundifolia*	
Mentha spicata	*Mentha spicata*	L.	yes	yes	yes			
Mentha spicata agg.	*Mentha spicata*	L.	yes	yes	yes	aggregate on species level		
Mentha spicata subsp. glabrata	*Mentha spicata*	L.	yes	yes	yes	aggregate on species level		
Mentha spicata subsp. spicata	*Mentha spicata*	L.	yes	yes	yes	aggregate on species level		
Mentha suaveolens	*Mentha suaveolens*	Ehrh.	yes	yes	yes			
Mentha verticillata	*Mentha verticillata*	L.	yes	yes	no		*Mentha x verticillata*	
Mentha verticillata agg.	*Mentha verticillata*	L.	yes	yes	no	aggregate on species level	*Mentha x verticillata*	
Mentha villosa	*Mentha villosa*	Huds.	yes	yes	no		*Mentha x villosa*	
Menyanthes trifoliata	*Menyanthes trifoliata*	L.	yes	yes	yes			
Mercurialis annua	*Mercurialis annua*	L.	yes	yes	yes			
Mercurialis perennis	*Mercurialis perennis*	L.	yes	yes	yes			
Mercurialis perennis agg.	*Mercurialis perennis*	L.	yes	yes	yes	aggregate on species level		

Mespilus germanica	*Mespilus germanica*	L.	yes	yes	yes			
Meum athamanticum	*Meum athamanticum*	Jacq.	yes	yes	yes			
Mibora minima	*Mibora minima*	(L.) Desv.	yes	yes	yes			
Milium effusum	*Milium effusum*	L.	yes	yes	yes			
Milium effusum subsp. effusum	*Milium effusum*	L.	yes	yes	yes	aggregate on species level		
Mimulus guttatus	*Mimulus guttatus*	DC.	yes	yes	no			
Mimulus moschatus	*Mimulus moschatus*	Douglas ex Lindl.	yes	yes	yes			*Erythranthe moschata*
Minuartia hybrida	*Minuartia hybrida*	(Vill.) Schischk.	yes	yes	no			
Minuartia hybrida subsp. hybrida	*Minuartia hybrida*	(Vill.) Schischk.	yes	yes	no	aggregate on species level		
Minuartia hybrida subsp. tenuifolia	*Minuartia hybrida*	(Vill.) Schischk.	yes	yes	no	aggregate on species level		
Minuartia hybrida subsp. Tenuifolia	*Minuartia hybrida*	(Vill.) Schischk.	yes	yes	no	aggregate on species level		
Minuartia hybrida subsp. vaillantiana	*Minuartia hybrida*	(Vill.) Schischk.	yes	yes	no	aggregate on species level		
Minuartia rubra	*Minuartia rubra*	(Sm.) Rchb.	yes	yes	no			
Misopates orontium	*Misopates orontium*	(L.) Raf.	yes	yes	yes			
Moehringia trinervia	*Moehringia trinervia*	(L.) Clairv.	yes	yes	yes			

Molinia arundinacea	*Molinia caerulea*	(L.) Moench	yes	yes	yes	taxonomic inconsistencies between first and second mapping		
Molinia caerulea	*Molinia caerulea*	(L.) Moench	yes	yes	yes			
Molinia caerulea agg.	*Molinia caerulea*	(L.) Moench	yes	yes	yes	aggregate on species level		
Moneses uniflora	*Moneses uniflora*	(L.) A. Gray	yes	yes	yes			
Monotropa hypophegea	*Monotropa hypophegea*	Wallr.	yes	yes	no			
Monotropa hypopitys	*Monotropa hypopitys*	L.	yes	yes	no			
Monotropa hypopitys agg.	*Monotropa hypopitys*	L.	yes	yes	no	aggregate on species level		
Montia fontana	*Montia fontana*	L.	yes	yes	yes			
Montia fontana subsp. amporitana	*Montia fontana*	L.	yes	yes	yes	aggregate on species level		
Montia fontana subsp. chondrosperma	*Montia fontana*	L.	yes	yes	yes	aggregate on species level		
Montia fontana subsp. fontana	*Montia fontana*	L.	yes	yes	yes	aggregate on species level		
Montia fontana subsp. variabilis	*Montia fontana*	L.	yes	yes	yes	aggregate on species level		
Muscari armeniacum	*Muscari armeniacum*	Baker	yes	yes	no			
Muscari botryoides	*Muscari botryoides*	(L.) Mill.	yes	yes	yes			

Muscari comosum	*Muscari comosum*	(L.) Mill.	yes	yes	yes			*Leopoldia comosa*
Muscari neglectum	*Muscari neglectum*	Guss. ex Ten.	yes	yes	no			
Myagrum perfoliatum	*Myagrum perfoliatum*	L.	yes	yes	yes			
Mycelis muralis	*Mycelis muralis*	(L.) Dumort.	yes	yes	yes			*Lactuca muralis*
Myosotis arvensis	*Myosotis arvensis*	(L.) Hill	yes	yes	yes			
Myosotis arvensis subsp. arvensis	*Myosotis arvensis*	(L.) Hill	yes	yes	yes	aggregate on species level		
Myosotis arvensis subsp. umbrata	*Myosotis arvensis*	(L.) Hill	yes	yes	yes	aggregate on species level		
Myosotis discolor	*Myosotis discolor*	Pers.	yes	yes	yes			
Myosotis laxa	*Myosotis laxa*	Lehm.	yes	yes	yes			
Myosotis nemorosa	*Myosotis scorpioides*	L.	yes	yes	yes	taxonomic inconsistencies between first and second mapping		
Myosotis ramosissima	*Myosotis ramosissima*	Rochel ex Schult.	yes	yes	yes			
Myosotis rehsteineri	*Myosotis rehsteineri*	Wartm.	yes	yes	no			
Myosotis scorpioides	*Myosotis scorpioides*	L.	yes	yes	yes			
Myosotis scorpioides agg.	*Myosotis scorpioides*	L.	yes	yes	yes	aggregate on species level		

Myosotis scorpioides subsp. scorpioides	*Myosotis scorpioides*	L.	yes	yes	yes	aggregate on species level		
Myosotis stricta	*Myosotis stricta*	Link ex Roem. & Schult.	yes	yes	yes			
Myosotis sylvatica	*Myosotis sylvatica*	Ehrh. ex Hoffm.	yes	yes	yes			
Myosotis sylvatica agg.	*Myosotis sylvatica*	Ehrh. ex Hoffm.	yes	yes	yes	aggregate on species level		
Myosurus minimus	*Myosurus minimus*	L.	yes	yes	yes			
Myricaria germanica	*Myricaria germanica*	(L.) Desv.	yes	yes	no			
Myriophyllum spicatum	*Myriophyllum spicatum*	L.	yes	yes	no			
Myriophyllum verticillatum	*Myriophyllum verticillatum*	L.	yes	yes	no			
Najas marina	*Najas marina*	L.	yes	yes	no			
Najas marina subsp. intermedia	*Najas marina*	Wolfg. ex Gorski	yes	yes	no	aggregate on species level		
Najas marina subsp. marina	*Najas marina*	L.	yes	yes	no	aggregate on species level		
Najas minor	*Najas minor*	All.	yes	yes	no			
Narcissus poeticus agg.	*Narcissus poeticus*	L.	yes	yes	no	aggregate on species level		
Nardus stricta	*Nardus stricta*	L.	yes	yes	yes			
Nasturtium microphyllum	*Nasturtium microphyllum*	Boenn. ex Rchb.	yes	yes	yes			
Nasturtium officinale	*Nasturtium officinale*	R. Br.	yes	yes	yes			
Nasturtium officinale agg.	*Nasturtium officinale*	R. Br.	yes	yes	yes	aggregate on species level		
Neottia nidus-avis	*Neottia nidus-avis*	(L.) Rich.	yes	yes	no			
Nepeta cataria	*Nepeta cataria*	L.	yes	yes	yes			
Nepeta racemosa	*Nepeta racemosa*	Lam.	yes	yes	no			

Neslia paniculata	*Neslia paniculata*	(L.) Desv.	yes	yes	no			
Neslia paniculata subsp. paniculata	*Neslia paniculata*	(L.) Desv.	yes	yes	no	aggregate on species level		
Nicandra physalodes	*Nicandra physalodes*	(L.) P. Gaertn.	yes	yes	yes			
Nigella damascena	*Nigella damascena*	L.	yes	yes	yes			
Nonea pulla	*Nonea pulla*	(L.) DC.	yes	yes	yes			
Nuphar lutea	*Nuphar lutea*	(L.) Sibth. & Sm.	yes	yes	no			
Nuphar pumila	*Nuphar pumila*	(Timm) DC.	yes	yes	no			
Nymphaea alba	*Nymphaea alba*	L.	yes	yes	no			
Nymphaea alba var. rosea	*Nymphaea alba*	L.	yes	yes	no	aggregate on species level		
Nymphaea candida	*Nymphaea candida*	C. Presl	yes	yes	no			
Nymphoides peltata	*Nymphoides peltata*	(S. G. Gmel.) Kuntze	yes	yes	no			
Odontites luteus	*Odontites luteus*	(L.) Clairv.	yes	yes	yes			
Odontites vernus	*Odontites vernus*	(Bellardi) Dumort.	yes	yes	no			
Odontites vernus agg.	*Odontites vernus*	(Bellardi) Dumort.	yes	yes	no	aggregate on species level		
Odontites vulgaris	*Odontites vulgaris*	Moench	yes	yes	no			
Oenanthe aquatica	*Oenanthe aquatica*	(L.) Poir.	yes	yes	yes			
Oenanthe aquatica agg.	*Oenanthe aquatica*	(L.) Poir.	yes	yes	yes	aggregate on species level		
Oenanthe fistulosa	*Oenanthe fistulosa*	L.	yes	yes	yes			
Oenanthe lachenalii	*Oenanthe lachenalii*	C. C. Gmel.	yes	yes	yes			
Oenothera biennis	*Oenothera biennis*	L.	yes	yes	yes			

Oenothera glazioviana	*Oenothera biennis*	L.	yes	yes	yes	taxonomic inconsistencies between first and second mapping			
Omphalodes verna	*Omphalodes verna*	Moench	yes	yes	no				
Onobrychis arenaria	*Onobrychis arenaria*	(Kit.) DC.	yes	yes	yes				
Onobrychis arenaria subsp. arenaria	*Onobrychis arenaria*	(Kit.) DC.	yes	yes	yes	aggregate on species level			
Onobrychis montana	*Onobrychis montana*	DC.	yes	yes	yes				
Onobrychis viciifolia	*Onobrychis viciifolia*	Scop.	yes	yes	yes				
Ononis repens	*Ononis repens*	L.	yes	yes	yes				*Ononis spinosa*
Ononis repens subsp. procurrens	*Ononis repens*	(Wallr.) Asch. & Graebn.	yes	yes	yes	aggregate on species level			
Ononis repens subsp. repens	*Ononis repens*	L.	yes	yes	yes	aggregate on species level			
Ononis spinosa	*Ononis spinosa*	L.	yes	yes	yes				
Ononis spinosa agg.	*Ononis spinosa*	L.	yes	yes	yes	aggregate on species level			
Ononis spinosa subsp. austriaca	*Ononis spinosa*	L.	yes	yes	yes	aggregate on species level			
Ononis spinosa subsp. spinosa	*Ononis spinosa*	L.	yes	yes	yes	aggregate on species level			
Onopordum acanthium	*Onopordum acanthium*	L.	yes	yes	yes				

Ophioglossum vulgatum	*Ophioglossum vulgatum*	L.	yes	yes	no			
Ophioglossum vulgatum agg.	*Ophioglossum vulgatum*	L.	yes	yes	no	aggregate on species level		
Ophrys apifera	*Ophrys apifera*	Huds.	yes	yes	yes			
Ophrys apifera subsp. apifera	*Ophrys apifera*	Huds.	yes	yes	yes	aggregate on species level		
Ophrys apifera subsp. friburgensis	*Ophrys apifera*	Huds.	yes	yes	yes	aggregate on species level		
Ophrys apifera var. aurita	*Ophrys apifera*	Huds.	yes	yes	yes	aggregate on species level		
Ophrys araneola	*Ophrys araneola*	Rchb.	yes	yes	no			
Ophrys holoserica	*Ophrys holoserica*	(Burm.f.) Greuter	yes	yes	no			
Ophrys holoserica subsp. holoserica	*Ophrys holoserica*	(Burm.f.) Greuter	yes	yes	no	aggregate on species level		
Ophrys insectifera	*Ophrys insectifera*	L.	yes	yes	no			
Ophrys sphegodes	*Ophrys sphegodes*	Mill.	yes	yes	no			
Ophrys sphegodes agg.	*Ophrys sphegodes*	Mill.	yes	yes	no	aggregate on species level		
Orchis coriophora	*Orchis coriophora*	L.	yes	yes	no			
Orchis mascula	*Orchis mascula*	(L.) L.	yes	yes	yes			
Orchis mascula subsp. mascula	*Orchis mascula*	(L.) L.	yes	yes	yes	aggregate on species level		
Orchis militaris	*Orchis militaris*	L.	yes	yes	yes			
Orchis morio	*Orchis morio*	L.	yes	yes	yes			*Anacamptis morio*
Orchis morio subsp. morio	*Orchis morio*	L.	yes	yes	yes	aggregate on species level		

Orchis pallens	*Orchis pallens*	L.	yes	yes	no			
Orchis palustris	*Orchis palustris*	Jacq.	yes	yes	yes			*Anacamptis palustris*
Orchis palustris var. palustris	*Orchis palustris*	Jacq.	yes	yes	yes	aggregate on species level		
Orchis purpurea	*Orchis purpurea*	Huds.	yes	yes	yes			
Orchis simia	*Orchis simia*	Lam.	yes	yes	no			
Orchis simia var. albiflora	*Orchis simia*	Lam.	yes	yes	no	aggregate on species level		
Orchis ustulata	*Orchis ustulata*	L.	yes	yes	no			
Orchis ustulata subsp. aestivalis	*Orchis ustulata*	L.	yes	yes	no	aggregate on species level		
Oreopteris limbosperma	*Oreopteris limbosperma*	(Bellardi ex All.) H. P. Fuchs	yes	yes	no		*Lastrea limbosperma*	
Origanum vulgare	*Origanum vulgare*	L.	yes	yes	yes			
Origanum vulgare agg.	*Origanum vulgare*	L.	yes	yes	yes	aggregate on species level		
Origanum vulgare subsp. prismaticum	*Origanum vulgare*	L.	yes	yes	yes	aggregate on species level		
Origanum vulgare subsp. vulgare	*Origanum vulgare*	L.	yes	yes	yes	aggregate on species level		
Origanum vulgare var. prismaticum	*Origanum vulgare*	L.	yes	yes	yes	aggregate on species level		
Orlaya grandiflora	*Orlaya grandiflora*	(L.) Hoffm.	yes	yes	yes			
Ornithogalum boucheanum	*Ornithogalum boucheanum*	(Kunth) Asch.	yes	yes	no			
Ornithogalum nutans	*Ornithogalum nutans*	L.	yes	yes	no			

Ornithogalum nutans agg.	*Ornithogalum nutans*	L.	yes	yes	no	aggregate on species level		
Ornithogalum pyramidale	*Ornithogalum pyramidale*	L.	yes	yes	no			
Ornithogalum pyrenaicum	*Ornithogalum pyrenaicum*	L.	yes	yes	no			
Ornithogalum umbellatum	*Ornithogalum umbellatum*	L.	yes	yes	yes			
Ornithogalum umbellatum agg.	*Ornithogalum umbellatum*	L.	yes	yes	yes	aggregate on species level		
Ornithopus perpusillus	*Ornithopus perpusillus*	L.	yes	yes	yes			
Ornithopus sativus	*Ornithopus sativus*	Brot.	yes	yes	no			
Orobanche alba	*Orobanche alba*	Stephan ex Willd.	yes	yes	no			
Orobanche alsatica	*Orobanche alsatica*	Kirschl.	yes	yes	no			
Orobanche alsatica subsp. alsatica	*Orobanche alsatica*	Kirschl.	yes	yes	no	aggregate on species level		
Orobanche alsatica subsp. libanotidis	*Orobanche alsatica*	Kirschl.	yes	yes	no	aggregate on species level		
Orobanche alsatica subsp. mayeri	*Orobanche alsatica*	Kirschl.	yes	yes	no	aggregate on species level		
Orobanche amethystea	*Orobanche amethystea*	Thuill.	yes	yes	no			
Orobanche arenaria	*Orobanche arenaria*	Borkh.	yes	yes	no			
Orobanche caryophyllacea	*Orobanche caryophyllacea*	Sm.	yes	yes	no			
Orobanche elatior	*Orobanche elatior*	Sutton	yes	yes	no			
Orobanche gracilis	*Orobanche gracilis*	Sm.	yes	yes	no			
Orobanche hederae	*Orobanche hederae*	Vaucher ex Duby	yes	yes	no			
Orobanche lucorum	*Orobanche lucorum*	F. W. Schultz	yes	yes	no			
Orobanche lutea	*Orobanche lutea*	Baumg.	yes	yes	no			

Orobanche lutea lus. violacea	*Orobanche lutea*	Baumg.	yes	yes	no	aggregate on species level		
Orobanche minor	*Orobanche minor*	Sm.	yes	yes	no			
Orobanche picridis	*Orobanche picridis*	F. W. Schultz	yes	yes	no			
Orobanche purpurea	*Orobanche purpurea*	Jacq.	yes	yes	no			
Orobanche ramosa	*Orobanche ramosa*	L.	yes	yes	no			
Orobanche rapum-genistae	*Orobanche rapum-genistae*	Thuill.	yes	yes	no			
Orobanche reticulata	*Orobanche reticulata*	Wallr.	yes	yes	no			
Orobanche reticulata subsp. pallidiflora	*Orobanche reticulata*	Wallr.	yes	yes	no	aggregate on species level		
Orobanche reticulata subsp. reticulata	*Orobanche reticulata*	Wallr.	yes	yes	no	aggregate on species level		
Orobanche teucrii	*Orobanche teucrii*	Holandre	yes	yes	no			
Orthilia secunda	*Orthilia secunda*	(L.) House	yes	yes	yes			
Osmunda regalis	*Osmunda regalis*	L.	yes	yes	yes			
Oxalis acetosella	*Oxalis acetosella*	L.	yes	yes	yes			
Oxalis corniculata	*Oxalis corniculata*	L.	yes	yes	yes			
Oxalis dillenii	*Oxalis dillenii*	Jacq.	yes	yes	yes			
Oxalis stricta	*Oxalis stricta*	Bunge	yes	yes	no		*Oxalis fontana*	
Oxytropis pilosa	*Oxytropis pilosa*	(L.) DC.	yes	yes	no			
Panicum capillare	*Panicum capillare*	L.	yes	yes	yes			
Panicum miliaceum	*Panicum miliaceum*	L.	yes	yes	no			
Panicum miliaceum subsp. agricola	*Panicum miliaceum*	L.	yes	yes	no	aggregate on species level		

Panicum miliaceum subsp. miliaceum	*Panicum miliaceum*	L.	yes	yes	no	aggregate on species level		
Panicum miliaceum subsp. ruderale	*Panicum miliaceum*	L.	yes	yes	no	aggregate on species level		
Papaver argemone	*Papaver argemone*	L.	yes	yes	yes			
Papaver dubium	*Papaver dubium*	L.	yes	yes	yes			
Papaver dubium subsp. confine	*Papaver dubium*	L.	yes	yes	yes	aggregate on species level		
Papaver dubium subsp. dubium	*Papaver dubium*	L.	yes	yes	yes	aggregate on species level		
Papaver dubium subsp. lecoqii	*Papaver dubium*	L.	yes	yes	yes	aggregate on species level		
Papaver rhoeas	*Papaver rhoeas*	L.	yes	yes	yes			
Parietaria judaica	*Parietaria judaica*	L.	yes	yes	yes			
Parietaria officinalis	*Parietaria officinalis*	L.	yes	yes	yes			
Paris quadrifolia	*Paris quadrifolia*	L.	yes	yes	yes			
Parnassia palustris	*Parnassia palustris*	L.	yes	yes	yes			
Parthenocissus inserta	*Parthenocissus inserta*	(Kern.) Fritsch	yes	yes	yes			
Parthenocissus quinquefolia	*Parthenocissus quinquefolia*	(L.) Planch.	yes	yes	yes			
Parthenocissus quinquefolia agg.	*Parthenocissus quinquefolia*	(L.) Planch.	yes	yes	yes	aggregate on species level		
Pastinaca sativa	*Pastinaca sativa*	L.	yes	yes	yes			
Pastinaca sativa subsp. sativa	*Pastinaca sativa*	L.	yes	yes	yes	aggregate on species level		
Pastinaca sativa subsp. sylvestris	*Pastinaca sativa*	L.	yes	yes	yes	aggregate on species level		

Pastinaca sativa subsp. urens	*Pastinaca sativa*	L.	yes	yes	yes	aggregate on species level		
Pastinaca sativa var. sativa	*Pastinaca sativa*	L.	yes	yes	yes	aggregate on species level		
Paulownia tomentosa	*Paulownia tomentosa*	(Thunb.) Steud.	yes	yes	no			
Pedicularis foliosa	*Pedicularis foliosa*	L.	yes	yes	no			
Pedicularis palustris	*Pedicularis palustris*	L.	yes	yes	yes			
Pedicularis palustris subsp. palustris	*Pedicularis palustris*	L.	yes	yes	yes	aggregate on species level		
Pedicularis sceptrum-carolinum	*Pedicularis sceptrum-carolinum*	L.	yes	yes	yes			
Pedicularis sylvatica	*Pedicularis sylvatica*	L.	yes	yes	yes			
Peplis portula	*Peplis portula*	L.	yes	yes	yes			*Ludwigia palustris*
Persicaria amphibia	*Persicaria amphibia*	L.	yes	yes	yes			
Persicaria dubia	*Persicaria dubia*	Schrank	yes	yes	no			
Persicaria hydropiper	*Persicaria hydropiper*	L.	yes	yes	yes			
Persicaria lapathifolia	*Persicaria lapathifolia*	(L.) Delarbre	yes	yes	yes			
Persicaria lapathifolia subsp. brittingeri	*Persicaria lapathifolia*	(L.) Delarbre	yes	yes	yes	aggregate on species level		
Persicaria lapathifolia subsp. lapathifolia	*Persicaria lapathifolia*	(L.) Delarbre	yes	yes	yes	aggregate on species level		
Persicaria lapathifolia subsp. mesomorpha	*Persicaria lapathifolia*	(L.) Delarbre	yes	yes	yes	aggregate on species level		
Persicaria lapathifolia subsp. pallida	*Persicaria lapathifolia*	(L.) Delarbre	yes	yes	yes	aggregate on species level		

Persicaria maculosa	*Persicaria maculosa*	L.	yes	yes	yes			
Persicaria minor	*Persicaria minor*	Huds.	yes	yes	no			
Petasites albus	*Petasites albus*	(L.) P. Gaertn.	yes	yes	yes			
Petasites hybridus	*Petasites hybridus*	(L.) P. Gaertn.. B. Mey. & Scherb.	yes	yes	yes			
Petrorhagia prolifera	*Petrorhagia prolifera*	(L.) P. W. Ball & Heywood	yes	yes	yes			
Petrorhagia saxifraga	*Petrorhagia saxifraga*	(L.) Link	yes	yes	no			
Peucedanum alsaticum	*Peucedanum alsaticum*	L.	yes	yes	no			
Peucedanum cervaria	*Peucedanum cervaria*	(L.) Lapeyr.	yes	yes	yes			*Cervaria rivini*
Peucedanum officinale	*Peucedanum officinale*	L.	yes	yes	yes			*Peucedanum officinale*
Peucedanum oreoselinum	*Peucedanum oreoselinum*	(L.) Moench	yes	yes	yes			
Peucedanum palustre	*Peucedanum palustre*	(L.) Moench	yes	yes	yes			
Phacelia tanacetifolia	*Phacelia tanacetifolia*	Benth.	yes	yes	yes			
Phalaris arundinacea	*Phalaris arundinacea*	L.	yes	yes	yes			
Phalaris arundinacea var. picta	*Phalaris arundinacea*	L.	yes	yes	yes	aggregate on species level		
Phegopteris connectilis	*Phegopteris connectilis*	(L.) Sloss.	yes	yes	no			
Phleum bertolonii	*Phleum pratense*	L.	yes	yes	no	taxonomic inconsistencies between first and second mapping		

Phleum phleoides	*Phleum phleoides*	(L.) H. Karst.	yes	yes	yes			
Phleum pratense	*Phleum pratense*	L.	yes	yes	yes			
Phleum pratense agg.	*Phleum pratense*	L.	yes	yes	yes	aggregate on species level		
Phragmites australis	*Phragmites australis*	(Cav.) Trin. ex Steud.	yes	yes	yes			
Phragmites australis var. australis	*Phragmites australis*	(Cav.) Trin. ex Steud.	yes	yes	yes	aggregate on species level		
Phyteuma nigrum	*Phyteuma nigrum*	F. W. Schmidt	yes	yes	yes			
Phyteuma orbiculare	*Phyteuma orbiculare*	L.	yes	yes	yes			
Phyteuma orbiculare subsp. orbiculare	*Phyteuma orbiculare*	L.	yes	yes	yes	aggregate on species level		
Phyteuma spicatum agg.	*Phyteuma spicatum*	L.	yes	yes	no	aggregate on species level		
Phyteuma spicatum subsp. occidentale	*Phyteuma spicatum*	L.	yes	yes	no	aggregate on species level		
Phyteuma spicatum subsp. spicatum	*Phyteuma spicatum*	L.	yes	yes	no	aggregate on species level		
Phytolacca americana	*Phytolacca americana*	L.	yes	yes	no			
Phytolacca esculenta	*Phytolacca esculenta*	Van Houtte	yes	yes	no			
Picea abies	*Picea abies*	(L.) H. Karst.	yes	yes	yes			
Picea abies subsp. abies	*Picea abies*	(L.) H. Karst.	yes	yes	yes	aggregate on species level		
Picris echioides	*Picris echioides*	L.	yes	yes	yes			*Helminthotheca echioides*

Picris hieracioides	*Picris hieracioides*	L.	yes	yes	yes			
Picris hieracioides subsp. grandiflora	*Picris hieracioides*	L.	yes	yes	yes	aggregate on species level		
Picris hieracioides subsp. hieracioides	*Picris hieracioides*	L.	yes	yes	yes	aggregate on species level		
Picris hieracioides subsp. spinulosa	*Picris hieracioides*	L.	yes	yes	yes	aggregate on species level		
Picris hieracioides subsp. villarsii	*Picris hieracioides*	L.	yes	yes	yes	aggregate on species level		
Pilularia globulifera	*Pilularia globulifera*	L.	yes	yes	no			
Pimpinella major	*Pimpinella major*	(L.) Huds.	yes	yes	yes			
Pimpinella major subsp. major	*Pimpinella major*	(L.) Huds.	yes	yes	yes	aggregate on species level		
Pimpinella major subsp. rubra	*Pimpinella major*	(Hoppe) O. Schwarz	yes	yes	yes	aggregate on species level		
Pimpinella nigra	*Pimpinella nigra*	Mill.	yes	yes	no			
Pimpinella peregrina	*Pimpinella peregrina*	L.	yes	yes	no			
Pimpinella saxifraga	*Pimpinella saxifraga*	L.	yes	yes	yes			
Pimpinella saxifraga agg.	*Pimpinella saxifraga*	L.	yes	yes	yes	aggregate on species level		
Pimpinella saxifraga subsp. minor	*Pimpinella saxifraga*	L.	yes	yes	yes	aggregate on species level		
Pimpinella saxifraga subsp. saxifraga	*Pimpinella saxifraga*	L.	yes	yes	yes	aggregate on species level		

Pimpinella saxifraga var. hircina	*Pimpinella saxifraga*	L.	yes	yes	yes	aggregate on species level		
Pinguicula alpina	*Pinguicula alpina*	L.	yes	yes	yes			
Pinguicula vulgaris	*Pinguicula vulgaris*	L.	yes	yes	yes			
Pinus mugo	*Pinus mugo*	Turra	yes	yes	no			
Pinus mugo agg.	*Pinus mugo*	Turra	yes	yes	no	aggregate on species level		
Pinus mugo subsp. rotundata	*Pinus mugo*	Turra	yes	yes	no	aggregate on species level		
Pinus mugo subsp. uncinata	*Pinus mugo*	Turra	yes	yes	no	aggregate on species level		
Pinus nigra	*Pinus nigra*	J. F. Arnold	yes	yes	yes			
Pinus nigra subsp. nigra	*Pinus nigra*	J. F. Arnold	yes	yes	yes	aggregate on species level		
Pinus sylvestris	*Pinus sylvestris*	L.	yes	yes	yes			
Pinus sylvestris subsp. sylvestris	*Pinus sylvestris*	L.	yes	yes	yes	aggregate on species level		
Plantago coronopus	*Plantago coronopus*	L.	yes	yes	yes			
Plantago coronopus subsp. commutatus	*Plantago coronopus*	L.	yes	yes	yes	aggregate on species level		
Plantago lanceolata	*Plantago lanceolata*	L.	yes	yes	yes			
Plantago lanceolata subsp. lanceolata	*Plantago lanceolata*	L.	yes	yes	yes	aggregate on species level		
Plantago lanceolata subsp. sphaerostachya	*Plantago lanceolata*	L.	yes	yes	yes	aggregate on species level		
Plantago major	*Plantago major*	L.	yes	yes	yes			

Plantago major subsp. intermedia	*Plantago major*	L.	yes	yes	yes	aggregate on species level		
Plantago major subsp. major	*Plantago major*	L.	yes	yes	yes	aggregate on species level		
Plantago major subsp. winteri	*Plantago major*	L.	yes	yes	yes	aggregate on species level		
Plantago major var. maxima	*Plantago major*	L.	yes	yes	yes	aggregate on species level		
Plantago maritima subsp. maritima	*Plantago maritima*	L.	yes	no	no	aggregate on species level		
Plantago media	*Plantago media*	L.	yes	yes	yes			
Plantago media agg.	*Plantago media*	L.	yes	yes	yes	aggregate on species level		
Plantago media subsp. media	*Plantago media*	L.	yes	yes	yes	aggregate on species level		
Platanthera bifolia	*Platanthera bifolia*	(L.) Rich.	yes	yes	yes			
Platanthera bifolia subsp. bifolia	*Platanthera bifolia*	(L.) Rich.	yes	yes	yes	aggregate on species level		
Platanthera chlorantha	*Platanthera chlorantha*	(Custer) Rchb.	yes	yes	yes			
Pleurospermum austriacum	*Pleurospermum austriacum*	(L.) Hoffm.	yes	yes	no			
Poa alpina	*Poa alpina*	L.	yes	yes	yes			
Poa angustifolia	*Poa angustifolia*	L.	yes	yes	yes			
Poa annua	*Poa annua*	L.	yes	yes	yes			
Poa annua agg.	*Poa annua*	L.	yes	yes	yes	aggregate on species level		
Poa bulbosa	*Poa bulbosa*	L.	yes	yes	yes			

Poa bulbosa subsp. bulbosa	*Poa bulbosa*	L.	yes	yes	yes	aggregate on species level		
Poa chaixii	*Poa chaixii*	Vill.	yes	yes	yes			
Poa compressa	*Poa compressa*	L.	yes	yes	yes			
Poa humilis	*Poa humilis*	Ehrh. ex Hoffm.	yes	yes	no			
Poa nemoralis	*Poa nemoralis*	L.	yes	yes	yes			
Poa nemoralis agg.	*Poa nemoralis*	L.	yes	yes	yes	aggregate on species level		
Poa palustris	*Poa palustris*	L.	yes	yes	yes			
Poa pratensis	*Poa pratensis*	L.	yes	yes	yes			
Poa pratensis agg.	*Poa pratensis*	L.	yes	yes	yes	aggregate on species level		
Poa pratensis subsp. pratensis	*Poa pratensis*	L.	yes	yes	yes	aggregate on species level		
Poa remota	*Poa remota*	Forselles	yes	yes	no			
Poa supina	*Poa supina*	Schrad.	yes	yes	no			
Poa trivialis	*Poa trivialis*	L.	yes	yes	no			
Poa trivialis subsp. trivialis	*Poa trivialis*	L.	yes	yes	no	aggregate on species level		
Polemonium caeruleum	*Polemonium caeruleum*	L.	yes	yes	yes			
Polycarpon tetraphyllum	*Polycarpon tetraphyllum*	(L.) L.	yes	yes	no			
Polygala amara subsp. brachyptera	*Polygala amara*	(Chodat) Hayek	yes	yes	no	aggregate on species level		
Polygala amarella	*Polygala amarella*	Crantz	yes	yes	yes			
Polygala calcarea	*Polygala calcarea*	F. W. Schultz	yes	yes	no			
Polygala chamaebuxus	*Polygala chamaebuxus*	L.	yes	yes	yes			
Polygala comosa	*Polygala comosa*	Schkuhr	yes	yes	yes			
Polygala serpyllifolia	*Polygala serpyllifolia*	Host	yes	yes	no			
Polygala vulgaris	*Polygala vulgaris*	L.	yes	yes	yes			

Polygala vulgaris subsp. oxyptera	*Polygala vulgaris*	L.	yes	yes	yes	aggregate on species level		
Polygala vulgaris subsp. vulgaris	*Polygala vulgaris*	L.	yes	yes	yes	aggregate on species level		
Polygonatum multiflorum	*Polygonatum multiflorum*	(L.) All.	yes	yes	yes			
Polygonatum odoratum	*Polygonatum odoratum*	(Mill.) Druce	yes	yes	yes			
Polygonatum verticillatum	*Polygonatum verticillatum*	(L.) All.	yes	yes	yes			
Polygonum arenastrum	*Polygonum aviculare*	L.	yes	yes	yes	taxonomic inconsistencies between first and second mapping		
Polygonum arenastrum subsp. arenastrum	*Polygonum aviculare*	L.	yes	yes	yes	taxonomic inconsistencies between first and second mapping		
Polygonum arenastrum subsp. calcatum	*Polygonum aviculare*	L.	yes	yes	yes	taxonomic inconsistencies between first and second mapping		

Polygonum arenastrum subsp. microspermum	*Polygonum aviculare*	L.	yes	yes	yes	taxonomic inconsistencies between first and second mapping		
Polygonum aviculare	*Polygonum aviculare*	L.	yes	yes	yes			
Polygonum aviculare agg.	*Polygonum aviculare*	L.	yes	yes	yes	aggregate on species level		
Polygonum aviculare subsp. aviculare	*Polygonum aviculare*	L.	yes	yes	yes	aggregate on species level		
Polygonum aviculare subsp. rectum	*Polygonum aviculare*	L.	yes	yes	yes	aggregate on species level		
Polygonum aviculare subsp. rurivagum	*Polygonum aviculare*	L.	yes	yes	yes	aggregate on species level		
Polypodium interjectum	*Polypodium interjectum*	Shivas	yes	yes	no			
Polypodium vulgare	*Polypodium vulgare*	L.	yes	yes	no			
Polypodium vulgare agg.	*Polypodium vulgare*	L.	yes	yes	no	aggregate on species level		
Polypodium vulgare subsp. prionodes	*Polypodium vulgare*	L.	yes	yes	no	aggregate on species level		
Polystichum aculeatum	*Polystichum aculeatum*	(L.) Roth	yes	yes	no			
Polystichum aculeatum agg.	*Polystichum aculeatum*	(L.) Roth	yes	yes	no	aggregate on species level		
Polystichum braunii	*Polystichum braunii*	(Spenn.) Fée	yes	yes	no			

Polystichum lonchitis	*Polystichum lonchitis*	(L.) Roth	yes	yes	no			
Polystichum setiferum	*Polystichum setiferum*	(Forssk.) T. Moore ex Woyn.	yes	yes	no			
Populus alba	*Populus alba*	L.	yes	yes	yes			
Populus nigra	*Populus nigra*	L.	yes	yes	yes			
Populus nigra subsp. nigra	*Populus nigra*	L.	yes	yes	yes	aggregate on species level		
Populus nigra var. italica	*Populus nigra*	L.	yes	yes	yes	aggregate on species level		
Populus nigra var. nigra	*Populus nigra*	L.	yes	yes	yes	aggregate on species level		
Populus tremula	*Populus tremula*	L.	yes	yes	yes			
Populus trichocarpa	*Populus trichocarpa*	Hook.	yes	yes	no			
Portulaca oleracea	*Portulaca oleracea*	L.	yes	yes	yes			
Portulaca oleracea subsp. oleracea	*Portulaca oleracea*	L.	yes	yes	yes	aggregate on species level		
Portulaca oleracea subsp. sativa	*Portulaca oleracea*	L.	yes	yes	yes	aggregate on species level		
Potamogeton berchtoldii	*Potamogeton berchtoldii*	Fieber	yes	yes	no			
Potamogeton crispus	*Potamogeton crispus*	L.	yes	yes	no			
Potamogeton gramineus	*Potamogeton gramineus*	L.	yes	yes	no			
Potamogeton helveticus	*Potamogeton pectinatus*	(G. Fisch) W. Koch	yes	yes	yes	Buttler & Hand (2023)		*Stuckenia pectinata*
Potamogeton lucens	*Potamogeton lucens*	L.	yes	yes	no			
Potamogeton natans	*Potamogeton natans*	L.	yes	yes	no			
Potamogeton nodosus	*Potamogeton nodosus*	Poir.	yes	yes	no			

Potamogeton obtusifolius	*Potamogeton obtusifolius*	Mert. & W. D. J. Koch	yes	yes	no			
Potamogeton pectinatus	*Potamogeton pectinatus*	L.	yes	yes	yes			*Stuckenia pectinata*
Potamogeton pectinatus agg.	*Potamogeton pectinatus*	L.	yes	yes	yes	aggregate on species level		
Potamogeton pectinatus subsp. pectinatus	*Potamogeton pectinatus*	L.	yes	yes	yes	aggregate on species level		
Potamogeton perfoliatus	*Potamogeton perfoliatus*	L.	yes	yes	no			
Potamogeton pusillus	*Potamogeton pusillus*	L.	yes	yes	no			
Potamogeton pusillus agg.	*Potamogeton pusillus*	L.	yes	yes	no	aggregate on species level		
Potamogeton trichoides	*Potamogeton trichoides*	Cham. & Schltdl.	yes	yes	no			
Potentilla alba	*Potentilla alba*	L.	yes	yes	yes			
Potentilla anglica	*Potentilla anglica*	Laichard.	yes	yes	yes			
Potentilla anglica agg.	*Potentilla anglica*	Laichard.	yes	yes	yes	aggregate on species level		
Potentilla anserina	*Potentilla anserina*	L.	yes	yes	yes			
Potentilla arenaria	*Potentilla incana*	NA	yes	no	no	Buttler & Hand (2023)		
Potentilla argentea	*Potentilla argentea*	L.	yes	yes	yes			
Potentilla argentea agg.	*Potentilla argentea*	L.	yes	yes	yes	aggregate on species level		
Potentilla aurea	*Potentilla aurea*	L.	yes	yes	yes			
Potentilla cinerea	*Potentilla incana*	Vill.	yes	no	no	Buttler & Hand (2023)		
Potentilla cinerea subsp. incana	*Potentilla incana*	Vill.	yes	no	no	Buttler & Hand (2023)		

Potentilla collina	*Potentilla collina*	Wibel	yes	yes	no			
Potentilla collina agg.	*Potentilla collina*	Wibel	yes	yes	no	aggregate on species level		
Potentilla erecta	*Potentilla erecta*	(L.) Raeusch.	yes	yes	yes			
Potentilla heptaphylla	*Potentilla heptaphylla*	L.	yes	yes	yes			
Potentilla heptaphylla agg.	*Potentilla heptaphylla*	L.	yes	yes	yes	aggregate on species level		
Potentilla incana	*Potentilla incana*	P. Gaertn.. B. Mey. & Scherb.	yes	yes	yes			
Potentilla inclinata	*Potentilla inclinata*	Vill.	yes	yes	yes			
Potentilla intermedia	*Potentilla intermedia*	L.	yes	yes	yes			
Potentilla micrantha	*Potentilla micrantha*	Ramond ex DC.	yes	yes	yes			
Potentilla neglecta	*Potentilla argentea*	Baumg.	yes	yes	yes	POWO (2023)		
Potentilla neumanniana	*Potentilla tabernaemontani*	NA	yes	yes	yes	Sebald et al. (1992)		
Potentilla norvegica	*Potentilla norvegica*	L.	yes	yes	yes			
Potentilla palustris	*Potentilla palustris*	(L.) Scop.	yes	yes	yes			*Comarum palustre*
Potentilla recta	*Potentilla recta*	L.	yes	yes	yes			
Potentilla recta agg.	*Potentilla recta*	L.	yes	yes	yes	aggregate on species level		
Potentilla reptans	*Potentilla reptans*	L.	yes	yes	yes			
Potentilla rupestris	*Potentilla rupestris*	L.	yes	yes	no			
Potentilla sterilis	*Potentilla sterilis*	(L.) Garcke	yes	yes	yes			
Potentilla supina	*Potentilla supina*	L.	yes	yes	no			

Potentilla tabernaemontani	*Potentilla tabernaemontani*	Asch.	yes	yes	yes			*Potentilla neumanniana*
Potentilla verna	*Potentilla tabernaemontani*	Asch.	yes	yes	yes	Sebald et al. (1992)		
Potentilla verna agg.	*Potentilla tabernaemontani*	Asch.	yes	yes	yes	Sebald et al. (1992)		
Prenanthes purpurea	*Prenanthes purpurea*	L.	yes	yes	yes			
Primula auricula	*Primula auricula*	L.	yes	yes	yes			
Primula elatior	*Primula elatior*	(L.) Hill	yes	yes	yes			
Primula elatior agg.	*Primula elatior*	(L.) Hill	yes	yes	yes	aggregate on species level		
Primula farinosa	*Primula farinosa*	L.	yes	yes	yes			
Primula veris	*Primula veris*	L.	yes	yes	yes			
Primula veris subsp. canescens	*Primula veris*	L.	yes	yes	yes	aggregate on species level		
Primula veris subsp. columnae	*Primula veris*	L.	yes	yes	yes	aggregate on species level		
Primula veris subsp. veris	*Primula veris*	L.	yes	yes	yes	aggregate on species level		
Primula veris subsp. veris var. canescens	*Primula veris*	L.	yes	yes	yes	aggregate on species level		
Primula vulgaris	*Primula vulgaris*	Huds.	yes	yes	yes			*Primula acaulis*
Prunella grandiflora	*Prunella grandiflora*	(L.) Scholler	yes	yes	yes			
Prunella laciniata	*Prunella laciniata*	(L.) L.	yes	yes	no			
Prunella vulgaris	*Prunella vulgaris*	L.	yes	yes	yes			
Prunus avium	*Prunus avium*	L.	yes	yes	yes			
Prunus avium subsp. avium	*Prunus avium*	L.	yes	yes	yes	aggregate on species level		

Prunus avium subsp. juliana	*Prunus avium*	L.	yes	yes	yes	aggregate on species level		
Prunus cerasifera	*Prunus cerasifera*	Ehrh.	yes	yes	no			
Prunus cerasifera subsp. myrobalanus	*Prunus cerasifera*	Ehrh.	yes	yes	no	aggregate on species level		
Prunus cerasus agg.	*Prunus cerasus*	L.	yes	yes	no	aggregate on species level		
Prunus cerasus subsp. acida	*Prunus cerasus*	L.	yes	yes	no	aggregate on species level		
Prunus cerasus subsp. cerasus	*Prunus cerasus*	L.	yes	yes	no	aggregate on species level		
Prunus domestica subsp. domestica	*Prunus domestica*	L.	yes	yes	no	aggregate on species level		
Prunus domestica subsp. insititia	*Prunus domestica*	L.	yes	yes	no	aggregate on species level		
Prunus domestica subsp. syriaca	*Prunus domestica*	L.	yes	yes	no	aggregate on species level		
Prunus laurocerasus	*Prunus laurocerasus*	L.	yes	yes	no			
Prunus mahaleb	*Prunus mahaleb*	L.	yes	yes	yes			
Prunus padus	*Prunus padus*	L.	yes	yes	yes			
Prunus padus subsp. borealis	*Prunus padus*	L.	yes	yes	yes	aggregate on species level		
Prunus padus subsp. padus	*Prunus padus*	L.	yes	yes	yes	aggregate on species level		
Prunus padus subsp. petraea	*Prunus padus*	L.	yes	yes	yes	aggregate on species level		

Prunus serotina	*Prunus serotina*	Ehrh.	yes	yes	yes			
Prunus spinosa	*Prunus spinosa*	L.	yes	yes	yes			
Prunus spinosa agg.	*Prunus spinosa*	L.	yes	yes	yes	aggregate on species level		
Prunus spinosa subsp. fruticans	*Prunus spinosa*	L.	yes	yes	yes	aggregate on species level		
Pseudofumaria lutea	*Pseudofumaria lutea*	(L.) DC.	yes	yes	yes			
Pseudognaphalium luteoalbum	*Pseudognaphalium luteoalbum*	(L.) Hilliard & B. L. Burtt	yes	yes	yes			*Gnaphalium luteoalbum*
Pseudolysimachion longifolium	*Pseudolysimachion longifolium*	(L.) Opiz	yes	yes	yes			*Veronica longifolia*
Pseudolysimachion longifolium subsp. maritimum	*Pseudolysimachion longifolium*	(L.) Opiz	yes	yes	yes	aggregate on species level		
Pseudolysimachion spicatum	*Pseudolysimachion spicatum*	L.	yes	yes	yes			*Veronica spicata*
Pseudolysimachion spicatum subsp. spicatum	*Pseudolysimachion spicatum*	L.	yes	yes	yes	aggregate on species level		
Pseudorchis albida	*Pseudorchis albida*	(L.) Å. Loeve & D. Loeve	yes	yes	yes			
Pseudotsuga menziesii	*Pseudotsuga menziesii*	(Mirb.) Franco	yes	yes	yes			
Psyllium arenarium	*Psyllium arenarium*	L.	yes	no	yes			*Plantago arenaria*
Pteridium aquilinum	*Pteridium aquilinum*	(L.) Kuhn	yes	yes	yes			
Pteridium aquilinum subsp. aquilinum	*Pteridium aquilinum*	(L.) Kuhn	yes	yes	yes	aggregate on species level		
Pteridium aquilinum subsp. pinetorum	*Pteridium aquilinum*	(L.) Kuhn	yes	yes	yes	aggregate on species level		

Puccinellia distans	*Puccinellia distans*	(Jacq.) Parl.	yes	yes	no			
Puccinellia distans agg.	*Puccinellia distans*	(Jacq.) Parl.	yes	yes	no	aggregate on species level		
Pulicaria dysenterica	*Pulicaria dysenterica*	(L.) Bernh.	yes	yes	yes			
Pulicaria dysenterica subsp. dysenterica	*Pulicaria dysenterica*	(L.) Bernh.	yes	yes	yes	aggregate on species level		
Pulicaria vulgaris	*Pulicaria vulgaris*	Gaertn.	yes	yes	yes			
Pulmonaria collina	*Pulmonaria collina*	W. Sauer	yes	yes	no			
Pulmonaria mollis	*Pulmonaria mollis*	Wulfen ex Hornem.	yes	yes	no			
Pulmonaria mollis subsp. mollis	*Pulmonaria mollis*	Wulfen ex Hornem.	yes	yes	no	aggregate on species level		
Pulmonaria montana	*Pulmonaria montana*	Lej.	yes	yes	no			
Pulmonaria montana agg.	*Pulmonaria montana*	Lej.	yes	yes	no	aggregate on species level		
Pulmonaria montana subsp. montana	*Pulmonaria montana*	Lej.	yes	yes	no	aggregate on species level		
Pulmonaria obscura	*Pulmonaria obscura*	Dumort.	yes	yes	yes			
Pulmonaria officinalis	*Pulmonaria officinalis*	L.	yes	yes	yes			
Pulsatilla vulgaris	*Pulsatilla vulgaris*	Mill.	yes	yes	yes			
Pulsatilla vulgaris subsp. vulgaris	*Pulsatilla vulgaris*	Mill.	yes	yes	yes	aggregate on species level		
Pyrola chlorantha	*Pyrola chlorantha*	Sw.	yes	yes	yes			
Pyrola minor	*Pyrola minor*	L.	yes	yes	yes			
Pyrola rotundifolia	*Pyrola rotundifolia*	L.	yes	yes	yes			

Pyrola rotundifolia subsp. rotundifolia	*Pyrola rotundifolia*	L.	yes	yes	yes	aggregate on species level		
Pyrus communis	*Pyrus communis*	L.	yes	yes	yes			
Pyrus communis agg.	*Pyrus communis*	L.	yes	yes	yes	aggregate on species level		
Pyrus pyraster	*Pyrus pyraster*	Burgsd.	yes	yes	yes			*Pyrus communis*
Quercus cerris	*Quercus cerris*	L.	yes	yes	yes			
Quercus petraea	*Quercus petraea*	Liebl.	yes	yes	yes			
Quercus petraea agg.	*Quercus petraea*	Liebl.	yes	yes	yes	aggregate on species level		
Quercus pubescens	*Quercus pubescens*	Willd.	yes	yes	yes			
Quercus pubescens agg.	*Quercus pubescens*	Willd.	yes	yes	yes	aggregate on species level		
Quercus robur	*Quercus robur*	L.	yes	yes	yes			
Quercus rubra	*Quercus rubra*	L.	yes	yes	yes			
Radiola linoides	*Radiola linoides*	Roth	yes	yes	yes			
Ranunculus abstrusus	*Ranunculus auricomus*	O. Schwarz	yes	yes	no	Buttler & Hand (2023)		
Ranunculus aconitifolius	*Ranunculus aconitifolius*	L.	yes	yes	yes			
Ranunculus aconitifolius agg.	*Ranunculus aconitifolius*	L.	yes	yes	yes	aggregate on species level		
Ranunculus aconitifolius subsp. platanifolius	*Ranunculus platanifolius*	L.	yes	yes	yes	aggregate on species level		
Ranunculus acris	*Ranunculus acris*	L.	yes	yes	yes			
Ranunculus acris agg.	*Ranunculus acris*	L.	yes	yes	yes	aggregate on species level		

Ranunculus acris subsp. acris	*Ranunculus acris*	L.	yes	yes	yes	aggregate on species level		
Ranunculus acris subsp. friesianus	*Ranunculus acris*	L.	yes	yes	yes	aggregate on species level		
Ranunculus acris subsp. friesianus	*Ranunculus acris*	L.	yes	yes	yes	aggregate on species level		
Ranunculus aquatilis	*Ranunculus aquatilis*	L.	yes	yes	no			
Ranunculus aquatilis agg.	*Ranunculus aquatilis*	L.	yes	yes	no	aggregate on species level		
Ranunculus argoviensis	*Ranunculus auricomus*	W. Koch	yes	yes	no	Buttler & Hand (2023)		
Ranunculus arvensis	*Ranunculus arvensis*	L.	yes	yes	yes			
Ranunculus auricomus agg.	*Ranunculus auricomus*	L.	yes	yes	no	aggregate on species level		
Ranunculus bayerae	*Ranunculus auricomus*	L.	yes	yes	no	Müller et al. (2016) Rothmaler krit Bd.		
Ranunculus biformis	*Ranunculus auricomus*	L.	yes	yes	no	Buttler & Hand (2023)		
Ranunculus breyninus	*Ranunculus breyninus*	M. Bieb.	yes	yes	no			
Ranunculus bulbosus	*Ranunculus bulbosus*	L.	yes	yes	yes			
Ranunculus bulbosus subsp. bulbosus	*Ranunculus bulbosus*	L.	yes	yes	yes	aggregate on species level		
Ranunculus carinthiacus	*Ranunculus carinthiacus*	Hoppe	yes	yes	no			
Ranunculus cassubicifolius	*Ranunculus auricomus*	L.	yes	yes	no	Buttler & Hand (2023)		

Ranunculus circinatus	*Ranunculus circinatus*	Sibth.	yes	yes	no			
Ranunculus ficaria	*Ranunculus ficaria*	L.	yes	yes	yes			*Ficaria verna*
Ranunculus ficaria subsp. bulbilifer	*Ranunculus ficaria*	L.	yes	yes	yes	aggregate on species level		
Ranunculus flammula	*Ranunculus flammula*	L.	yes	yes	yes			
Ranunculus flammula agg.	*Ranunculus flammula*	L.	yes	yes	yes	aggregate on species level		
Ranunculus flammula subsp. flammula	*Ranunculus flammula*	L.	yes	yes	yes	aggregate on species level		
Ranunculus fluitans	*Ranunculus fluitans*	Lam.	yes	yes	no			
Ranunculus fluitans agg.	*Ranunculus fluitans*	Lam.	yes	yes	no	aggregate on species level		
Ranunculus fluitans var. heterophyllus	*Ranunculus fluitans*	Glueck	yes	yes	no	aggregate on species level		
Ranunculus haasii	*Ranunculus auricomus*	L.	yes	yes	no	Buttler & Hand (2023)		
Ranunculus hederaceus	*Ranunculus hederaceus*	L.	yes	yes	no			
Ranunculus hevellus	*Ranunculus auricomus*	L.	yes	yes	no	Buttler & Hand (2023)		
Ranunculus kochii	*Ranunculus auricomus*	L.	yes	yes	no	Buttler & Hand (2023)		
Ranunculus kunzii	*Ranunculus auricomus*	L.	yes	yes	no	Buttler & Hand (2023)		
Ranunculus lanuginosus	*Ranunculus lanuginosus*	L.	yes	yes	no			
Ranunculus leptomeris	*Ranunculus auricomus*	L.	yes	yes	no	Buttler & Hand (2023)		
Ranunculus lingua	*Ranunculus lingua*	L.	yes	yes	yes			

Ranunculus lucorum	*Ranunculus auricomus*	L.	yes	yes	no	Buttler & Hand (2023)		
Ranunculus lunaris	*Ranunculus auricomus*	L.	yes	yes	no	Buttler & Hand (2023)		
Ranunculus macrotis	*Ranunculus auricomus*	L.	yes	yes	no	Buttler & Hand (2023)		
Ranunculus megacarpus	*Ranunculus auricomus*	L.	yes	yes	no	Buttler & Hand (2023)		
Ranunculus mosbachensis	*Ranunculus auricomus*	L.	yes	yes	no	Buttler & Hand (2023)		
Ranunculus multisectus	*Ranunculus auricomus*	L.	yes	yes	no	Buttler & Hand (2023)		
Ranunculus nemorosus	*Ranunculus nemorosus*	DC.	yes	yes	no			
Ranunculus opimus	*Ranunculus auricomus*	L.	yes	yes	no	Buttler & Hand (2023)		
Ranunculus peltatus	*Ranunculus peltatus*	Schrank	yes	yes	no			
Ranunculus peltatus subsp. baudotii	*Ranunculus peltatus*	Schrank	yes	yes	no	aggregate on species level		
Ranunculus peltatus subsp. peltatus	*Ranunculus peltatus*	Schrank	yes	yes	no	aggregate on species level		
Ranunculus penicillatus	*Ranunculus penicillatus*	(Dumort.) Bab.	yes	yes	no			
Ranunculus penicillatus subsp. penicillatus	*Ranunculus penicillatus*	(Dumort.) Bab.	yes	yes	no	aggregate on species level		
Ranunculus penicillatus subsp. pseudofluitans	*Ranunculus penicillatus*	(Dumort.) Bab.	yes	yes	no	aggregate on species level		
Ranunculus phragmiteti	*Ranunculus auricomus*	L.	yes	yes	no	Buttler & Hand (2023)		
Ranunculus platanifolius	*Ranunculus platanifolius*	L.	yes	yes	yes			

Ranunculus polyanthemoides	*Ranunculus polyanthemos*	L.	yes	yes	yes	Buttler & Hand (2023)		
Ranunculus polyanthemophyllus	*Ranunculus polyanthemos*	L.	yes	yes	yes	Buttler & Hand (2023)		
Ranunculus polyanthemos	*Ranunculus polyanthemos*	L.	yes	yes	yes			
Ranunculus polyanthemos agg.	*Ranunculus polyanthemos*	L.	yes	yes	yes	aggregate on species level		
Ranunculus polyanthemos subsp. nemorosus	*Ranunculus polyanthemos*	L.	yes	yes	yes	aggregate on species level		
Ranunculus polyanthemos subsp. polyanthemoides	*Ranunculus polyanthemos*	L.	yes	yes	yes	aggregate on species level		
Ranunculus polyanthemos subsp. polyanthemophyllus	*Ranunculus polyanthemos*	L.	yes	yes	yes	aggregate on species level		
Ranunculus pseudocassubicus	*Ranunculus auricomus*	L.	yes	yes	no	Buttler & Hand (2023)		
Ranunculus pseudopimus	*Ranunculus auricomus*	L.	yes	yes	no	Buttler & Hand (2023)		
Ranunculus pseudovertumnalis	*Ranunculus auricomus*	L.	yes	yes	no	Buttler & Hand (2023)		
Ranunculus puberulus	*Ranunculus auricomus*	L.	yes	yes	no	Buttler & Hand (2023)		
Ranunculus repens	*Ranunculus repens*	L.	yes	yes	yes			
Ranunculus reptans	*Ranunculus reptans*	L.	yes	yes	yes			
Ranunculus rionii	*Ranunculus rionii*	Lagger	yes	yes	no			
Ranunculus roessleri	*Ranunculus auricomus*	Borch.-Kolb	yes	yes	no	Buttler & Hand (2023)		
Ranunculus rotundatus	*Ranunculus auricomus*	Borch.-Kolb	yes	yes	no	Buttler & Hand (2023)		

Ranunculus sardous	*Ranunculus sardous*	Crantz	yes	yes	yes			
Ranunculus sceleratus	*Ranunculus sceleratus*	L.	yes	yes	yes			
Ranunculus serpens	*Ranunculus polyanthemos*	Schrank	yes	yes	yes	Buttler & Hand (2023)		
Ranunculus stricticaulis	*Ranunculus auricomus*	L.	yes	yes	no	Buttler & Hand (2023)		
Ranunculus suevicus	*Ranunculus auricomus*	L.	yes	yes	no	Buttler & Hand (2023)		
Ranunculus trichophyllus	*Ranunculus trichophyllus*	Chaix	yes	yes	no			
Ranunculus trichophyllus subsp. trichophyllus	*Ranunculus trichophyllus*	Chaix	yes	yes	no	aggregate on species level		
Raphanus raphanistrum	*Raphanus raphanistrum*	L.	yes	yes	yes			
Raphanus raphanistrum agg.	*Raphanus raphanistrum*	L.	yes	yes	yes	aggregate on species level		
Raphanus sativus	*Raphanus sativus*	L.	yes	yes	yes			
Raphanus sativus var. oleiferus	*Raphanus sativus*	L.	yes	yes	yes	aggregate on species level		
Rapistrum rugosum	*Rapistrum rugosum*	(L.) All.	yes	yes	yes			
Rapistrum rugosum subsp. orientale	*Rapistrum rugosum*	(L.) All.	yes	yes	yes	aggregate on species level		
Rapistrum rugosum subsp. rugosum	*Rapistrum rugosum*	(L.) All.	yes	yes	yes	aggregate on species level		
Reseda lutea	*Reseda lutea*	L.	yes	yes	yes			
Reseda luteola	*Reseda luteola*	L.	yes	yes	yes			
Rhamnus cathartica	*Rhamnus cathartica*	L.	yes	yes	yes			
Rhamnus saxatilis	*Rhamnus saxatilis*	Jacq.	yes	yes	yes			
Rhinanthus alectorolophus	*Rhinanthus alectorolophus*	(Scop.) Pollich	yes	yes	yes			

Rhinanthus alectorolophus subsp. alectorolophus	*Rhinanthus alectorolophus*	(Scop.) Pollich	yes	yes	yes	aggregate on species level		
Rhinanthus alectorolophus subsp. buccalis	*Rhinanthus alectorolophus*	(Scop.) Pollich	yes	yes	yes	aggregate on species level		
Rhinanthus angustifolius	*Rhinanthus angustifolius*	Ehrh.	yes	yes	yes			
Rhinanthus glacialis	*Rhinanthus glacialis*	Personnat	yes	yes	no			
Rhinanthus minor	*Rhinanthus minor*	L.	yes	yes	yes			
Rhus hirta	*Rhus hirta*	L.	yes	yes	no			
Rhynchospora alba	*Rhynchospora alba*	(L.) Vahl	yes	yes	yes			
Rhynchospora fusca	*Rhynchospora fusca*	(L.) W. T. Aiton	yes	yes	yes			
Ribes alpinum	*Ribes alpinum*	L.	yes	yes	yes			
Ribes nigrum	*Ribes nigrum*	L.	yes	yes	yes			
Ribes petraeum	*Ribes petraeum*	Wulfen	yes	yes	no			
Ribes rubrum	*Ribes rubrum*	L.	yes	yes	yes			
Ribes rubrum agg.	*Ribes rubrum*	L.	yes	yes	yes	aggregate on species level		
Ribes rubrum var. domesticum	*Ribes rubrum*	L.	yes	yes	yes	aggregate on species level		
Ribes rubrum var. rubrum	*Ribes rubrum*	L.	yes	yes	yes	aggregate on species level		
Ribes uva-crispa	*Ribes uva-crispa*	L.	yes	yes	yes			*RIBES UVA-CRISPA*
Robinia pseudoacacia	*Robinia pseudoacacia*	L.	yes	yes	yes			
Rorippa amphibia	*Rorippa amphibia*	(L.) Besser	yes	yes	yes			
Rorippa anceps	*Rorippa anceps*	(Wahlenb.) Rchb.	yes	yes	no			
Rorippa austriaca	*Rorippa austriaca*	(Crantz) Besser	yes	yes	no			
Rorippa palustris	*Rorippa palustris*	(L.) Besser	yes	yes	yes			
Rorippa pyrenaica	*Rorippa pyrenaica*	(L.) Rchb.	yes	yes	no			

Rorippa sylvestris	*Rorippa sylvestris*	(L.) Besser	yes	yes	yes			
Rosa abietina	*Rosa abietina*	Gren. ex H. Christ	yes	yes	no			
Rosa agrestis	*Rosa agrestis*	Savi	yes	yes	yes			
Rosa arvensis	*Rosa arvensis*	Huds.	yes	yes	yes			
Rosa caesia	*Rosa caesia*	Sm.	yes	yes	no			
Rosa caesia agg.	*Rosa caesia*	Sm.	yes	yes	no	aggregate on species level		
Rosa canina	*Rosa canina*	L.	yes	yes	yes			
Rosa canina agg.	*Rosa canina*	L.	yes	yes	yes	Import from raw data		
Rosa canina subsp. glauca	*Rosa canina*	L.	yes	yes	yes	aggregate on species level		
Rosa canina var. andegavensis	*Rosa canina*	L.	yes	yes	yes	aggregate on species level		
Rosa canina var. blondaeana	*Rosa canina*	L.	yes	yes	yes	aggregate on species level		
Rosa canina var. canina	*Rosa canina*	L.	yes	yes	yes	aggregate on species level		
Rosa canina var. dumalis	*Rosa canina*	L.	yes	yes	yes	aggregate on species level		
Rosa corymbifera	*Rosa corymbifera*	Borkh.	yes	yes	no			
Rosa corymbifera var. corymbifera	*Rosa corymbifera*	Borkh.	yes	yes	no	aggregate on species level		
Rosa corymbifera var. deseglisei	*Rosa corymbifera*	Borkh.	yes	yes	no	aggregate on species level		
Rosa dumalis	*Rosa dumalis*	Bechst.	yes	yes	no			

Rosa dumalis agg.	*Rosa dumalis*	Bechst.	yes	yes	no	aggregate on species level		
Rosa elliptica	*Rosa elliptica*	Tausch	yes	yes	yes			*Rosa inodora*
Rosa foetida	*Rosa foetida*	Mill.	yes	yes	no			
Rosa gallica	*Rosa gallica*	L.	yes	yes	no			
Rosa glauca	*Rosa glauca*	Pourr.	yes	yes	yes			
Rosa inodora	*Rosa inodora*	Fr.	yes	yes	yes			
Rosa jundzillii	*Rosa jundzillii*	Wallr.	yes	yes	yes			*Rosa marginata*
Rosa majalis	*Rosa majalis*	Herrm.	yes	yes	yes			
Rosa micrantha	*Rosa micrantha*	Borrer ex Sm.	yes	yes	yes			
Rosa multiflora	*Rosa multiflora*	Thunb.	yes	yes	yes			
Rosa pendulina	*Rosa pendulina*	L.	yes	yes	yes			
Rosa pseudoscabriuscula	*Rosa pseudoscabriuscula*	(R. Keller) Henker & G. Schulze	yes	yes	no			
Rosa rubiginosa	*Rosa rubiginosa*	L.	yes	yes	yes			
Rosa rubiginosa agg.	*Rosa rubiginosa*	L.	yes	yes	yes	aggregate on species level		
Rosa rugosa	*Rosa rugosa*	Thunb.	yes	yes	yes			
Rosa sherardii	*Rosa sherardii*	Davies	yes	yes	no			
Rosa spinosissima	*Rosa spinosissima*	L.	yes	yes	no			
Rosa stylosa	*Rosa stylosa*	Desv.	yes	yes	no			
Rosa subcanina	*Rosa subcanina*	(H. Christ) R. Keller	yes	yes	no			
Rosa subcollina	*Rosa subcollina*	(H. Christ) R. Keller	yes	yes	no			
Rosa tomentella	*Rosa tomentella*	(Léman) F. Herm.	yes	yes	no			
Rosa tomentella agg.	*Rosa tomentella*	(Léman) F. Herm.	yes	yes	no	aggregate on species level		
Rosa tomentosa	*Rosa tomentosa*	Sm.	yes	yes	no			
Rosa villosa	*Rosa villosa*	L.	yes	yes	yes			

Rosa villosa agg.	*Rosa villosa*	L.	yes	yes	yes	aggregate on species level		
Rubus adscitus	*Rubus sect. Rubus*		yes	yes	yes	Buttler et al 2018		
Rubus albiflorus	*Rubus sect. Rubus*		yes	yes	yes	Buttler et al 2018		
Rubus allegheniensis	*Rubus sect. Rubus*		yes	yes	yes	Buttler et al 2018		
Rubus ambulans	*Rubus sect. Rubus*		yes	yes	yes	Buttler et al 2018		
Rubus amiantinus	*Rubus sect. Rubus*		yes	yes	yes	Buttler et al 2018		
Rubus amphimalacus	*Rubus sect. Corylifolii*		yes	yes	no	Buttler et al 2018		
Rubus appropinquatus	*Rubus sect. Rubus*		yes	yes	yes	Buttler et al 2018		
Rubus apricus	*Rubus sect. Rubus*		yes	yes	yes	Buttler et al 2018		
Rubus armeniacus	*Rubus sect. Rubus*		yes	yes	yes	Buttler et al 2018		
Rubus atrichantherus	*Rubus sect. Rubus*		yes	yes	yes	Buttler et al 2018		
Rubus austroslovacus	*Rubus sect. Rubus*		yes	yes	yes	Buttler et al 2018		
Rubus baruthicus	*Rubus sect. Corylifolii*		yes	yes	no	Buttler et al 2018		
Rubus bavaricus	*Rubus sect. Rubus*		yes	yes	yes	Buttler et al 2018		
Rubus bertramii	*Rubus sect. Rubus*		yes	yes	yes	Buttler et al 2018		
Rubus bifrons	*Rubus sect. Rubus*		yes	yes	yes	Buttler et al 2018		

Rubus bregutiensis	*Rubus sect. Rubus*		yes	yes	yes	Buttler et al 2018		
Rubus caesius	*Rubus caesius*	L.	yes	yes	yes	Durka and Michalski 2012		
Rubus caflischii	*Rubus sect. Rubus*		yes	yes	yes	Buttler et al 2018		
Rubus camptostachys	*Rubus sect. Corylifolii*		yes	yes	no	Buttler et al 2018		
Rubus canaliculatus	*Rubus sect. Rubus*		yes	yes	yes	Buttler et al 2018		
Rubus canescens	*Rubus sect. Rubus*		yes	yes	yes	Buttler et al 2018		
Rubus canescens var. canescens	*Rubus sect. Rubus*		yes	yes	yes	Buttler et al 2018		
Rubus canescens var. glabratus	*Rubus sect. Rubus*		yes	yes	yes	Buttler et al 2018		
Rubus caninitergi	*Rubus sect. Rubus*		yes	yes	yes	Buttler et al 2018		
Rubus clusii	*Rubus sect. Rubus*		yes	yes	yes	Buttler et al 2018		
Rubus condensatus	*Rubus sect. Rubus*		yes	yes	yes	Buttler et al 2018		
Rubus conspicuus	*Rubus sect. Rubus*		yes	yes	yes	Buttler et al 2018		
Rubus constrictus	*Rubus sect. Rubus*		yes	yes	yes	Buttler et al 2018		
Rubus corylifolius	*Rubus sect. Corylifolii*		yes	yes	no	Buttler et al 2018		
Rubus corylifolius agg.	*Rubus sect. Corylifolii*		yes	yes	no	Buttler et al 2018		
Rubus curvaciculatus	*Rubus sect. Corylifolii*		yes	yes	no	Buttler et al 2018		

Rubus cuspidatus	*Rubus sect. Corylifolii*		yes	yes	no	Buttler et al 2018		
Rubus devitatus	*Rubus sect. Rubus*		yes	yes	yes	Buttler et al 2018		
Rubus distractus	*Rubus sect. Rubus*		yes	yes	yes	Buttler et al 2018		
Rubus divaricatus	*Rubus sect. Rubus*		yes	yes	yes	Buttler et al 2018		
Rubus drejeri	*Rubus sect. Rubus*		yes	yes	yes	Buttler et al 2018		
Rubus dumetorum	*Rubus sect. Corylifolii*		yes	yes	no	https://daten.bayernflora.de/de/info_pflanzen.php?taxnr=35926		
Rubus elatior	*Rubus sect. Rubus*		yes	yes	yes	Buttler et al 2018		
Rubus elegans	*Rubus sect. Rubus*		yes	yes	yes	Buttler et al 2018		
Rubus elegantispinosus	*Rubus sect. Rubus*		yes	yes	yes	Buttler et al 2018		
Rubus epipsilos	*Rubus sect. Rubus*		yes	yes	yes	Buttler et al 2018		
Rubus fabrimontanus	*Rubus sect. Rubus*		yes	yes	yes	Buttler et al 2018		
Rubus fasciculatiformis	*Rubus sect. Corylifolii*		yes	yes	no	Buttler et al 2018		
Rubus fasciculatus	*Rubus sect. Corylifolii*		yes	yes	no	Buttler et al 2018		
Rubus fissipetalus	*Rubus sect. Rubus*		yes	yes	yes	Buttler et al 2018		

Rubus flexuosus	*Rubus sect. Rubus*		yes	yes	yes	Buttler et al 2018		
Rubus flos-amygdalae	*Rubus sect. Rubus*		yes	yes	yes	Buttler et al 2018		
Rubus foliosus	*Rubus sect. Rubus*		yes	yes	yes	Buttler et al 2018		
Rubus foliosus var. corymbosus	*Rubus sect. Rubus*		yes	yes	yes	Buttler et al 2018		
Rubus franconicus	*Rubus sect. Corylifolii*		yes	yes	no	Buttler et al 2018		
Rubus fruticosus agg.	*Rubus sect. Rubus*		yes	yes	yes	Buttler et al 2018		
Rubus fuernrohrii	*Rubus sect. Corylifolii*		yes	yes	no	Buttler et al 2018		
Rubus geniculatus	*Rubus sect. Rubus*		yes	yes	yes	Buttler et al 2018		
Rubus glandulosus	*Rubus sect. Rubus*		yes	yes	yes	Buttler et al 2018		
Rubus godronii	*Rubus sect. Rubus*		yes	yes	yes	Buttler et al 2018		
Rubus gothicus	*Rubus sect. Corylifolii*		yes	yes	no	Buttler et al 2018		
Rubus grabowskii	*Rubus sect. Rubus*		yes	yes	yes	Buttler et al 2018		
Rubus grabowskii agg.	*Rubus sect. Rubus*		yes	yes	yes	Buttler et al 2018		
Rubus gracilis	*Rubus sect. Rubus*		yes	yes	yes	Buttler et al 2018		
Rubus gracilis subsp. gracilis	*Rubus sect. Rubus*		yes	yes	yes	Buttler et al 2018		
Rubus gremlii	*Rubus sect. Rubus*		yes	yes	yes	Buttler et al 2018		

Rubus grossus	*Rubus sect. Corylifolii*		yes	yes	no	Buttler et al 2018		
Rubus hadracanthos	*Rubus sect. Corylifolii*		yes	yes	no	Buttler et al 2018		
Rubus haesitans	*Rubus sect. Corylifolii*		yes	yes	no	Buttler et al 2018		
Rubus henrici-egonis	*Rubus sect. Rubus*		yes	yes	yes	Buttler et al 2018		
Rubus hirtus	*Rubus sect. Rubus*		yes	yes	yes	Buttler et al 2018		
Rubus histrionicus	*Rubus sect. Corylifolii*		yes	yes	no	Buttler et al 2018		
Rubus hypomalacus	*Rubus sect. Rubus*		yes	yes	yes	Buttler et al 2018		
Rubus idaeus	*Rubus idaeus*	L.	yes	yes	yes			
Rubus integribasis	*Rubus sect. Rubus*		yes	yes	yes	Buttler et al 2018		
Rubus josefianus	*Rubus sect. Corylifolii*		yes	yes	no	Buttler et al 2018		
Rubus koehleri	*Rubus sect. Rubus*		yes	yes	yes	Buttler et al 2018		
Rubus laciniatus	*Rubus sect. Rubus*		yes	yes	yes	Buttler et al 2018		
Rubus lamprocaulos	*Rubus sect. Corylifolii*		yes	yes	no	Buttler et al 2018		
Rubus leucophaeus	*Rubus sect. Corylifolii*		yes	yes	no	Buttler et al 2018		
Rubus lictorum	*Rubus sect. Corylifolii*		yes	yes	no	Buttler et al 2018		
Rubus lidforssii	*Rubus sect. Corylifolii*		yes	yes	no	Buttler et al 2018		
Rubus limitis	*Rubus sect. Corylifolii*		yes	yes	no	Buttler et al 2018		

Rubus lividus	*Rubus sect. Rubus*		yes	yes	yes	Buttler et al 2018		
Rubus lusaticus	*Rubus sect. Rubus*		yes	yes	yes	Buttler et al 2018		
Rubus macrophyllus	*Rubus sect. Rubus*		yes	yes	yes	Buttler et al 2018		
Rubus macrostachys	*Rubus sect. Rubus*		yes	yes	yes	Buttler et al 2018		
Rubus marianus	*Rubus sect. Rubus*		yes	yes	yes	Buttler et al 2018		
Rubus mollis	*Rubus sect. Corylifolii*		yes	yes	no	Buttler et al 2018		
Rubus montanus	*Rubus sect. Rubus*		yes	yes	yes	Buttler et al 2018		
Rubus mougeotii	*Rubus sect. Corylifolii*		yes	yes	no	Buttler et al 2018		
Rubus multicaudatus	*Rubus sect. Rubus*		yes	yes	yes	Buttler et al 2018		
Rubus nemoralis	*Rubus sect. Rubus*		yes	yes	yes	Buttler et al 2018		
Rubus nemorosus	*Rubus sect. Corylifolii*		yes	yes	no	Buttler et al 2018		
Rubus nessensis	*Rubus sect. Rubus*		yes	yes	yes	Buttler et al 2018		
Rubus nessensis subsp. nessensis	*Rubus sect. Rubus*		yes	yes	yes	Buttler et al 2018		
Rubus neumannianus	*Rubus sect. Rubus*		yes	yes	yes	Buttler et al 2018		
Rubus oberdorferi	*Rubus sect. Rubus*		yes	yes	yes	Buttler et al 2018		
Rubus obtusangulus	*Rubus sect. Rubus*		yes	yes	yes	Buttler et al 2018		
Rubus odoratus	*Rubus odoratus*	L.	yes	yes	no			

Rubus oenensis	*Rubus sect. Rubus*		yes	yes	yes	Buttler et al 2018		
Rubus orthostachyoides	*Rubus sect. Corylifolii*		yes	yes	no	Buttler et al 2018		
Rubus orthostachys	*Rubus sect. Corylifolii*		yes	yes	no	Buttler et al 2018		
Rubus pallidus	*Rubus sect. Rubus*		yes	yes	yes	Buttler et al 2018		
Rubus parthenocissus	*Rubus sect. Rubus*		yes	yes	yes	Buttler et al 2018		
Rubus pedemontanus	*Rubus sect. Rubus*		yes	yes	yes	Buttler et al 2018		
Rubus pedica	*Rubus sect. Rubus*		yes	yes	yes	Buttler et al 2018		
Rubus pericrispatus	*Rubus sect. Rubus*		yes	yes	yes	Buttler et al 2018		
Rubus perlongus	*Rubus sect. Rubus*		yes	yes	yes	Buttler et al 2018		
Rubus perperus	*Rubus sect. Rubus*		yes	yes	yes	Buttler et al 2018		
Rubus phyllostachys	*Rubus sect. Rubus*		yes	yes	yes	Buttler et al 2018		
Rubus placidus	*Rubus sect. Corylifolii*		yes	yes	no	Buttler et al 2018		
Rubus plicatus	*Rubus sect. Rubus*		yes	yes	yes	Buttler et al 2018		
Rubus plicatus f. rosulentus	*Rubus sect. Rubus*		yes	yes	yes	Buttler et al 2018		
Rubus praecox	*Rubus sect. Rubus*		yes	yes	yes	Buttler et al 2018		
Rubus pruinosus	*Rubus sect. Corylifolii*		yes	yes	no	Buttler et al 2018		

Rubus pseudinfestus	*Rubus sect. Rubus*		yes	yes	yes	Buttler et al 2018		
Rubus pulchricaulis	*Rubus sect. Rubus*		yes	yes	yes	Buttler et al 2018		
Rubus pyramidalis	*Rubus sect. Rubus*		yes	yes	yes	Buttler et al 2018		
Rubus radula	*Rubus sect. Rubus*		yes	yes	yes	Buttler et al 2018		
Rubus remotifolius	*Rubus sect. Corylifolii*		yes	yes	no	Buttler et al 2018		
Rubus rhombicus	*Rubus sect. Corylifolii*		yes	yes	no	Buttler et al 2018		
Rubus rotundifoliatus	*Rubus rotundifoliatus*	Sudre	yes	yes	no	Buttler et al 2018		
Rubus rudis	*Rubus sect. Rubus*		yes	yes	yes	Buttler et al 2018		
Rubus saxatilis	*Rubus saxatilis*	L.	yes	yes	yes	is in daphne		
Rubus saxicola	*Rubus sect. Rubus*		yes	yes	yes	Buttler et al 2018		
Rubus scabrosus	*Rubus sect. Corylifolii*		yes	yes	no	Buttler et al 2018		
Rubus schleicheri	*Rubus sect. Rubus*		yes	yes	yes	Buttler et al 2018		
Rubus schnedleri	*Rubus sect. Rubus*		yes	yes	yes	Buttler et al 2018		
Rubus sciocharis	*Rubus sect. Rubus*		yes	yes	yes	https://daten.bayernflora.de/de/info_pflanzen.php?taxnr=5044		

Rubus senticosus	*Rubus sect. Rubus*		yes	yes	yes	Buttler et al 2018		
Rubus ser. Glandulosi	*Rubus sect. Rubus*		yes	yes	yes	https://daten.bayernflora.de/de/info_pflanzen.php?taxnr=27547&suchtext=Rubus&g=&de=		
Rubus Ser. Pallidi	*Rubus sect. Rubus*		yes	yes	yes	https://daten.bayernflora.de/de/info_pflanzen.php?taxnr=27547&suchtext=Rubus&g=&de=		
Rubus Ser. Subradula	*Rubus sect. Corylifolii*		yes	yes	no	https://daten.bayernflora.de/de/info_pflanzen.php?taxnr=27547&suchtext=Rubus&g=&de=		

Rubus spinulatus	*Rubus sect. Rubus*		yes	yes	yes	https://daten.bayernflora.de/de/info_pflanzen.php?taxnr=27538		
Rubus stimulifer	*Rubus sect. Rubus*		yes	yes	yes	Buttler et al 2018		
Rubus striaticaulis	*Rubus sect. Rubus*		yes	yes	yes	Buttler et al 2018		
Rubus subcordatus	*Rubus sect. Rubus*		yes	yes	yes	Buttler et al 2018		
Rubus suevicola	*Rubus sect. Corylifolii*		yes	yes	no	Buttler et al 2018		
Rubus sulcatus	*Rubus sect. Rubus*		yes	yes	yes	Buttler et al 2018		
Rubus tauni	*Rubus sect. Rubus*		yes	yes	yes	Buttler et al 2018		
Rubus tereticaulis	*Rubus sect. Rubus*		yes	yes	yes	Buttler et al 2018		
Rubus thelybatos	*Rubus sect. Rubus*		yes	yes	yes	Buttler et al 2018		
Rubus transvestitus	*Rubus sect. Rubus*		yes	yes	yes	Buttler et al 2018		
Rubus ulmifolius	*Rubus sect. Rubus*		yes	yes	yes	Buttler et al 2018		
Rubus vestitus	*Rubus sect. Rubus*		yes	yes	yes	Buttler et al 2018		
Rubus villarsianus	*Rubus sect. Corylifolii*		yes	yes	no	Buttler et al 2018		
Rubus visurgianus	*Rubus sect. Corylifolii*		yes	yes	no	Buttler et al 2018		

Rubus walteri	*Rubus sect. Rubus*		yes	yes	yes	Buttler et al 2018		
Rubus winteri	*Rubus sect. Rubus*		yes	yes	yes	Buttler et al 2018		
Rudbeckia hirta	*Rudbeckia hirta*	L.	yes	yes	yes			
Rudbeckia laciniata	*Rudbeckia laciniata*	L.	yes	yes	yes			
Rumex acetosa	*Rumex acetosa*	L.	yes	yes	yes			
Rumex acetosella	*Rumex acetosella*	L.	yes	yes	yes			
Rumex acetosella agg.	*Rumex acetosella*	L.	yes	yes	yes	aggregate on species level		
Rumex acetosella subsp. acetosella	*Rumex acetosella*	L.	yes	yes	yes	aggregate on species level		
Rumex acetosella subsp. angiocarpus	*Rumex acetosella*	L.	yes	yes	yes	aggregate on species level		
Rumex acetosella subsp. pyrenaicus	*Rumex acetosella*	L.	yes	yes	yes	aggregate on species level		
Rumex acetosella var. acetosella	*Rumex acetosella*	L.	yes	yes	yes	aggregate on species level		
Rumex acetosella var. tenuifolius	*Rumex acetosella*	L.	yes	yes	yes	aggregate on species level		
Rumex aquaticus	*Rumex aquaticus*	L.	yes	yes	yes			
Rumex arifolius	*Rumex arifolius*	All.	yes	yes	no			
Rumex conglomeratus	*Rumex conglomeratus*	Murray	yes	yes	yes			
Rumex crispus	*Rumex crispus*	L.	yes	yes	yes			
Rumex hydrolapathum	*Rumex hydrolapathum*	Huds.	yes	yes	yes			
Rumex maritimus	*Rumex maritimus*	L.	yes	yes	yes			
Rumex obtusifolius	*Rumex obtusifolius*	L.	yes	yes	yes			

Rumex obtusifolius subsp. obtusifolius	*Rumex obtusifolius*	L.	yes	yes	yes	aggregate on species level		
Rumex obtusifolius subsp. sylvestris	*Rumex obtusifolius*	L.	yes	yes	yes	aggregate on species level		
Rumex obtusifolius subsp. transiens	*Rumex obtusifolius*	L.	yes	yes	yes	aggregate on species level		
Rumex palustris	*Rumex palustris*	Sm.	yes	yes	yes			
Rumex patientia	*Rumex patientia*	L.	yes	yes	no			
Rumex patientia agg.	*Rumex patientia*	L.	yes	yes	no	aggregate on species level		
Rumex pratensis	*Rumex pratensis*	Mert. & W. D. J. Koch	yes	yes	no		Kumar et al 2022, sistertaxon to R. obtusifolius	
Rumex pseudoalpinus	*Rumex alpinus*	NA	yes	yes	no	POWO (2023)		
Rumex sanguineus	*Rumex sanguineus*	L.	yes	yes	yes			
Rumex scutatus	*Rumex scutatus*	L.	yes	yes	yes			
Rumex thyrsiflorus	*Rumex thyrsiflorus*	Fingerh.	yes	yes	yes			
Ruta graveolens	*Ruta graveolens*	L.	yes	yes	no			
Sagina apetala	*Sagina apetala*	Ard.	yes	yes	yes			
Sagina apetala agg.	*Sagina apetala*	Ard.	yes	yes	yes	aggregate on species level		

Sagina micropetala	*Sagina apetala*	Ard.	yes	yes	no	taxonomic inconsistencies between first and second mapping		
Sagina nodosa	*Sagina nodosa*	(L.) Fenzl	yes	yes	yes			
Sagina procumbens	*Sagina procumbens*	L.	yes	yes	yes			
Sagina procumbens var. procumbens	*Sagina procumbens*	L.	yes	yes	yes	aggregate on species level		
Sagittaria sagittifolia	*Sagittaria sagittifolia*	L.	yes	yes	yes			
Salix alba	*Salix alba*	L.	yes	yes	yes			
Salix alba agg.	*Salix alba*	L.	yes	yes	yes	aggregate on species level		
Salix alba var. alba	*Salix alba*	L.	yes	yes	yes	aggregate on species level		
Salix alba var. britzensis	*Salix alba*	L.	yes	yes	yes	aggregate on species level		
Salix alba var. caerulea	*Salix alba*	L.	yes	yes	yes	aggregate on species level		
Salix alba var. vitellina	*Salix alba*	L.	yes	yes	yes	aggregate on species level		
Salix appendiculata	*Salix appendiculata*	Vill.	yes	yes	yes			
Salix aurita	*Salix aurita*	L.	yes	yes	yes			
Salix caprea	*Salix caprea*	L.	yes	yes	yes			
Salix cinerea	*Salix cinerea*	L.	yes	yes	yes			
Salix cinerea subsp. cinerea	*Salix cinerea*	L.	yes	yes	yes	aggregate on species level		

Salix cinerea subsp. oleifolia	*Salix cinerea*	Brot.	yes	yes	yes	aggregate on species level		
Salix daphnoides	*Salix daphnoides*	Vill.	yes	yes	yes			
Salix dasyclados	*Salix dasyclados*	Wimm.	yes	yes	no			
Salix eleagnos	*Salix eleagnos*	Scop.	yes	yes	no			
Salix fragilis	*Salix fragilis*	L.	yes	yes	yes			*Salix fragilis _x*
Salix fragilis agg.	*Salix fragilis*	L.	yes	yes	yes	aggregate on species level		
Salix multinervis	*Salix multinervis*	Doell	yes	yes	no		*Salix x multinervis*	
Salix myrsinifolia	*Salix myrsinifolia*	Sm.	yes	yes	yes			
Salix pentandra	*Salix pentandra*	L.	yes	yes	yes			
Salix purpurea	*Salix purpurea*	L.	yes	yes	yes			
Salix purpurea subsp. gracilis	*Salix purpurea*	L.	yes	yes	yes	aggregate on species level		
Salix repens	*Salix repens*	L.	yes	yes	yes			
Salix repens agg.	*Salix repens*	L.	yes	yes	yes	aggregate on species level		
Salix repens subsp. dunensis	*Salix repens*	L.	yes	yes	yes	aggregate on species level		
Salix repens subsp. repens	*Salix repens*	L.	yes	yes	yes	aggregate on species level		
Salix rubens	*Salix rubens*	Schrank	yes	yes	no		*Salix x subens*	
Salix rubra	*Salix rubra*	Huds.	yes	yes	no		*Salix x rubra*	
Salix smithiana	*Salix smithiana*	Willd.	yes	yes	no		*Salix x smithiana*	
Salix starkeana	*Salix starkeana*	Willd.	yes	yes	no			
Salix triandra	*Salix triandra*	L.	yes	yes	yes			

Salix triandra subsp. amygdalina	*Salix triandra*	L.	yes	yes	yes	aggregate on species level		
Salix triandra subsp. triandra	*Salix triandra*	L.	yes	yes	yes	aggregate on species level		
Salix viminalis	*Salix viminalis*	L.	yes	yes	no			
Salsola kali	*Salsola kali*	L.	yes	yes	yes			*Kali turgidum*
Salsola kali subsp. Kali	*Salsola kali*	L.	yes	yes	yes	aggregate on species level		
Salsola kali subsp. tragus	*Salsola kali*	L.	yes	yes	yes	aggregate on species level		
Salvia glutinosa	*Salvia glutinosa*	L.	yes	yes	yes			
Salvia nemorosa	*Salvia nemorosa*	L.	yes	yes	yes			
Salvia pratensis	*Salvia pratensis*	L.	yes	yes	yes			
Salvia verticillata	*Salvia verticillata*	L.	yes	yes	yes			
Salvinia natans	*Salvinia natans*	(L.) All.	yes	yes	no			
Sambucus ebulus	*Sambucus ebulus*	L.	yes	yes	yes			
Sambucus nigra	*Sambucus nigra*	L.	yes	yes	yes			
Sambucus racemosa	*Sambucus racemosa*	L.	yes	yes	yes			
Samolus valerandi	*Samolus valerandi*	L.	yes	yes	yes			
Sanguisorba minor	*Sanguisorba minor*	Scop.	yes	yes	yes			
Sanguisorba minor subsp. balearica	*Sanguisorba minor*	Scop.	yes	yes	yes	aggregate on species level		
Sanguisorba minor subsp. minor	*Sanguisorba minor*	Scop.	yes	yes	yes	aggregate on species level		
Sanguisorba minor subsp. minor	*Sanguisorba minor*	Scop.	yes	yes	yes	aggregate on species level		

Sanguisorba minor subsp. polygama	*Sanguisorba minor*	Scop.	yes	yes	yes	aggregate on species level		
Sanguisorba officinalis	*Sanguisorba officinalis*	L.	yes	yes	yes			
Sanicula europaea	*Sanicula europaea*	L.	yes	yes	yes			
Saponaria officinalis	*Saponaria officinalis*	L.	yes	yes	yes			
Saxifraga granulata	*Saxifraga granulata*	L.	yes	yes	yes			
Saxifraga paniculata	*Saxifraga paniculata*	Mill.	yes	yes	yes			
Saxifraga rosacea	*Saxifraga rosacea*	Moench	yes	yes	no			
Saxifraga rosacea subsp. rosacea	*Saxifraga rosacea*	Moench	yes	yes	no	aggregate on species level		
Saxifraga rotundifolia	*Saxifraga rotundifolia*	L.	yes	yes	yes			
Saxifraga stellaris	*Saxifraga stellaris*	L.	yes	yes	yes			*Micranthes stellaris*
Saxifraga stellaris subsp. robusta	*Saxifraga stellaris*	L.	yes	yes	yes	aggregate on species level		
Saxifraga tridactylites	*Saxifraga tridactylites*	L.	yes	yes	yes			
Saxifraga tridactylites subsp. tridactylites	*Saxifraga tridactylites*	L.	yes	yes	yes	aggregate on species level		
Scabiosa canescens	*Scabiosa canescens*	Waldst. & Kit.	yes	yes	yes			
Scabiosa columbaria	*Scabiosa columbaria*	L.	yes	yes	yes			
Scabiosa columbaria subsp. columbaria	*Scabiosa columbaria*	L.	yes	yes	yes	aggregate on species level		
Scabiosa ochroleuca	*Scabiosa ochroleuca*	L.	yes	yes	yes			

Scandix pecten-veneris	*Scandix pecten-veneris*	L.	yes	yes	yes			*SCANDIX PECTEN-VENERIS*
Scandix pecten-veneris subsp. pecten-veneris	*Scandix pecten-veneris*	L.	yes	yes	yes	aggregate on species level		
Scheuchzeria palustris	*Scheuchzeria palustris*	L.	yes	yes	yes			
Schoenoplectus lacustris	*Schoenoplectus lacustris*	(L.) Palla	yes	yes	yes			
Schoenoplectus lacustris agg.	*Schoenoplectus lacustris*	(L.) Palla	yes	yes	yes	aggregate on species level		
Schoenoplectus mucronatus	*Schoenoplectus mucronatus*	(L.) Palla	yes	yes	no			
Schoenoplectus supinus	*Schoenoplectus supinus*	(L.) Palla	yes	yes	no			
Schoenoplectus tabernaemontani	*Schoenoplectus tabernaemontani*	(C. C. Gmel.) Palla	yes	yes	yes			
Schoenus ferrugineus	*Schoenus ferrugineus*	L.	yes	yes	yes			
Schoenus nigricans	*Schoenus nigricans*	L.	yes	yes	yes			
Scilla bifolia	*Scilla bifolia*	L.	yes	yes	yes			
Scilla siberica	*Scilla siberica*	Haw.	yes	yes	yes			
Scirpus radicans	*Scirpus radicans*	Schkuhr	yes	yes	no			
Scirpus sylvaticus	*Scirpus sylvaticus*	L.	yes	yes	yes			
Scleranthus annuus	*Scleranthus annuus*	L.	yes	yes	yes			
Scleranthus annuus agg.	*Scleranthus annuus*	L.	yes	yes	yes	aggregate on species level		
Scleranthus perennis	*Scleranthus perennis*	L.	yes	yes	yes			
Scleranthus polycarpos	*Scleranthus polycarpos*	(L.) Bonnier & Layens	yes	yes	yes			*Scleranthus annuus*
Sclerochloa dura	*Sclerochloa dura*	(L.) P. Beauv.	yes	yes	no			
Scorzonera hispanica	*Scorzonera hispanica*	L.	yes	yes	yes			

Scorzonera humilis	*Scorzonera humilis*	L.	yes	yes	yes			
Scorzonera laciniata	*Scorzonera laciniata*	L.	yes	yes	yes		*Podospermum laciniatum*	
Scrophularia auriculata	*Scrophularia auriculata*	L.	yes	yes	yes			
Scrophularia canina	*Scrophularia canina*	L.	yes	yes	yes			
Scrophularia nodosa	*Scrophularia nodosa*	L.	yes	yes	yes			
Scrophularia umbrosa	*Scrophularia umbrosa*	Dumort.	yes	yes	yes			
Scrophularia umbrosa subsp. neesii	*Scrophularia umbrosa*	Dumort.	yes	yes	yes	aggregate on species level		
Scrophularia umbrosa subsp. umbrosa	*Scrophularia umbrosa*	Dumort.	yes	yes	yes	aggregate on species level		
Scutellaria altissima	*Scutellaria altissima*	L.	yes	yes	no			
Scutellaria galericulata	*Scutellaria galericulata*	L.	yes	yes	yes			
Scutellaria minor	*Scutellaria minor*	Huds.	yes	yes	yes			
Securigera varia	*Securigera varia*	(L.) Lassen	yes	yes	yes			
Sedum acre	*Sedum acre*	L.	yes	yes	yes			
Sedum album	*Sedum album*	L.	yes	yes	yes			
Sedum annuum	*Sedum annuum*	L.	yes	yes	yes			
Sedum dasyphyllum	*Sedum dasyphyllum*	L.	yes	yes	yes			
Sedum hispanicum	*Sedum hispanicum*	L.	yes	yes	no			
Sedum maximum	*Sedum maximum*	(L.) Hoffm.	yes	yes	no			
Sedum rupestre	*Sedum rupestre*	L.	yes	yes	no			
Sedum rupestre agg.	*Sedum rupestre*	L.	yes	yes	no	aggregate on species level		
Sedum sexangulare	*Sedum sexangulare*	L.	yes	yes	yes			
Sedum spurium	*Sedum spurium*	M. Bieb.	yes	yes	yes			*Phedimus spurius*

Sedum telephium	*Sedum telephium*	(L.) Ohba	yes	yes	yes			*Hylotelephium telephium*
Sedum telephium agg.	*Sedum telephium*	(L.) Ohba	yes	yes	yes	aggregate on species level		
Sedum telephium subsp. fabaria	*Sedum telephium*	(L.) Ohba	yes	yes	yes	aggregate on species level		
Sedum telephium subsp. telephium	*Sedum telephium*	(L.) Ohba	yes	yes	yes	aggregate on species level		
Sedum villosum	*Sedum villosum*	L.	yes	yes	no			
Selinum carvifolia	*Selinum carvifolia*	(L.) L.	yes	yes	yes			
Sempervivum tectorum	*Sempervivum tectorum*	L.	yes	yes	yes			
Senecio alpinus	*Senecio alpinus*	(L.) Scop.	yes	yes	no			
Senecio aquaticus	*Senecio aquaticus*	Hill	yes	yes	yes			*Jacobaea aquatica*
Senecio aquaticus agg.	*Senecio aquaticus*	Hill	yes	yes	yes	aggregate on species level		
Senecio cacaliaster subsp. hercynicus	*Senecio hercynicus*	NA	yes	yes	no	Sebald et al. (1996)		
Senecio erraticus subsp. barbareifolius	*Senecio erraticus*	(Wimm. & Grab.) Beger	yes	yes	no	aggregate on species level		
Senecio erucifolius	*Senecio erucifolius*	L.	yes	yes	yes			*Jacobaea erucifolia*
Senecio erucifolius subsp. erucifolius	*Senecio erucifolius*	L.	yes	yes	yes	aggregate on species level		

Senecio erucifolius subsp. tenuifolius	*Senecio erucifolius*	L.	yes	yes	yes	aggregate on species level		
Senecio hercynicus	*Senecio hercynicus*	Herborg	yes	yes	no			
Senecio inaequidens	*Senecio inaequidens*	DC.	yes	yes	yes			
Senecio jacobaea	*Senecio jacobaea*	L.	yes	yes	yes			*Jacobaea vulgaris*
Senecio jacobaea subsp. jacobaea	*Senecio jacobaea*	L.	yes	yes	yes	aggregate on species level		
Senecio nemorensis agg.	*Senecio nemorensis*	L.	yes	yes	no	aggregate on species level		
Senecio ovatus	*Senecio ovatus*	C. C. Gmel.	yes	yes	no			
Senecio ovatus subsp. alpestris	*Senecio ovatus*	C. C. Gmel.	yes	yes	no	aggregate on species level		
Senecio ovatus subsp. ovatus	*Senecio ovatus*	C. C. Gmel.	yes	yes	no	aggregate on species level		
Senecio paludosus	*Senecio paludosus*	L.	yes	yes	yes			*Jacobaea paludosa*
Senecio sarracenicus	*Senecio hercynicus*	L.	yes	yes	no	Sebald et al. (1996)		
Senecio sylvaticus	*Senecio sylvaticus*	L.	yes	yes	yes			
Senecio vernalis	*Senecio vernalis*	Waldst. & Kit.	yes	yes	no			
Senecio viscosus	*Senecio viscosus*	L.	yes	yes	yes			
Senecio vulgaris	*Senecio vulgaris*	L.	yes	yes	yes			
Serratula tinctoria	*Serratula tinctoria*	L.	yes	yes	yes			
Serratula tinctoria subsp. tinctoria	*Serratula tinctoria*	L.	yes	yes	yes	aggregate on species level		
Seseli annuum	*Seseli annuum*	L.	yes	yes	no			

Seseli hippomarathrum	*Seseli hippomarathrum*	Jacq.	yes	yes	no			
Seseli libanotis	*Seseli libanotis*	Crantz	yes	yes	no			
Sesleria albicans	*Sesleria caerulea*	Kit. ex Schult.	yes	yes	yes		*Sesleria albicans*	
Sesleria caerulea	*Sesleria caerulea*	Kit. ex Schult.	yes	yes	yes		*Sesleria albicans*	
Sesleria varia agg.	*Sesleria caerulea*	Kit. ex Schult.	yes	yes	yes	aggregate on species level	*Sesleria albicans*	
Setaria italica	*Setaria italica*	(L.) P. Beauv.	yes	yes	yes			
Setaria pumila	*Setaria pumila*	(Poir.) Roem. & Schult.	yes	yes	yes			*Setaria helvola*
Setaria verticillata	*Setaria verticillata*	(L.) P. Beauv.	yes	yes	no			
Setaria verticillata var. ambigua	*Setaria verticillata*	(L.) P. Beauv.	yes	yes	no	aggregate on species level		
Setaria verticillata var. verticillata	*Setaria verticillata*	(L.) P. Beauv.	yes	yes	no	aggregate on species level		
Setaria viridis	*Setaria viridis*	(L.) P. Beauv.	yes	yes	yes			
Setaria viridis ssp. pycnocoma	*Setaria viridis*	(L.) P. Beauv.	yes	yes	yes	aggregate on species level		
Sherardia arvensis	*Sherardia arvensis*	L.	yes	yes	yes			
Silaum silaus	*Silaum silaus*	(L.) Schinz & Thell.	yes	yes	yes			
Silene armeria	*Silene armeria*	L.	yes	yes	no			
Silene conica	*Silene conica*	L.	yes	yes	yes			
Silene coronaria	*Silene coronaria*	(L.) Desr.	yes	yes	no		*Lychnis coronaria*	
Silene dichotoma	*Silene dichotoma*	Ehrh.	yes	yes	yes			

Silene dichotoma subsp. dichotoma	*Silene dichotoma*	Ehrh.	yes	yes	yes	aggregate on species level		
Silene dioica	*Silene dioica*	(L.) Clairv.	yes	yes	yes			
Silene flos-cuculi	*Silene flos-cuculi*	L.	yes	yes	yes		*Lychnis flos-cuculi*	*LYCHNIS FLOS-CUCULI*
Silene flos-jovis	*Silene flos-jovis*	(L.) Desr.	yes	yes	yes			*Lychnis flos-jovis*
Silene gallica	*Silene gallica*	L.	yes	yes	yes			
Silene latifolia	*Silene latifolia*	Poir.	yes	yes	no			
Silene latifolia subsp. alba	*Silene latifolia*	Poir.	yes	yes	no	aggregate on species level		
Silene noctiflora	*Silene noctiflora*	L.	yes	yes	yes			
Silene nutans	*Silene nutans*	L.	yes	yes	yes			
Silene otites	*Silene otites*	(L.) Wibel	yes	yes	yes			
Silene rupestris	*Silene rupestris*	L.	yes	yes	no			
Silene viscaria	*Silene viscaria*	L.	yes	yes	yes		*Lychnis viscaria*	*Viscaria vulgaris*
Silene vulgaris	*Silene vulgaris*	(Moench) Garcke	yes	yes	yes			
Silene vulgaris subsp. glareosa	*Silene vulgaris*	(Moench) Garcke	yes	yes	yes	aggregate on species level		
Silene vulgaris subsp. vulgaris	*Silene vulgaris*	(Moench) Garcke	yes	yes	yes	aggregate on species level		
Silphium perfoliatum	*Silphium perfoliatum*	L.	yes	no	no			
Silybum marianum	*Silybum marianum*	(L.) P. Gaertn.	yes	yes	no			
Sinapis alba	*Sinapis alba*	L.	yes	yes	yes			
Sinapis arvensis	*Sinapis arvensis*	L.	yes	yes	yes			
Sisymbrium altissimum	*Sisymbrium altissimum*	L.	yes	yes	yes			
Sisymbrium austriacum	*Sisymbrium austriacum*	Jacq.	yes	yes	yes			

Sisymbrium loeselii	*Sisymbrium loeselii*	L.	yes	yes	yes			
Sisymbrium officinale	*Sisymbrium officinale*	(L.) Scop.	yes	yes	yes			
Sisymbrium orientale	*Sisymbrium orientale*	L.	yes	yes	yes			
Sisymbrium strictissimum	*Sisymbrium strictissimum*	L.	yes	yes	no			
Sium latifolium	*Sium latifolium*	L.	yes	yes	yes			
Solanum dulcamara	*Solanum dulcamara*	L.	yes	yes	yes			
Solanum nigrum	*Solanum nigrum*	L.	yes	yes	yes			
Solanum nigrum subsp. nigrum	*Solanum nigrum*	L.	yes	yes	yes	aggregate on species level		
Solanum nigrum subsp. schultesii	*Solanum nigrum*	L.	yes	yes	yes	aggregate on species level		
Solanum nigrum var. atriplicifolium	*Solanum nigrum*	L.	yes	yes	yes	aggregate on species level		
Solanum physalifolium	*Solanum physalifolium*	Rusby	yes	yes	no			
Solanum physalifolium var. nitidibaccatum	*Solanum physalifolium*	Rusby	yes	yes	no	aggregate on species level		
Solanum sarachoides	*Solanum sarachoides*	Sendtner	yes	yes	no			
Solanum sarachoides agg.	*Solanum sarachoides*	Sendtner	yes	yes	no	aggregate on species level		
Solanum triflorum	*Solanum triflorum*	Nutt.	yes	yes	no			
Solanum villosum	*Solanum villosum*	Mill.	yes	yes	no			
Solanum villosum subsp. alatum	*Solanum villosum*	Mill.	yes	yes	no	aggregate on species level		
Solanum villosum subsp. villosum	*Solanum villosum*	Mill.	yes	yes	no	aggregate on species level		
Soldanella alpina	*Soldanella alpina*	L.	yes	yes	yes			

Solidago canadensis	*Solidago canadensis*	L.	yes	yes	yes			
Solidago gigantea	*Solidago gigantea*	Aiton	yes	yes	yes			
Solidago graminifolia	*Solidago graminifolia*	(L.) Salisb.	yes	yes	yes			*Euthamia graminifolia*
Solidago virgaurea	*Solidago virgaurea*	L.	yes	yes	yes			
Solidago virgaurea subsp. minuta	*Solidago virgaurea*	L.	yes	yes	yes	aggregate on species level		
Solidago virgaurea subsp. virgaurea	*Solidago virgaurea*	L.	yes	yes	yes	aggregate on species level		
Sonchus arvensis	*Sonchus arvensis*	L.	yes	yes	yes			
Sonchus arvensis subsp. arvensis	*Sonchus arvensis*	L.	yes	yes	yes	aggregate on species level		
Sonchus arvensis subsp. uliginosus	*Sonchus arvensis*	(M. Bieb.) Nyman	yes	yes	yes	aggregate on species level		
Sonchus asper	*Sonchus asper*	(L.) Hill	yes	yes	yes			
Sonchus asper subsp. asper	*Sonchus asper*	(L.) Hill	yes	yes	yes	aggregate on species level		
Sonchus asper subsp. glaucescens	*Sonchus asper*	(Jord.) Ball	yes	yes	yes	aggregate on species level		
Sonchus oleraceus	*Sonchus oleraceus*	L.	yes	yes	yes			
Sorbus aria	*Sorbus aria*	(L.) Crantz	yes	yes	yes			
Sorbus aria agg.	*Sorbus aria*	(L.) Crantz	yes	yes	yes	aggregate on species level		
Sorbus aucuparia	*Sorbus aucuparia*	L.	yes	yes	yes			

Sorbus aucuparia subsp. aucuparia	*Sorbus aucuparia*	L.	yes	yes	yes	aggregate on species level		
Sorbus aucuparia subsp. glabrata	*Sorbus aucuparia*	L.	yes	yes	yes	aggregate on species level		
Sorbus badensis	*Sorbus latifolia*	Duell	yes	yes	no	Durka and Michalski 2012		
Sorbus decipiens	*Sorbus latifolia*	(Bechst.) Irmisch ex Petz. & Kirchn.	yes	yes	no	Durka and Michalski 2012		
Sorbus domestica	*Sorbus domestica*	L.	yes	yes	yes			
Sorbus dubronensis	*Sorbus aria*	(L.) Crantz	yes	yes	yes	Flora Germanica / Hassler 2024 World Plants (https://www.worldplants.de/world-plants-complete-list/complete-plant-list#plantUid-196463)		
Sorbus graeca	*Sorbus aria*	(L.) Crantz	yes	yes	yes	Durka and Michalski 2012		

Sorbus herbipolitana	*Sorbus latifolia*	Meierott	yes	yes	no	http://blumeninschwaben.de/Zweikeimblaettrige/Rosengewaechse/latifolia_agg.htm			
Sorbus hybrida agg.	*Sorbus aria*	(L.) Crantz	yes	yes	yes	Durka and Michalski 2012			
Sorbus intermedia	*Sorbus intermedia*	(L.) Crantz	yes	yes	yes	Durka and Michalski 2012			
Sorbus latifolia	*Sorbus latifolia*	(Lam.) Pers.	yes	yes	no				
Sorbus latifolia agg.	*Sorbus latifolia*	(Lam.) Pers.	yes	yes	no	aggregate on species level			
Sorbus lonetalensis	*Sorbus aria*	(L.) Crantz	yes	yes	yes				
Sorbus meyeri	*Sorbus aria*	(L.) Crantz	yes	yes	yes				
Sorbus moenofranconica	*Sorbus aria*	(L.) Crantz	yes	yes	yes				
Sorbus mougeotii	*Sorbus mougeotii*	Soy.-Will. & Godr.	yes	yes	no				
Sorbus mougeotii agg.	*Sorbus mougeotii*	Soy.-Will. & Godr.	yes	yes	no	aggregate on species level			
Sorbus pannonica	*Sorbus aria*	(L.) Crantz	yes	yes	yes	Durka and Michalski 2012			

Sorbus pseudothuringiaca	*Sorbus mougeotii*	Soy.-Will. & Godr.	yes	yes	no	Durka and Michalski 2012		
Sorbus puellarum	*Sorbus latifolia*	(Lam.) Pers.	yes	yes	no	http://blumeninschwaben.de/Zweikeimblaettrige/Rosengewaechse/latifolia_agg.htm		
Sorbus seyboldiana	*Sorbus aria*	(L.) Crantz	yes	yes	yes			
Sorbus torminalis	*Sorbus torminalis*	(L.) Crantz	yes	yes	yes			
Sorghum bicolor	*Sorghum bicolor*	(L.) Moench	yes	yes	no			
Sorghum halepense	*Sorghum halepense*	(L.) Pers.	yes	yes	yes			
Sorghum halepense var. muticum	*Sorghum halepense*	(L.) Pers.	yes	yes	yes	aggregate on species level		
Sparganium angustifolium	*Sparganium angustifolium*	Michx.	yes	yes	no			
Sparganium emersum	*Sparganium emersum*	Rehmann	yes	yes	no			
Sparganium erectum	*Sparganium erectum*	L.	yes	yes	no			
Sparganium erectum subsp. erectum	*Sparganium erectum*	L.	yes	yes	no	aggregate on species level		
Sparganium erectum subsp. microcarpum	*Sparganium erectum*	L.	yes	yes	no	aggregate on species level		
Sparganium erectum subsp. neglectum	*Sparganium erectum*	L.	yes	yes	no	aggregate on species level		

Sparganium erectum subsp. oocarpum	*Sparganium erectum*	L.	yes	yes	no	aggregate on species level		
Sparganium natans	*Sparganium natans*	Wallr.	yes	yes	no			
Spergula arvensis	*Spergula arvensis*	L.	yes	yes	yes			
Spergula arvensis subsp. arvensis	*Spergula arvensis*	L.	yes	yes	yes	aggregate on species level		
Spergula morisonii	*Spergula morisonii*	Boreau	yes	yes	yes			
Spergula pentandra	*Spergula pentandra*	L.	yes	yes	no			
Spergularia media	*Spergularia media*	(L.) C. Presl	yes	yes	yes			
Spergularia rubra	*Spergularia rubra*	(L.) J. Presl & C. Presl	yes	yes	yes			
Spergularia salina	*Spergularia salina*	J. Presl & C. Presl	yes	yes	yes			
Spiraea billardii	*Spiraea billardii*		yes	yes	no		*Spiraea x billardii*	
Spiraea chamaedryfolia	*Spiraea chamaedryfolia*	L.	yes	yes	no			
Spiraea douglasii	*Spiraea douglasii*	Hook.	yes	yes	no			
Spiraea japonica	*Spiraea japonica*	L.f.	yes	yes	no			
Spiraea media	*Spiraea media*	F. Schmidt	yes	yes	yes			
Spiraea salicifolia	*Spiraea salicifolia*	L.	yes	yes	no			
Spiraea salicifolia agg.	*Spiraea salicifolia*	L.	yes	yes	no	aggregate on species level		
Spiranthes aestivalis	*Spiranthes aestivalis*	(Poir.) Rich.	yes	yes	no			
Spiranthes spiralis	*Spiranthes spiralis*	(L.) Chevall.	yes	yes	no			
Spirodela polyrhiza	*Spirodela polyrhiza*	(L.) Schleid.	yes	yes	no			
Stachys alpina	*Stachys alpina*	L.	yes	yes	no			
Stachys annua	*Stachys annua*	(L.) L.	yes	yes	no			
Stachys arvensis	*Stachys arvensis*	(L.) L.	yes	yes	yes			

Stachys byzantina	*Stachys byzantina*	K. Koch	yes	yes	no			
Stachys germanica	*Stachys germanica*	L.	yes	yes	yes			
Stachys palustris	*Stachys palustris*	L.	yes	yes	yes			
Stachys recta	*Stachys recta*	L.	yes	yes	yes			
Stachys recta subsp. recta	*Stachys recta*	L.	yes	yes	yes	aggregate on species level		
Stachys sylvatica	*Stachys sylvatica*	L.	yes	yes	yes			
Staphylea pinnata	*Staphylea pinnata*	L.	yes	yes	yes			
Stellaria alsine	*Stellaria alsine*	Grimm	yes	yes	yes			
Stellaria aquatica	*Stellaria aquatica*	(L.) Moench	yes	yes	yes		*Myosoton aquaticum*	
Stellaria graminea	*Stellaria graminea*	L.	yes	yes	yes			
Stellaria holostea	*Stellaria holostea*	L.	yes	yes	yes			
Stellaria media	*Stellaria media*	(L.) Vill.	yes	yes	yes			
Stellaria media agg.	*Stellaria media*	(L.) Vill.	yes	yes	yes	aggregate on species level		
Stellaria neglecta	*Stellaria media*	(L.) Vill.	yes	yes	yes	part of S. media-complex, taxonomy difficult, therefore aggregate on species level		
Stellaria nemorum	*Stellaria nemorum*	L.	yes	yes	yes			
Stellaria nemorum subsp. montana	*Stellaria nemorum*	L.	yes	yes	yes	aggregate on species level		

Stellaria nemorum subsp. nemorum	*Stellaria nemorum*	L.	yes	yes	yes	aggregate on species level		
Stellaria pallida	*Stellaria media*	(L.) Vill.	yes	yes	yes	part of S. media-complex, taxonomy difficult, therefore aggregate on species level		
Stellaria palustris	*Stellaria palustris*	Ehrh. ex Hoffm.	yes	yes	yes			
Stipa calamagrostis	*Stipa calamagrostis*	(L.) Wahlenb.	yes	yes	no		*Achnatherum calamagrostis*	
Stipa capillata	*Stipa capillata*	L.	yes	yes	yes			
Stipa eriocaulis	*Stipa eriocaulis*	Borbás	yes	yes	no			
Stipa eriocaulis subsp. austriaca	*Stipa eriocaulis*	Borbás	yes	yes	no	aggregate on species level		
Stipa eriocaulis subsp. lutetiana	*Stipa eriocaulis*	Borbás	yes	yes	no	aggregate on species level		
Stipa pennata	*Stipa pennata*	Celak.	yes	yes	yes			
Stipa pennata agg.	*Stipa pennata*	Celak.	yes	yes	yes	aggregate on species level		
Stratiotes aloides	*Stratiotes aloides*	L.	yes	yes	no			
Streptopus amplexifolius	*Streptopus amplexifolius*	(L.) DC.	yes	yes	yes			
Succisa pratensis	*Succisa pratensis*	Moench	yes	yes	yes			
Swertia perennis	*Swertia perennis*	L.	yes	yes	yes			

Swertia perennis subsp. alpestris	*Swertia perennis*	L.	yes	yes	yes	aggregate on species level		
Symphoricarpos albus	*Symphoricarpos albus*	Suksd.	yes	yes	yes			
Symphytum asperum	*Symphytum asperum*	Lepech.	yes	yes	no			
Symphytum asperum agg.	*Symphytum asperum*	Lepech.	yes	yes	no	aggregate on species level		
Symphytum bulbosum	*Symphytum bulbosum*	Schimp.	yes	yes	no			
Symphytum officinale	*Symphytum officinale*	L.	yes	yes	yes			
Symphytum officinale agg.	*Symphytum officinale*	L.	yes	yes	yes	aggregate on species level		
Symphytum officinale subsp. bohemicum	*Symphytum officinale*	L.	yes	yes	yes	aggregate on species level		
Symphytum officinale subsp. officinale	*Symphytum officinale*	L.	yes	yes	yes	aggregate on species level		
Symphytum officinale subsp. uliginosum	*Symphytum officinale*	L.	yes	yes	yes	aggregate on species level		
Symphytum uplandicum	*Symphytum uplandicum*	Nyman	yes	yes	no		*Symphytum x uplandicum*	
Syringa vulgaris	*Syringa vulgaris*	L.	yes	yes	yes			
Tamus communis	*Tamus communis*	L.	yes	yes	yes			*Dioscorea communis*
Tanacetum corymbosum	*Tanacetum corymbosum*	(L.) Sch. Bip.	yes	yes	yes			
Tanacetum corymbosum	*Tanacetum corymbosum*	(L.) Sch. Bip.	yes	yes	yes	aggregate on species level		
Tanacetum parthenium	*Tanacetum parthenium*	(L.) Sch. Bip.	yes	yes	yes			

Tanacetum vulgare	*Tanacetum vulgare*	L.	yes	yes	yes			
Taraxacum sect. Ruderalia	*Taraxacum sect. Ruderalia*		yes	yes	no	Buttler et al 2018		
Taraxacum acervatulum	*Taraxacum sect. Ruderalia*		yes	yes	no	Buttler et al 2018		
Taraxacum aequilobum	*Taraxacum sect. Ruderalia*		yes	yes	no	Buttler et al 2018		
Taraxacum aganophytum	*Taraxacum sect. Ruderalia*		yes	yes	no	Buttler et al 2018		
Taraxacum albulense	*Taraxacum sect. Alpestria*		yes	yes	no	Buttler et al 2018	Kumar et al 2022, added to genus Taraxacum	
Taraxacum atactum	*Taraxacum sect. Hamata*		yes	yes	no	Buttler et al 2018	Kumar et al 2022, added to genus Taraxacum	

Taraxacum austriacum	*Taraxacum sect. Erythrosperma*		yes	yes	no	https://europlusmed.org/cdm_dataportal/taxon/6208fe76-ae78-428d-ba24-d5b653dae8dc/synonymy?highlite=d5649510-0cd3-43c4-9ad8-bc27da77f2d9&acceptedFor=d5649510-0cd3-43c4-		
Taraxacum austrinum	*Taraxacum sect. Palustria*		yes	yes	no	Buttler et al 2018		
Taraxacum baeckiiforme	*Taraxacum sect. Ruderalia*		yes	yes	no	Buttler et al 2018		
Taraxacum balticiforme	*Taraxacum sect. Palustria*		yes	yes	no	Buttler et al 2018		
Taraxacum bellicum	*Taraxacum sect. Erythrosperma*		yes	yes	no	Buttler et al 2018		
Taraxacum brachyglossum	*Taraxacum sect. Erythrosperma*		yes	yes	no	Buttler et al 2018		
Taraxacum brevisectoides	*Taraxacum sect. Ruderalia*		yes	yes	no	Buttler et al 2018		
Taraxacum caloschistoides	*Taraxacum sect. Ruderalia*		yes	yes	no	Buttler et al 2018		

Taraxacum clemens	*Taraxacum sect. Erythrosperma*		yes	yes	no	https://europlusmed.org/cdm_dataportal/taxon/519e524c-ade2-400c-b397-d8cf812f488d		
Taraxacum concinnum	*Taraxacum sect. Ruderalia*		yes	yes	no	Buttler et al 2018		
Taraxacum demotes	*Taraxacum sect. Ruderalia*		yes	yes	no	Buttler et al 2018		
Taraxacum disseminatum	*Taraxacum sect. Erythrosperma*		yes	yes	no	Buttler et al 2018		
Taraxacum duplidentifrons	*Taraxacum sect. Celtica*		yes	yes	no	Durka and Michalski 2012		
Taraxacum fasciatum	*Taraxacum sect. Ruderalia*		yes	yes	no	Buttler et al 2018		
Taraxacum franconicum	*Taraxacum sect. Erythrosperma*		yes	yes	no	Buttler et al 2018		
Taraxacum gelertii	*Taraxacum sect. Celtica*		yes	yes	no	Durka and Michalski 2012		
Taraxacum guttigestans	*Taraxacum sect. Ruderalia*		yes	yes	no	Buttler et al 2018		
Taraxacum haematicum	*Taraxacum sect. Ruderalia*		yes	yes	no	Buttler et al 2018		

Taraxacum hamatiforme	*Taraxacum sect. Hamata*		yes	yes	no	Buttler et al 2018	Kumar et al 2022, added to genus Taraxacum		
Taraxacum hamatum	*Taraxacum sect. Hamata*		yes	yes	no	Buttler et al 2018	Kumar et al 2022, added to genus Taraxacum		
Taraxacum heleonastes	*Taraxacum sect. Palustria*		yes	yes	no	Buttler et al 2018			
Taraxacum hepaticum	*Taraxacum sect. Ruderalia*		yes	yes	no	Buttler et al 2018			
Taraxacum hollandicum	*Taraxacum sect. Hamata*		yes	yes	no	Buttler et al 2018			
Taraxacum lacinulatum	*Taraxacum sect. Ruderalia*		yes	yes	no	Buttler et al 2018			
Taraxacum lacistophylloides	*Taraxacum sect. Erythrosperma*		yes	yes	no	Buttler et al 2018			
Taraxacum lacistophyllum	*Taraxacum sect. Erythrosperma*		yes	yes	no	Buttler et al 2018			
Taraxacum laevigatum	*Taraxacum sect. Erythrosperma*		yes	yes	no	Buttler et al 2018			
Taraxacum laticordatum	*Taraxacum sect. Ruderalia*		yes	yes	no	Buttler et al 2018			
Taraxacum leontodontoides	*Taraxacum sect. Ruderalia*		yes	yes	no	Buttler et al 2018			
Taraxacum lojoense	*Taraxacum sect. Ruderalia*		yes	yes	no	Buttler et al 2018			
Taraxacum madidum	*Taraxacum sect. Palustria*		yes	yes	no	Buttler et al 2018			

Taraxacum melanostigma	*Taraxacum sect. Ruderalia*		yes	yes	no	Buttler et al 2018		
Taraxacum multilepis	*Taraxacum sect. Palustria*		yes	yes	no	Buttler et al 2018		
Taraxacum neoaellenii	*Taraxacum sect. Ruderalia*		yes	yes	no	Buttler et al 2018		
Taraxacum nordstedtii	*Taraxacum sect. Celtica*		yes	yes	no	Buttler et al 2018		
Taraxacum officinale	*Taraxacum sect. Ruderalia*		yes	yes	no	Buttler et al 2018		
Taraxacum officinale agg.	*Taraxacum sect. Ruderalia*		yes	yes	no	Buttler et al 2018		
Taraxacum pallescentiforme	*Taraxacum sect. Ruderalia*		yes	yes	no	Buttler et al 2018		
Taraxacum paradoxachrum	*Taraxacum sect. Ruderalia*		yes	yes	no	Buttler et al 2018		
Taraxacum paucijugum	*Taraxacum sect. Ruderalia*		yes	yes	no	Buttler et al 2018		
Taraxacum pauckertianum	*Taraxacum sect. Palustria*		yes	yes	no	Buttler et al 2018		
Taraxacum perfissum	*Taraxacum sect. Alpestria*		yes	yes	no	Buttler et al 2018	Kumar et al 2022, added to genus Taraxacum	
Taraxacum pittochromatum	*Taraxacum sect. Ruderalia*		yes	yes	no	Buttler et al 2018		
Taraxacum plicatiangulatum	*Taraxacum sect. Ruderalia*		yes	yes	no	Buttler et al 2018		
Taraxacum plumbeum	*Taraxacum sect. Erythrosperma*		yes	yes	no	Buttler et al 2018		
Taraxacum porrigens	*Taraxacum sect. Ruderalia*		yes	yes	no	Buttler et al 2018		

Taraxacum privum	*Taraxacum sect. Ruderalia*		yes	yes	no	Buttler et al 2018		
Taraxacum prunicolor	*Taraxacum sect. Erythrosperma*		yes	yes	no	Buttler et al 2018		
Taraxacum pseudohabile	*Taraxacum sect. Ruderalia*		yes	yes	no	Buttler et al 2018		
Taraxacum pulchrifolium	*Taraxacum sect. Ruderalia*		yes	yes	no	Buttler et al 2018		
Taraxacum quadrangulum	*Taraxacum sect. Ruderalia*		yes	yes	no	Buttler et al 2018		
Taraxacum rubicundum	*Taraxacum sect. Erythrosperma*		yes	yes	no	Buttler et al 2018		
Taraxacum sahlinii	*Taraxacum sect. Ruderalia*		yes	yes	no	Buttler et al 2018		
Taraxacum scanicum	*Taraxacum sect. Erythrosperma*		yes	yes	no	Buttler et al 2018		
Taraxacum sect. Celtica	*Taraxacum sect. Celtica*		yes	yes	no	Buttler et al 2018		
Taraxacum sect. Erythrosperma	*Taraxacum sect. Erythrosperma*		yes	yes	no	Buttler et al 2018		
Taraxacum sect. Hamata	*Taraxacum sect. Hamata*		yes	yes	no	Buttler et al 2018	Kumar et al 2022, added to genus Taraxacum	
Taraxacum sect. Obliqua	*Taraxacum sect. Obliqua*		yes	yes	no	Buttler et al 2018		
Taraxacum sect. Palustria	*Taraxacum sect. Palustria*		yes	yes	no	Buttler et al 2018		
Taraxacum sect. Ruderalia	*Taraxacum sect. Ruderalia*		yes	yes	no	Buttler et al 2018		
Taraxacum selenoides	*Taraxacum sect. Ruderalia*		yes	yes	no	Buttler et al 2018		

Taraxacum sertatum	*Taraxacum sect. Ruderalia*		yes	yes	no	Buttler et al 2018		
Taraxacum silesiacum	*Taraxacum sect. Erythrosperma*		yes	yes	no	Buttler et al 2018		
Taraxacum subcanescens	*Taraxacum sect. Ruderalia*		yes	yes	no	Buttler et al 2018		
Taraxacum subsaxenii	*Taraxacum sect. Ruderalia*		yes	yes	no	Buttler et al 2018		
Taraxacum tanyolobum	*Taraxacum sect. Erythrosperma*		yes	yes	no	Buttler et al 2018		
Taraxacum tenuilobum	*Taraxacum sect. Erythrosperma*		yes	yes	no	Buttler et al 2018		
Taraxacum tortilobum	*Taraxacum sect. Erythrosperma*		yes	yes	no	Buttler et al 2018		
Taraxacum trilobifolium	*Taraxacum sect. Palustria*		yes	yes	no	Buttler et al 2018		
Taraxacum turfosum	*Taraxacum sect. Palustria*		yes	yes	no	Buttler et al 2018		
Taraxacum udum	*Taraxacum sect. Palustria*		yes	yes	no	Buttler et al 2018		
Taxus baccata	*Taxus baccata*	L.	yes	yes	yes			
Teesdalia nudicaulis	*Teesdalia nudicaulis*	(L.) R. Br.	yes	yes	yes			
Telekia speciosa	*Telekia speciosa*	(Schreb.) Baumg.	yes	yes	no			
Tephroseris helenitis	*Tephroseris helenitis*	(L.) B. Nord.	yes	yes	no			
Tephroseris helenitis subsp. helenitis	*Tephroseris helenitis*	(L.) B. Nord.	yes	yes	no	aggregate on species level		
Tetragonolobus maritimus	*Tetragonolobus maritimus*	(L.) Roth	yes	yes	yes			*Lotus maritimus*
Teucrium botrys	*Teucrium botrys*	L.	yes	yes	yes			
Teucrium chamaedrys	*Teucrium chamaedrys*	L.	yes	yes	yes			

Teucrium chamaedrys subsp. chamaedrys	*Teucrium chamaedrys*	L.	yes	yes	yes	aggregate on species level		
Teucrium chamaedrys subsp. germanicum	*Teucrium chamaedrys*	L.	yes	yes	yes	aggregate on species level		
Teucrium montanum	*Teucrium montanum*	L.	yes	yes	yes			
Teucrium scordium	*Teucrium scordium*	L.	yes	yes	yes			
Teucrium scorodonia	*Teucrium scorodonia*	L.	yes	yes	yes			
Teucrium scorodonia subsp. scorodonia	*Teucrium scorodonia*	L.	yes	yes	yes	aggregate on species level		
Thalictrum aquilegiifolium	*Thalictrum aquilegiifolium*	L.	yes	yes	yes			
Thalictrum flavum	*Thalictrum flavum*	L.	yes	yes	yes			
Thalictrum minus	*Thalictrum minus*	L.	yes	yes	yes			
Thalictrum minus agg.	*Thalictrum minus*	L.	yes	yes	yes	aggregate on species level		
Thalictrum minus subsp. majus	*Thalictrum minus*	L.	yes	yes	yes	aggregate on species level		
Thalictrum minus subsp. minus	*Thalictrum minus*	L.	yes	yes	yes	aggregate on species level		
Thalictrum minus subsp. pratense	*Thalictrum minus*	L.	yes	yes	yes	aggregate on species level		
Thalictrum minus subsp. saxatile	*Thalictrum minus*	L.	yes	yes	yes	aggregate on species level		
Thalictrum simplex	*Thalictrum simplex*	L.	yes	yes	yes			
Thalictrum simplex subsp. galioides	*Thalictrum simplex*	L.	yes	yes	yes	aggregate on species level		

Thalictrum simplex subsp. tenuifolium	*Thalictrum simplex*	L.	yes	yes	yes	aggregate on species level				
Thelypteris palustris	*Thelypteris palustris*	Schott	yes	yes	no					
Thesium bavarum	*Thesium bavarum*	Schrank	yes	yes	yes					
Thesium linophyllon	*Thesium linophyllon*	L.	yes	yes	yes					
Thesium pyrenaicum	*Thesium pyrenaicum*	Pourr.	yes	yes	yes					
Thesium pyrenaicum subsp. pyrenaicum	*Thesium pyrenaicum*	Pourr.	yes	yes	yes	aggregate on species level				
Thesium rostratum	*Thesium rostratum*	Mert. & W. D. J. Koch	yes	yes	no					
Thlaspi alliaceum	*Thlaspi alliaceum*	L.	yes	yes	yes			*Mummenhoffia alliacea*		
Thlaspi arvense	*Thlaspi arvense*	L.	yes	yes	yes					
Thlaspi caerulescens	*Thlaspi caerulescens*	J. Presl & C. Presl	yes	yes	yes			*Noccaea caerulescens*		
Thlaspi erraticum	*Thlaspi perfoliatum*	Jord.	yes	yes	yes	taxonomic inconsistencies between first and second mapping		*Microthlaspi erraticum*		
Thlaspi montanum	*Thlaspi montanum*	L.	yes	yes	yes			*Noccaea montana*		
Thlaspi perfoliatum	*Thlaspi perfoliatum*	L.	yes	yes	yes			*Noccaea perfoliata*		
Thymelaea passerina	*Thymelaea passerina*	(L.) Coss. & Germ.	yes	yes	no					

Thymus praecox	*Thymus praecox*	Opiz	yes	yes	yes			
Thymus praecox agg.	*Thymus praecox*	Opiz	yes	yes	yes	aggregate on species level		
Thymus praecox subsp. arcticus	*Thymus praecox*	Opiz	yes	yes	yes	aggregate on species level		
Thymus praecox subsp. polytrichus	*Thymus praecox*	Opiz	yes	yes	yes	aggregate on species level		
Thymus praecox subsp. praecox	*Thymus praecox*	Opiz	yes	yes	yes	aggregate on species level		
Thymus pulegioides	*Thymus pulegioides*	L.	yes	yes	yes			
Thymus pulegioides agg.	*Thymus pulegioides*	L.	yes	yes	yes	aggregate on species level		
Thymus pulegioides subsp. carniolicus	*Thymus pulegioides*	L.	yes	yes	yes	aggregate on species level		
Thymus pulegioides subsp. pulegioides	*Thymus pulegioides*	L.	yes	yes	yes	aggregate on species level		
Thymus pulegioides var. praeflorens	*Thymus pulegioides*	L.	yes	yes	yes	aggregate on species level		
Thymus pulegioides var. pulegioides	*Thymus pulegioides*	L.	yes	yes	yes	aggregate on species level		
Thymus serpyllum	*Thymus serpyllum*	L.	yes	yes	yes			
Thymus serpyllum agg.	*Thymus serpyllum*	L.	yes	yes	yes	aggregate on species level		
Thymus vulgaris	*Thymus vulgaris*	L.	yes	yes	yes			
Tilia cordata	*Tilia cordata*	Mill.	yes	yes	yes			
Tilia platyphyllos	*Tilia platyphyllos*	Scop.	yes	yes	yes			

Tilia platyphyllos subsp. cordifolia	*Tilia platyphyllos*	Scop.	yes	yes	yes	aggregate on species level		
Tilia platyphyllos subsp. platyphyllos	*Tilia platyphyllos*	Scop.	yes	yes	yes	aggregate on species level		
Tofieldia calyculata	*Tofieldia calyculata*	(L.) Wahlenb.	yes	yes	yes			
Torilis arvensis	*Torilis arvensis*	(Huds.) Link	yes	yes	yes			
Torilis arvensis subsp. arvensis	*Torilis arvensis*	(Huds.) Link	yes	yes	yes	aggregate on species level		
Torilis japonica	*Torilis japonica*	(Houtt.) DC.	yes	yes	yes			
Torilis nodosa	*Torilis nodosa*	(L.) P. Gaertn.	yes	yes	yes			
Tragopogon dubius	*Tragopogon dubius*	Scop.	yes	yes	yes			
Tragopogon pratensis	*Tragopogon pratensis*	L.	yes	yes	yes			
Tragopogon pratensis agg.	*Tragopogon pratensis*	NA	yes	yes	yes	aggregate on species level		
Tragopogon pratensis subsp. grandiflorus	*Tragopogon pratensis*	L.	yes	yes	yes	aggregate on species level		
Tragopogon pratensis subsp. minor	*Tragopogon pratensis*	L.	yes	yes	yes	aggregate on species level		
Tragopogon pratensis subsp. orientalis	*Tragopogon pratensis*	L.	yes	yes	yes	aggregate on species level		
Tragopogon pratensis subsp. pratensis	*Tragopogon pratensis*	L.	yes	yes	yes	aggregate on species level		
Tragus racemosus	*Tragus racemosus*	(L.) All.	yes	yes	yes			
Trapa natans	*Trapa natans*	L.	yes	yes	no			
Traunsteinera globosa	*Traunsteinera globosa*	(L.) Rchb.	yes	yes	no			

Trichomanes speciosum	*Trichomanes speciosum*	Willd.	yes	yes	no			
Trichophorum alpinum	*Trichophorum alpinum*	(L.) Pers.	yes	yes	yes			
Trichophorum cespitosum	*Trichophorum cespitosum*	(L.) Hartm.	yes	yes	no			
Trichophorum cespitosum agg.	*Trichophorum cespitosum*	(L.) Hartm.	yes	yes	no	aggregate on species level		
Trichophorum cespitosum subsp. cespitosum	*Trichophorum cespitosum*	(L.) Hartm.	yes	yes	no	aggregate on species level		
Trichophorum cespitosum subsp. germanicum	*Trichophorum cespitosum*	(L.) Hartm.	yes	yes	no	aggregate on species level		
Trientalis europaea	*Trientalis europaea*	L.	yes	yes	yes			
Trifolium alexandrinum	*Trifolium alexandrinum*	L.	yes	yes	no			
Trifolium alpestre	*Trifolium alpestre*	L.	yes	yes	yes			
Trifolium arvense	*Trifolium arvense*	L.	yes	yes	yes			
Trifolium arvense subsp. arvense	*Trifolium arvense*	L.	yes	yes	yes	aggregate on species level		
Trifolium aureum	*Trifolium aureum*	Pollich	yes	yes	yes			
Trifolium campestre	*Trifolium campestre*	Schreb.	yes	yes	yes			
Trifolium dubium	*Trifolium dubium*	Sibth.	yes	yes	yes			
Trifolium dubium agg.	*Trifolium dubium*	Sibth.	yes	yes	yes	aggregate on species level		
Trifolium fragiferum	*Trifolium fragiferum*	L.	yes	yes	yes			
Trifolium fragiferum subsp. fragiferum	*Trifolium fragiferum*	L.	yes	yes	yes	aggregate on species level		
Trifolium hybridum	*Trifolium hybridum*	L.	yes	yes	yes			

Trifolium hybridum subsp. elegans	*Trifolium hybridum*	L.	yes	yes	yes	aggregate on species level		
Trifolium hybridum subsp. hybridum	*Trifolium hybridum*	L.	yes	yes	yes	aggregate on species level		
Trifolium incarnatum	*Trifolium incarnatum*	L.	yes	yes	yes			
Trifolium medium	*Trifolium medium*	L.	yes	yes	yes			
Trifolium montanum	*Trifolium montanum*	L.	yes	yes	yes			
Trifolium ochroleucon	*Trifolium ochroleucon*	Huds.	yes	yes	no			
Trifolium patens	*Trifolium patens*	Schreb.	yes	yes	no			
Trifolium pratense	*Trifolium pratense*	L.	yes	yes	yes			
Trifolium pratense subsp. expansum	*Trifolium pratense*	L.	yes	yes	yes	aggregate on species level		
Trifolium pratense subsp. pratense	*Trifolium pratense*	L.	yes	yes	yes	aggregate on species level		
Trifolium pratense subsp. sativum	*Trifolium pratense*	L.	yes	yes	yes	aggregate on species level		
Trifolium repens	*Trifolium repens*	L.	yes	yes	yes			
Trifolium repens subsp. prostratum	*Trifolium repens*	L.	yes	yes	yes	aggregate on species level		
Trifolium repens subsp. repens	*Trifolium repens*	L.	yes	yes	yes	aggregate on species level		
Trifolium resupinatum	*Trifolium resupinatum*	L.	yes	yes	yes			
Trifolium rubens	*Trifolium rubens*	L.	yes	yes	yes			
Trifolium scabrum	*Trifolium scabrum*	L.	yes	yes	no			
Trifolium spadiceum	*Trifolium spadiceum*	L.	yes	yes	yes			
Trifolium striatum	*Trifolium striatum*	L.	yes	yes	yes			

Triglochin palustris	*Triglochin palustris*	L.	yes	yes	yes		*Triglochin palustre*	
Trinia glauca	*Trinia glauca*	(L.) Dumort.	yes	yes	yes			
Tripleurospermum perforatum	*Tripleurospermum perforatum*	(K. Koch) Sch. Bip.	yes	yes	yes			*Tripleurospermum inodorum*
Trisetum flavescens	*Trisetum flavescens*	(L.) P. Beauv.	yes	yes	yes			
Trisetum flavescens agg.	*Trisetum flavescens*	(L.) P. Beauv.	yes	yes	yes	aggregate on species level		
Trisetum flavescens subsp. flavescens	*Trisetum flavescens*	(L.) P. Beauv.	yes	yes	yes	aggregate on species level		
Trollius europaeus	*Trollius europaeus*	L.	yes	yes	yes			
Trollius europaeus subsp. europaeus	*Trollius europaeus*	L.	yes	yes	yes	aggregate on species level		
Tsuga canadensis	*Tsuga canadensis*	(L.) Carr.	yes	yes	yes			
Tulipa gesnerana agg.	*Tulipa gesneriana*	L.	yes	yes	no	aggregate on species level		
Tulipa gesneriana	*Tulipa gesneriana*	L.	yes	yes	no			
Tulipa sylvestris	*Tulipa sylvestris*	L.	yes	yes	no			
Tulipa sylvestris subsp. sylvestris	*Tulipa sylvestris*	L.	yes	yes	no	aggregate on species level		
Turgenia latifolia	*Turgenia latifolia*	(L.) Hoffm.	yes	yes	yes			
Tussilago farfara	*Tussilago farfara*	L.	yes	yes	yes			
Typha angustifolia	*Typha angustifolia*	L.	yes	yes	yes			
Typha latifolia	*Typha latifolia*	L.	yes	yes	yes			
Typha laxmannii	*Typha laxmannii*	Lepech.	yes	yes	no			
Typha minima	*Typha minima*	Funck	yes	yes	no			

Typha shuttleworthii	*Typha shuttleworthii*	W. D. J. Koch & Sond.	yes	yes	no			
Ulex europaeus	*Ulex europaeus*	L.	yes	yes	yes			
Ulmus glabra	*Ulmus glabra*	Huds.	yes	yes	yes			
Ulmus laevis	*Ulmus laevis*	Pall.	yes	yes	yes			
Ulmus minor	*Ulmus minor*	Mill.	yes	yes	yes			
Ulmus minor agg.	*Ulmus minor*	Mill.	yes	yes	yes	aggregate on species level		
Urtica dioica	*Urtica dioica*	L.	yes	yes	yes			
Urtica dioica agg.	*Urtica dioica*	L.	yes	yes	yes	aggregate on species level		
Urtica dioica subsp. dioica	*Urtica dioica*	L.	yes	yes	yes	aggregate on species level		
Urtica dioica subsp. subinermis	*Urtica dioica*	L.	yes	yes	yes	aggregate on species level		
Urtica urens	*Urtica urens*	L.	yes	yes	yes			
Utricularia australis	*Utricularia australis*	R. Br.	yes	yes	no			
Utricularia australis R. Br. und U. vulgaris L.	*Utricularia australis*	R. Br.	yes	yes	no	aggregate on species level		
Utricularia intermedia	*Utricularia intermedia*	Hayne	yes	yes	no			
Utricularia intermedia agg.	*Utricularia intermedia*	Hayne	yes	yes	no	aggregate on species level		
Utricularia minor	*Utricularia minor*	L.	yes	yes	no			
Utricularia minor agg.	*Utricularia minor*	L.	yes	yes	no	aggregate on species level		
Utricularia ochroleuca	*Utricularia ochroleuca*	R. W. Hartm.	yes	yes	no			
Utricularia ochroleuca R.W. Hartm. und U. stygia G. Thor.	*Utricularia ochroleuca*	R. W. Hartm.	yes	yes	no	aggregate on species level		

Utricularia stygia	*Utricularia stygia*	G. Thor	yes	yes	no			
Utricularia vulgaris	*Utricularia vulgaris*	L.	yes	yes	no			
Utricularia vulgaris agg.	*Utricularia vulgaris*	L.	yes	yes	no	aggregate on species level		
Vaccaria hispanica	*Vaccaria hispanica*	(Mill.) Rauschert	yes	yes	yes			
Vaccaria hispanica subsp. hispanica	*Vaccaria hispanica*	(Mill.) Rauschert	yes	yes	yes	aggregate on species level		
Vaccinium macrocarpon	*Vaccinium macrocarpon*	(Aiton) Pursh	yes	yes	yes			
Vaccinium myrtillus	*Vaccinium myrtillus*	L.	yes	yes	yes			
Vaccinium oxycoccos	*Vaccinium oxycoccos*	L.	yes	yes	yes			
Vaccinium oxycoccos agg.	*Vaccinium oxycoccos*	NA	yes	yes	yes	aggregate on species level		
Vaccinium uliginosum	*Vaccinium uliginosum*	L.	yes	yes	yes			
Vaccinium uliginosum subsp. uliginosum	*Vaccinium uliginosum*	L.	yes	yes	yes	aggregate on species level		
Vaccinium vitis-idaea	*Vaccinium vitis-idaea*	L.	yes	yes	yes			*VACCINIUM VITIS-IDAEA*
Valeriana dioica	*Valeriana dioica*	L.	yes	yes	yes			
Valeriana dioica agg.	*Valeriana dioica*	L.	yes	yes	yes	aggregate on species level		
Valeriana montana	*Valeriana montana*	L.	yes	yes	yes			
Valeriana officinalis	*Valeriana officinalis*	L.	yes	yes	yes			
Valeriana officinalis agg.	*Valeriana officinalis*	L.	yes	yes	yes	aggregate on species level		

Valeriana officinalis subsp. excelsa	*Valeriana officinalis*	L.	yes	yes	yes	aggregate on species level		
Valeriana officinalis subsp. tenuifolia	*Valeriana officinalis*	L.	yes	yes	yes	aggregate on species level		
Valeriana pratensis	*Valeriana pratensis*	Dierb.	yes	yes	no			
Valeriana pratensis subsp. angustifolia	*Valeriana pratensis*	Dierb.	yes	yes	no	aggregate on species level		
Valeriana pratensis subsp. franconica	*Valeriana pratensis*	Dierb.	yes	yes	no	aggregate on species level		
Valeriana procurrens	*Valeriana procurrens*	(Poir.) Rouy & Camus	yes	yes	no			
Valeriana sambucifolia	*Valeriana sambucifolia*	J. C. Mikan ex Pohl	yes	yes	no			
Valeriana tripteris	*Valeriana tripteris*	L.	yes	yes	yes			
Valeriana wallrothii	*Valeriana wallrothii*	Kreyer	yes	yes	no			
Valerianella carinata	*Valerianella carinata*	Loisel.	yes	yes	yes			
Valerianella dentata	*Valerianella dentata*	(L.) Pollich	yes	yes	yes			
Valerianella dentata var. eriosperma	*Valerianella dentata*	(L.) Pollich	yes	yes	yes	aggregate on species level		
Valerianella eriocarpa	*Valerianella eriocarpa*	Desv.	yes	yes	no			
Valerianella locusta	*Valerianella locusta*	(L.) Laterr.	yes	yes	yes			
Valerianella rimosa	*Valerianella rimosa*	Bastard	yes	yes	yes			
Vallisneria spiralis	*Vallisneria spiralis*	L.	yes	yes	no			
Veratrum album	*Veratrum album*	L.	yes	yes	no			
Veratrum album subsp. lobelianum	*Veratrum album*	(Bernh.) Arcang.	yes	yes	no	aggregate on species level		

Verbascum blattaria	*Verbascum blattaria*	L.	yes	yes	yes			
Verbascum densiflorum	*Verbascum densiflorum*	Bertol.	yes	yes	yes			
Verbascum lychnitis	*Verbascum lychnitis*	L.	yes	yes	yes			
Verbascum nigrum	*Verbascum nigrum*	L.	yes	yes	yes			
Verbascum phlomoides	*Verbascum phlomoides*	L.	yes	yes	yes			
Verbascum phoeniceum	*Verbascum phoeniceum*	L.	yes	yes	no			
Verbascum pulverulentum	*Verbascum pulverulentum*	Vill.	yes	yes	yes			
Verbascum speciosum	*Verbascum speciosum*	Schrad.	yes	yes	no		Kumar et al 2022, added to node N6926	
Verbascum thapsus	*Verbascum thapsus*	L.	yes	yes	yes			
Verbascum thapsus subsp. thapsus	*Verbascum thapsus*	L.	yes	yes	yes	aggregate on species level		
Verbena bonariensis	*Verbena bonariensis*	NA	yes	yes	no		Kumar et al 2022, sistertaxon to V. officinalis	
Verbena officinalis	*Verbena officinalis*	L.	yes	yes	yes			
Veronica acinifolia	*Veronica acinifolia*	L.	yes	yes	no			
Veronica agrestis	*Veronica agrestis*	L.	yes	yes	yes			
Veronica anagallis-aquatica	*Veronica anagallis-aquatica*	L.	yes	yes	yes			*VERONICA ANAGALLIS-AQUATICA*

Veronica anagallis-aquatica agg.	*Veronica anagallis-aquatica*	L.	yes	yes	yes	aggregate on species level		
Veronica anagallis-aquatica subsp. anagallis-aquatica	*Veronica anagallis-aquatica*	L.	yes	yes	yes	aggregate on species level		
Veronica anagallis-aquatica subsp. divaricata	*Veronica anagallis-aquatica*	L.	yes	yes	yes	aggregate on species level		
Veronica arvensis	*Veronica arvensis*	L.	yes	yes	yes			
Veronica austriaca	*Veronica austriaca*	L.	yes	yes	yes			
Veronica austriaca agg.	*Veronica austriaca*	L.	yes	yes	yes	aggregate on species level		
Veronica austriaca subsp. austriaca	*Veronica austriaca*	L.	yes	yes	yes	aggregate on species level		
Veronica beccabunga	*Veronica beccabunga*	L.	yes	yes	yes			
Veronica catenata	*Veronica catenata*	Pennell	yes	yes	yes			
Veronica chamaedrys	*Veronica chamaedrys*	L.	yes	yes	yes			
Veronica chamaedrys subsp. chamaedrys	*Veronica chamaedrys*	L.	yes	yes	yes	aggregate on species level		
Veronica chamaedrys subsp. vindobonensis	*Veronica chamaedrys*	L.	yes	yes	yes	aggregate on species level		
Veronica cymbalaria	*Veronica cymbalaria*	Bod.	yes	yes	no			
Veronica filiformis	*Veronica filiformis*	Sm.	yes	yes	yes			
Veronica fruticans	*Veronica fruticans*	Jacq.	yes	yes	yes			
Veronica fruticulosa	*Veronica fruticulosa*	L.	yes	yes	yes			
Veronica hederifolia	*Veronica hederifolia*	L.	yes	yes	yes			

Veronica hederifolia subsp. hederifolia	*Veronica hederifolia*	L.	yes	yes	yes	aggregate on species level		
Veronica hederifolia subsp. lucorum	*Veronica hederifolia*	L.	yes	yes	yes	aggregate on species level		
Veronica hederifolia subsp. triloba	*Veronica hederifolia*	L.	yes	yes	yes	aggregate on species level		
Veronica montana	*Veronica montana*	L.	yes	yes	yes			
Veronica officinalis	*Veronica officinalis*	L.	yes	yes	yes			
Veronica opaca	*Veronica opaca*	Fr.	yes	yes	no			
Veronica peregrina	*Veronica peregrina*	L.	yes	yes	yes			
Veronica persica	*Veronica persica*	Poir.	yes	yes	yes			
Veronica polita	*Veronica polita*	Fr.	yes	yes	yes			
Veronica praecox	*Veronica praecox*	All.	yes	yes	no			
Veronica prostrata	*Veronica prostrata*	L.	yes	yes	no			
Veronica prostrata var. satureiifolia	*Veronica prostrata*	L.	yes	yes	no	aggregate on species level		
Veronica scutellata	*Veronica scutellata*	L.	yes	yes	yes			
Veronica serpyllifolia	*Veronica serpyllifolia*	L.	yes	yes	yes			
Veronica serpyllifolia var. humifusa	*Veronica serpyllifolia*	(Dicks.) Syme	yes	yes	yes	aggregate on species level		
Veronica serpyllifolia var. serpyllifolia	*Veronica serpyllifolia*	NA	yes	yes	yes	aggregate on species level		
Veronica teucrium	*Veronica teucrium*	L.	yes	yes	yes			
Veronica triphyllos	*Veronica triphyllos*	L.	yes	yes	yes			
Veronica urticifolia	*Veronica urticifolia*	Jacq.	yes	yes	no			
Veronica verna	*Veronica verna*	L.	yes	yes	no			
Viburnum lantana	*Viburnum lantana*	L.	yes	yes	yes			

Viburnum opulus	*Viburnum opulus*	L.	yes	yes	yes			
Viburnum rhytidophyllum	*Viburnum rhytidophyllum*	NA	yes	yes	no			
Vicia angustifolia	*Vicia angustifolia*	L.	yes	yes	no			
Vicia angustifolia subsp. angustifolia	*Vicia angustifolia*	L.	yes	yes	no	aggregate on species level		
Vicia angustifolia subsp. segetalis	*Vicia angustifolia*	L.	yes	yes	no	aggregate on species level		
Vicia articulata	*Vicia articulata*	Hornem.	yes	yes	yes			
Vicia bithynica	*Vicia bithynica*	(L.) L.	yes	yes	no			
Vicia cassubica	*Vicia cassubica*	L.	yes	yes	yes			
Vicia cracca	*Vicia cracca*	L.	yes	yes	yes			
Vicia cracca agg.	*Vicia cracca*	L.	yes	yes	yes	aggregate on species level		
Vicia dalmatica	*Vicia dalmatica*	Kern.	yes	yes	yes			*Vicia tenuifolia*
Vicia dumetorum	*Vicia dumetorum*	L.	yes	yes	no			
Vicia faba	*Vicia faba*	L.	yes	yes	yes			
Vicia grandiflora	*Vicia grandiflora*	Scop.	yes	yes	yes			
Vicia hirsuta	*Vicia hirsuta*	(L.) Gray	yes	yes	yes			
Vicia johannis	*Vicia johannis*	Roth	yes	yes	no		*Vicia narbonensis*	
Vicia lathyroides	*Vicia lathyroides*	L.	yes	yes	yes			
Vicia lutea	*Vicia lutea*	L.	yes	yes	no			
Vicia pannonica	*Vicia pannonica*	Crantz	yes	yes	no			
Vicia pannonica subsp. pannonica	*Vicia pannonica*	Crantz	yes	yes	no	aggregate on species level		
Vicia pannonica subsp. striata	*Vicia pannonica*	Crantz	yes	yes	no	aggregate on species level		
Vicia pisiformis	*Vicia pisiformis*	L.	yes	yes	no			
Vicia sativa	*Vicia sativa*	L.	yes	yes	yes			
Vicia sativa agg.	*Vicia sativa*	L.	yes	yes	yes	aggregate on species level		

Vicia sativa subsp. cordata	*Vicia sativa*	L.	yes	yes	yes	aggregate on species level		
Vicia sativa subsp. nigra	*Vicia sativa*	L.	yes	yes	yes	aggregate on species level		
Vicia sativa subsp. sativa	*Vicia sativa*	L.	yes	yes	yes	aggregate on species level		
Vicia sepium	*Vicia sepium*	L.	yes	yes	yes			
Vicia sylvatica	*Vicia sylvatica*	L.	yes	yes	yes			
Vicia tenuifolia	*Vicia tenuifolia*	Roth	yes	yes	yes			
Vicia tetrasperma	*Vicia tetrasperma*	(L.) Schreb.	yes	yes	yes			
Vicia tetrasperma agg.	*Vicia tetrasperma*	(L.) Schreb.	yes	yes	yes	aggregate on species level		
Vicia villosa	*Vicia villosa*	Roth	yes	yes	yes			
Vicia villosa subsp. pseudocracca	*Vicia villosa*	Roth	yes	yes	yes	aggregate on species level		
Vicia villosa subsp. varia	*Vicia villosa*	Roth	yes	yes	yes	aggregate on species level		
Vicia villosa subsp. villosa	*Vicia villosa*	Roth	yes	yes	yes	aggregate on species level		
Vinca major	*Vinca major*	L.	yes	yes	no			
Vinca minor	*Vinca minor*	L.	yes	yes	yes			
Vincetoxicum hirundinaria	*Vincetoxicum hirundinaria*	Medik.	yes	yes	yes			
Viola alba	*Viola alba*	Besser	yes	yes	yes			
Viola alba subsp. alba	*Viola alba*	Besser	yes	yes	yes	aggregate on species level		
Viola alba subsp. scotophylla	*Viola alba*	Besser	yes	yes	yes	aggregate on species level		
Viola arvensis	*Viola arvensis*	Murray	yes	yes	yes			

Viola arvensis subsp. arvensis	*Viola arvensis*	Murray	yes	yes	yes	aggregate on species level		
Viola arvensis subsp. megalantha	*Viola arvensis*	Murray	yes	yes	yes	aggregate on species level		
Viola biflora	*Viola biflora*	L.	yes	yes	yes			
Viola canina	*Viola canina*	L.	yes	yes	yes			
Viola canina agg.	*Viola canina*	L.	yes	yes	yes	aggregate on species level		
Viola canina subsp. canina	*Viola canina*	L.	yes	yes	yes	aggregate on species level		
Viola canina subsp. montana	*Viola canina*	L.	yes	yes	yes	aggregate on species level		
Viola canina subsp. schultzii	*Viola canina*	L.	yes	yes	yes	aggregate on species level		
Viola collina	*Viola collina*	L.	yes	yes	no			
Viola elatior	*Viola elatior*	Fr.	yes	yes	no			
Viola hirta	*Viola hirta*	L.	yes	yes	yes			
Viola mirabilis	*Viola mirabilis*	L.	yes	yes	yes			
Viola odorata	*Viola odorata*	L.	yes	yes	yes			
Viola palustris	*Viola palustris*	L.	yes	yes	yes			
Viola persicifolia	*Viola persicifolia*	Kit. ex Schult.	yes	yes	yes		*Viola stagnina*	
Viola pumila	*Viola pumila*	Chaix	yes	yes	no			
Viola reichenbachiana	*Viola reichenbachiana*	Boreau	yes	yes	yes			
Viola reichenbachiana agg.	*Viola reichenbachiana*	Boreau	yes	yes	yes	aggregate on species level		
Viola riviniana	*Viola riviniana*	Rchb.	yes	yes	yes			
Viola riviniana agg.	*Viola riviniana*	Rchb.	yes	yes	yes	aggregate on species level		
Viola rupestris	*Viola rupestris*	F. W. Schmidt	yes	yes	yes			

Viola scabra	*Viola scabra*	F. Braun	yes	yes	no		*Viola x scabra*	
Viola suavis	*Viola suavis*	M. Bieb.	yes	yes	no			
Viola tricolor	*Viola tricolor*	L.	yes	yes	yes			
Viola tricolor agg.	*Viola tricolor*	L.	yes	yes	yes	aggregate on species level		
Viola tricolor subsp. saxatilis	*Viola tricolor*	L.	yes	yes	yes	aggregate on species level		
Viola tricolor subsp. tricolor	*Viola tricolor*	L.	yes	yes	yes	aggregate on species level		
Viola wittrockiana	*Viola wittrockiana*	L.	yes	yes	no			
Viola wittrockiana	*Viola wittrockiana*	Gams	yes	yes	no			
Viscum album	*Viscum album*	L.	yes	yes	yes			
Viscum album subsp. abietis	*Viscum album*	L.	yes	yes	yes	aggregate on species level		
Viscum album subsp. album	*Viscum album*	L.	yes	yes	yes	aggregate on species level		
Viscum album subsp. austriacum	*Viscum album*	L.	yes	yes	yes	aggregate on species level		
Vitis vinifera	*Vitis vinifera*	L.	yes	yes	yes			
Vitis vinifera subsp. sylvestris	*Vitis vinifera*	(C. C. Gmel.) Beck	yes	yes	yes	aggregate on species level		
Vitis vinifera subsp. vinifera	*Vitis vinifera*	L.	yes	yes	yes	aggregate on species level		
Vulpia bromoides	*Vulpia bromoides*	(L.) Gray	yes	yes	yes			
Vulpia ciliata	*Vulpia ciliata*	Dumort.	yes	yes	no			
Vulpia myuros	*Vulpia myuros*	(L.) C. C. Gmel.	yes	yes	no			
Wahlenbergia hederacea	*Wahlenbergia hederacea*	(L.) Rchb.	yes	yes	yes			

Waldsteinia ternata	*Waldsteinia ternata*	(Stephan) Fritsch	yes	yes	no		Kumar et al 2022, sistertaxon to genus Geum	
Willemetia stipitata	*Willemetia stipitata*	(Jacq.) Rauschert	yes	yes	no			
Woodsia ilvensis	*Woodsia ilvensis*	(L.) R. Br.	yes	yes	no			
Xanthium albinum	*Xanthium albinum*	(Widder) H. Scholz	yes	yes	no			
Xanthium albinum subsp. albinum	*Xanthium albinum*	(Widder) H. Scholz	yes	yes	no	aggregate on species level		
Xanthium orientale agg.	*Xanthium orientale*	L.	yes	yes	no	aggregate on species level		
Xanthium strumarium	*Xanthium strumarium*	L.	yes	yes	yes			
Yucca filamentosa	*Yucca filamentosa*	L.	yes	yes	no		Kumar et al 2022, sistertaxon to Agava americana	
Zannichellia palustris	*Zannichellia palustris*	L.	yes	yes	no			
Zannichellia palustris subsp. palustris	*Zannichellia palustris*	L.	yes	yes	no	aggregate on species level		
