# Supplementary material for: Five Decades of Citizen Science Reveal Erosion of Hotspots of Multifaceted Plant Diversity
Source: Glob Chang Biol. 2026 Oct 27;32(10):e71150. doi: 10.1111/gcb.71150 (PMC13711242; doi:10.1111/gcb.71150)
Supplement: Supplementary file 3 [file GCB-32-e71150-s003.pdf]

Supporting Information 3

Five decades of Citizen Science reveal erosion of hotspots of multifaceted plant diversity

Viktoria Ferenc*[1,2,3], viktoria.ferenc@smns-bw.de, +49 711-8936203
Cornelia Krause[1,3], cornelia.krause@smns-bw.de
Jörn Pagel[2,3], joern.pagel@uni-hohenheim.de
Michael Boxriker[1,3], michael.boxriker@smns-bw.de
Monika Voggesberger[1], monika.voggesberger@smns-bw.de
Arno Wörz[1], awoerz@gmx.de
Mike Thiv[1,3], mike.thiv@smns-bw.de
Pierre Liancourt[1,3,4], pierre.liancourt@smns-bw.de

[1]Department of Botany, State Museum of Natural History, Stuttgart, Germany
[2]Institute of Landscape and Plant Ecology, University of Hohenheim, Stuttgart, Germany
[3]KomBioTa – Center for Biodiversity and Integrative Taxonomy, University of Hohenheim & State Museum of Natural History, Stuttgart, Germany
[4]Plant Ecology, University of Tübingen, Tübingen, Germany

*Corresponding author

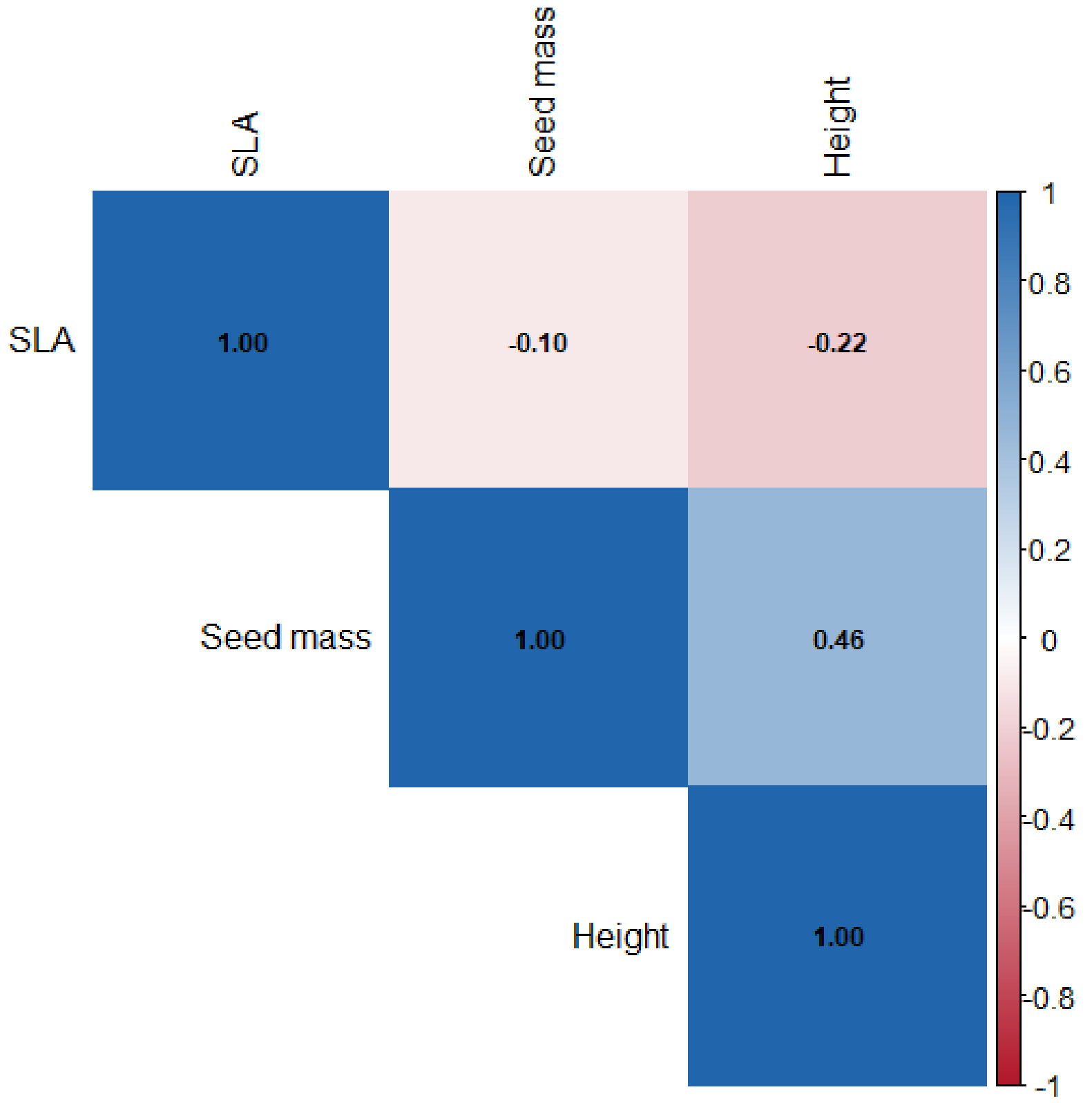

Figure S5. Pearson's correlation matrix of the used traits.

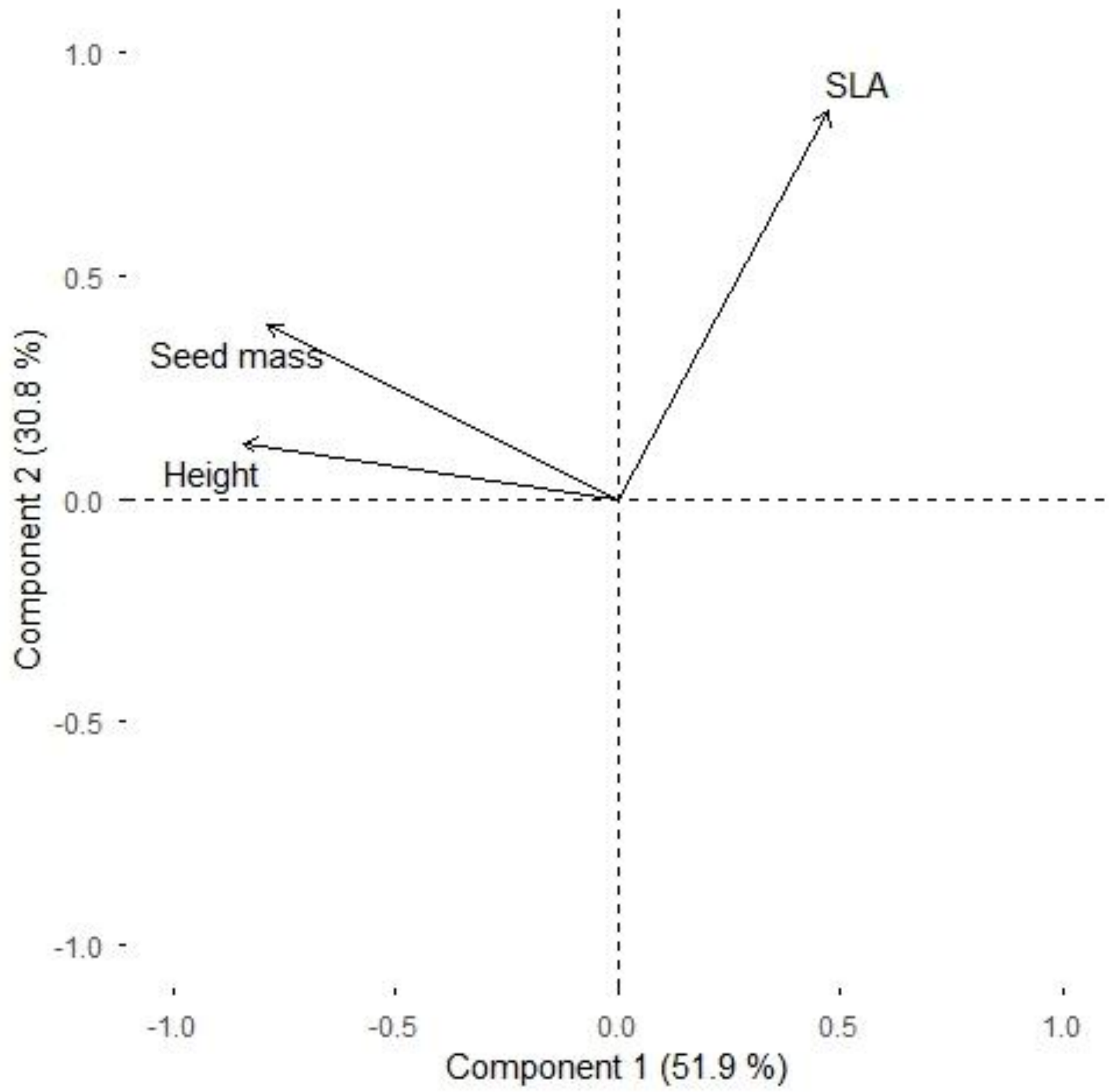

Figure S6. PCA of the used traits.

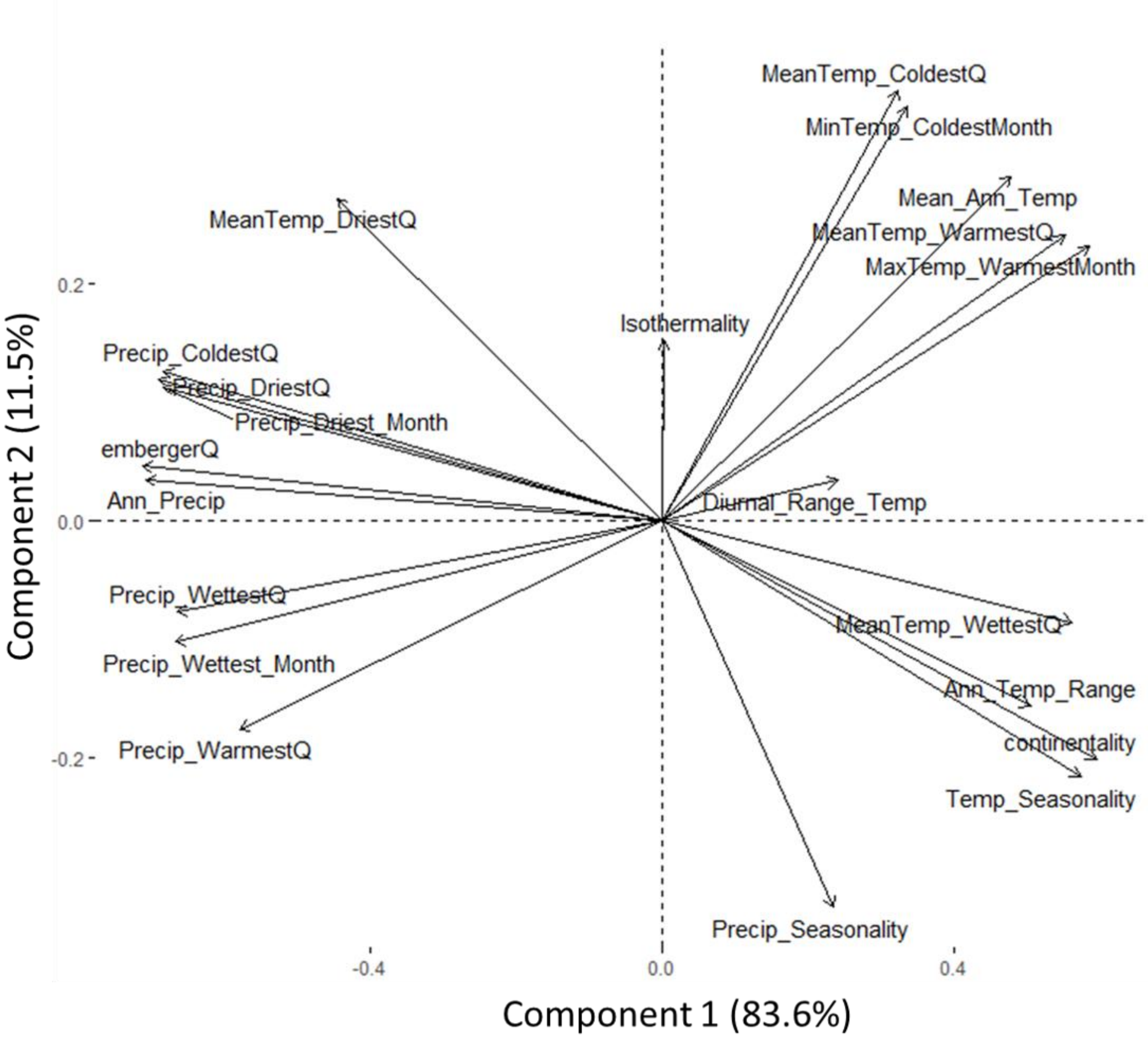

Figure S7. PCA of all 21 climate variables.

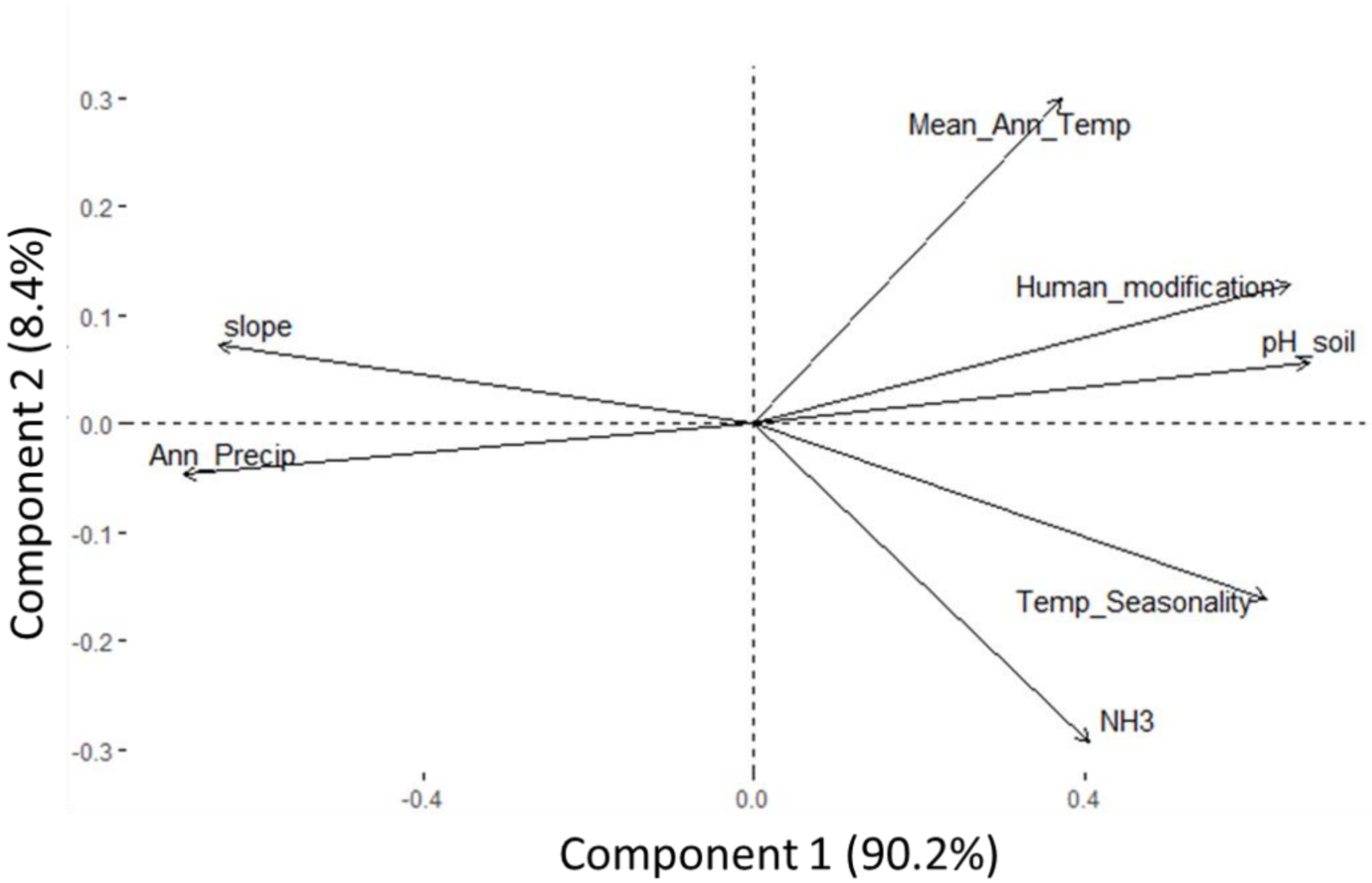

Figure S8. PCA of all used environmental variables.

Table S1. List of the 19 bioclimatic variables provided by WorldClim 2 (Fick & Hijmans, 2017).

Bioclim variable	Description
**BIO1**	Annual Mean Temperature
**BIO2**	Mean Diurnal Range (Mean of monthly (max temp - min temp))
**BIO3**	Isothermality (BIO2/BIO7) (×100)
**BIO4**	Temperature Seasonality (standard deviation ×100)
**BIO5**	Max Temperature of Warmest Month
**BIO6**	Min Temperature of Coldest Month
**BIO7**	Temperature Annual Range (BIO5-BIO6)
**BIO8**	Mean Temperature of Wettest Quarter
**BIO9**	Mean Temperature of Driest Quarter
**BIO10**	Mean Temperature of Warmest Quarter
**BIO11**	Mean Temperature of Coldest Quarter
**BIO12**	Annual Precipitation
**BIO13**	Precipitation of Wettest Month
**BIO14**	Precipitation of Driest Month
**BIO15**	Precipitation Seasonality (Coefficient of Variation)
**BIO16**	Precipitation of Wettest Quarter
**BIO17**	Precipitation of Driest Quarter
**BIO18**	Precipitation of Warmest Quarter
**BIO19**	Precipitation of Coldest Quarter

Table S2. Loadings of the Δ diversity metrics in the three components after applying a varimax rotation to the corresponding PCAs. Numbers depicted in bold indicate the highest contribution of the Δ diversity metric.

Diversity metric	Component 1	Component 2	Component 3
**Δ FRic**	**0.85**	0.14	0.40
**Δ FEve**	**-1.00**		0.18
**Δ FDiv**	0.48		**0.61**
**Δ FDis**		0.22	**0.93**
**Δ MPD**	0.21	**0.78**	0.20
**Δ MNTD**		**0.80**	0.28
**Δ Species Richness**	0.13	**-0.76**	0.29

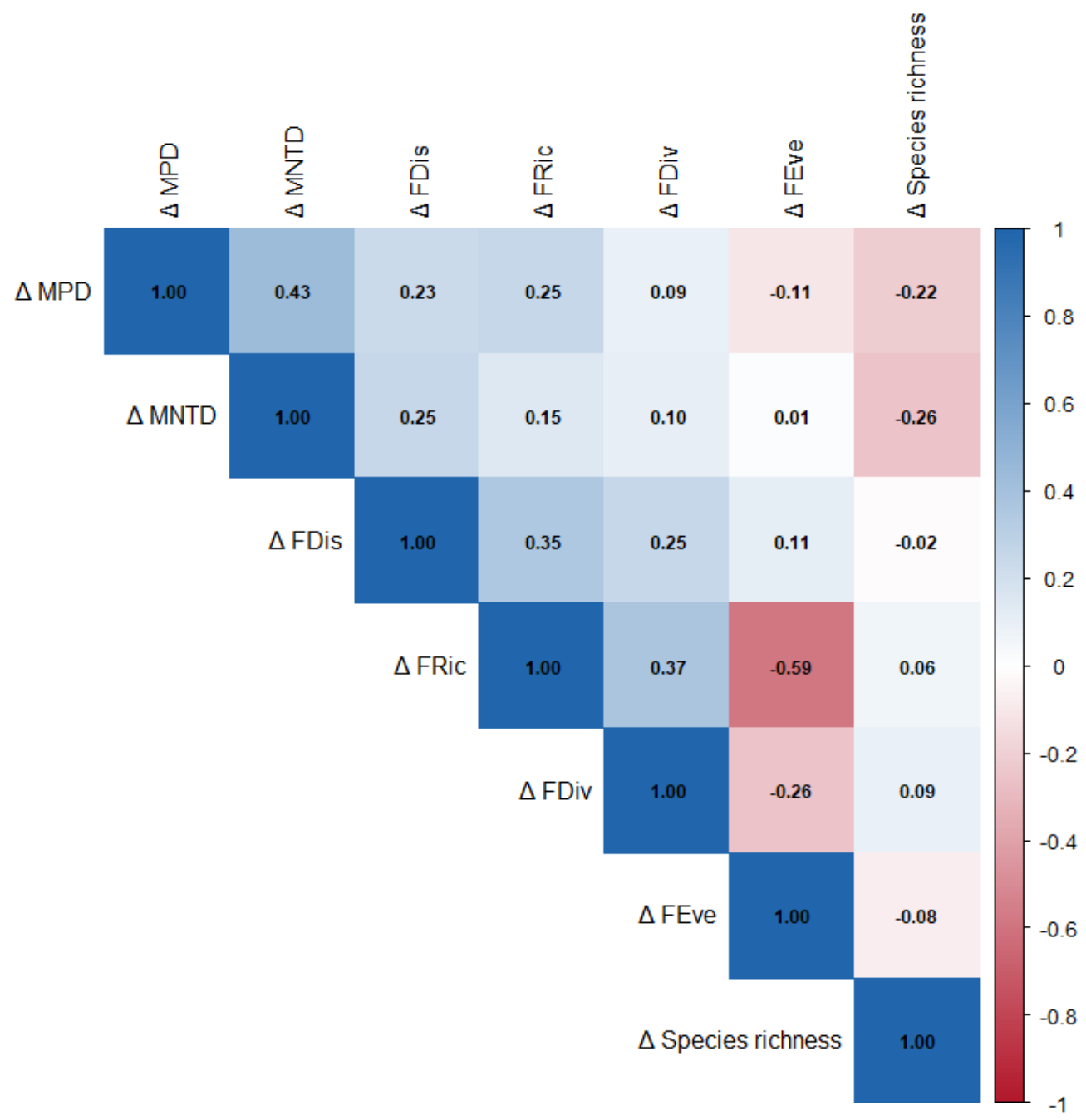

Figure S9. Pearson's correlation matrix of the Δ diversity metrics.

Table S3. Variance Inflation Factor (VIF) for all variables retained in the respective model.

	**Δ Species Richness**	**Δ MPD**	**Δ MNTD**	**Δ FRic**	**Δ FEve**	**Δ FDiv**	**Δ FDis**
TS	4.87	4.91	4.57	-	-	4.52	3.16
MAT	-	2.16	1.97	2.03	2.05	1.93	2.05
MAP	5.36	6.53	5.58	3.91	3.51	5.35	-
HM	3.27	6.29	5.84	-	5.55	5.67	3.06
NH3	2.51	2.88	2.67	1.61	1.56	2.60	2.22
Soil pH	-	7.71	6.19	4.31	6.21	6.04	-
Slope	2.70	2.85	2.68	2.43	-	2.59	2.64
Initial	1.42	3.85	1.48	1.41	1.21	1.11	1.13

Table S4. Species that have increased most between the first and second mapping.

Species name	Occurrence second mapping	Occurrence first mapping	Δ Species occurrence
Herniaria glabra	740	128	612
Hieracium aurantiacum	643	159	484
Eragrostis minor	743	291	452
Oxalis corniculata	710	303	407
Pseudotsuga menziesii	444	69	375
Portulaca oleracea	538	172	366
Lysimachia punctata	512	150	362
Sedum sexangulare	652	298	354
Buddleja davidii	448	98	350
Juglans regia	705	357	348
Muscari armeniacum	347	13	334
Lathyrus latifolius	452	120	332
Mahonia aquifolium	380	49	331
Dryopteris affinis	473	149	324
Sedum spurium	475	152	323
Erigeron annuus	835	513	322
Cardamine hirsuta	809	500	309
Galanthus nivalis	416	108	308
Sedum album	739	435	304
Aquilegia vulgaris	705	406	299
Senecio inaequidens	380	82	298
Setaria pumila	583	305	278
Echinochloa crus-galli	717	452	265
Sedum rupestre	483	219	264
Melissa officinalis	298	35	263
Prunus cerasifera	278	25	253
Aesculus hippocastanum	608	358	250
Prunus laurocerasus	268	19	249
Oenothera biennis	653	412	241
Duchesnea indica	274	33	241
Sedum hispanicum	281	43	238
Parthenocissus inserta	328	97	231
Impatiens glandulifera	741	512	229
Epilobium ciliatum	594	371	223
Chamaesyce maculata	374	157	217
Cerastium glomeratum	739	524	215
Lactuca serriola	855	643	212
Arctium lappa	651	448	203
Taxus baccata	316	115	201
Digitaria sanguinalis	561	361	200
Valerianella locusta	659	463	196
Solidago gigantea	644	451	193
Rhus hirta	267	76	191
Hieracium piloselloides	498	308	190
Malva moschata	705	515	190
Anthemis tinctoria	365	177	188
Impatiens parviflora	780	593	187
Viola wittrockiana	193	8	185
Vinca minor	730	547	183
Panicum capillare	209	28	181

Table S5. Species that have decreased most between the first and second mapping.

Species name	Occurrence second mapping	Occurrence first mapping	Δ Species occurrence
Dactylorhiza maculata	221	546	-325
Raphanus raphanistrum	417	733	-316
Vicia sativa	273	579	-306
Listera ovata	490	788	-298
Gymnadenia conopsea	315	563	-248
Carex panicea	343	584	-241
Orchis mascula	206	444	-238
Dactylorhiza majalis	269	505	-236
Rosa villosa	46	279	-233
Succisa pratensis	282	509	-227
Platanthera chlorantha	222	449	-227
Chenopodium bonus-henricus	265	491	-226
Epipactis purpurata	335	554	-219
Salix fragilis	462	676	-214
Mentha arvensis	540	753	-213
Platanthera bifolia	314	526	-212
Carex nigra	272	484	-212
Bidens tripartita	80	288	-208
Callitriche palustris	286	492	-206
Carex rostrata	218	418	-200
Orchis morio	118	317	-199
Crepis paludosa	335	530	-195
Carex montana	382	577	-195
Orchis militaris	252	446	-194
Apera spica-venti	426	620	-194
Neottia nidus-avis	485	678	-193
Euphrasia officinalis	327	517	-190
Salix triandra	213	400	-187
Gentianella ciliata	199	384	-185
Gnaphalium sylvaticum	191	374	-183
Carex leporina	405	587	-182
Carex umbrosa	211	393	-182
Sparganium erectum	326	507	-181
Valeriana dioica	428	608	-180
Koeleria pyramidata	303	480	-177
Avena fatua	426	602	-176
Ranunculus nemorosus	159	334	-175
Euphorbia exigua	438	613	-175
Carex acuta	298	471	-173
Pyrus communis	205	376	-171
Gentianella germanica	126	296	-170
Silene noctiflora	214	382	-168
Sonchus arvensis	524	691	-167
Betonica officinalis	511	676	-165
Valeriana officinalis	736	900	-164
Molinia caerulea	535	699	-164
Sinapis arvensis	622	785	-163
Cephalanthera rubra	242	405	-163
Maianthemum bifolium	496	659	-163
Ranunculus arvensis	127	289	-162
